# Chromatin accessibility and regulatory vocabulary across indicine cattle tissues

**DOI:** 10.1186/s13059-021-02489-7

**Published:** 2021-09-21

**Authors:** Pâmela A. Alexandre, Marina Naval-Sánchez, Moira Menzies, Loan T. Nguyen, Laercio R. Porto-Neto, Marina R. S. Fortes, Antonio Reverter

**Affiliations:** 1 CSIRO Agriculture & Food, 306 Carmody Rd., QLD 4067 Brisbane, Australia; 2 Institute for Molecular Bioscience, The University of Queensland, Brisbane, QLD 4072 Australia; 3 Queensland Alliance for Agriculture and Food Innovation, The University of Queensland, Brisbane, QLD 4072 Australia; 4 School of Chemistry and Molecular Biosciences, The University of Queensland, Brisbane, QLD 4072 Australia

**Keywords:** *Bos indicus*, ATAC-seq, Motif discovery, Open chromatin region

## Abstract

**Background:**

Spatiotemporal changes in the chromatin accessibility landscape are essential to cell differentiation, development, health, and disease. The quest of identifying regulatory elements in open chromatin regions across different tissues and developmental stages is led by large international collaborative efforts mostly focusing on model organisms, such as ENCODE. Recently, the Functional Annotation of Animal Genomes (FAANG) has been established to unravel the regulatory elements in non-model organisms, including cattle. Now, we can transition from prediction to validation by experimentally identifying the regulatory elements in tropical indicine cattle. The identification of regulatory elements, their annotation and comparison with the taurine counterpart, holds high promise to link regulatory regions to adaptability traits and improve animal productivity and welfare.

**Results:**

We generate open chromatin profiles for liver, muscle, and hypothalamus of indicine cattle through ATAC-seq. Using robust methods for motif discovery, motif enrichment and transcription factor binding sites, we identify potential master regulators of the epigenomic profile in these three tissues, namely HNF4, MEF2, and SOX factors, respectively. Integration with transcriptomic data allows us to confirm some of their target genes. Finally, by comparing our results with Bos taurus data we identify potential indicine-specific open chromatin regions and overlaps with indicine selective sweeps.

**Conclusions:**

Our findings provide insights into the identification and analysis of regulatory elements in non-model organisms, the evolution of regulatory elements within two cattle subspecies as well as having an immediate impact on the animal genetics community in particular for a relevant productive species such as tropical cattle.

**Supplementary Information:**

The online version contains supplementary material available at 10.1186/s13059-021-02489-7.

## Background

Chromatin is a complex of DNA and proteins (nucleosomes) found in the nucleus of eukaryotic cells. The non-uniform topological organization of nucleosomes across the genome, as well as their post-translational modifications, reflects a dynamic process that controls chromatin accessibility, switching between transcriptionally active euchromatin and inactive heterochromatin [1]. The landscape of chromatin accessibility regulates the degree to which nuclear macromolecules can bind the double helix, thus affecting transcription, DNA repair, replication, and recombination [2]. Nucleosomes are known to be depleted at regulatory loci, including enhancers, insulators, and transcribed gene bodies, making binding sites available to transcription factors (TFs) and the transcription machinery [3]. These epigenetic changes are instrumental to cell differentiation, environmental signaling, and disease development. The quest of identifying regulatory elements in open chromatin regions across different tissues and developmental stages is led by large international consortia mostly focussing on model organisms, such as the Encyclopedia of DNA Elements (ENCODE) in humans [4, 5], the mouseENCODE for mouse [6] and the modENCODE for fruitfly and *C. elegans* [7, 8].

Recently, the Functional Annotation of Animal Genomes (FAANG [9]) and its counterpart, Functional Annotation of All Salmonid Genomes (FAASG [10]), have been established with the aim to unravel the regulatory elements in non-model organisms, including chicken, pig, cattle, ovine, and aquaculture species. In this context, our group has contributed the first draft of cattle and sheep functional regulatory regions based on the identification of orthologous regulatory regions [11, 12] from human and mouse [4, 6, 13]. However, particularly in cattle, the possibility of investigating chromatin accessibility sheds light on the expected differences between the two subspecies, *Bos taurus indicus* and *Bos taurus taurus* [14, 15]. Indicine (or zebu) breeds (*B. indicus*) are highly adapted to tropical environments, including resistance to disease and parasites, heat stress, and severe drought conditions. Considering more than half of livestock heads are found in tropical and subtropical environments [16], understanding and selecting animals for adaptability traits is of high economic and welfare relevance. The functional genomic basis of climatic adaptation in beef cattle is not well understood, and resolving tissue-specific deployment of regulatory activity directed by small sequences is paramount.

In the quest of detecting chromatin accessibility, the Assay of Transposase Accessible Chromatin sequencing (ATAC-seq) has become increasingly popular [1]. The libraries for ATAC-seq are constructed by incorporating a hyperactive Tn5 transposase that simultaneously cuts open chromatin on both ends, leaving a 9 bp staggered nick. Then, high-throughput sequencing adapters are ligated to these regions [17]. PCR is used for library construction, followed by paired-end next-generation sequencing. This simple and fast protocol, paired with its high sensitivity and low requirement for starting cell number, are the reasons for the popularity of this assay [1]. Recently, ATAC-seq data has been used to annotate and compare domesticated farm animals, namely bovine (*B. taurus*), chicken, goat, and pig [18, 19]. Here, we generated open chromatin profiles for three tissues (liver, muscle, and hypothalamus) of tropical cattle (*B. t. indicus*) through ATAC-seq. The liver was chosen for being a central organ of metabolism, including bilirubin, bile acids, carbohydrates, lipids, xenobiotics, protein synthesis, and immunity [20]. Similarly, the hypothalamus is a representative of the neuroendocrine system involved in the regulation of several body processes, such as stress reaction, digestion, immunity, behavior, sexual behavior, and energy storage and expenditure. Finally, the skeletal muscle was chosen for being the ultimate focus for beef cattle production.

Using several bioinformatics approaches, such as motif discovery, as well as publicly available datasets, we aimed to functionally characterize regulatory elements in indicine tissues as well as their underlying regulatory code. That is the combination of transcription factor binding sites (TFBS) that govern the spatiotemporal regulatory activity and gene expression. This improved knowledge of regulatory annotation sheds high promise in linking sequence to phenotype and posing new questions on our current understanding of productive traits of agricultural relevance.

## Results

### Regulatory landscapes across three tissues in tropical cattle

To annotate regulatory elements in tropical cattle, we generated ATAC-seq data from liver, hypothalamus, and muscle of three post-puberty Brahman heifers [21–23]. After quality control, samples resulted in an average of 82,988,361 uniquely mapped reads (Additional file 1), in agreement with the quality standards determined by ENCODE ATAC-seq pipeline [24].

Open chromatin regions were identified through consensus peaks across biological replicates in each tissue, resulting in 78,528 peaks for hypothalamus, 40,104 peaks for muscle, and 22,291 peaks for liver (Additional files 2-4); covering 2.41%, 0.98%, and 0.52% of the genome, respectively (Additional file 5). The average peak length was 836 bp, 667 bp, and 635 bp for hypothalamus, muscle, and liver, respectively (Table 1).

**Table 1:** Peak calling metrics

	Total peaks identified	Consensus peaks (P < 0.01)	Average peak length (bp)	Peaks on chr1-29 and X	Proportion of peaks near TSS (±3Kb, %)
Hypothalamus	212,636,473	78,528	836	71,028	18.07
Liver	285,783,943	22,291	635	12,063	50.54
Muscle	248,240,326	40,104	667	30,483	30.95
Hypothalamus-specific	-	53,289	630	53,103	9.08
Liver-specific	-	2213	361	938	9.49
Muscle-specific	-	11,439	474	10,976	7.56
Constitutive	-	11,983	578	9803	59.37

**Fig. 1:**
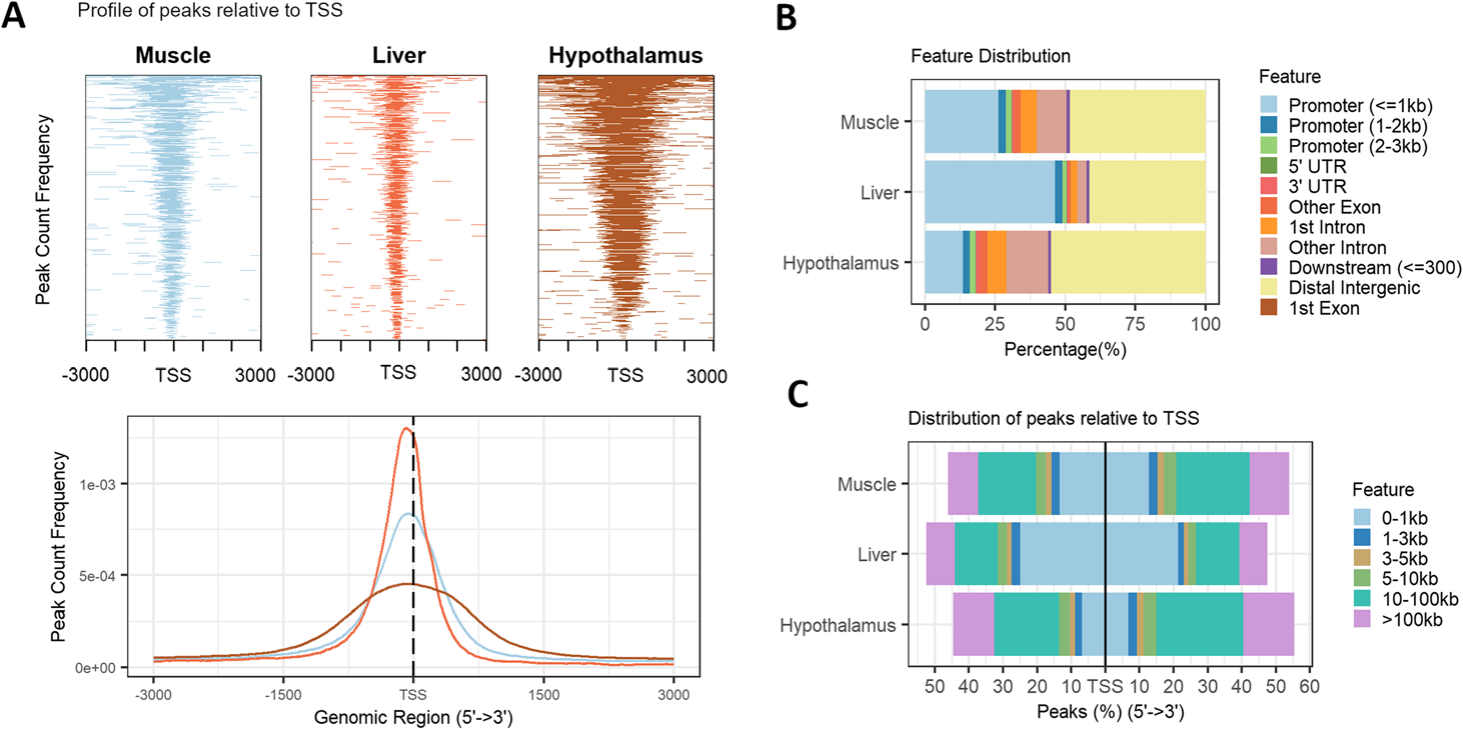
Comparison of ATAC-seq peaks across different tissues (considering chromosomes 1-29 and X). **A** Profile of peaks relative to transcription start sites (TSS), considering a ± 3 kb region, for individual tissues (top heatmap) and comparing tissue average profiles (bottom distribution). **B** Percentage of overlap between peaks and genomic features. **C** Percentage of peaks upstream and downstream from the TSS of their nearest genes

A signal of good quality in ATAC-seq data is to present enrichment for transcription start sites (TSS), which can be seen in Fig. 1A. However, open chromatin regions can be mapped into different functional categories, including gene bodies, promoters, and distal regulatory elements (Fig. 1B). Most of our called peaks fall within distal intergenic (41-55%), followed by promoter regions (18–50%). A small percentage of peaks fall within exons (0.8–3%) and untranslated regions (UTR, < 1%). Although peaks are assigned to the most representative genomic feature to allow for easy comparison across tissues, often the same peak can span multiple features, which was captured in Additional file 6. It is noteworthy that samples with a lower number of peaks present a higher percentage of peaks within Promoter/TSS regions. This behavior is also observed for the distribution of peaks in terms of distance to the TSS of the nearest gene (Fig. 1C).

### Genome-wide differences in chromatin accessibility profiles across tissues

**Fig. 2:**
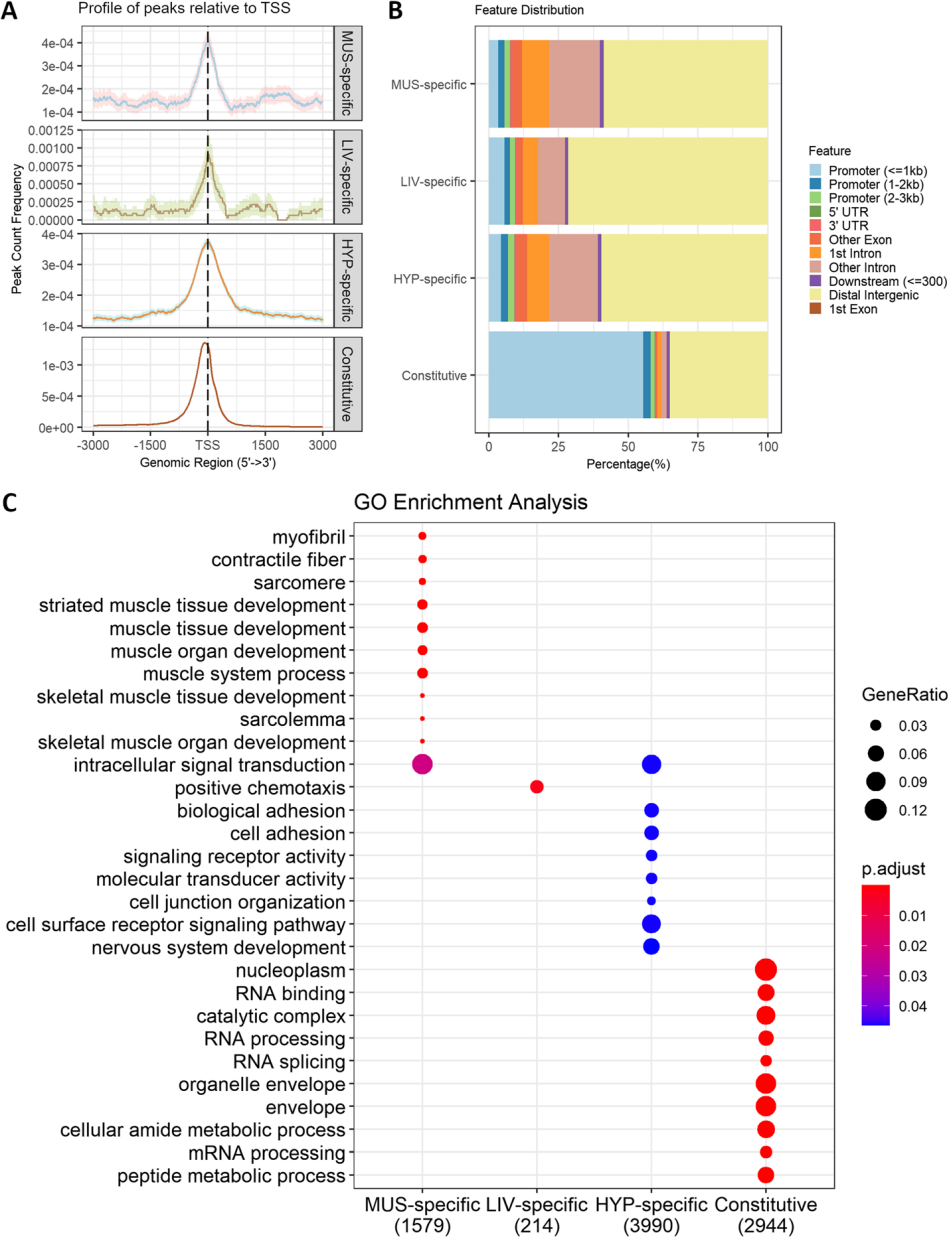
Comparison between tissue-specific (TS) peaks and constitutive regions for muscle (MUS), liver (LIV), and hypothalamus (HYP). **A** Profile of peaks relative to transcription start sites (TSS) considering a ± 3 kb region—confidence intervals were estimated by bootstrap method (500 iterations) and is shown as the shading that follows each curve. **B** Percentage of overlap between peaks and genomic features. **C** Functional enrichment of top 10 gene ontology (GO) terms for genes associated to peaks

The identified peaks were compared between tissues and tissue-specific peaks were defined. Conversely, overlapping regions in all three tissues were considered constitutive. We were able to identify 2213, 11,439, and 53,289 tissue-specific peaks for liver, muscle, and hypothalamus, respectively, and 11,983 constitutive regions (Additional files 2-4, 7). For the four subsets, an enrichment around TSS can still be seen (Fig. 2A). However, while more than half of constitutive regions lie in promoter regions and gene bodies, most tissue-specific peaks fall into intergenic and intronic regions (Fig. 2B).

Assigning peaks to genomic features invites relating those peaks to the nearest annotated gene and, therefore, identifying key biological functions associated with open chromatin regions (Fig. 2C, Additional file 8). Muscle-specific peaks were associated mostly with terms related to muscle tissue development (Padj = 2.13E−06) and muscle system process (Padj = 1.12E−05). Hypothalamus presented enrichment for terms related to cell communication such as cell surface receptor signaling pathway (Padj = 0.04) and for nervous system development (Padj = 0.04). Liver only presented enrichment for positive chemotaxis (Padj = 1.10E−06) which might be related to the movement of immune-competent cells characteristics of this tissue. Finally, constitutive regions were mostly related to RNA processing (Padj = 5.93E−10), translation (Padj = 1.54E−06), and protein catabolic process (Padj = 1.14E−06).

### Motif discovery unravels master tissue-specific regulators

**Fig. 3:**
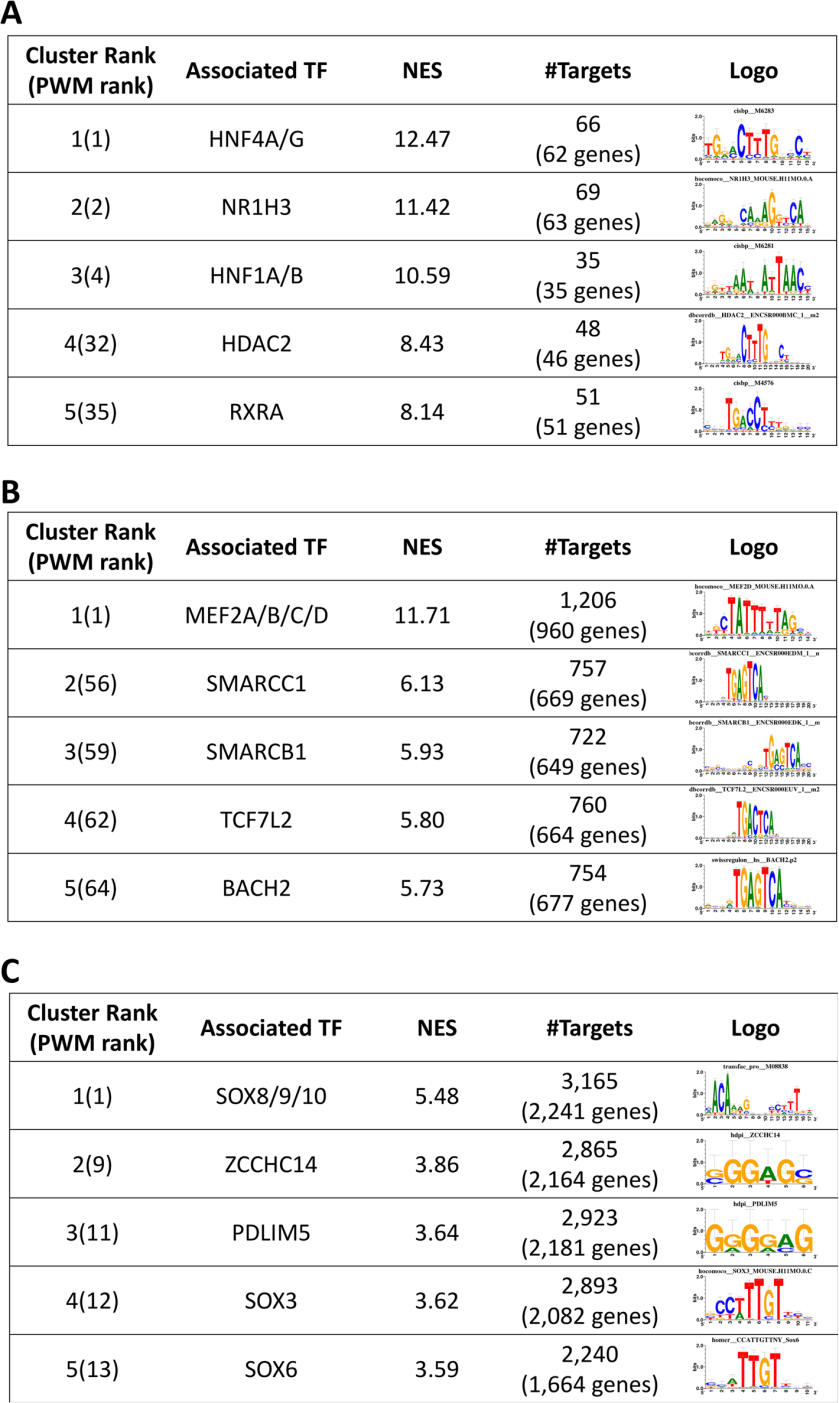
Top 5 iRegulon motif discovery results on liver-specific (**A**), muscle-specific (**B**), and hypothalamus-specific (**C**) open chromatin regions

The 2213 liver-specific ATAC-seq peaks were converted into 546 human genome regions which were enriched for master regulators of liver and hepatocyte differentiation, namely hepatocyte nuclear factors HNF4A/G (normalized enrichment score—NES = 12.47), and HNF1A/B (NES = 10.59) (Fig. 3A, Additional file 9). The enrichment analysis of our liver-specific regions against a public TF ChIP-seq bound regions database in human cell lines from ENCODE confirmed the experimental binding of HNF4G on human HepG2 cells as the most enriched track (ENCFF001UGI, NES = 7.39), followed by RXRA (ENCFF001UHJ, NES = 6.81) and HNF4A (ENCFF001UGH, NES = 6.79; ENCFF001UGG, NES = 6.78). Using the same methodology, we confirmed our liver-specific regions were enriched for open chromatin in hepatocyte cell lines from ENCODE, namely H3K27ac in HEpG2 Hepatocellular carcinoma cell line (NES = 8.55) and FAIRE-seq on HepG2 (ENCFF001UYN, NES = 8.55), thus strongly indicating our regions are functionally active in hepatocytes. When we converted the predicted target regions of HNF4 back to cattle coordinates and compared them with our open chromatin regions in liver, we were able to annotate 27 possible binding sites, with scores (log likelihood ratios) varying from 0.03 to 12.5 (Additional file 10). No exact score threshold exists and therefore, we reported scores for all identified target regions. Nevertheless, the higher the score the better.

**Fig. 4:**
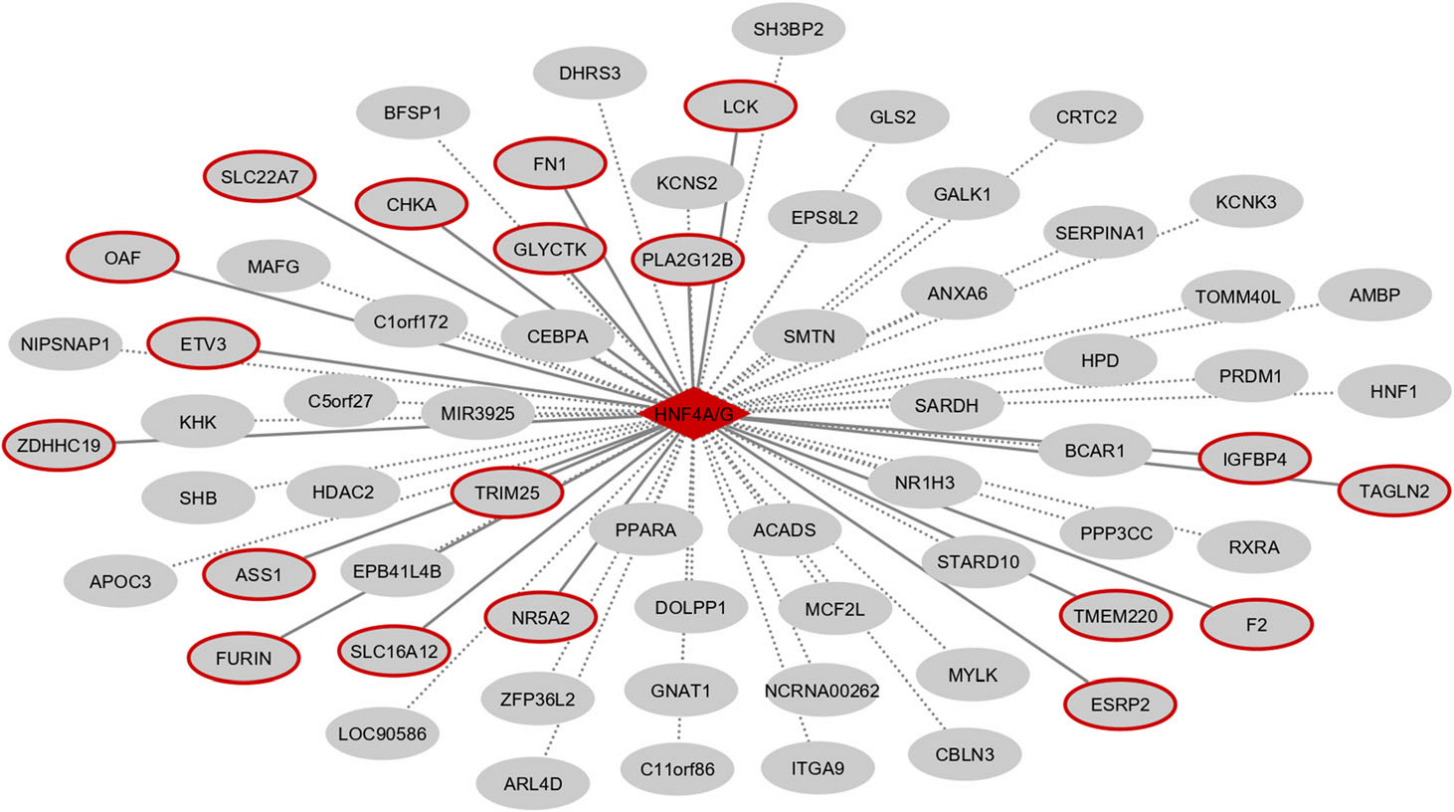
Liver-specific master regulator HNF4 and its predicted targets. Dotted edges represent predicted targets, continuous edges and red borders represent targets with significant co-expression using RNA-seq data

Finally, we validated the predicted targets of HNF4 by testing their co-expression based on RNA-seq data. From the 62 predicted HNF4 targets, 52 were considered expressed in our liver data. In addition, except for HNF1B, all top TF enriched in liver presented gene expression and were included in the co-expression analysis. From 52 expressed targets, 20 (38%) presented significant co-expression with HNF4 (Fig. 4, Additional file 11). Also, HNF1A was the only top TF co-expressed with HNF4.

The 11,439 muscle-specific ATAC-seq peaks were converted into 10,067 human genome regions which were enriched for a family of master regulators of muscle differentiation (Fig. 3B, Additional file 12), namely myocyte enhancer factor-2 (MEF2, NES = 11.71). To validate our predictions, we looked at the enrichment for ENCODE ChIP-seq experiments which resulted in skeletal muscle cell lines both in male (E107-H3K4me1, NES = 8.44; E107-H3K4me1-broadpeak, NES = 5.52) and female (E108-H3K27ac, NES = 7.50; E108-H3K4me1, NES = 7.04) and FAIRE-seq on the skeletal myoblasts cell line LHCN-M2 (ENCFF001WPB, NES = 5.23) as the most enriched tracks. These results confirm our muscle-specific regions are indeed functionally active in muscle cells. By converting the predicted target regions of MEF2 back to cattle coordinates and comparing them with our open chromatin regions in muscle, we identified 667 possible binding sites, with scores varying from 3.25E−03 to 23.4 (Additional file 13).

The 53,289 hypothalamus-specific ATAC-seq peaks were converted into 48,067 human genome regions which were enriched for an important family of transcription factors for neuronal development (Fig. 3C, Additional file 14), namely SRY-related HMG box genes (SOX, NES = 5.48). The SOX family can regulate several different aspects of development in general, which explains the enrichment for FAIRE-seq for Foreskin Melanocyte Primary Cells as the top enriched track (E059-DNase.hotspot.all.peaks-narrowpeak NES = 6.98) and DNase-seq on human iPS (ENCFF001SPB, NES = 5.96) as the third. Nevertheless, among the top 10 enriched tracks are ENCODE ChIP-seq results for Brain Inferior Temporal Lobe (E072-H3K27ac, NES = 5.97; E072-H3K4me1, NES = 5.32), Brain Substantia Nigra (E074-H3K27ac, NES = 5.80; E074-H3K4me1, NES = 5.62), and Brain Hippocampus Middle (E071-H3K4me1, NES = 5.41). SOX targets coordinate when compared with our cattle open chromatin regions in hypothalamus, represented 2166 possible binding sites, with scores varying from 4.05E−06 to 18.8 (Additional file 15).

Considering RNA-seq data in hypothalamus, from the 2241 predicted SOX targets, 1632 presented gene expression, including the top TFs with the only exception of SOX3. From the expressed targets included in the co-expression analysis, 360 (22%) presented significant results (Additional file 16, Additional file 17). Among those, SOX8/9/10 were co-expressed with other members of SOX family, namely SOX1, SOX2, SOX5, SOX6, SOX13, and SOX21.

**Fig. 5:**
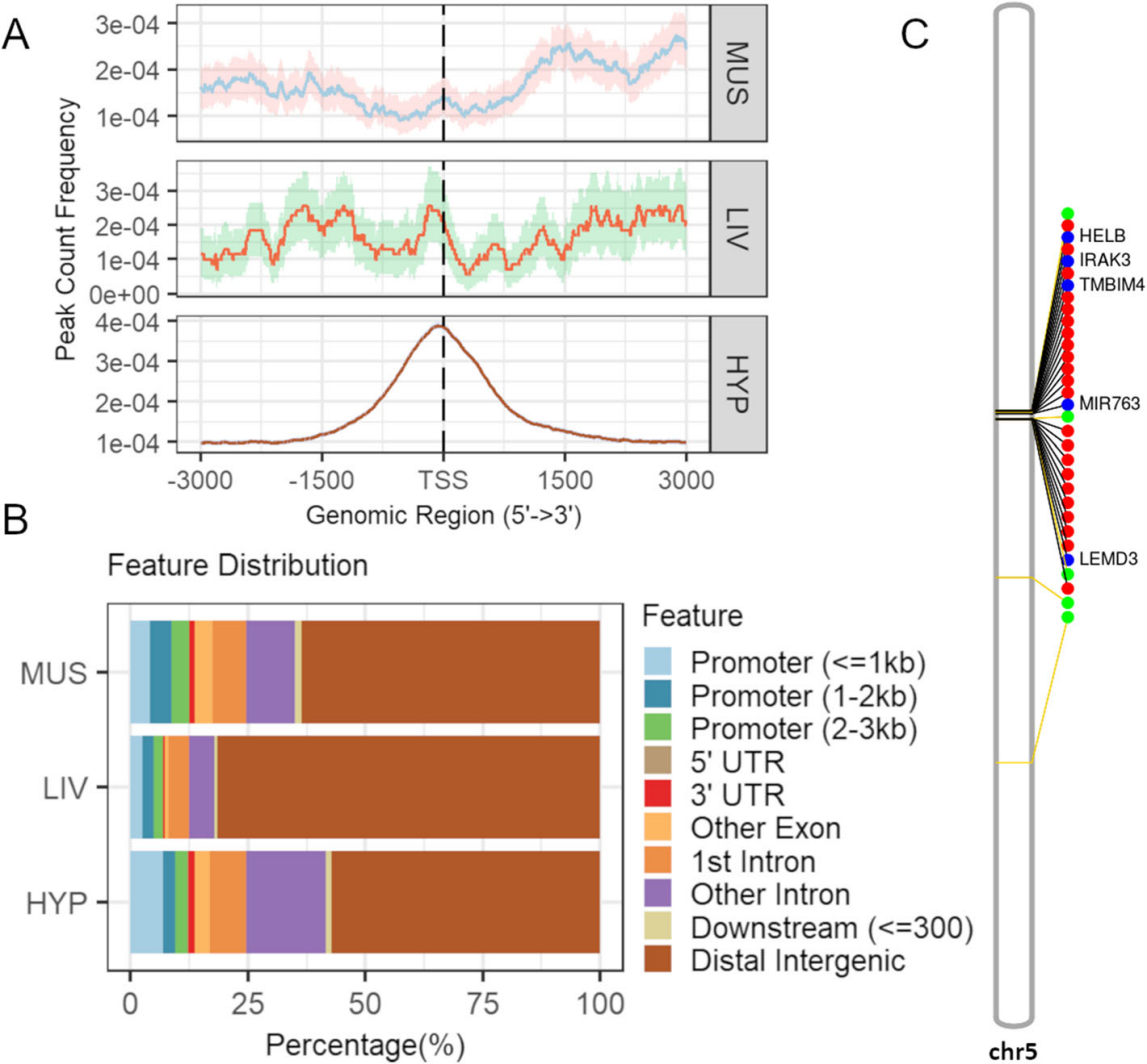
Potential indicine-specific peaks in muscle (MUS), liver (LIV) and hypothalamus (HYP). **A** Profile of peaks relative to transcription start sites (TSS) considering a ± 3 kb region—confidence intervals were estimated by bootstrap method (500 iterations) and is shown as the shading that follows each curve. **B** Percentage of overlap between peaks and genomic features. **C** Representation of bovine chromosome 5 and the location of indicine selective sweeps (green dots), peaks from all three tissues overlapping selective sweeps (red dots), and genes in close proximity (blue dots)

Although publicly available data on open chromatin regions of *B. taurus* were generated using different sex and methods [19], it provides us with a unique opportunity to identify possible indicine-specific regulatory regions. While we identified 78,528 peaks for hypothalamus, 40,104 peaks for muscle and 22,291 peaks for liver in *B. indicus*, the correspondent numbers in *B. taurus* data were 20,045, 77,378, and 58,853, respectively. Indicine-specific peaks falling on chr1-29 and X totalized 54,971 for hypothalamus, 4216 for muscle, and 2217 for liver (Additional file 18). Clearly, the higher number of peaks in hypothalamus identified in our study in comparison with the *B. taurus* data resulted in an inflation of indicine-specific peaks in that tissue, and therefore, the results need to be evaluated with caution. Apart from hypothalamus, indicine-specific peaks seem to be depleted from TSS regions (Fig. 5A) and concentrated on distal intergenic regions (Fig. 5B). Peaks in intergenic regions represented 57%, 63%, and 82% of indicine-specific peaks in hypothalamus, muscle, and liver, respectively, followed by 24%, 17%, and 10% in introns. Indicine-specific peaks in promoter regions only accounted for 12%, 12%, and 7%, respectively.

When we compared indicine-specific peaks with previously identified selective sweeps for indicus cattle [25], we found 3, 6, and 31 peaks with an overlap in liver, muscle, and hypothalamus, respectively (Additional file 19). Most of the overlapping peaks were located on chromosome 5 and, in all tissues, they felt in distal intergenic regions with the closest genes being MIR763 and LEMD3 (Fig. 5C). In addition, in hypothalamus the peaks on chromosome 5 also were distal to HELB and IRAK3, and in the promoter region of TMBIM4 and LEMD3. Peaks in other chromosomes only appeared in hypothalamus including chr4, chr6, chr8, chr12, chr18, and chr22. Among those, we can highlight the ones overlapping promoter regions of genes MEPE, FRY, and ZNF19.

## Discussion

The vast non-coding portion of the bovine genome, the one that regulates epigenetics changes, responds to environmental stimuli, and determines cell and tissue activity, is only starting to be characterized [12, 18, 19]. This non-coding genome is the key to understanding the expected differences between *B. indicus* and *B. taurus* and uncover the genetic basis of adaptability of indicine cattle to tropical and subtropical environments. Here, we used ATAC-seq data from indicine tissues that are key to adaptability and beef production (liver, muscle, and hypothalamus) not only to identify regulatory elements but to annotate their possible biding sites and targets in each tissue. We were able to identify HNF4 as a key regulator in liver, MEF2 in muscle and SOX in hypothalamus, and support those results based on gene co-expression and publicly available ChIP-seq and FAIRE-seq data. In addition, we compared *B. indicus* and *B. taurus* data and identified potential indicine-specific open chromatin regions, which mostly correspond to distal regulatory elements.

The peaks detected in the ATAC-seq data are expected to correspond to regulatory regions harboring functional combinations of regulatory elements, which dictate their spatiotemporal function [26]. Unravelling this regulation is the key to understanding the molecular mechanisms that control gene expression. For all our three tissues, most peaks fall within intergenic and promoter regions. This distribution of genomic features is in accordance with literature in human, mouse, and livestock species [18, 19], and correspond to two main types of regulatory elements involved in transcriptional regulation: promoters and enhancers. While promoters are located up to a few kilobases from a TSS, enhancers can be located long distances upstream or downstream of a target gene [27].

The proportion of peaks falling in promoters and intergenic regions changes when we look at tissue-specific peaks and constitutive regions. Although we can still see an enrichment around TSS for all subsets, the percentage of tissue-specific peaks within promoter regions drop dramatically (see Table 1), while for constitutive regions this percentage increases. This behavior suggests tissue-specific functions are more finely regulated by long-range regulatory elements, such as enhancers, silencers, and insulators. Indeed, it has been reported before that major tissue differences are due to changes in distal elements [4, 5, 28]. Conversely, constitutive regions represent housekeeping functions, and therefore, promoter regions are expected to be of open chromatin. In terms of transcriptional regulation, this distinction between constitutive/housekeeping vs. regulated/regulatory/developmental genes has proven to represent a real biological distinction rather than a human-defined classification [29].

Liver presented the smallest number of identified peaks and, consequently, of tissue-specific peaks. It also presented the highest proportion of tissue-specific peaks located in distal intergenic regions. This could be the reason why only one gene ontology term was enriched when considering the peaks nearest genes—positive chemotaxis. Chemotaxis refers to the directional migration of cells in response to a chemical stimulus, which is part of normal function and health in humans, such as immune system cells fighting injuries and infections, and tissue regeneration [30]. Liver is a frontline immune organ, responsible for detecting and clearing bacteria, viruses, and macromolecules from the blood, and is populated with several immune-centric cell types, including Kupfer cells, T cells, and NK cells [31]. In a healthy liver, metabolic functions and tissue remodeling are both requisites to maintain homeostasis [32]. In this context, hepatocyte nuclear factors (HNF) are transcription factors expressed predominately in liver. They work synergistically to raise transcriptional levels of distinct sets of hepatocyte-specific genes responsible for tissue development and metabolic homeostasis, among other functions [33, 34]. In our study, HNF4 was identified as the key regulator of liver-specific expression, but HNF1 also appear among the top TFs and both present co-expression in our RNA-seq data. Although HNF4 isoforms comprise two genes (HNF4A and HNF4G), a comparison of the DNA-binding domain of humans showed high homology between them, suggesting both genes may have similar functions in the transcriptional regulation of hepatic genes [35]. Importantly, in beef cattle, HNF4G has already been pointed as a key regulator when considering 29 traits (including meat quality, conformation, development, and metabolism) based on a marker-derived gene network [36].

Genes associated with muscle-specific peaks were enriched mostly with muscle cell development and MEF2 was pointed as a master regulator of muscle-specific open chromatin regions. The myocyte enhancer factor 2 family of transcription factors is comprised of variants A, B, C, and D with highly conserved protein domains across the MEF2 family [37]. In our study, MEF2A appear as the top enriched feature, and although it is expressed in various tissues/organs and plays crucial roles in multiple biological processes, it is widely present in muscle cells and involved in the development and differentiation of vertebrate skeletal, cardiac, and smooth muscle during myogenesis [38]. In cattle, MEF2A is a positive regulator in skeletal muscle myoblast proliferation and differentiation [38, 39] and mutations in its promoter region are highly associated with MEF2A mRNA expression in bulls, which in turn might be related to differences in muscle development and growth traits [40]. As in liver, the enrichment of open chromatin data in human cell lines specific to each tissue confirms the tissue specificity of the peaks/targets, validating our master regulators.

In cattle, hypothalamus is the least studied tissue of the three, probably due to difficulties to access and correctly identify it. It is also the most complex tissue involved in feedback loops related to the releasing of hormones, regulation of body temperature, maintenance of daily physiological cycles, control of appetite, management of sexual behavior, and regulation of emotional responses [41]. It has three main regions and our open chromatin regions are expected to represent all those regions collectively. It is no surprise then that hypothalamus presented the highest number of peaks and tissue-specific peaks. Indeed, gene expression in hypothalamus when compared to liver and muscle has shown to be higher in indicine cattle [42, 43]. Concordantly with its functions, genes associated with hypothalamus-specific peaks were enriched for terms related to cell communication and nervous system development. Admittedly, our definition of tissue-specific peaks is limited as we are only comparing three tissues and hypothalamus-specific peaks could include open chromatin regions common to other nervous system tissues. Nevertheless, our data points to the SRY-related HMG box (SOX) genes as candidate master regulators of hypothalamus expression.

The HMG box is a DNA-binding domain highly conserved throughout eukaryotic species and the SOX family is divided into subgroups according to homology within this domain and other structural motifs—SOX8, SOX9, and SOX10 are part of Sox group [44]. Apart from SoxE group, SOX3 (SoxB1 group) and SOX6 (SoxD group) also appear in the top 5 enriched transcription factors. Several SOX genes presented expression in hypothalamus and the co-expression of SOX8/9/10 with SOX1 and SOX2 (SoxB1 group); SOX5, SOX6, and SOX13 (SoxD group) and SOX21 (SoxB2 group) show a coordinated activity of this family. SOX genes are related to several different aspects of development and while many are involved in sex determination, some are also important in processes such as neuronal development. Within the tuberal hypothalamus, neural progenitors are known to give rise to supportive and active signaling central nervous system glial cells. This process starts with progenitor cells expressing SOX9 which further mature and start to express SOX10 [45]. As a parallel, in pituitary, Sox2+/Sox9+ cells were demonstrated to be able to generate all hormone-producing cell subtypes [46]. Altogether, SOX genes and in particular group SoxE are indicated as a potential master regulator of hypothalamic gene expression.

Finally, the *B. taurus vs B. indicus* contrast has long been the subject of studies aimed at characterizing signatures of selection [47]. Mutations affecting complex traits may be subject to natural or artificial selection, which leaves a selection signature in the genome [48, 49]. However, while the cattle genome has been shaped significantly by human domestication [50], earlier work in cattle suggested few discernible signatures of selection in the cattle genome sequence after strong artificial selection for complex traits [51]. Nevertheless, the epigenome can be responsible for carrying some of the answers for adaptation-related traits that differ across subspecies. In our study, indicine-specific peaks were conspicuously lacking near TSS, which was less apparent for hypothalamus due to the large difference in identified peaks between datasets. For all tissues, most of the peaks were in intergenic regions, corresponding to enhancers, which is in accordance with what was observed when comparing different species [18, 19]. For instance, a comparison of cattle with pig and mouse showed as little as 17% and 6% overlap in intergenic open chromatin, respectively [19]. Enhancers are rapidly evolving regulatory sequences, being a species-specific feature likely to impact differing phenotypes [52–55]. In the comparison between the subspecies indicus and taurus, this enhancer-based regulation seems to be even more explicit. Therefore, we trust the indicine-specific open chromatin regions reported here represent a rich source for mining mutations likely to affect cattle adaptation to different climatic zones.

Because most of the indicine-specific peaks were located in intergenic locations, using the nearest gene to draw possible biological functions could be misleading, as enhancers can regulate genes in long distances and even different chromosomes and not necessarily the nearest gene [56]. However, testing the overlap between selective sweeps in *Bos indicus* and indicine-specific peaks could demonstrate overlapping mechanisms in the control of adaptive differences. Indeed, there was overlap, which mostly happened on chromosome 5. Importantly, hypothalamus peaks on chromosome 5 were distal to HELB, a gene already shown to be related to differences between both cattle subspecies [25].

## Conclusions

A comparative analysis of the chromatin accessibility in muscle, liver, and hypothalamus of *Bos indicus* cattle revealed new insights into the tissue-specific regulation of gene expression with an unprecedented level of accuracy. The integration of transcriptomic data allowed us to indicate, more accurately, possible targets of master regulators in each tissue, including a prediction of their biding sites. Furthermore, the indication of indicine-specific open chromatin regions provides a promising avenue to exploit molecular mechanisms to artificial selection for traits of relevance to the adaptation to tropical and subtropical climates.

## Methods

### Collection of tissue and generation of ATAC-seq libraries

Liver, hypothalamus, and muscle samples were collected from three unrelated, post-pubertal Brahman heifers of similar age and weight as previously described [21–23]. Heifers used in this study were managed, handled, and euthanized as per approval of the Animal Ethics Committee of the University of Queensland, Production and Companion Animal group (certificate number QAAFI/279/12). After slaughter, tissue samples were collected as fast as possible and stored at − 80 °C.

ATAC-seq libraries were prepared from frozen tissues using the Omni-ATAC method [57] with the following modifications. Frozen tissue (20 mg) was ground in liquid nitrogen using a mortar and pestle. The pulverized tissue was transferred to a pre-chilled 2 ml Dounce homogenizer containing 1 ml cold 1× homogenization buffer and homogenized with the pestle until a uniform suspension was seen (10–20 strokes). The homogenate was filtered with a 40-μM nylon cell strainer (BD Falcon) before layering onto the iodixanol solution as described previously [57]. The ratio of nuclei to enzyme concentration was optimized for each sample by performing transposition reactions containing 50,000, 100,000, and 200,000 nuclei with 2.5 μl of tagment enzyme in 50 μl of transposition mix [57]. The transposed DNA was amplified with custom primers as previously described [58]. Amplified libraries were purified using Agencourt AMPure XP beads (Beckman Coulter) and quality controlled using a Bioanalyser High Sensitivity DNA Analysis kit (Agilent). ATAC-seq libraries were sequenced at IMB sequencing facility (University of Queensland) on an Illumina NextSeq 150 cycle (2X 75 bp). Three biological replicates were performed per tissue. This dataset is publicly available at NCBI Gene Expression Omnibus (GEO) under the accession number GSE182909 [59]. All assays were performed according to FAANG guidelines and recommendations, available at http://www.faang.org. The detailed protocol used in ATAC-seq is available at https://data.faang.org/protocol/samples/ROSLIN_SOP_ATAC_Seq_DNAIsolationandTagmentation_Frozen_Muscle_Tissue_20200720.pdf.

### Mapping and ATAC-seq peak calling

ATAC-seq data processing and alignment was completed using the Harvard pipeline (https://informatics.fas.harvard.edu/atac-seq-guidelines.html). First, reads quality was accessed using the tool FastQC (https://www.bioinformatics.babraham.ac.uk/projects/fastqc/). After checking no adapter contamination existed, sample reads were aligned to the cattle reference genome (ARS-UCD1.2) using HISAT2 v2.1.0 with -k 10 to allow for multiple alignments [60]. Summary mapping statistics were performed using Samtools flagstat (v1.9) [61].

Peak calling was performed using the tool Genrich v0.6.1 (available at https://github.com/jsh58/Genrich) including all biological replicates per tissue and parameters -j (ATAC-seq mode) -r (remove PCR duplicates) -e MT (to exclude mitochondrial chromosome) -p 0.01 (*p* value). Genrich analyzes reads that map to multiple locations in the genome by adding a fractional count to each location, allowing for peak detection in regions that are otherwise inaccessible to the assay. Moreover, it calls peaks for multiple biological replicates collectively by first analyzing the replicates separately and then combining the multiple replicates’ *p* values at each genomic position using Fisher’s method to identify significant consensus peaks per tissue.

### Annotation of peaks and tissue specificity

After peak calling, peak location was accessed by the R package ChIPseeker [62] using as reference the Bioconductor *Bos taurus* annotation libraries TxDb.Btaurus.UCSC.bosTau9.refGene [63] and org. Bt.eg.db [64] (A detailed tutorial can be found at https://www.bioconductor.org/packages/release/bioc/vignettes/ChIPseeker/inst/doc/ChIPseeker.html). Briefly, the ChIPseeker covplot function was used to calculate and visualize the coverage of peak regions over chromosomes. Then, the profile of peaks binding to TSS regions was visualized by first defining the TSS regions as ± 3 kb of TSS sites, and then aligning the peaks that were mapped to these regions using the ChIPseeker getTagMatrix function and TxDb.Btaurus.UCSC.bosTau9.refGene database as reference. Heatmaps of peak profile around TSS were produced using ChIPseeker tagHeatmap function and peak distribution profiles were produced using ChIPseeker plotAvProf which generate confidence intervals estimated by bootstrap method. Peak annotation to functional categories was performed by ChIPseeker annotatePeak function, which reports the genomic region of the peak (following the priority order: Promoter, 5′ UTR, 3′ UTR, Exon, Intron, Downstream, and Intergenic), the position and strand of the nearest gene, and the distance to TSS of the nearest gene using the org.Bt.eg.db database as a reference. ChIPseeker plotDistToTSS was used to calculate the percentage of peaks upstream and downstream from the TSS of the nearest genes and visualize the distribution.

To compare the three tissues and identify tissue-specific peaks, we used bedtools intersect -v (v. 2.29.2) [65] for each pairwise contrast. By looking at the results of the multiple contrasts, we defined peaks exclusive to one tissue as tissue-specific. Then, bedtools multiIntersectBed [65] was used to identify overlapping regions across the three tissues. Although a perfect overlap of peaks from different tissues is unlikely, regions of overlap can be of biological significance and will be referred to as constitutive regions. Considering the annotated nearest gene of peaks/regions, we performed an enrichment analysis of Gene Ontology (GO) terms using the function compareCluster from R package clusterProfiler [66] using the following parameters: OrgDb = org.Bt.eg.db, fun = “enrichGO,” ont = “ALL,” pAdjustMethod = “BH,” pvalueCutoff = 0.05.

### Motif enrichment analysis

To identify enriched TFBSs within tissue-specific peaks, peaks coordinates in each tissue were converted first to human hg38 coordinates using the liftOver tool [67] (minMatch = 0.1), and then to human hg19 coordinates (minMatch = 0.95). The hg19 orthologous regions were used as input to the motif discovery tool i-*cis*Target v6.0 [68] which contains 24,453 PWMs gathered from multiple databases (see:http://iregulon.aertslab.org/collections.html#motifcolldesc). The i-*cis*Target tool contains motif information across seven species, including cow, and those motifs were previously scored for the enrichment of homotypic clusters of PWM using a Hidden Markov Model from the tool Cluster-Buster [69]. The seven species whole-genome rankings per motif were combined in a final rank using order statistics to prioritize highly ranked regions per motif across species [70]. The user-defined regions are interrogated for motifs significantly enriched using the cross-species final rank. In addition, it provides the identification of target regions for a PWM by determining the optimal threshold through a receiver operating characteristic curve which compares the enrichment of a PWM versus the enrichment across all 24,453 PWMs average. The Normalized Enrichment Score (NES) is the AUC score normalized by subtracting the mean of all AUC overall motifs and dividing it by the standard deviation. Finally, motifs referring to similar TFs are colour coded and master regulators for each tissue can be determined. For each predicted TF, the top 10 PWMs are gathered to determine which Human regions are predicted as targets.

In addition, i-*cis*Target has a collection of 1331 TF ChIP-seq information and 2450 Histone modification tracks in human tissues and cell lines extracted from ENCODE and RoadMap Epigenomics databases [13]. The user-defined regions are interrogated for each track collection and tracks significantly enriched are identified by determining the optimal threshold through a receiver operating characteristic curve. This analysis compares the enrichment of a track of the collection versus the average enrichment across the whole track collection and generates an NES.

### Annotating the location of TF binding sites in the cattle genome

Human (hg19) regions predicted as targets for the top TF in liver, muscle, and hypothalamus were converted back to ARS-UCD1.2 coordinates using the liftOver tool as described before [67]. Next, the overlap between these target regions and ATAC-seq peaks in each tissue was calculated using bedtools intersect (-wa -F 0.40) (v2.29.2) [65]. To score and locate the potential TFBSs per TF of interest, we downloaded the PWMs for the top 10 PWMs associated with a TF from the motif discovery analysis [68]. Peaks overlapping target regions for a TF were converted to fasta and re-scanned for the homotypic cluster of PWMs using the Hidden Markov Model from the tool Cluster-Buster (-m 0 -c 0) [69]. The homotypic cluster score, motif score, and predicted binding location were calculated to annotate the active biding sites of the key transcription factor in each tissue.

### Gene regulatory network

To validate the relationships between the master regulator of each tissue (top TF) and its predicted targets at transcriptional level, we used the previously described RNA-seq data of liver and hypothalamus to investigate gene co-expression [21–23]. In addition to the three post-pubertal Brahman heifers used for ATAC-seq libraries, RNA-seq data included three prepubertal Brahman heifers coming from the same original data so the number of samples would enable a co-expression study. This dataset is publicly available at EMBL-EBI BioSamples repository (www.ebi.ac.uk/biosamples) under the submission identifiers GSB-113 and GSB-8708 [71]. RNA-seq reads were aligned to the same cattle reference genome, and read counts were estimated using -tools [72]. The EdgeR R package [73] was used to normalize the counts by TMM (trimmed mean of M values) for each tissue, and only genes presenting at least 1 count per million reads mapped (CPM) in at least half of the samples were considered for further analysis. For each tissue, gene expression in log2CPM of the master regulator and its predicted targets were used to identify significant connections using the Partial Correlation and Information Theory (PCIT) algorithm [74]. PCIT determinates significant correlations between two genes after accounting for all the other genes under scrutiny.

Gene Regulatory Network visualization was performed using Cytoscape 3.6.0 [75]. Genes having ATAC-seq peaks associated with binding for a TF were drawn in the network as potential target genes and connections validated by co-expression were highlighted.

### Identification of Bos indicus-specific open chromatin regions

To compare open chromatin regions between *B. taurus* and *B. indicus* and identify indicine-specific regions, we used data from [19]. Briefly, the authors generated ATAC-seq data from liver, muscle, and hypothalamus of two Hereford males and the identified peaks per sample were available in their Additional File 2. Although there are differences in sex and methods regarding library preparation, sequencing, and peak calling, both studies used the same reference genome which provides us with a unique opportunity to compare results. As described by the authors, peaks called for individual biological replicates were compared with bedtools intersect and then merge collapsed with bedtools merge (v2.29.2) [65]. To identify indicine-specific peaks, we compared indicus and taurus peaks in each tissue using bedtools intersect -v. Peaks were then annotated to functional categories using ChIPseeker annotatePeak [62] as described before. Finally, we compared indicine-specific peaks with regions of selective sweeps for indicine cattle, which were previously identified by our group [25] and are publicly available as Table S5 (selective sweeps in Asian Indicine cattle based on Fst and nucleotide diversity across *Bos indicus* and *Bos taurus* cattle, Padj < 0.05). For this comparison, we used bedtools intersect -wa to identify overlaps.

## Supplementary Information

**Additional file 1.** Summary mapping statistics per sample.

**Additional file 2.** Identified peaks and annotation for hypothalamus. The first 10 columns correspond to ENCODE narrowPeak format, the following columns are the annotation output of ChIPseeker [62], and the last column indicates which of the peaks are tissue-specific.

**Additional file 3.** Identified peaks and annotation for liver. The first 10 columns correspond to ENCODE narrowPeak format, the following columns are the annotation output of ChIPseeker [62], and the last column indicates which of the peaks are tissue-specific.

**Additional file 4.** Identified peaks and annotation for muscle. The first 10 columns correspond to ENCODE narrowPeak format, the following columns are the annotation output of ChIPseeker [62], and the last column indicates which of the peaks are tissue-specific.

**Additional file 5.** Distribution of peaks by chromosome for muscle (A), liver (B) and hypothalamus (C).

**Additional file 6.** Complete distribution of genomic features overlapping peaks identified in muscle (A), liver (B) and hypothalamus (C).

**Additional file 7.** Identified peaks and annotation for constitutive regions. The first 10 columns correspond to ENCODE narrowPeak format and the following columns are the annotation output of ChIPseeker [62].

**Additional file 8.** Functional enrichment of gene ontology terms for tissue-specific peaks.

**Additional file 9.** (AdditionalFile9.pdf) - Enriched regulatory features in liver-specific peaks according to i-cisTarget online tool [68].

**Additional file 10.** Annotation of possible binding sites of NHF4 in cattle liver according to Cluster-Buster [69].

**Additional file 11.** Significant partial correlation between HNF4 and its possible targets in liver using RNAseq data.

**Additional file 12.** Enriched regulatory features in muscle-specific peaks according to i-cisTarget online tool [68].

**Additional file 13.** Annotation of possible binding sites of MEF2 in cattle muscle according to Cluster-Buster [69].

**Additional file 14.** Enriched regulatory features in hypothalamus-specific peaks according to i-cisTarget online tool [68].

**Additional file 15.** Annotation of possible binding sites of SOX8/9/10 in cattle hypothalamus according to Cluster-Buster [69].

**Additional file 16.** Hypothalamus-specific master regulator SOX and its predicted targets. Dotted edges represent predicted targets, continuous edges and red borders represent targets with significant co-expression using RNA-seq data.

**Additional file 17.** Significant partial correlation between SOX8/9/10 and its possible targets in hypothalamus using RNAseq data.

**Additional file 18.** Indicine-specific peaks in liver, muscle, and hypothalamus.

**Additional file 19.** Indicine-specific peaks in liver, muscle, and hypothalamus which overlap selective sweeps in indicine cattle.

**Additional file 20.** Peer review history.

## Data Availability

The ATAC-seq dataset supporting the conclusions of this article is publicly available at NCBI Gene Expression Omnibus (https://www.ncbi.nlm.nih.gov/geo/) under the accession number GSE182909 [59]. The RNA-seq dataset used to build the co-expression networks is publicly available at EMBL-EBI BioSamples repository (www.ebi.ac.uk/biosamples) under the submission identifiers GSB-113 and GSB-8708 [71].
