# Supplementary figures and images for: Chromatin accessibility and regulatory vocabulary across indicine cattle tissues
Source: Genome Biol. 2021 Sep 21;22:273. doi: 10.1186/s13059-021-02489-7 (PMC8454054; doi:10.1186/s13059-021-02489-7)

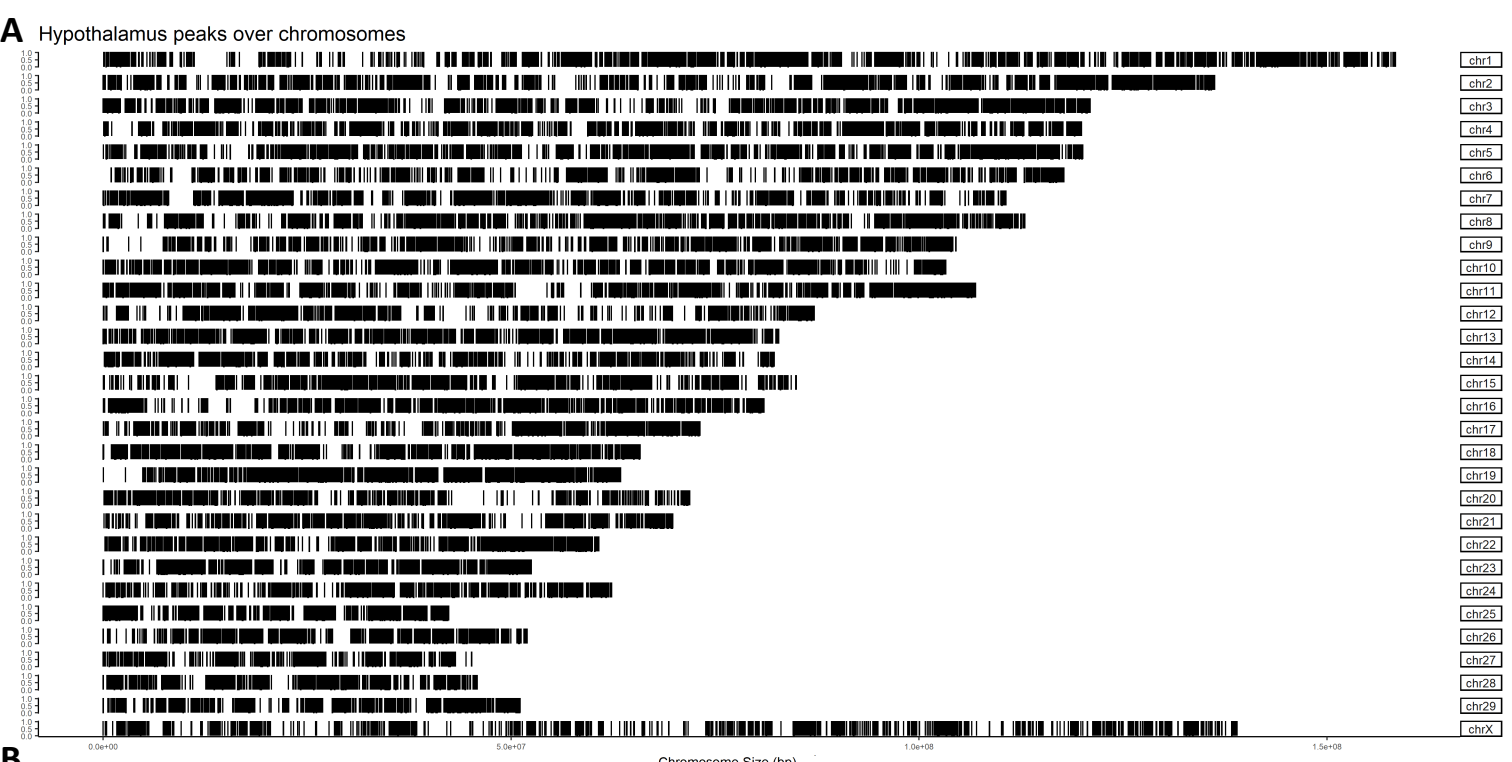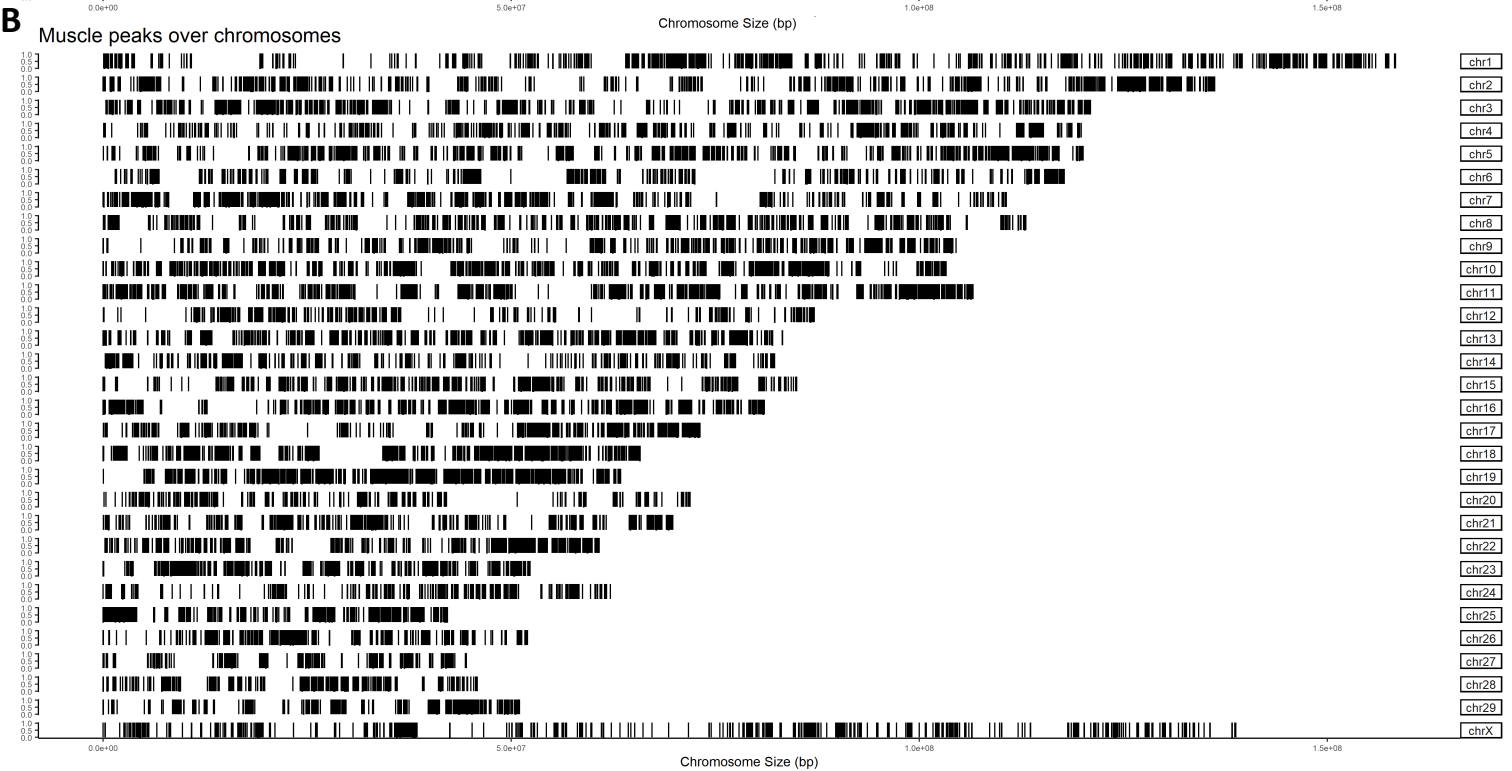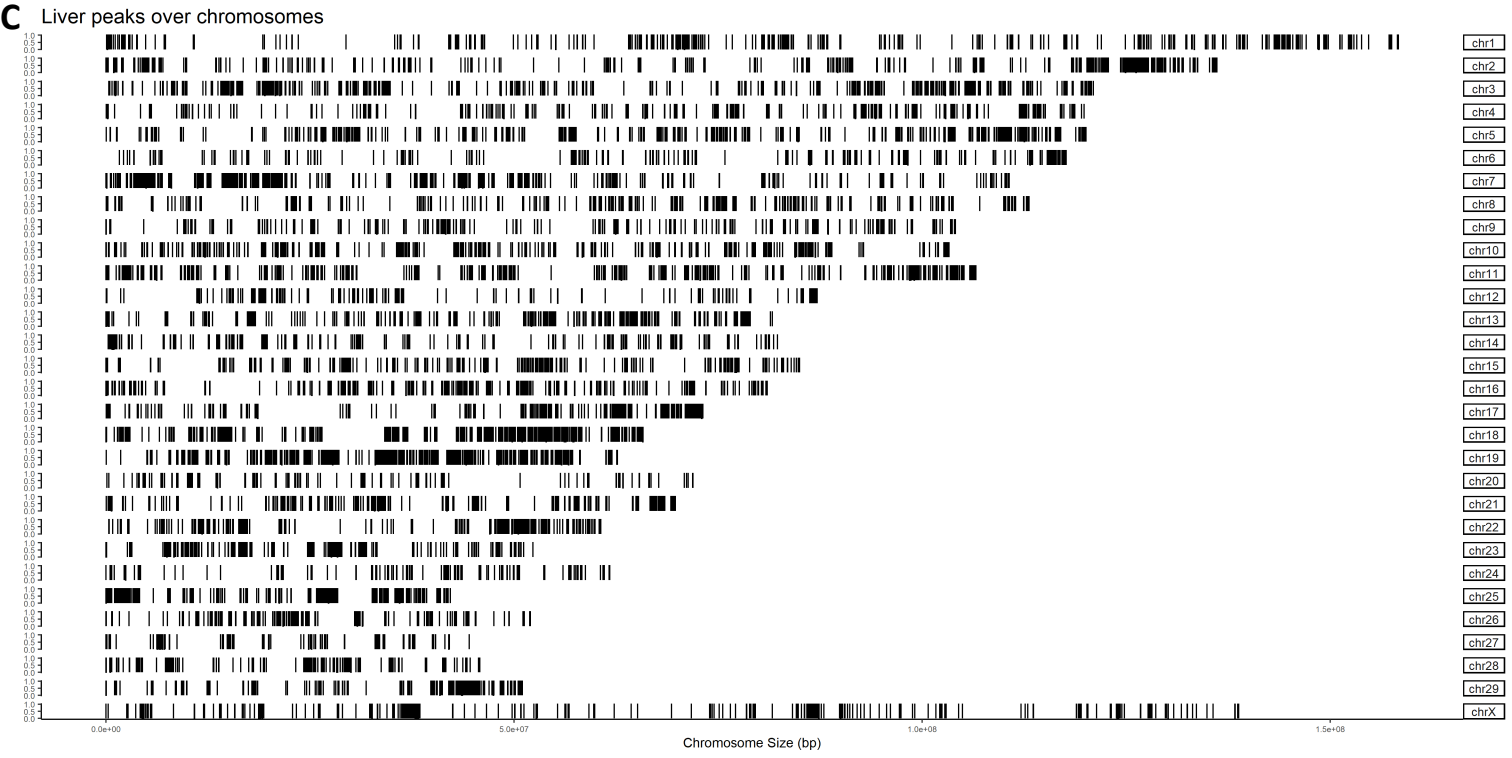

Supplement: Supplementary file 5 — Additional file 5. Distribution of peaks by chromosome for muscle (A), liver (B) and hypothalamus (C). [file 13059_2021_2489_MOESM5_ESM.pdf]

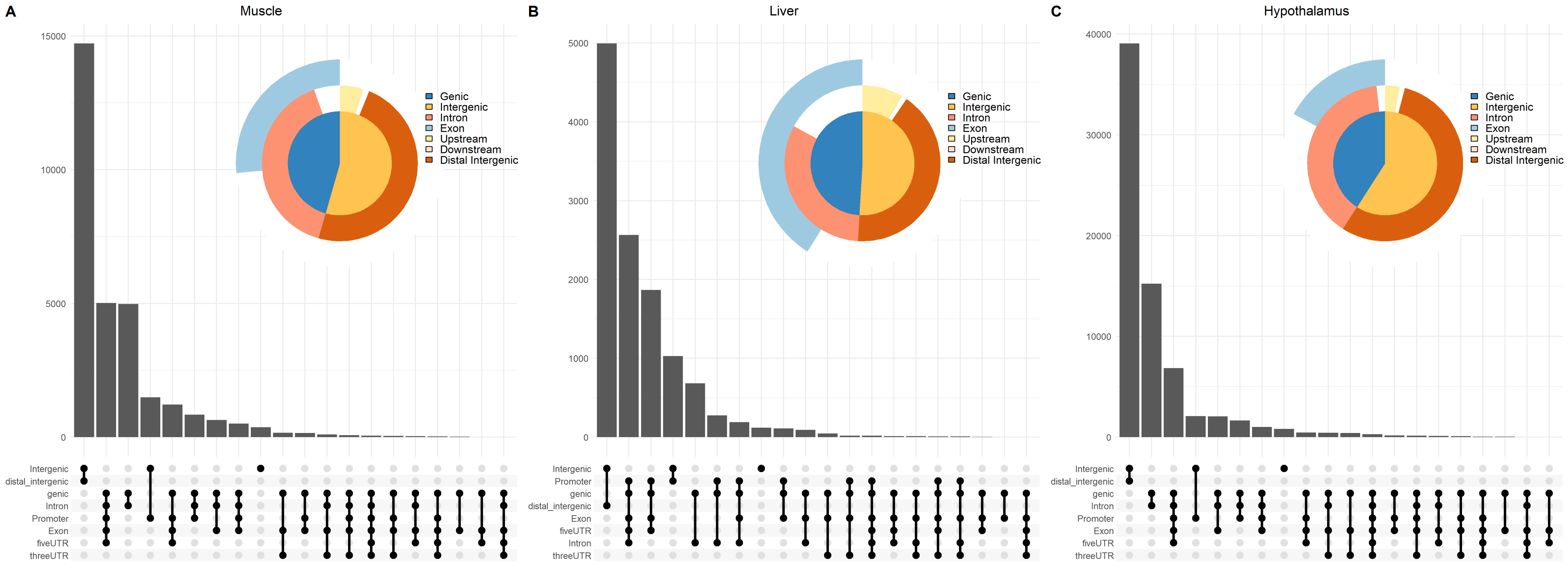

Supplement: Supplementary file 6 — Additional file 6. Complete distribution of genomic features overlapping peaks identified in muscle (A), liver (B) and hypothalamus (C). [file 13059_2021_2489_MOESM6_ESM.pdf]

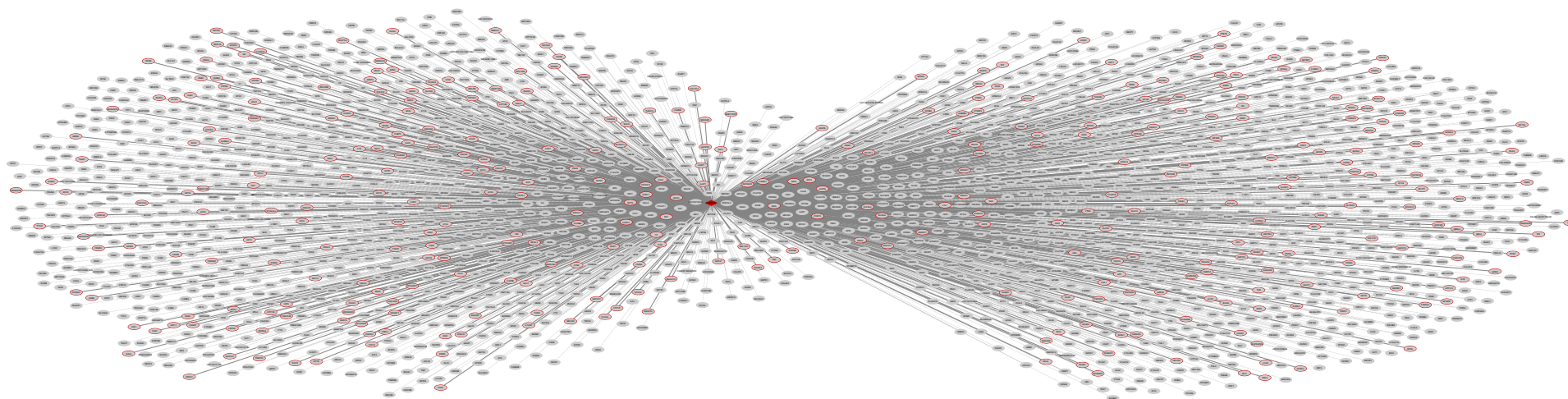

Supplement: Supplementary file 16 — Additional file 16. Hypothalamus-specific master regulator SOX and its predicted targets. Dotted edges represent predicted targets, continuous edges and red borders represent targets with significant co-expression using RNA-seq data. [file 13059_2021_2489_MOESM16_ESM.pdf]
