# Supplementary material for: Chromatin accessibility and regulatory vocabulary across indicine cattle tissues
Source: Genome Biol. 2021 Sep 21;22:273. doi: 10.1186/s13059-021-02489-7 (PMC8454054; doi:10.1186/s13059-021-02489-7)
Supplement: Supplementary file 9 — Additional file 9. (AdditionalFile9.pdf) - Enriched regulatory features in liver-specific peaks according to i-cisTarget online tool [68]. [file 13059_2021_2489_MOESM9_ESM.pdf]

i-cisTarget

An integrative genomics method for the prediction of regulatory features and cis-regulatory modules.

Parameters and statistics for Liver

Number of features	29890
Number of enriched features (NES > 3.0)	440
Total number of ranked regions	1223024
Type of input query	bed
Number of **i-cisTarget regions in input set**	419 (**Results of the region mapping**)
Minimum fraction of overlap	0.4
Normalized enrichment score (NES) threshold	3.0
AUC threshold (fraction / # of ranked regions)	0.005 (6115)
Recovery curve threshold (# of regions)	20000

AUC distribution

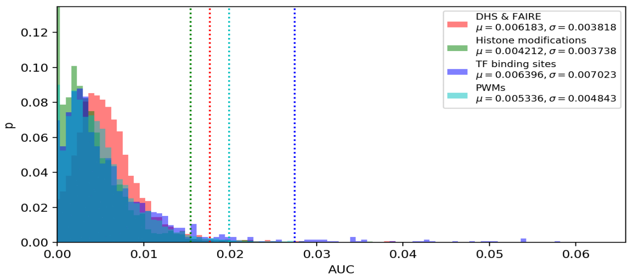

Recovery of best feature

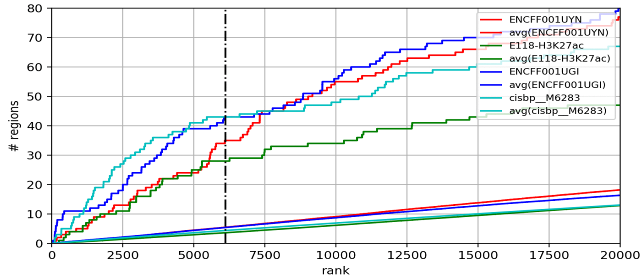

Results for Liver

Select features in the table below, select an operation and proceed.

1. ○ Use candidate target regions as **filter** and use as input for i-cisTarget again.
2. ○ **Scan** candidate target regions of selected features for multiple homotypic CRMs.
3. ○ **Create SIF file** for the selected features.

This report is also available as an **archive**.

For some databases, randomly generated features were enriched. Also, non-random features in these same databases that have a lower enrichment than the first randomly generated enriched feature were removed from the report. The involved databases and their adjusted NES thresholds are: DHS & FAIRE (3.46670).

#	Feature	NES	Logo	Recovery Curve	Candidate targets	All regions in top 20000	Database
1	☐ cisbp__M6283<br>Description: HNF4A[gene ID: "ENSG00000101076" species: "Homo sapiens" TF status: "direct" TF family: "Nuclear receptor" DBDs: "zf-C4"]; Hnf4g[gene ID: "ENSMUSG00000017688" species: "Mus musculus" TF status: "inferred" TF family: "Nuclear receptor" DBDs: "zf-C4"]<br>Possible TFs: HNF4A	*12.47575*	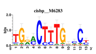	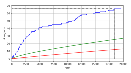	link	link	PWMs
2	☐ hocomoco__NR1H3_MOUSE.H11MO.0.A<br>Description: NR1H3_MOUSE<br>Possible TFs: NR1H3	*11.42016*	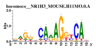	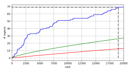	link	link	PWMs
3	☐ hocomoco__HNF4G_HUMAN.H11MO.0.B<br>Description: HNF4G_HUMAN<br>Possible TFs: HNF4G	*10.59755*	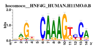	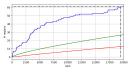	link	link	PWMs
4	☐ cisbp__M6281<br>Description: HNF1A[gene ID: "ENSG00000135100" species: "Homo sapiens" TF status: "direct" TF family: "Homeodomain" DBDs: "Homeobox"]<br>Possible TFs: HNF1A	*10.58329*	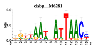	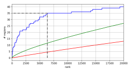	link	link	PWMs

#	Feature	NES	Logo	Recovery Curve	Candidate targets	All regions in top 20000	Database
5	☐ hocomoco__HNF1A_MOUSE.H11MO.0.A<br>Description: HNF1A_MOUSE<br>Possible TFs: HNF1A	*10.52663*	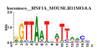	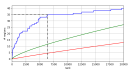	**link**	**link**	PWMs
6	☐ transfac_pro__M01031<br>Description: V$HNF4_Q6_01: HNF-4<br>Possible TFs: HNF4A	*10.27817*	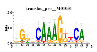	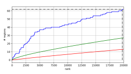	**link**	**link**	PWMs
7	☐ dbcorrdb__HNF4A__ENCSR000EEU_1__m1<br>Description: HNF4A (ENCSR000EEU-1, motif 1)<br>Possible TFs: HNF4A	*10.25472*	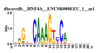	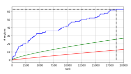	**link**	**link**	PWMs
8	☐ hocomoco__HNF1A_HUMAN.H11MO.0.C<br>Description: HNF1A_HUMAN<br>Possible TFs: HNF1A	*10.20338*	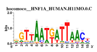	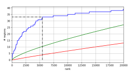	**link**	**link**	PWMs
9	☐ transfac_pro__M02220<br>Description: V$HNF4A_03: HNF4A<br>Possible TFs: HNF4A	*10.14737*	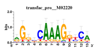	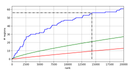	**link**	**link**	PWMs
10	☐ dbcorrdb__HNF4A__ENCSR000EEU_1__m2<br>Description: HNF4A (ENCSR000EEU-1, motif 2)<br>Possible TFs: HNF4A	*9.96910*	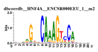	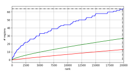	**link**	**link**	PWMs
11	☐ hocomoco__HNF4A_MOUSE.H11MO.0.A<br>Description: HNF4A_MOUSE<br>Possible TFs: HNF4A	*9.82105*	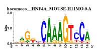	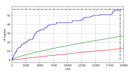	**link**	**link**	PWMs
12	☐ hocomoco__HNF1B_MOUSE.H11MO.0.A<br>Description: HNF1B_MOUSE<br>Possible TFs: HNF1B	*9.53970*	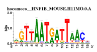	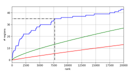	**link**	**link**	PWMs
13	☐ factorbook__HNF4<br>Description: HNF4<br>Possible TFs: HNF4G, HNF4A	*9.53229*	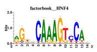	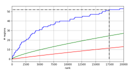	**link**	**link**	PWMs
14	☐ dbcorrdb__HNF4G__ENCSR000BNJ_1__m1<br>Description: HNF4G (ENCSR000BNJ-1, motif 1)<br>Possible TFs: HNF4G	*9.37449*	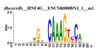	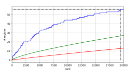	**link**	**link**	PWMs
15	☐ hocomoco__HNF1B_HUMAN.H11MO.0.A<br>Description: HNF1B_HUMAN<br>Possible TFs: HNF1B	*9.34523*	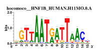	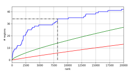	**link**	**link**	PWMs
16	☐ cisbp__M1936<br>Description: HNF4A[gene ID: "ENSG00000101076" species: "Homo sapiens" TF status: "direct" TF family: "Nuclear receptor" DBDs: "zf-C4"]; Hnf4g[gene ID: "ENSMUSG00000017688" species: "Mus musculus" TF status: "inferred" TF family: "Nuclear receptor" DBDs: "zf-C4"]<br>Possible TFs: HNF4A	*9.26335*	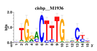	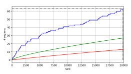	**link**	**link**	PWMs
17	☐ transfac_pro__M07321<br>Description: V$HNF4A_Q3: HNF-4A<br>Possible TFs: HNF4A	*9.22660*	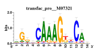	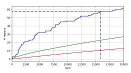	**link**	**link**	PWMs
18	☐ transfac_pro__M07215<br>Description: V$HNF4A_11: HNF4A<br>Possible TFs: HNF4A	*9.12506*	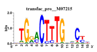	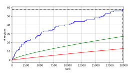	**link**	**link**	PWMs
19	☐ dbcorrdb__HNF4G__ENCSR000BNJ_1__m2<br>Description: HNF4G (ENCSR000BNJ-1, motif 2)<br>Possible TFs: HNF4G	*9.07219*	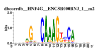	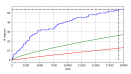	**link**	**link**	PWMs
20	☐ transfac_pro__M04719<br>Description: V$HNF4G_01: HNF-4gamma<br>Possible TFs: HNF4G	*9.03874*	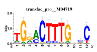	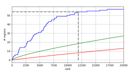	**link**	**link**	PWMs
21	☐ cisbp__M6282<br>Description: HNF1B[gene ID: "ENSG00000108753" species: "Homo sapiens" TF status: "direct" TF family: "Homeodomain" DBDs: "Homeobox"]	*8.99627*	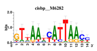	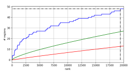	**link**	**link**	PWMs
22	☐ transfac_public__M00132<br>Description: V$HNF1_01: HNF-1alpha<br>Possible TFs: HNF1A	*8.94276*	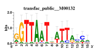	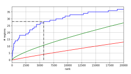	**link**	**link**	PWMs

#	Feature	NES	Logo	Recovery Curve	Candidate targets	All regions in top 20000	Database
23	☐ hocomoco__HNF4A_HUMAN.H11MO.0.A<br>Description: HNF4A_HUMAN<br>Possible TFs: HNF4A	*8.82912*	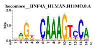	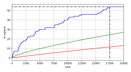	link	link	PWMs
24	☐ dbcorrdb__HNF4A__ENCSR000BLF_1__m1<br>Description: HNF4A (ENCSR000BLF-1, motif 1)<br>Possible TFs: HNF4A	*8.75111*	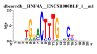	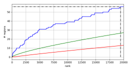	link	link	PWMs
25	☐ cisbp__M4698<br>Description: HNF4A[gene ID: "ENSG00000101076" species: "Homo sapiens" TF status: "direct" TF family: "Nuclear receptor" DBDs: "zf-C4"]; Hnf4g[gene ID: "ENSMUSG00000017688" species: "Mus musculus" TF status: "inferred" TF family: "Nuclear receptor" DBDs: "zf-C4"]<br>Possible TFs: HNF4A	*8.73894*	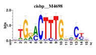	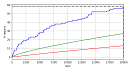	link	link	PWMs
26	☐ dbcorrdb__HNF4A__ENCSR000BLF_1__m2<br>Description: HNF4A (ENCSR000BLF-1, motif 2)<br>Possible TFs: HNF4A	*8.70114*	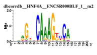	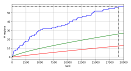	link	link	PWMs
27	☐ transfac_pro__M02162<br>Description: V$HNF1A_01: HNF1A<br>Possible TFs: HNF1A	*8.64118*	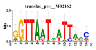	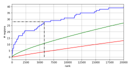	link	link	PWMs
28	☐ swissregulon__hs__HNF1A.p2<br>Description: hs__HNF1A.p2<br>Possible TFs: HNF1A	*8.60516*	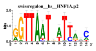	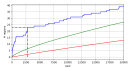	link	link	PWMs
29	☐ ENCFF001UYN<br>Description: FAIRE-seq on HepG2	*8.55241*		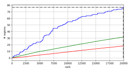	link	link	DHS & FAIRE
30	☐ E118-H3K27ac<br>Description: H3K27ac in HepG2 Hepatocellular Carcinoma Cell Line (E118,)	*8.54822*		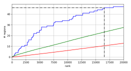	link	link	Histone modifications
31	☐ transfac_pro__M03828<br>Description: V$HNF4_Q6_04: HNF-4<br>Possible TFs: HNF4A	*8.53158*	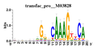	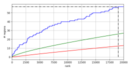	link	link	PWMs
32	☐ dbcorrdb__HDAC2__ENCSR000BMC_1__m2<br>Description: HDAC2 (ENCSR000BMC-1, motif 2)<br>Possible TFs: HDAC2	*8.43148*	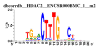	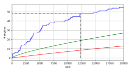	link	link	PWMs
33	☐ E084-DNase.hotspot.all.peaks-narrowpeak<br>Description: DNase in Fetal Intestine Large (E084, narrowpeak, hotspot)	*8.41412*		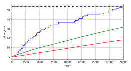	link	link	DHS & FAIRE
34	☐ cisbp__M1878<br>Description: HNF1A[gene ID: "ENSG00000135100" species: "Homo sapiens" TF status: "direct" TF family: "Homeodomain" DBDs: "Homeobox"]<br>Possible TFs: HNF1A	*8.16940*	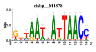	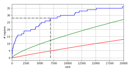	link	link	PWMs
35	☐ cisbp__M4576<br>Description: AL662824.5[gene ID: "ENSG00000227322" species: "Homo sapiens" TF status: "inferred" TF family: "Nuclear receptor" DBDs: "zf-C4"]; AL844527.7[gene ID: "ENSG00000206289" species: "Homo sapiens" TF status: "inferred" TF family: "Nuclear receptor" DBDs: "zf-C4"]; CR354565.3[gene ID: "ENSG00000231321" species: "Homo sapiens" TF status: "inferred" TF family: "Nuclear receptor" DBDs: "zf-C4"]; CR759733.4[gene ID: "ENSG00000228333" species: "Homo sapiens" TF status: "inferred" TF family: "Nuclear receptor" DBDs: "zf-C4"]; CR936877.3[gene ID: "ENSG00000235712" species: "Homo sapiens" TF status: "inferred" TF family: "Nuclear receptor" DBDs: "zf-C4"]; RXRA[gene ID: "ENSG00000186350" species: "Homo sapiens" TF status: "direct" TF family: "Nuclear receptor" DBDs: "zf-C4"]<br>Possible TFs: RXRA	*8.14643*	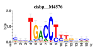	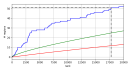	link	link	PWMs
36	☐ cisbp__M3402<br>Description: HNF1A[gene ID: "ENSG00000135100" species: "Homo sapiens" TF status: "direct" TF family: "Homeodomain" DBDs: "Homeobox"]<br>Possible TFs: HNF1A	*8.12112*	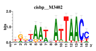	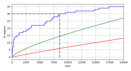	link	link	PWMs
37	☐ cisbp__M5523<br>Description: HNF1B[gene ID: "ENSG00000108753" species: "Homo sapiens" TF status: "direct" TF family: "Homeodomain" DBDs: "Homeobox"]	*8.11266*	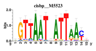	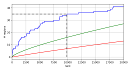	link	link	PWMs

#	Feature	NES	Logo	Recovery Curve	Candidate targets	All regions in top 20000	Database
38	☐ transfac_pro__M01011<br>Description: V$HNF1_Q6_01: HNF-1<br>Possible TFs: HNF1B, HNF1A	*8.04311*	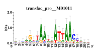	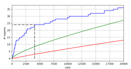	link	link	PWMs
39	☐ homer__CAGAGGNCAAAGTCCA_HNF4a<br>Description: HNF4a(NR),DR1/HepG2-HNF4a-ChIP-Seq(GSE25021)/Homer<br>Possible TFs: HNF4A	*7.93318*	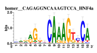	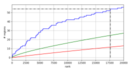	link	link	PWMs
40	☐ taipale__HNF1B_full_NRTTAATNATTAACN<br>Description: HNF1B dimeric homeodomain full<br>Possible TFs: HNF1B	*7.84904*	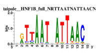	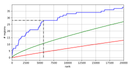	link	link	PWMs
41	☐ factorbook__RXRA<br>Description: RXRA<br>Possible TFs: RXRA	*7.84308*	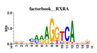	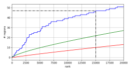	link	link	PWMs
42	☐ transfac_pro__M00790<br>Description: V$HNF1_Q6: HNF-1<br>Possible TFs: HNF1B, HNF1A	*7.82261*	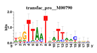	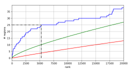	link	link	PWMs
43	☐ swissregulon__hs__HNF4A_NR2F1_2.p2<br>Description: hs__HNF4A_NR2F1,2.p2<br>Possible TFs: NR2F1, HNF4A, NR2F2	*7.81592*	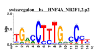	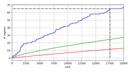	link	link	PWMs
44	☐ jaspar__MA0484.1<br>Description: HNF4G<br>Possible TFs: HNF4G	*7.71494*	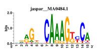	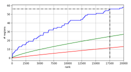	link	link	PWMs
45	☐ cisbp__M2286<br>Description: HNF4G[gene ID: "ENSG00000164749" species: "Homo sapiens" TF status: "direct" TF family: "Nuclear receptor" DBDs: "zf-C4"]; Hnf4g[gene ID: "ENSMUSG00000017688" species: "Mus musculus" TF status: "inferred" TF family: "Nuclear receptor" DBDs: "zf-C4"]<br>Possible TFs: HNF4G	*7.69697*	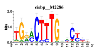	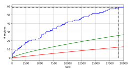	link	link	PWMs
46	☐ transfac_public__M00134<br>Description: V$HNF4_01: HNF-4<br>Possible TFs: HNF4A	*7.62959*	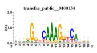	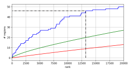	link	link	PWMs
47	☐ homer__GGTTAAACATTAAC_Hnf1<br>Description: Hnf1(Homeobox)/Liver-Foxa2-Chip-Seq(GSE25694)/Homer<br>Possible TFs: HNF1A	*7.59163*	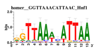	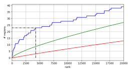	link	link	PWMs
48	☐ cisbp__M5521<br>Description: HNF1A[gene ID: "ENSG00000135100" species: "Homo sapiens" TF status: "direct" TF family: "Homeodomain" DBDs: "Homeobox"]<br>Possible TFs: HNF1A	*7.57632*	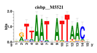	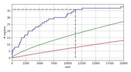	link	link	PWMs
49	☐ transfac_pro__M02016<br>Description: V$HNF4A_Q6_01: HNF-4alpha<br>Possible TFs: HNF4A	*7.49637*	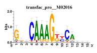	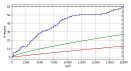	link	link	PWMs
50	☐ cisbp__M3415<br>Description: HNF4A[gene ID: "ENSG00000101076" species: "Homo sapiens" TF status: "direct" TF family: "Nuclear receptor" DBDs: "zf-C4"]; Hnf4g[gene ID: "ENSMUSG00000017688" species: "Mus musculus" TF status: "inferred" TF family: "Nuclear receptor" DBDs: "zf-C4"]<br>Possible TFs: HNF4A	*7.47977*	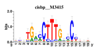	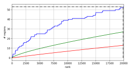	link	link	PWMs
51	☐ transfac_pro__M01033<br>Description: V$HNF4_Q6_03: HNF-4<br>Possible TFs: HNF4A	*7.45253*	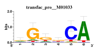	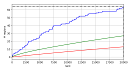	link	link	PWMs
52	☐ cisbp__M5522<br>Description: HNF1B[gene ID: "ENSG00000108753" species: "Homo sapiens" TF status: "direct" TF family: "Homeodomain" DBDs: "Homeobox"]	*7.40998*	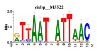	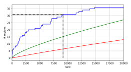	link	link	PWMs
53	☐ ENCFF001UGI<br>Description: HNF4G ChIP-seq protocol v041610.1 on human HepG2	*7.39519*		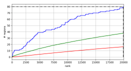	link	link	TF binding sites

#	Feature	NES	Logo	Recovery Curve	Candidate targets	All regions in top 20000	Database
54	☐ cisbp__M6284<br>Description: HNF4G[gene ID: "ENSG00000164749" species: "Homo sapiens" TF status: "direct" TF family: "Nuclear receptor" DBDs: "zf-C4"]; Hnf4g[gene ID: "ENSMUSG00000017688" species: "Mus musculus" TF status: "inferred" TF family: "Nuclear receptor" DBDs: "zf-C4"]<br>Possible TFs: HNF4G	7.32149	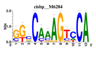	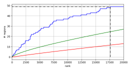	link	link	PWMs
55	☐ taipale__HNF1A_full_NRTTAATNATTAACN_repr<br>Description: HNF1A dimeric homeodomain full<br>Possible TFs: HNF1A	7.26685	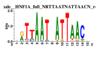	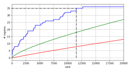	link	link	PWMs
56	☐ transfac_pro__M08805<br>Description: V$EAR2_Q4: EAR2<br>Possible TFs: NR2F6	7.19294	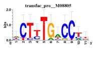	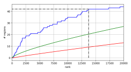	link	link	PWMs
57	☐ transfac_pro__M07044<br>Description: V$HNF1A_Q4: HNF-1alpha<br>Possible TFs: HNF1A	7.18376	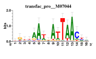	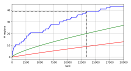	link	link	PWMs
58	☐ taipale__HNF1B_full_GTTAATNATTAAY<br>Description: HNF1B dimeric homeodomain full<br>Possible TFs: HNF1B	7.18150	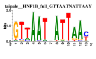	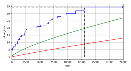	link	link	PWMs
59	☐ transfac_pro__M03872<br>Description: V$HNF1B_Q6_01: HNF-1beta<br>Possible TFs: HNF1B	7.15901	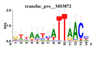	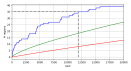	link	link	PWMs
60	☐ hocomoco__HNF1B_MOUSE.H11MO.1.A<br>Description: HNF1B_MOUSE<br>Possible TFs: HNF1B	7.05465	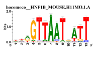	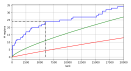	link	link	PWMs
61	☐ transfac_pro__M04850<br>Description: V$RAD21_10: Rad21<br>Possible TFs: RAD21	7.00291	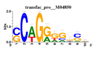	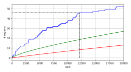	link	link	PWMs
62	☐ transfac_pro__M02013<br>Description: V$HNF1A_Q5: HNF-1alpha<br>Possible TFs: HNF1A	6.92925	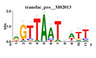	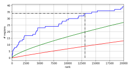	link	link	PWMs
63	☐ jaspar__MA0065.2<br>Description: Pparg::Rxra<br>Possible TFs: RXRA	6.88178	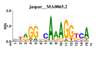	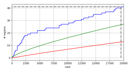	link	link	PWMs
64	☐ ENCFF001UHJ<br>Description: RXRA ChIP-seq protocol PCR1x on human HepG2	6.81861		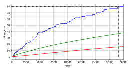	link	link	TF binding sites
65	☐ ENCFF001UGH<br>Description: HNF4A ChIP-seq protocol v041610.1 on human HepG2	6.79004		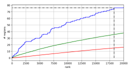	link	link	TF binding sites
66	☐ ENCFF001UGG<br>Description: HNF4A ChIP-seq protocol v041610.1 on human HepG2	6.78438		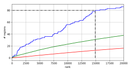	link	link	TF binding sites
67	☐ transfac_pro__M03790<br>Description: V$FXR_Q5: FXR<br>Possible TFs: NR1H4	6.77105	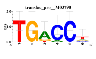	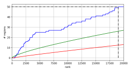	link	link	PWMs
68	☐ transfac_pro__M03826<br>Description: V$HNF1B_Q6: HNF-1beta<br>Possible TFs: HNF1B	6.65443	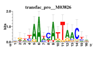	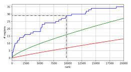	link	link	PWMs
69	☐ hocomoco__HNF1B_HUMAN.H11MO.1.A<br>Description: HNF1B_HUMAN<br>Possible TFs: HNF1B	6.60229	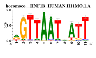	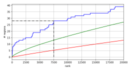	link	link	PWMs
70	☐ E118-H3K27ac-broadpeak<br>Description: H3K27ac in HepG2 Hepatocellular Carcinoma Cell Line (E118, broadpeak)	6.49617		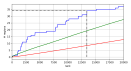	link	link	Histone modifications
71	☐ dbcorrdb__SP1__ENCSR000BJX_1__m1<br>Description: SP1 (ENCSR000BJX-1, motif 1)<br>Possible TFs: SP1	6.45166	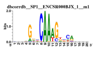	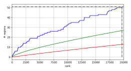	link	link	PWMs
72	☐ taipale__HNF4A_full_NRGTCCAAAGTCCANY<br>Description: HNF4A dimeric nuclearreceptor full<br>Possible TFs: HNF4A	6.35334	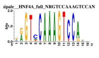	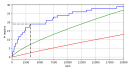	link	link	PWMs

#	Feature	NES	Logo	Recovery Curve	Candidate targets	All regions in top 20000	Database
73	☐ ENCFF001UFK<br>Description: CEBPD ChIP-seq protocol v041610.1 on human HepG2	6.31678		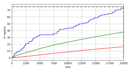	link	link	TF binding sites
74	☐ taipale_cyt_meth__HNF4A_NRGTCCAAAGTCCRN_eDBD_meth_repr<br>Description: HNF4A [Nuclear_receptor, CpG-meth]<br>Possible TFs: HNF4A	6.30563	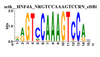	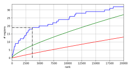	link	link	PWMs
75	☐ cisbp__M5526<br>Description: HNF4A[gene ID: "ENSG00000101076" species: "Homo sapiens" TF status: "direct" TF family: "Nuclear receptor" DBDs: "zf-C4"]; Hnf4g[gene ID: "ENSMUSG00000017688" species: "Mus musculus" TF status: "inferred" TF family: "Nuclear receptor" DBDs: "zf-C4"]<br>Possible TFs: HNF4A	6.30168	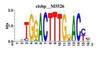	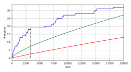	link	link	PWMs
76	☐ elemento__CTGGGCC<br>Description: Conserved regulatory element CTGGGCC between Hs and Mm	6.24478	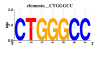	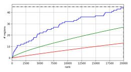	link	link	PWMs
77	☐ transfac_pro__M02266<br>Description: V$HNF1B_04: HNF1B<br>Possible TFs: HNF1B	6.24228	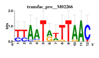	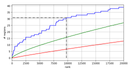	link	link	PWMs
78	☐ ENCFF001UIG<br>Description: ZBTB7A ChIP-seq protocol v041610.1 on human HepG2	6.21625		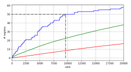	link	link	TF binding sites
79	☐ ENCFF001UGS<br>Description: NFIC ChIP-seq protocol v042211.1 on human HepG2	6.18568		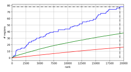	link	link	TF binding sites
80	☐ hdpi__MTHFD1<br>Description: MTHFD1<br>Possible TFs: MTHFD1	6.15145	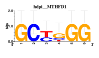	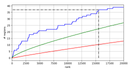	link	link	PWMs
81	☐ homer__TAGGGCAAAGGTCA_RXR<br>Description: RXR(NR),DR1/3T3L1-RXR-ChIP-Seq(GSE13511)/Homer	6.10592	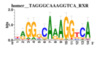	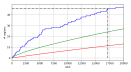	link	link	PWMs
82	☐ transfac_pro__M01728<br>Description: V$EAR2_Q2: EAR2<br>Possible TFs: NR2F6	6.09875	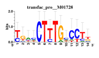	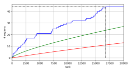	link	link	PWMs
83	☐ E085-H3K4me1<br>Description: H3K4me1 in Fetal Intestine Small (E085,)	6.03514		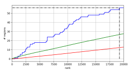	link	link	Histone modifications
84	☐ taipale__HNF4A_full_RRGGTCAAAGTCCRNN_repr<br>Description: HNF4A dimeric nuclearreceptor full<br>Possible TFs: HNF4A	6.01485	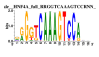	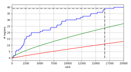	link	link	PWMs
85	☐ taipale_cyt_meth__HNF4A_NRGTCCAAAGTCCRN_eDBD<br>Description: HNF4A [Nuclear_receptor]<br>Possible TFs: HNF4A	5.96287	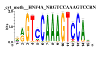	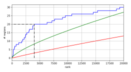	link	link	PWMs
86	☐ transfac_pro__M00762<br>Description: V$DR1_Q3: PPAR, HNF-4, COUP, RAR<br>Possible TFs: HNF4A, HNF4G, NR2F1, RXRA, NR2F2, PPARG	5.95134	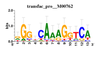	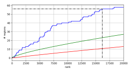	link	link	PWMs
87	☐ ENCFF001UIH<br>Description: ZBTB7A ChIP-seq protocol v041610.1 on human HepG2	5.94382		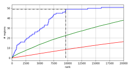	link	link	TF binding sites
88	☐ hocomoco__RARA_MOUSE.H11MO.3.A<br>Description: RARA_MOUSE<br>Possible TFs: RARA	5.94006	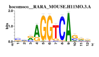	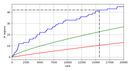	link	link	PWMs
89	☐ hdpi__NAP1L1<br>Description: NAP1L1<br>Possible TFs: NAP1L1	5.87478	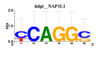	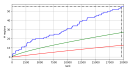	link	link	PWMs
90	☐ hdpi__RBM17<br>Description: RBM17<br>Possible TFs: RBM17	5.86422	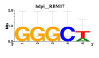	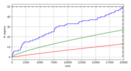	link	link	PWMs

#	Feature	NES	Logo	Recovery Curve	Candidate targets	All regions in top 20000	Database
91	☐ cisbp__M5528<br>Description: HNF4A[gene ID: "ENSG00000101076" species: "Homo sapiens" TF status: "direct" TF family: "Nuclear receptor" DBDs: "zf-C4"]; Hnf4g[gene ID: "ENSMUSG00000017688" species: "Mus musculus" TF status: "inferred" TF family: "Nuclear receptor" DBDs: "zf-C4"]<br>Possible TFs: HNF4A	*5.85939*	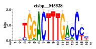	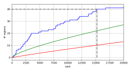	link	link	PWMs
92	☐ transfac_public__M00411<br>Description: V$HNF4_01_B: HNF4alpha1<br>Possible TFs: HNF4A	*5.85544*	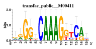	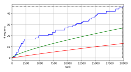	link	link	PWMs
93	☐ E109-DNase.hotspot.all.peaks-narrowpeak<br>Description: DNase in Small Intestine (E109, narrowpeak, hotspot)	*5.85337*		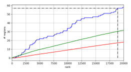	link	link	DHS & FAIRE
94	☐ transfac_pro__M01032<br>Description: V$HNF4_Q6_02: HNF-4<br>Possible TFs: HNF4A	*5.83795*	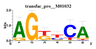	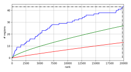	link	link	PWMs
95	☐ hdpi__TRIM21<br>Description: TRIM21<br>Possible TFs: TRIM21	*5.80402*	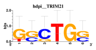	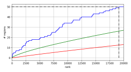	link	link	PWMs
96	☐ taipale_cyt_meth__HNF4A_NRGGTCAAAGTCCRN_eDBD_meth_repr<br>Description: HNF4A [Nuclear_receptor, CpG-meth]<br>Possible TFs: HNF4A	*5.79886*	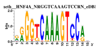	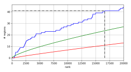	link	link	PWMs
97	☐ dbcorrdb__RXRA__ENCSR000BHU_1__m2<br>Description: RXRA (ENCSR000BHU-1, motif 2)<br>Possible TFs: RXRA	*5.75808*	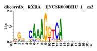	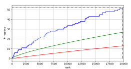	link	link	PWMs
98	☐ c2h2_zfs__M0442<br>Description: NCU05064	*5.74479*	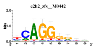	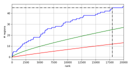	link	link	PWMs
99	☐ E098-DNase.hotspot.all.peaks-narrowpeak<br>Description: DNase in Pancreas (E098, narrowpeak, hotspot)	*5.69239*		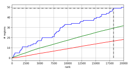	link	link	DHS & FAIRE
100	☐ taipale__Hnf4a_DBD_RRGGTCAAAGTCCRNN<br>Description: Hnf4a dimeric nuclearreceptor DBD<br>Possible TFs: HNF4A	*5.66065*	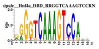	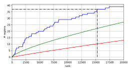	link	link	PWMs
101	☐ ENCFF001UGJ<br>Description: HNF4G ChIP-seq protocol v041610.1 on human HepG2	*5.64628*		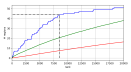	link	link	TF binding sites
102	☐ homer__TGACCTTTGCCCCA_PPARE<br>Description: PPARE(NR),DR1/3T3L1-Pparg-ChIP-Seq(GSE13511)/Homer<br>Possible TFs: PPARG	*5.62027*	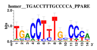	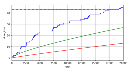	link	link	PWMs
103	☐ transfac_pro__M00763<br>Description: V$PPARDR1_Q2: PPAR direct repeat 1<br>Possible TFs: PPARG, PPARD, RXRA, PPARA	*5.60262*	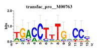	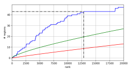	link	link	PWMs
104	☐ cisbp__M6460<br>Description: AL662824.5[gene ID: "ENSG00000227322" species: "Homo sapiens" TF status: "inferred" TF family: "Nuclear receptor" DBDs: "zf-C4"]; AL844527.7[gene ID: "ENSG00000206289" species: "Homo sapiens" TF status: "inferred" TF family: "Nuclear receptor" DBDs: "zf-C4"]; CR354565.3[gene ID: "ENSG00000231321" species: "Homo sapiens" TF status: "inferred" TF family: "Nuclear receptor" DBDs: "zf-C4"]; CR759733.4[gene ID: "ENSG00000228333" species: "Homo sapiens" TF status: "inferred" TF family: "Nuclear receptor" DBDs: "zf-C4"]; CR936877.3[gene ID: "ENSG00000235712" species: "Homo sapiens" TF status: "inferred" TF family: "Nuclear receptor" DBDs: "zf-C4"]; RXRA[gene ID: "ENSG00000186350" species: "Homo sapiens" TF status: "direct" TF family: "Nuclear receptor" DBDs: "zf-C4"]<br>Possible TFs: RXRA	*5.59166*	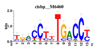	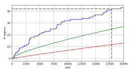	link	link	PWMs
105	☐ cisbp__M1443<br>Description: Hnf4a[gene ID: "ENSMUSG00000017950" species: "Mus musculus" TF status: "direct" TF family: "Nuclear receptor" DBDs: "zf-C4"]; Hnf4g[gene ID: "ENSMUSG00000017688" species: "Mus musculus" TF status: "inferred" TF family: "Nuclear receptor" DBDs: "zf-C4"]<br>Possible TFs: HNF4A	*5.57417*	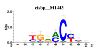	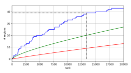	link	link	PWMs

#	Feature	NES	Logo	Recovery Curve	Candidate targets	All regions in top 20000	Database
106	☐ cisbp__M4697<br>Description: ESR1[gene ID: "ENSG00000091831" species: "Homo sapiens" TF status: "direct" TF family: "Nuclear receptor" DBDs: "zf-C4"]; Esr2[gene ID: "ENSMUSG00000021055" species: "Mus musculus" TF status: "inferred" TF family: "Nuclear receptor" DBDs: "zf-C4"]<br>Possible TFs: ESR1	*5.56466*	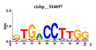	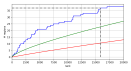	**link**	**link**	PWMs
107	☐ dbcorrdb__EP300__ENCSR000EDV_1__m1<br>Description: EP300 (ENCSR000EDV-1, motif 1)<br>Possible TFs: EP300	*5.53452*	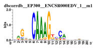	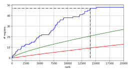	**link**	**link**	PWMs
108	☐ cisbp__M6232<br>Description: CG11152[gene ID: "FBgn0039937" species: "Drosophila melanogaster" TF status: "inferred" TF family: "Forkhead" DBDs: "Fork_head"]; CG9571[gene ID: "FBgn0031086" species: "Drosophila melanogaster" TF status: "inferred" TF family: "Forkhead" DBDs: "Fork_head"]; FOXA1[gene ID: "ENSG00000129514" species: "Homo sapiens" TF status: "direct" TF family: "Forkhead" DBDs: "Fork_head"]; FOXB2[gene ID: "ENSG00000204612" species: "Homo sapiens" TF status: "inferred" TF family: "Forkhead" DBDs: "Fork_head"]; FOXD4L1[gene ID: "ENSG00000184492" species: "Homo sapiens" TF status: "inferred" TF family: "Forkhead" DBDs: "Fork_head"]; FOXD4L3[gene ID: "ENSG00000187559" species: "Homo sapiens" TF status: "inferred" TF family: "Forkhead" DBDs: "Fork_head"]; FOXD4L5[gene ID: "ENSG00000204779" species: "Homo sapiens" TF status: "inferred" TF family: "Forkhead" DBDs: "Fork_head"]; FOXD4L6[gene ID: "ENSG00000204793" species: "Homo sapiens" TF status: "inferred" TF family: "Forkhead" DBDs: "Fork_head"]; FOXD4[gene ID: "ENSG00000170122" species: "Homo sapiens" TF status: "inferred" TF family: "Forkhead" DBDs: "Fork_head"]; FOXE1[gene ID: "ENSG00000178919" species: "Homo sapiens" TF status: "inferred" TF family: "Forkhead" DBDs: "Fork_head"]; FOXE3[gene ID: "ENSG00000186790" species: "Homo sapiens" TF status: "inferred" TF family: "Forkhead" DBDs: "Fork_head"]; FOXI2[gene ID: "ENSG00000186766" species: "Homo sapiens" TF status: "inferred" TF family: "Forkhead" DBDs: "Fork_head"]; FOXK2[gene ID: "ENSG00000141568" species: "Homo sapiens" TF status: "inferred" TF family: "Forkhead" DBDs: "Fork_head"]; FOXS1[gene ID: "ENSG00000179772" species: "Homo sapiens" TF status: "inferred" TF family: "Forkhead" DBDs: "Fork_head"]; Foxa1[gene ID: "ENSMUSG00000035451" species: "Mus musculus" TF status: "inferred" TF family: "Forkhead" DBDs: "Fork_head"]; Foxa3[gene ID: "ENSMUSG00000040891" species: "Mus musculus" TF status: "inferred" TF family: "Forkhead" DBDs: "Fork_head"]; Foxb2[gene ID: "ENSMUSG00000056829" species: "Mus musculus" TF status: "inferred" TF family: "Forkhead" DBDs: "Fork_head"]; Foxd2[gene ID: "ENSMUSG00000055210" species: "Mus musculus" TF status: "inferred" TF family: "Forkhead" DBDs: "Fork_head"]; Foxd3[gene ID: "ENSMUSG00000067261" species: "Mus musculus" TF status: "inferred" TF family: "Forkhead" DBDs: "Fork_head"]; Foxd4[gene ID: "ENSMUSG00000051490" species: "Mus musculus" TF status: "inferred" TF family: "Forkhead" DBDs: "Fork_head"]; Foxe1[gene ID: "ENSMUSG00000070990" species: "Mus musculus" TF status: "inferred" TF family: "Forkhead" DBDs: "Fork_head"]; Foxe3[gene ID: "ENSMUSG00000044518" species: "Mus musculus" TF status: "inferred" TF family: "Forkhead" DBDs: "Fork_head"]; Foxf1a[gene ID: "ENSMUSG00000042812" species: "Mus musculus" TF status: "inferred" TF family: "Forkhead" DBDs: "Fork_head"]; Foxf2[gene ID: "ENSMUSG00000038402" species: "Mus musculus" TF status: "inferred" TF family: "Forkhead" DBDs: "Fork_head"]; Foxi1[gene ID: "ENSMUSG00000047861" species: "Mus musculus" TF status: "inferred" TF family: "Forkhead" DBDs: "Fork_head"]; Foxi2[gene ID: "ENSMUSG00000048377" species: "Mus musculus" TF status: "inferred" TF family: "Forkhead" DBDs: "Fork_head"]; Foxi3[gene ID: "ENSMUSG00000055874" species: "Mus musculus" TF status: "inferred" TF family: "Forkhead" DBDs: "Fork_head"]; Foxk2[gene ID: "ENSMUSG00000039275" species: "Mus musculus" TF status: "inferred" TF family: "Forkhead" DBDs: "Fork_head"]; Foxl2[gene ID: "ENSMUSG00000050397" species: "Mus musculus" TF status: "inferred" TF family: "Forkhead" DBDs: "Fork_head"]; Foxq1[gene ID: "ENSMUSG00000038415" species: "Mus musculus" TF status: "inferred" TF	*5.50366*	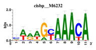	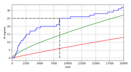	**link**	**link**	PWMs

#	Feature	NES	Logo	Recovery Curve	Candidate targets	All regions in top 20000	Database
	family: "Forkhead" DBDs: "Fork_head"]; Foxs1[gene ID: "ENSMUSG00000074676" species: "Mus musculus" TF status: "inferred" TF family: "Forkhead" DBDs: "Fork_head"]; KIAA0415[gene ID: "ENSG00000242802" species: "Homo sapiens" TF status: "inferred" TF family: "Forkhead" DBDs: "Fork_head"]; Mnf[gene ID: "FBgn0036134" species: "Drosophila melanogaster" TF status: "inferred" TF family: "Forkhead" DBDs: "Fork_head"]; fd59A[gene ID: "FBgn0004896" species: "Drosophila melanogaster" TF status: "inferred" TF family: "Forkhead" DBDs: "Fork_head"]; fd96Ca[gene ID: "FBgn0004897" species: "Drosophila melanogaster" TF status: "inferred" TF family: "Forkhead" DBDs: "Fork_head"]; fd96Cb[gene ID: "FBgn0004898" species: "Drosophila melanogaster" TF status: "inferred" TF family: "Forkhead" DBDs: "Fork_head"]<br>Possible TFs: FOXA1						
109	☐ taipale_cyt_meth__HNF4A_NRGGTCAAAGTCCRN_eDBD<br>Description: HNF4A [Nuclear_receptor]<br>Possible TFs: HNF4A	*5.48810*	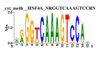	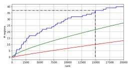	**link**	**link**	PWMs
110	☐ transfac_pro__M07958<br>Description: I$HNF4_01: Hnf-4<br>Possible TFs: HNF4G, HNF4A	*5.46618*	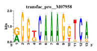	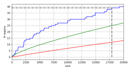	**link**	**link**	PWMs
111	☐ neph__UW.Motif.0032<br>Description: cagcccag	*5.43136*	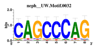	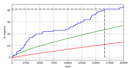	**link**	**link**	PWMs
112	☐ bergman__Dip3<br>Description: Dip3	*5.42403*	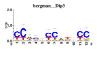	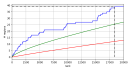	**link**	**link**	PWMs
113	☐ dbcorrdb__HDAC2__ENCSR000BMC_1__m1<br>Description: HDAC2 (ENCSR000BMC-1, motif 1)<br>Possible TFs: HDAC2	*5.40549*	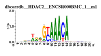	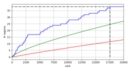	**link**	**link**	PWMs
114	☐ transfac_pro__M01721<br>Description: V$PUR1_Q4: PUR1<br>Possible TFs: PURA	*5.38357*	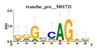	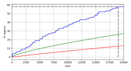	**link**	**link**	PWMs
115	☐ transfac_pro__M08906<br>Description: V$TR4_Q3: TR4<br>Possible TFs: NR2C2	*5.33393*	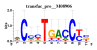	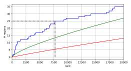	**link**	**link**	PWMs
116	☐ transfac_pro__M00764<br>Description: V$HNF4DR1_Q3: HNF4 direct repeat 1<br>Possible TFs: HNF4G, HNF4A	*5.32950*	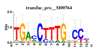	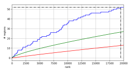	**link**	**link**	PWMs
117	☐ ENCFF001UHU<br>Description: TCF12 ChIP-seq protocol PCR1x on human HepG2	*5.32912*		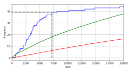	**link**	**link**	TF binding sites

#	Feature	NES	Logo	Recovery Curve	Candidate targets	All regions in top 20000	Database
118	☐ cisbp__M2339<br>Description: CG11152[gene ID: "FBgn0039937" species: "Drosophila melanogaster" TF status: "inferred" TF family: "Forkhead" DBDs: "Fork_head"]; CG9571[gene ID: "FBgn0031086" species: "Drosophila melanogaster" TF status: "inferred" TF family: "Forkhead" DBDs: "Fork_head"]; FOXB2[gene ID: "ENSG00000204612" species: "Homo sapiens" TF status: "inferred" TF family: "Forkhead" DBDs: "Fork_head"]; FOXD4L1[gene ID: "ENSG00000184492" species: "Homo sapiens" TF status: "inferred" TF family: "Forkhead" DBDs: "Fork_head"]; FOXD4L3[gene ID: "ENSG00000187559" species: "Homo sapiens" TF status: "inferred" TF family: "Forkhead" DBDs: "Fork_head"]; FOXD4L5[gene ID: "ENSG00000204779" species: "Homo sapiens" TF status: "inferred" TF family: "Forkhead" DBDs: "Fork_head"]; FOXD4L6[gene ID: "ENSG00000204793" species: "Homo sapiens" TF status: "inferred" TF family: "Forkhead" DBDs: "Fork_head"]; FOXD4[gene ID: "ENSG00000170122" species: "Homo sapiens" TF status: "inferred" TF family: "Forkhead" DBDs: "Fork_head"]; FOXE1[gene ID: "ENSG00000178919" species: "Homo sapiens" TF status: "inferred" TF family: "Forkhead" DBDs: "Fork_head"]; FOXE3[gene ID: "ENSG00000186790" species: "Homo sapiens" TF status: "inferred" TF family: "Forkhead" DBDs: "Fork_head"]; FOXI2[gene ID: "ENSG00000186766" species: "Homo sapiens" TF status: "inferred" TF family: "Forkhead" DBDs: "Fork_head"]; FOXK2[gene ID: "ENSG00000141568" species: "Homo sapiens" TF status: "inferred" TF family: "Forkhead" DBDs: "Fork_head"]; FOXS1[gene ID: "ENSG00000179772" species: "Homo sapiens" TF status: "inferred" TF family: "Forkhead" DBDs: "Fork_head"]; Foxa1[gene ID: "ENSMUSG00000035451" species: "Mus musculus" TF status: "inferred" TF family: "Forkhead" DBDs: "Fork_head"]; Foxa3[gene ID: "ENSMUSG00000040891" species: "Mus musculus" TF status: "inferred" TF family: "Forkhead" DBDs: "Fork_head"]; Foxb2[gene ID: "ENSMUSG00000056829" species: "Mus musculus" TF status: "inferred" TF family: "Forkhead" DBDs: "Fork_head"]; Foxd2[gene ID: "ENSMUSG00000055210" species: "Mus musculus" TF status: "inferred" TF family: "Forkhead" DBDs: "Fork_head"]; Foxd3[gene ID: "ENSMUSG00000067261" species: "Mus musculus" TF status: "inferred" TF family: "Forkhead" DBDs: "Fork_head"]; Foxd4[gene ID: "ENSMUSG00000051490" species: "Mus musculus" TF status: "inferred" TF family: "Forkhead" DBDs: "Fork_head"]; Foxe1[gene ID: "ENSMUSG00000070990" species: "Mus musculus" TF status: "inferred" TF family: "Forkhead" DBDs: "Fork_head"]; Foxe3[gene ID: "ENSMUSG00000044518" species: "Mus musculus" TF status: "inferred" TF family: "Forkhead" DBDs: "Fork_head"]; Foxf1a[gene ID: "ENSMUSG00000042812" species: "Mus musculus" TF status: "inferred" TF family: "Forkhead" DBDs: "Fork_head"]; Foxf2[gene ID: "ENSMUSG00000038402" species: "Mus musculus" TF status: "inferred" TF family: "Forkhead" DBDs: "Fork_head"]; Foxi1[gene ID: "ENSMUSG00000047861" species: "Mus musculus" TF status: "inferred" TF family: "Forkhead" DBDs: "Fork_head"]; Foxi2[gene ID: "ENSMUSG00000048377" species: "Mus musculus" TF status: "inferred" TF family: "Forkhead" DBDs: "Fork_head"]; Foxi3[gene ID: "ENSMUSG00000055874" species: "Mus musculus" TF status: "inferred" TF family: "Forkhead" DBDs: "Fork_head"]; Foxk2[gene ID: "ENSMUSG00000039275" species: "Mus musculus" TF status: "inferred" TF family: "Forkhead" DBDs: "Fork_head"]; Foxl2[gene ID: "ENSMUSG00000050397" species: "Mus musculus" TF status: "inferred" TF family: "Forkhead" DBDs: "Fork_head"]; Foxs1[gene ID: "ENSMUSG00000074676" species: "Mus musculus" TF status: "inferred" TF family: "Forkhead" DBDs: "Fork_head"]; KIAA0415[gene ID: "ENSG00000242802" species: "Homo sapiens" TF status: "inferred" TF family: "Forkhead" DBDs: "Fork_head"]; Mnf[gene ID: "FBgn0036134" species: "Drosophila melanogaster" TF status: "inferred" TF family: "Forkhead" DBDs: "Fork_head"]; fd59A[gene ID: "FBgn0004896" species: "Drosophila melanogaster" TF status: "inferred" TF family: "Forkhead" DBDs: "Fork_head"]; fd96Ca[gene ID: "FBgn0004897" species: "Drosophila melanogaster" TF status: "inferred" TF family: "Forkhead" DBDs: "Fork_head"]; fd96Cb[gene ID: "FBgn0004898" species: "Drosophila melanogaster" TF status: "inferred" TF family: "Forkhead" DBDs: "Fork_head"]	*5.30209*	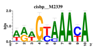	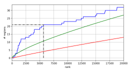	link	link	PWMs

#	Feature	NES	Logo	Recovery Curve	Candidate targets	All regions in top 20000	Database
119	☐ E118-H3K9ac<br>Description: H3K9ac in HepG2 Hepatocellular Carcinoma Cell Line (E118,)	*5.29819*		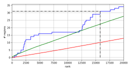	link	link	Histone modifications
120	☐ transfac_public__M00206<br>Description: V$HNF1_C: HNF-1<br>Possible TFs: HNF1A	*5.27961*	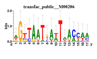	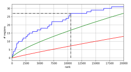	link	link	PWMs
121	☐ cisbp__M5525<br>Description: HNF4A[gene ID: "ENSG00000101076" species: "Homo sapiens" TF status: "direct" TF family: "Nuclear receptor" DBDs: "zf-C4"]; Hnf4g[gene ID: "ENSMUSG00000017688" species: "Mus musculus" TF status: "inferred" TF family: "Nuclear receptor" DBDs: "zf-C4"]<br>Possible TFs: HNF4A	*5.22803*	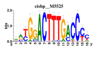	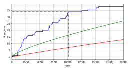	link	link	PWMs
122	☐ ENCFF001UFV<br>Description: FOXA1 ChIP-seq protocol v041610.1 on human HepG2	*5.21887*		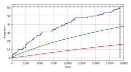	link	link	TF binding sites
123	☐ hdpi__SMAP1L<br>Description: SMAP1L<br>Possible TFs: SMAP2	*5.21377*	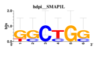	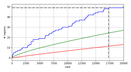	link	link	PWMs
124	☐ hdpi__NXPH3<br>Description: NXPH3<br>Possible TFs: NXPH3	*5.20498*	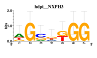	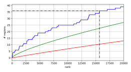	link	link	PWMs
125	☐ ENCFF001UHF<br>Description: POLR2A ChIP-seq protocol PCR2x on human HepG2	*5.19586*		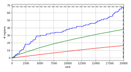	link	link	TF binding sites
126	☐ transfac_pro__M00963<br>Description: V$T3R_Q6: T3R<br>Possible TFs: RARA, RARB, RARG, RXRG, THRB, RXRA, RXRB, THRA	*5.19056*	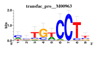	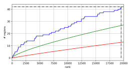	link	link	PWMs
127	☐ taipale__HNF4A_DBD_RRGGTCAAAGTCCRNN<br>Description: HNF4A dimeric nuclearreceptor DBD<br>Possible TFs: HNF4A	*5.18765*	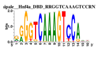	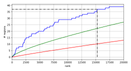	link	link	PWMs
128	☐ hocomoco__RXRA_HUMAN.H11MO.1.A<br>Description: RXRA_HUMAN<br>Possible TFs: RXRA	*5.16009*	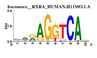	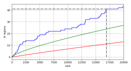	link	link	PWMs
129	☐ ENCFF001UGQ<br>Description: MYBL2 ChIP-seq protocol v042211.1 on human HepG2	*5.15696*		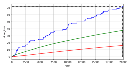	link	link	TF binding sites
130	☐ dbcorrdb__EP300__ENCSR000BLM_1__m1<br>Description: EP300 (ENCSR000BLM-1, motif 1)<br>Possible TFs: EP300	*5.15244*	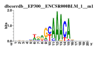	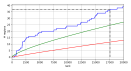	link	link	PWMs
131	☐ hocomoco__COT1_HUMAN.H11MO.1.C<br>Description: COT1_HUMAN<br>Possible TFs: NR2F1	*5.14534*	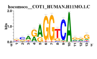	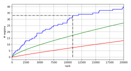	link	link	PWMs
132	☐ jaspar__MA0546.1<br>Description: pha-4	*5.09658*	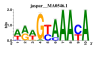	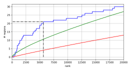	link	link	PWMs
133	☐ transfac_pro__M07602<br>Description: V$CP2_Q4: CP2<br>Possible TFs: TFCP2	*5.09505*	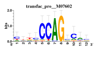	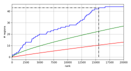	link	link	PWMs
134	☐ cisbp__M6390<br>Description: NR2C2[gene ID: "ENSG00000177463" species: "Homo sapiens" TF status: "direct" TF family: "Nuclear receptor" DBDs: "zf-C4"]<br>Possible TFs: NR2C2	*5.08055*	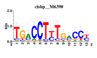	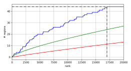	link	link	PWMs
135	☐ dbcorrdb__MYBL2__ENCSR000BRO_1__m2<br>Description: MYBL2 (ENCSR000BRO-1, motif 2)<br>Possible TFs: MYBL2	*5.06483*	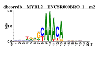	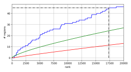	link	link	PWMs
136	☐ transfac_pro__M07959<br>Description: I$HNF4_02: Hnf-4<br>Possible TFs: HNF4G, HNF4A	*5.05290*	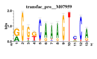	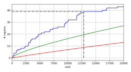	link	link	PWMs
137	☐ ENCFF001UGC<br>Description: HDAC2 ChIP-seq protocol v041610.1 on human HepG2	*5.01469*		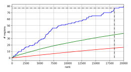	link	link	TF binding sites

#	Feature	NES	Logo	Recovery Curve	Candidate targets	All regions in top 20000	Database
138	☐ E094-DNase.macs2-narrowpeak<br>Description: DNase in Gastric (E094, narrowpeak, macs2)	*5.01400*		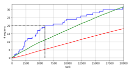	link	link	DHS & FAIRE
139	☐ hocomoco__RARA_HUMAN.H11MO.1.A<br>Description: RARA_HUMAN<br>Possible TFs: RARA	*5.01164*	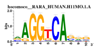	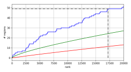	link	link	PWMs
140	☐ dbcorrdb__TCF12__ENCSR000BJG_1__m2<br>Description: TCF12 (ENCSR000BJG-1, motif 2)<br>Possible TFs: TCF12	*5.00632*	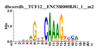	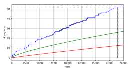	link	link	PWMs
141	☐ hdpi__PIR<br>Description: PIR<br>Possible TFs: PIR	*4.98642*	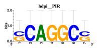	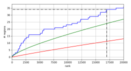	link	link	PWMs
142	☐ transfac_pro__M07960<br>Description: I$HNF4_03: Hnf-4<br>Possible TFs: HNF4G, HNF4A	*4.98593*	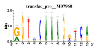	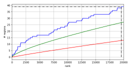	link	link	PWMs
143	☐ hdpi__NCALD<br>Description: NCALD<br>Possible TFs: NCALD	*4.96796*	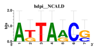	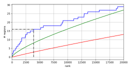	link	link	PWMs
144	☐ E114-H3K4me1<br>Description: H3K4me1 in A549 EtOH 0.02pct Lung Carcinoma Cell Line (E114,)	*4.92257*		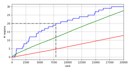	link	link	Histone modifications
145	☐ E102-H3K4me1<br>Description: H3K4me1 in Rectal Mucosa Donor 31 (E102,)	*4.91902*		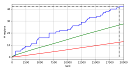	link	link	Histone modifications
146	☐ cisbp__M6176<br>Description: NR2F1[gene ID: "ENSG00000175745" species: "Homo sapiens" TF status: "direct" TF family: "Nuclear receptor" DBDs: "zf-C4"]<br>Possible TFs: NR2F1	*4.89913*	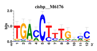	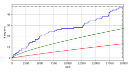	link	link	PWMs
147	☐ hocomoco__COT1_MOUSE.H11MO.1.C<br>Description: COT1_MOUSE	*4.86746*	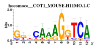	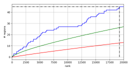	link	link	PWMs
148	☐ E094-DNase.hotspot.all.peaks-narrowpeak<br>Description: DNase in Gastric (E094, narrowpeak, hotspot)	*4.85843*		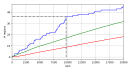	link	link	DHS & FAIRE
149	☐ hdpi__AKR1A1<br>Description: AKR1A1<br>Possible TFs: AKR1A1	*4.84659*	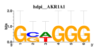	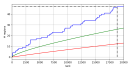	link	link	PWMs
150	☐ hocomoco__NR2C2_MOUSE.H11MO.0.A<br>Description: NR2C2_MOUSE<br>Possible TFs: NR2C2	*4.83087*	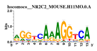	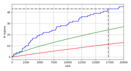	link	link	PWMs
151	☐ transfac_pro__M01036<br>Description: V$COUPTF_Q6: COUPTF<br>Possible TFs: NR2F1, NR2F2	*4.82918*	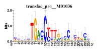	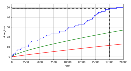	link	link	PWMs
152	☐ transfac_pro__M01859<br>Description: V$AP2GAMMA_Q5: AP-2 gamma<br>Possible TFs: TFAP2C	*4.81032*	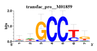	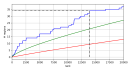	link	link	PWMs
153	☐ cisbp__M0476<br>Description: M0476	*4.80081*	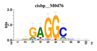	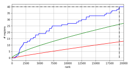	link	link	PWMs
154	☐ transfac_pro__M06617<br>Description: V$ZNF480_02: ZNF480<br>Possible TFs: ZNF480	*4.79848*	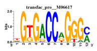	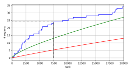	link	link	PWMs
155	☐ dbcorrdb__ARID3A__ENCSR000EDP_1__m1<br>Description: ARID3A (ENCSR000EDP-1, motif 1)<br>Possible TFs: ARID3A	*4.79517*	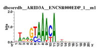	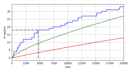	link	link	PWMs
156	☐ E085-DNase.hotspot.all.peaks-narrowpeak<br>Description: DNase in Fetal Intestine Small (E085, narrowpeak, hotspot)	*4.79404*		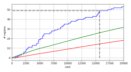	link	link	DHS & FAIRE
157	☐ transfac_pro__M03569<br>Description: V$VDR_Q6_01: VDR<br>Possible TFs: VDR	*4.77156*	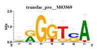	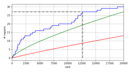	link	link	PWMs

#	Feature	NES	Logo	Recovery Curve	Candidate targets	All regions in top 20000	Database
158	☐ hocomoco__COT2_MOUSE.H11MO.1.B<br>Description: COT2_MOUSE<br>Possible TFs: NR2F2	*4.75302*	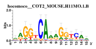	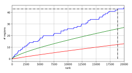	**link**	**link**	PWMs
159	☐ ENCFF001SWK<br>Description: H3K27ac ChIP-seq on human HepG2	*4.74718*		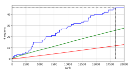	**link**	**link**	Histone modifications
160	☐ hocomoco__RORA_HUMAN.H11MO.0.C<br>Description: RORA_HUMAN<br>Possible TFs: RORA	*4.74545*	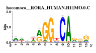	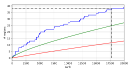	**link**	**link**	PWMs
161	☐ hocomoco__RARG_HUMAN.H11MO.1.B<br>Description: RARG_HUMAN<br>Possible TFs: RARG	*4.74343*	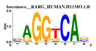	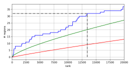	**link**	**link**	PWMs
162	☐ cisbp__M3414<br>Description: HNF4A[gene ID: "ENSG00000101076" species: "Homo sapiens" TF status: "direct" TF family: "Nuclear receptor" DBDs: "zf-C4"]; Hnf4g[gene ID: "ENSMUSG00000017688" species: "Mus musculus" TF status: "inferred" TF family: "Nuclear receptor" DBDs: "zf-C4"]<br>Possible TFs: HNF4A	*4.73303*	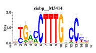	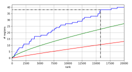	**link**	**link**	PWMs
163	☐ hocomoco__NR1H3_HUMAN.H11MO.1.B<br>Description: NR1H3_HUMAN<br>Possible TFs: NR1H3	*4.71426*	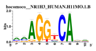	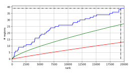	**link**	**link**	PWMs
164	☐ cisbp__M6025<br>Description: Hnf4a[gene ID: "ENSMUSG00000017950" species: "Mus musculus" TF status: "direct" TF family: "Nuclear receptor" DBDs: "zf-C4"]; Hnf4g[gene ID: "ENSMUSG00000017688" species: "Mus musculus" TF status: "inferred" TF family: "Nuclear receptor" DBDs: "zf-C4"]<br>Possible TFs: HNF4A	*4.70064*	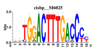	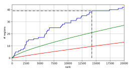	**link**	**link**	PWMs
165	☐ E057-H3K4me1<br>Description: H3K4me1 in Foreskin Keratinocyte Primary Cells skin02 (E057,)	*4.68026*		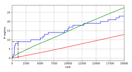	**link**	**link**	Histone modifications
166	☐ E066-H3K4me1<br>Description: H3K4me1 in Liver (E066,)	*4.67817*		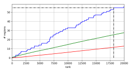	**link**	**link**	Histone modifications
167	☐ ENCFF001UHM<br>Description: SP1 ChIP-seq protocol PCR1x on human HepG2	*4.67358*		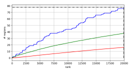	**link**	**link**	TF binding sites
168	☐ dbcorrdb__TCF12__ENCSR000BJG_1__m1<br>Description: TCF12 (ENCSR000BJG-1, motif 1)<br>Possible TFs: TCF12	*4.66187*	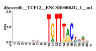	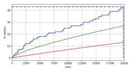	**link**	**link**	PWMs
169	☐ transfac_public__M00158<br>Description: V$COUP_01: COUP-TF, HNF-4<br>Possible TFs: NR2F1, HNF4A	*4.64414*	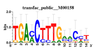	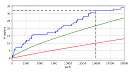	**link**	**link**	PWMs
170	☐ hocomoco__PPARG_HUMAN.H11MO.1.A<br>Description: PPARG_HUMAN<br>Possible TFs: PPARG	*4.63100*	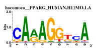	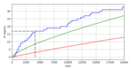	**link**	**link**	PWMs
171	☐ cisbp__M0403<br>Description: Zfp711[gene ID: "ENSMUSG00000025529" species: "Mus musculus" TF status: "direct" TF family: "C2H2 ZF" DBDs: "zf-C2H2"]<br>Possible TFs: ZNF711	*4.61585*	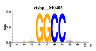	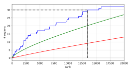	**link**	**link**	PWMs
172	☐ taipale_cyt_meth__HNF4A_NRGTCCAAAGGTCRN_eDBD<br>Description: HNF4A [Nuclear_receptor]<br>Possible TFs: HNF4A	*4.61424*	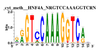	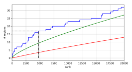	**link**	**link**	PWMs
173	☐ E048-H3K4me1<br>Description: H3K4me1 in Primary T CD8+ memory cells from peripheral blood (E048,)	*4.59914*		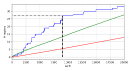	**link**	**link**	Histone modifications
174	☐ hdpi__TAGLN2<br>Description: TAGLN2<br>Possible TFs: TAGLN2	*4.59240*	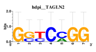	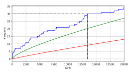	**link**	**link**	PWMs

#	Feature	NES	Logo	Recovery Curve	Candidate targets	All regions in top 20000	Database
175	☐ cisbp__M6179<br>Description: NR2F2[gene ID: "ENSG00000185551" species: "Homo sapiens" TF status: "direct" TF family: "Nuclear receptor" DBDs: "zf-C4"]<br>Possible TFs: NR2F2	*4.58563*	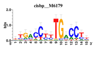	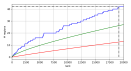	link	link	PWMs
176	☐ dbcorrdb__FOXA1__ENCSR000BMO_1__m1<br>Description: FOXA1 (ENCSR000BMO-1, motif 1)<br>Possible TFs: FOXA1	*4.56887*	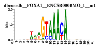	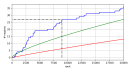	link	link	PWMs
177	☐ hdpi__AFF4<br>Description: AFF4<br>Possible TFs: AFF4	*4.56419*	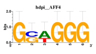	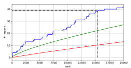	link	link	PWMs
178	☐ hocomoco__NR1H4_HUMAN.H11MO.1.B<br>Description: NR1H4_HUMAN<br>Possible TFs: NR1H4	*4.56323*	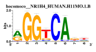	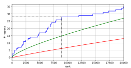	link	link	PWMs
179	☐ dbcorrdb__FOXA1__ENCSR000BKW_1__m1<br>Description: FOXA1 (ENCSR000BKW-1, motif 1)<br>Possible TFs: FOXA1	*4.56000*	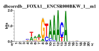	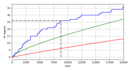	link	link	PWMs
180	☐ transfac_pro__M07961<br>Description: I$HNF4_04: Hnf-4<br>Possible TFs: HNF4G, HNF4A	*4.55444*	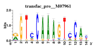	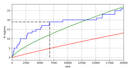	link	link	PWMs
181	☐ elemento__CCCCAGC<br>Description: Conserved regulatory element CCCCAGC between Hs and Mm	*4.53276*	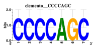	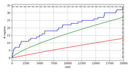	link	link	PWMs
182	☐ dbcorrdb__NFIC__ENCSR000BQX_1__m1<br>Description: NFIC (ENCSR000BQX-1, motif 1)<br>Possible TFs: NFIC	*4.53002*	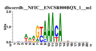	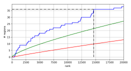	link	link	PWMs
183	☐ hocomoco__PPARG_HUMAN.H11MO.0.A<br>Description: PPARG_HUMAN<br>Possible TFs: PPARG	*4.52664*	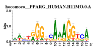	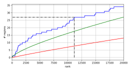	link	link	PWMs
184	☐ fantom__motif169_GCCTGGCC<br>Description: DENOVO_DMF_MOUSE: Motif229	*4.52430*	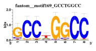	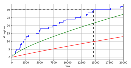	link	link	PWMs
185	☐ cisbp__M4689<br>Description: CG11152[gene ID: "FBgn0039937" species: "Drosophila melanogaster" TF status: "inferred" TF family: "Forkhead" DBDs: "Fork_head"]; CG9571[gene ID: "FBgn0031086" species: "Drosophila melanogaster" TF status: "inferred" TF family: "Forkhead" DBDs: "Fork_head"]; FOXA1[gene ID: "ENSG00000129514" species: "Homo sapiens" TF status: "direct" TF family: "Forkhead" DBDs: "Fork_head"]; FOXB2[gene ID: "ENSG00000204612" species: "Homo sapiens" TF status: "inferred" TF family: "Forkhead" DBDs: "Fork_head"]; FOXD4L1[gene ID: "ENSG00000184492" species: "Homo sapiens" TF status: "inferred" TF family: "Forkhead" DBDs: "Fork_head"]; FOXD4L3[gene ID: "ENSG00000187559" species: "Homo sapiens" TF status: "inferred" TF family: "Forkhead" DBDs: "Fork_head"]; FOXD4L5[gene ID: "ENSG00000204779" species: "Homo sapiens" TF status: "inferred" TF family: "Forkhead" DBDs: "Fork_head"]; FOXD4L6[gene ID: "ENSG00000204793" species: "Homo sapiens" TF status: "inferred" TF family: "Forkhead" DBDs: "Fork_head"]; FOXD4[gene ID: "ENSG00000170122" species: "Homo sapiens" TF status: "inferred" TF family: "Forkhead" DBDs: "Fork_head"]; FOXE1[gene ID: "ENSG00000178919" species: "Homo sapiens" TF status: "inferred" TF family: "Forkhead" DBDs: "Fork_head"]; FOXE3[gene ID: "ENSG00000186790" species: "Homo sapiens" TF status: "inferred" TF family: "Forkhead" DBDs: "Fork_head"]; FOXI2[gene ID: "ENSG00000186766" species: "Homo sapiens" TF status: "inferred" TF family: "Forkhead" DBDs: "Fork_head"]; FOXK2[gene ID: "ENSG00000141568" species: "Homo sapiens" TF status: "inferred" TF family: "Forkhead" DBDs: "Fork_head"]; FOXS1[gene ID: "ENSG00000179772" species: "Homo sapiens" TF status: "inferred" TF family: "Forkhead" DBDs: "Fork_head"]; Foxa1[gene ID: "ENSMUSG00000035451" species: "Mus musculus" TF status: "inferred" TF family: "Forkhead" DBDs: "Fork_head"]; Foxa3[gene ID: "ENSMUSG00000040891" species: "Mus musculus" TF status: "inferred" TF family: "Forkhead" DBDs: "Fork_head"]; Foxb2[gene ID: "ENSMUSG00000056829" species: "Mus musculus" TF status: "inferred" TF family: "Forkhead" DBDs: "Fork_head"]; Foxd2[gene ID: "ENSMUSG00000055210" species: "Mus musculus" TF status: "inferred" TF	*4.50923*	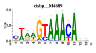	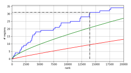	link	link	PWMs

#	Feature	NES	Logo	Recovery Curve	Candidate targets	All regions in top 20000	Database
	family: "Forkhead" DBDs: "Fork_head"]; Foxd3[gene ID: "ENSMUSG00000067261" species: "Mus musculus" TF status: "inferred" TF family: "Forkhead" DBDs: "Fork_head"]; Foxd4[gene ID: "ENSMUSG00000051490" species: "Mus musculus" TF status: "inferred" TF family: "Forkhead" DBDs: "Fork_head"]; Foxe1[gene ID: "ENSMUSG00000070990" species: "Mus musculus" TF status: "inferred" TF family: "Forkhead" DBDs: "Fork_head"]; Foxe3[gene ID: "ENSMUSG00000044518" species: "Mus musculus" TF status: "inferred" TF family: "Forkhead" DBDs: "Fork_head"]; Foxf1a[gene ID: "ENSMUSG00000042812" species: "Mus musculus" TF status: "inferred" TF family: "Forkhead" DBDs: "Fork_head"]; Foxf2[gene ID: "ENSMUSG00000038402" species: "Mus musculus" TF status: "inferred" TF family: "Forkhead" DBDs: "Fork_head"]; Foxi1[gene ID: "ENSMUSG00000047861" species: "Mus musculus" TF status: "inferred" TF family: "Forkhead" DBDs: "Fork_head"]; Foxi2[gene ID: "ENSMUSG00000048377" species: "Mus musculus" TF status: "inferred" TF family: "Forkhead" DBDs: "Fork_head"]; Foxi3[gene ID: "ENSMUSG00000055874" species: "Mus musculus" TF status: "inferred" TF family: "Forkhead" DBDs: "Fork_head"]; Foxk2[gene ID: "ENSMUSG00000039275" species: "Mus musculus" TF status: "inferred" TF family: "Forkhead" DBDs: "Fork_head"]; Foxl2[gene ID: "ENSMUSG00000050397" species: "Mus musculus" TF status: "inferred" TF family: "Forkhead" DBDs: "Fork_head"]; Foxq1[gene ID: "ENSMUSG00000038415" species: "Mus musculus" TF status: "inferred" TF family: "Forkhead" DBDs: "Fork_head"]; Foxs1[gene ID: "ENSMUSG00000074676" species: "Mus musculus" TF status: "inferred" TF family: "Forkhead" DBDs: "Fork_head"]; KIAA0415[gene ID: "ENSG00000242802" species: "Homo sapiens" TF status: "inferred" TF family: "Forkhead" DBDs: "Fork_head"]; Mnf[gene ID: "FBgn0036134" species: "Drosophila melanogaster" TF status: "inferred" TF family: "Forkhead" DBDs: "Fork_head"]; fd59A[gene ID: "FBgn0004896" species: "Drosophila melanogaster" TF status: "inferred" TF family: "Forkhead" DBDs: "Fork_head"]; fd96Ca[gene ID: "FBgn0004897" species: "Drosophila melanogaster" TF status: "inferred" TF family: "Forkhead" DBDs: "Fork_head"]; fd96Cb[gene ID: "FBgn0004898" species: "Drosophila melanogaster" TF status: "inferred" TF family: "Forkhead" DBDs: "Fork_head"]<br>Possible TFs: FOXA1						
186	☐ taipale_cyt_meth__HNF4A_NRGTCCAAAGGTCRN_eDBD_meth_repr<br>Description: HNF4A [Nuclear_receptor, CpG-meth]<br>Possible TFs: HNF4A	*4.50867*	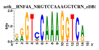	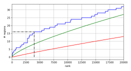	link	link	PWMs
187	☐ hocomoco__COT1_HUMAN.H11MO.0.C<br>Description: COT1_HUMAN<br>Possible TFs: NR2F1	*4.50810*	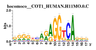	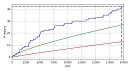	link	link	PWMs
188	☐ hocomoco__FOXA2_HUMAN.H11MO.0.A<br>Description: FOXA2_HUMAN<br>Possible TFs: FOXA2	*4.49754*	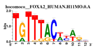	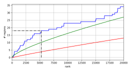	link	link	PWMs
189	☐ dbcorrdb__GATA3__ENCSR000BMX_1__m1<br>Description: GATA3 (ENCSR000BMX-1, motif 1)<br>Possible TFs: GATA3	*4.47917*	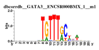	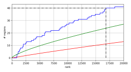	link	link	PWMs
190	☐ cisbp__M4439<br>Description: CG11152[gene ID: "FBgn0039937" species: "Drosophila melanogaster" TF status: "inferred" TF family: "Forkhead" DBDs: "Fork_head"]; CG9571[gene ID: "FBgn0031086" species: "Drosophila melanogaster" TF status: "inferred" TF family: "Forkhead" DBDs: "Fork_head"]; FOXA1[gene ID: "ENSG00000129514" species: "Homo sapiens" TF status: "direct" TF family: "Forkhead" DBDs: "Fork_head"]; FOXB2[gene ID: "ENSG00000204612" species: "Homo sapiens" TF status: "inferred" TF family: "Forkhead" DBDs: "Fork_head"]; FOXD4L1[gene ID: "ENSG00000184492" species: "Homo sapiens" TF status: "inferred" TF family: "Forkhead" DBDs: "Fork_head"]; FOXD4L3[gene ID: "ENSG00000187559" species: "Homo sapiens" TF status: "inferred" TF family: "Forkhead" DBDs: "Fork_head"]; FOXD4L5[gene ID: "ENSG00000204779" species: "Homo sapiens" TF status: "inferred" TF family: "Forkhead" DBDs: "Fork_head"]; FOXD4L6[gene ID: "ENSG00000204793" species: "Homo sapiens" TF status: "inferred" TF family: "Forkhead" DBDs: "Fork_head"]; FOXD4[gene ID: "ENSG00000170122" species: "Homo sapiens"	*4.47135*	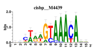	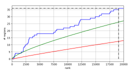	link	link	PWMs

#	Feature	NES	Logo	Recovery Curve	Candidate targets	All regions in top 20000	Database
	TF status: "inferred" TF family: "Forkhead" DBDs: "Fork_head"]; FOXE1[gene ID: "ENSG00000178919" species: "Homo sapiens" TF status: "inferred" TF family: "Forkhead" DBDs: "Fork_head"]; FOXE3[gene ID: "ENSG00000186790" species: "Homo sapiens" TF status: "inferred" TF family: "Forkhead" DBDs: "Fork_head"]; FOXI2[gene ID: "ENSG00000186766" species: "Homo sapiens" TF status: "inferred" TF family: "Forkhead" DBDs: "Fork_head"]; FOXK2[gene ID: "ENSG00000141568" species: "Homo sapiens" TF status: "inferred" TF family: "Forkhead" DBDs: "Fork_head"]; FOXS1[gene ID: "ENSG00000179772" species: "Homo sapiens" TF status: "inferred" TF family: "Forkhead" DBDs: "Fork_head"]; Foxa1[gene ID: "ENSMUSG00000035451" species: "Mus musculus" TF status: "inferred" TF family: "Forkhead" DBDs: "Fork_head"]; Foxa3[gene ID: "ENSMUSG00000040891" species: "Mus musculus" TF status: "inferred" TF family: "Forkhead" DBDs: "Fork_head"]; Foxb2[gene ID: "ENSMUSG00000056829" species: "Mus musculus" TF status: "inferred" TF family: "Forkhead" DBDs: "Fork_head"]; Foxd2[gene ID: "ENSMUSG00000055210" species: "Mus musculus" TF status: "inferred" TF family: "Forkhead" DBDs: "Fork_head"]; Foxd3[gene ID: "ENSMUSG00000067261" species: "Mus musculus" TF status: "inferred" TF family: "Forkhead" DBDs: "Fork_head"]; Foxd4[gene ID: "ENSMUSG00000051490" species: "Mus musculus" TF status: "inferred" TF family: "Forkhead" DBDs: "Fork_head"]; Foxe1[gene ID: "ENSMUSG00000070990" species: "Mus musculus" TF status: "inferred" TF family: "Forkhead" DBDs: "Fork_head"]; Foxe3[gene ID: "ENSMUSG00000044518" species: "Mus musculus" TF status: "inferred" TF family: "Forkhead" DBDs: "Fork_head"]; Foxf1a[gene ID: "ENSMUSG00000042812" species: "Mus musculus" TF status: "inferred" TF family: "Forkhead" DBDs: "Fork_head"]; Foxf2[gene ID: "ENSMUSG00000038402" species: "Mus musculus" TF status: "inferred" TF family: "Forkhead" DBDs: "Fork_head"]; Foxi1[gene ID: "ENSMUSG00000047861" species: "Mus musculus" TF status: "inferred" TF family: "Forkhead" DBDs: "Fork_head"]; Foxi2[gene ID: "ENSMUSG00000048377" species: "Mus musculus" TF status: "inferred" TF family: "Forkhead" DBDs: "Fork_head"]; Foxi3[gene ID: "ENSMUSG00000055874" species: "Mus musculus" TF status: "inferred" TF family: "Forkhead" DBDs: "Fork_head"]; Foxk2[gene ID: "ENSMUSG00000039275" species: "Mus musculus" TF status: "inferred" TF family: "Forkhead" DBDs: "Fork_head"]; Foxl2[gene ID: "ENSMUSG00000050397" species: "Mus musculus" TF status: "inferred" TF family: "Forkhead" DBDs: "Fork_head"]; Foxq1[gene ID: "ENSMUSG00000038415" species: "Mus musculus" TF status: "inferred" TF family: "Forkhead" DBDs: "Fork_head"]; Foxs1[gene ID: "ENSMUSG00000074676" species: "Mus musculus" TF status: "inferred" TF family: "Forkhead" DBDs: "Fork_head"]; KIAA0415[gene ID: "ENSG00000242802" species: "Homo sapiens" TF status: "inferred" TF family: "Forkhead" DBDs: "Fork_head"]; Mnf[gene ID: "FBgn0036134" species: "Drosophila melanogaster" TF status: "inferred" TF family: "Forkhead" DBDs: "Fork_head"]; fd59A[gene ID: "FBgn0004896" species: "Drosophila melanogaster" TF status: "inferred" TF family: "Forkhead" DBDs: "Fork_head"]; fd96Ca[gene ID: "FBgn0004897" species: "Drosophila melanogaster" TF status: "inferred" TF family: "Forkhead" DBDs: "Fork_head"]; fd96Cb[gene ID: "FBgn0004898" species: "Drosophila melanogaster" TF status: "inferred" TF family: "Forkhead" DBDs: "Fork_head"]<br>Possible TFs: FOXA1						
191	☐ E084-H3K4me1<br>Description: H3K4me1 in Fetal Intestine Large (E084,)	*4.47125*		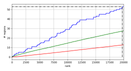	**link**	**link**	Histone modifications
192	☐ elemento__CCAGGCC<br>Description: Conserved regulatory element CCAGGCC between Hs and Mm	*4.46764*	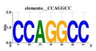	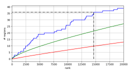	**link**	**link**	PWMs
193	☐ transfac_pro__M04854<br>Description: V$SMC3_03: SMC-3<br>Possible TFs: SMC3	*4.46547*	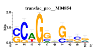	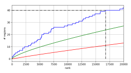	**link**	**link**	PWMs

#	Feature	NES	Logo	Recovery Curve	Candidate targets	All regions in top 20000	Database
194	☐ cisbp__M1851<br>Description: NR2F1[gene ID: "ENSG00000175745" species: "Homo sapiens" TF status: "direct" TF family: "Nuclear receptor" DBDs: "zf-C4"]<br>Possible TFs: NR2F1	*4.43476*	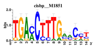	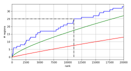	**link**	**link**	PWMs
195	☐ elemento__CCCAGCC<br>Description: Conserved regulatory element CCCAGCC between Hs and Mm	*4.41808*	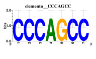	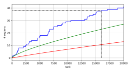	**link**	**link**	PWMs
196	☐ ENCFF001WTM<br>Description: DNase-seq on human Th1 (Wb54553204)	*4.41626*		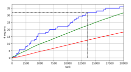	**link**	**link**	DHS & FAIRE
197	☐ E118-H3K4me2<br>Description: H3K4me2 in HepG2 Hepatocellular Carcinoma Cell Line (E118,)	*4.40914*		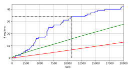	**link**	**link**	Histone modifications
198	☐ transfac_pro__M02119<br>Description: V$T3RBETA_Q6_01: T3R-beta<br>Possible TFs: THRB	*4.40785*	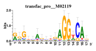	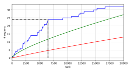	**link**	**link**	PWMs
199	☐ dbcorrdb__EP300__ENCSR000BLW_1__m1<br>Description: EP300 (ENCSR000BLW-1, motif 1)<br>Possible TFs: EP300	*4.37996*	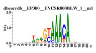	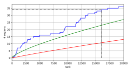	**link**	**link**	PWMs
200	☐ tfdimers__MD00020<br>Description: M00773_forward_9_M00377_forward dimer: MYB / Pax-4<br>Possible TFs: PAX4, MYB	*4.36441*	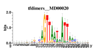	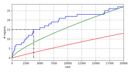	**link**	**link**	PWMs
201	☐ dbcorrdb__EP300__ENCSR000EDV_1__m2<br>Description: EP300 (ENCSR000EDV-1, motif 2)<br>Possible TFs: EP300	*4.36159*	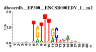	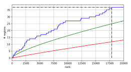	**link**	**link**	PWMs
202	☐ hocomoco__PPARD_HUMAN.H11MO.0.D<br>Description: PPARD_HUMAN<br>Possible TFs: PPARD	*4.35490*	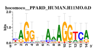	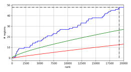	**link**	**link**	PWMs
203	☐ transfac_pro__M04924<br>Description: V$EBF1_05: COE1<br>Possible TFs: EBF1	*4.34402*	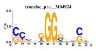	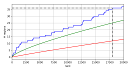	**link**	**link**	PWMs
204	☐ neph__UW.Motif.0023<br>Description: cagNNcccag	*4.33241*	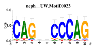	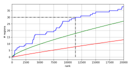	**link**	**link**	PWMs
205	☐ factorbook__FOXA<br>Description: FOXA<br>Possible TFs: FOXA1, FOXA2	*4.33128*	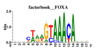	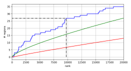	**link**	**link**	PWMs
206	☐ cisbp__M1434<br>Description: NR2F6[gene ID: "ENSG00000160113" species: "Homo sapiens" TF status: "direct" TF family: "Nuclear receptor" DBDs: "zf-C4"]<br>Possible TFs: NR2F6	*4.33040*	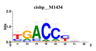	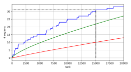	**link**	**link**	PWMs
207	☐ tfdimers__MD00002<br>Description: M01712_forward_5_M01712_reverse dimer: CRX / CRX<br>Possible TFs: CRX	*4.32395*	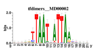	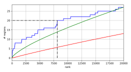	**link**	**link**	PWMs
208	☐ transfac_pro__M04891<br>Description: V$NANOG_06: nanog<br>Possible TFs: NANOG	*4.31444*	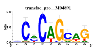	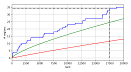	**link**	**link**	PWMs
209	☐ transfac_pro__M04904<br>Description: V$POLR3A_01: POLR3A<br>Possible TFs: POLR3A	*4.31202*	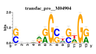	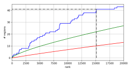	**link**	**link**	PWMs
210	☐ ENCFF001WTF<br>Description: DNase-seq on human Th1	*4.30587*		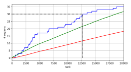	**link**	**link**	DHS & FAIRE
211	☐ ENCFF001WTI<br>Description: DNase-seq on human Th1 (Wb33676984)	*4.29994*		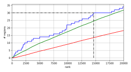	**link**	**link**	DHS & FAIRE
212	☐ tfdimers__MD00076<br>Description: M00790_forward_10_M01270_reverse dimer: HNF1 / PPARG<br>Possible TFs: HNF1B, PPARG, HNF1A	*4.27358*	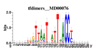	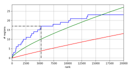	**link**	**link**	PWMs

#	Feature	NES	Logo	Recovery Curve	Candidate targets	All regions in top 20000	Database
213	☐ E030-H3K4me1<br>Description: H3K4me1 in Primary neutrophils from peripheral blood (E030,)	4.26956		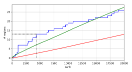	link	link	Histone modifications
214	☐ transfac_pro__M07280<br>Description: V$NR1NR2_Q3: RXR, LXR, PXR, CAR, RAR, COUP<br>Possible TFs: RARA, RARB, RARG, NR2F1, RXRA, RXRB, NR2F2, NR1I3, NR1I2, NR1H2, NR1H3	4.25843	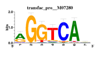	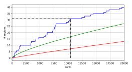	link	link	PWMs
215	☐ elemento__CTGGCCC<br>Description: Conserved regulatory element CTGGCCC between Hs and Mm	4.24441	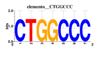	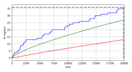	link	link	PWMs
216	☐ hdpi__SCC-112<br>Description: SCC-112<br>Possible TFs: PDS5A	4.23014	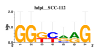	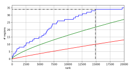	link	link	PWMs
217	☐ E101-H3K4me1<br>Description: H3K4me1 in Rectal Mucosa Donor 29 (E101,)	4.22853		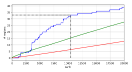	link	link	Histone modifications
218	☐ taipale__HNF4A_full_RRGGTCAAAGGTCA_repr<br>Description: HNF4A dimeric nuclearreceptor full<br>Possible TFs: HNF4A	4.22756	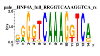	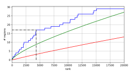	link	link	PWMs
219	☐ dbcorrdb__FOXA1__ENCSR000BKY_1__m1<br>Description: FOXA1 (ENCSR000BKY-1, motif 1)<br>Possible TFs: FOXA1	4.20830	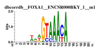	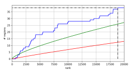	link	link	PWMs
220	☐ E118-H3K4me2-broadpeak<br>Description: H3K4me2 in HepG2 Hepatocellular Carcinoma Cell Line (E118, broadpeak)	4.19971		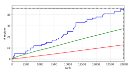	link	link	Histone modifications
221	☐ cisbp__M6219<br>Description: ESR2[gene ID: "ENSG00000140009" species: "Homo sapiens" TF status: "direct" TF family: "Nuclear receptor" DBDs: "zf-C4"]; Esr2[gene ID: "ENSMUSG00000021055" species: "Mus musculus" TF status: "inferred" TF family: "Nuclear receptor" DBDs: "zf-C4"]<br>Possible TFs: ESR2	4.19718	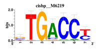	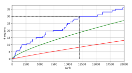	link	link	PWMs
222	☐ E114-H3K4me1-broadpeak<br>Description: H3K4me1 in A549 EtOH 0.02pct Lung Carcinoma Cell Line (E114, broadpeak)	4.19147		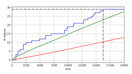	link	link	Histone modifications
223	☐ hocomoco__HAND1_HUMAN.H11MO.1.D<br>Description: HAND1_HUMAN<br>Possible TFs: HAND1	4.17526	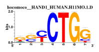	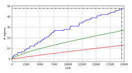	link	link	PWMs
224	☐ transfac_pro__M05547<br>Description: V$ZAC_04: ZAC<br>Possible TFs: PLAGL1	4.17437	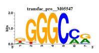	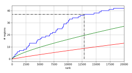	link	link	PWMs
225	☐ cisbp__M2311<br>Description: AL662824.5[gene ID: "ENSG00000227322" species: "Homo sapiens" TF status: "inferred" TF family: "Nuclear receptor" DBDs: "zf-C4"]; AL844527.7[gene ID: "ENSG00000206289" species: "Homo sapiens" TF status: "inferred" TF family: "Nuclear receptor" DBDs: "zf-C4"]; CR354565.3[gene ID: "ENSG00000231321" species: "Homo sapiens" TF status: "inferred" TF family: "Nuclear receptor" DBDs: "zf-C4"]; CR759733.4[gene ID: "ENSG00000228333" species: "Homo sapiens" TF status: "inferred" TF family: "Nuclear receptor" DBDs: "zf-C4"]; CR936877.3[gene ID: "ENSG00000235712" species: "Homo sapiens" TF status: "inferred" TF family: "Nuclear receptor" DBDs: "zf-C4"]; Rxra[gene ID: "ENSMUSG00000015846" species: "Mus musculus" TF status: "direct" TF family: "Nuclear receptor" DBDs: "zf-C4"]<br>Possible TFs: RXRA	4.17405	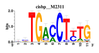	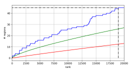	link	link	PWMs
226	☐ cisbp__M5529<br>Description: HNF4A[gene ID: "ENSG00000101076" species: "Homo sapiens" TF status: "direct" TF family: "Nuclear receptor" DBDs: "zf-C4"]; Hnf4g[gene ID: "ENSMUSG00000017688" species: "Mus musculus" TF status: "inferred" TF family: "Nuclear receptor" DBDs: "zf-C4"]<br>Possible TFs: HNF4A	4.17276	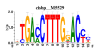	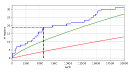	link	link	PWMs
227	☐ neph__UW.Motif.0021<br>Description: cagcctcc	4.17203	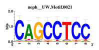	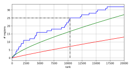	link	link	PWMs

#	Feature	NES	Logo	Recovery Curve	Candidate targets	All regions in top 20000	Database
228	☐ ENCFF001UFL<br>Description: CEBPD ChIP-seq protocol v041610.1 on human HepG2	*4.17075*		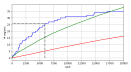	**link**	**link**	TF binding sites
229	☐ cisbp__M1452<br>Description: Rora[gene ID: "ENSMUSG00000032238" species: "Mus musculus" TF status: "direct" TF family: "Nuclear receptor" DBDs: "zf-C4"]; Rorc[gene ID: "ENSMUSG00000028150" species: "Mus musculus" TF status: "inferred" TF family: "Nuclear receptor" DBDs: "zf-C4"]<br>Possible TFs: RORA	*4.16744*	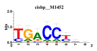	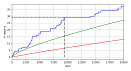	**link**	**link**	PWMs
230	☐ ENCFF001UGR<br>Description: MYBL2 ChIP-seq protocol v042211.1 on human HepG2	*4.15346*		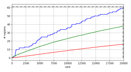	**link**	**link**	TF binding sites
231	☐ dbcorrdb__FOXA1__ENCSR000BLE_1__m1<br>Description: FOXA1 (ENCSR000BLE-1, motif 1)<br>Possible TFs: FOXA1	*4.13778*	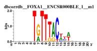	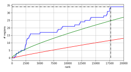	**link**	**link**	PWMs
232	☐ E098-H3K4me1<br>Description: H3K4me1 in Pancreas (E098,)	*4.13593*		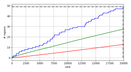	**link**	**link**	Histone modifications
233	☐ ENCFF001WTC<br>Description: UW human Th17 DNase-seq	*4.13282*		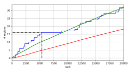	**link**	**link**	DHS & FAIRE
234	☐ E029-H3K27ac<br>Description: H3K27ac in Primary monocytes from peripheral blood (E029,)	*4.11463*		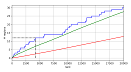	**link**	**link**	Histone modifications
235	☐ hdpi__TCEAL6<br>Description: TCEAL6<br>Possible TFs: TCEAL6	*4.10579*	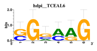	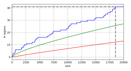	**link**	**link**	PWMs
236	☐ homer__CAAAGGTCAG_Erra<br>Description: Erra(NR)/HepG2-Erra-ChIP-Seq(GSE31477)/Homer<br>Possible TFs: ESRRA	*4.10506*	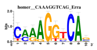	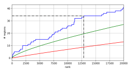	**link**	**link**	PWMs
237	☐ hocomoco__ESR2_MOUSE.H11MO.1.A<br>Description: ESR2_MOUSE<br>Possible TFs: ESR2	*4.09942*	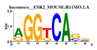	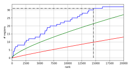	**link**	**link**	PWMs
238	☐ ENCFF001UFX<br>Description: FOXA1 ChIP-seq protocol v041610.1 on human HepG2	*4.09717*		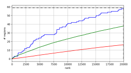	**link**	**link**	TF binding sites
239	☐ transfac_pro__M07289<br>Description: V$GKLF_Q3_01: GKLF<br>Possible TFs: KLF4	*4.08765*	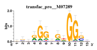	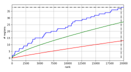	**link**	**link**	PWMs
240	☐ cisbp__M4567<br>Description: CG11152[gene ID: "FBgn0039937" species: "Drosophila melanogaster" TF status: "inferred" TF family: "Forkhead" DBDs: "Fork_head"]; CG9571[gene ID: "FBgn0031086" species: "Drosophila melanogaster" TF status: "inferred" TF family: "Forkhead" DBDs: "Fork_head"]; FOXA2[gene ID: "ENSG00000125798" species: "Homo sapiens" TF status: "direct" TF family: "Forkhead" DBDs: "Fork_head"]; FOXB2[gene ID: "ENSG00000204612" species: "Homo sapiens" TF status: "inferred" TF family: "Forkhead" DBDs: "Fork_head"]; FOXD4L1[gene ID: "ENSG00000184492" species: "Homo sapiens" TF status: "inferred" TF family: "Forkhead" DBDs: "Fork_head"]; FOXD4L3[gene ID: "ENSG00000187559" species: "Homo sapiens" TF status: "inferred" TF family: "Forkhead" DBDs: "Fork_head"]; FOXD4L5[gene ID: "ENSG00000204779" species: "Homo sapiens" TF status: "inferred" TF family: "Forkhead" DBDs: "Fork_head"]; FOXD4L6[gene ID: "ENSG00000204793" species: "Homo sapiens" TF status: "inferred" TF family: "Forkhead" DBDs: "Fork_head"]; FOXD4[gene ID: "ENSG00000170122" species: "Homo sapiens" TF status: "inferred" TF family: "Forkhead" DBDs: "Fork_head"]; FOXE1[gene ID: "ENSG00000178919" species: "Homo sapiens" TF status: "inferred" TF family: "Forkhead" DBDs: "Fork_head"]; FOXE3[gene ID: "ENSG00000186790" species: "Homo sapiens" TF status: "inferred" TF family: "Forkhead" DBDs: "Fork_head"]; FOXI2[gene ID: "ENSG00000186766" species: "Homo sapiens" TF status: "inferred" TF family: "Forkhead" DBDs: "Fork_head"]; FOXK2[gene ID: "ENSG00000141568" species: "Homo sapiens" TF status: "inferred" TF family: "Forkhead" DBDs: "Fork_head"]; FOXS1[gene ID: "ENSG00000179772" species:	*4.08717*	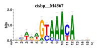	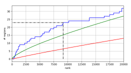	**link**	**link**	PWMs

#	Feature	NES	Logo	Recovery Curve	Candidate targets	All regions in top 20000	Database
	"Homo sapiens" TF status: "inferred" TF family: "Forkhead" DBDs: "Fork_head"]; Foxa1[gene ID: "ENSMUSG00000035451" species: "Mus musculus" TF status: "inferred" TF family: "Forkhead" DBDs: "Fork_head"]; Foxa3[gene ID: "ENSMUSG00000040891" species: "Mus musculus" TF status: "inferred" TF family: "Forkhead" DBDs: "Fork_head"]; Foxb2[gene ID: "ENSMUSG00000056829" species: "Mus musculus" TF status: "inferred" TF family: "Forkhead" DBDs: "Fork_head"]; Foxd2[gene ID: "ENSMUSG00000055210" species: "Mus musculus" TF status: "inferred" TF family: "Forkhead" DBDs: "Fork_head"]; Foxd3[gene ID: "ENSMUSG00000067261" species: "Mus musculus" TF status: "inferred" TF family: "Forkhead" DBDs: "Fork_head"]; Foxd4[gene ID: "ENSMUSG00000051490" species: "Mus musculus" TF status: "inferred" TF family: "Forkhead" DBDs: "Fork_head"]; Foxe1[gene ID: "ENSMUSG00000070990" species: "Mus musculus" TF status: "inferred" TF family: "Forkhead" DBDs: "Fork_head"]; Foxe3[gene ID: "ENSMUSG00000044518" species: "Mus musculus" TF status: "inferred" TF family: "Forkhead" DBDs: "Fork_head"]; Foxf1a[gene ID: "ENSMUSG00000042812" species: "Mus musculus" TF status: "inferred" TF family: "Forkhead" DBDs: "Fork_head"]; Foxf2[gene ID: "ENSMUSG00000038402" species: "Mus musculus" TF status: "inferred" TF family: "Forkhead" DBDs: "Fork_head"]; Foxi1[gene ID: "ENSMUSG00000047861" species: "Mus musculus" TF status: "inferred" TF family: "Forkhead" DBDs: "Fork_head"]; Foxi2[gene ID: "ENSMUSG00000048377" species: "Mus musculus" TF status: "inferred" TF family: "Forkhead" DBDs: "Fork_head"]; Foxi3[gene ID: "ENSMUSG00000055874" species: "Mus musculus" TF status: "inferred" TF family: "Forkhead" DBDs: "Fork_head"]; Foxk2[gene ID: "ENSMUSG00000039275" species: "Mus musculus" TF status: "inferred" TF family: "Forkhead" DBDs: "Fork_head"]; Foxl2[gene ID: "ENSMUSG00000050397" species: "Mus musculus" TF status: "inferred" TF family: "Forkhead" DBDs: "Fork_head"]; Foxq1[gene ID: "ENSMUSG00000038415" species: "Mus musculus" TF status: "inferred" TF family: "Forkhead" DBDs: "Fork_head"]; Foxs1[gene ID: "ENSMUSG00000074676" species: "Mus musculus" TF status: "inferred" TF family: "Forkhead" DBDs: "Fork_head"]; KIAA0415[gene ID: "ENSG00000242802" species: "Homo sapiens" TF status: "inferred" TF family: "Forkhead" DBDs: "Fork_head"]; Mnf[gene ID: "FBgn0036134" species: "Drosophila melanogaster" TF status: "inferred" TF family: "Forkhead" DBDs: "Fork_head"]; fd59A[gene ID: "FBgn0004896" species: "Drosophila melanogaster" TF status: "inferred" TF family: "Forkhead" DBDs: "Fork_head"]; fd96Ca[gene ID: "FBgn0004897" species: "Drosophila melanogaster" TF status: "inferred" TF family: "Forkhead" DBDs: "Fork_head"]; fd96Cb[gene ID: "FBgn0004898" species: "Drosophila melanogaster" TF status: "inferred" TF family: "Forkhead" DBDs: "Fork_head"]<br>Possible TFs: FOXA2						
241	☐ flyfactorsurvey__fkh_NAR_FBgn0000659<br>Description: fkh_NAR_FBgn0000659<br>Possible TFs: FOXA1, FOXA3, FOXA2	*4.08177*	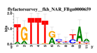	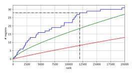	link	link	PWMs
242	☐ ENCFF001WCL<br>Description: UW human Th17 DNase-seq	*4.08018*		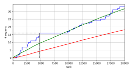	link	link	DHS & FAIRE
243	☐ cisbp__M6431<br>Description: PPARA[gene ID: "ENSG00000186951" species: "Homo sapiens" TF status: "direct" TF family: "Nuclear receptor" DBDs: "zf-C4"]; Ppara[gene ID: "ENSMUSG00000022383" species: "Mus musculus" TF status: "inferred" TF family: "Nuclear receptor" DBDs: "zf-C4"]; Ppard[gene ID: "ENSMUSG00000002250" species: "Mus musculus" TF status: "inferred" TF family: "Nuclear receptor" DBDs: "zf-C4"]; Pparg[gene ID: "ENSMUSG00000000440" species: "Mus musculus" TF status: "inferred" TF family: "Nuclear receptor" DBDs: "zf-C4"]<br>Possible TFs: PPARA	*4.06702*	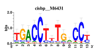	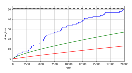	link	link	PWMs
244	☐ ENCFF001SOV<br>Description: DNase-seq on human Hepatocytes	*4.06280*		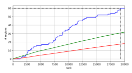	link	link	DHS & FAIRE

#	Feature	NES	Logo	Recovery Curve	Candidate targets	All regions in top 20000	Database
245	☐ cisbp__M3407<br>Description: HNF1A[gene ID: "ENSG00000135100" species: "Homo sapiens" TF status: "direct" TF family: "Homeodomain" DBDs: "Homeobox"]<br>Possible TFs: HNF1A	*4.06154*	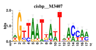	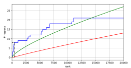	link	link	PWMs
246	☐ neph__UW.Motif.0600<br>Description: cNNccNNNNctgag	*4.03801*	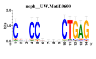	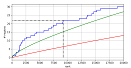	link	link	PWMs
247	☐ ENCFF001UHE<br>Description: POLR2A ChIP-seq protocol PCR2x on human HepG2	*4.02514*		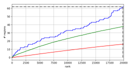	link	link	TF binding sites
248	☐ hdpi__SMPX<br>Description: SMPX<br>Possible TFs: SMPX	*4.01722*	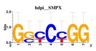	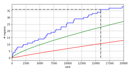	link	link	PWMs
249	☐ ENCFF001UGD<br>Description: HDAC2 ChIP-seq protocol v041610.1 on human HepG2	*4.01597*		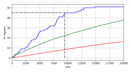	link	link	TF binding sites
250	☐ hocomoco__COT2_HUMAN.H11MO.1.A<br>Description: COT2_HUMAN<br>Possible TFs: NR2F2	*4.00642*	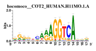	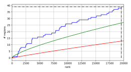	link	link	PWMs
251	☐ ENCFF001UHB<br>Description: EP300 ChIP-seq protocol v041610.1 on human HepG2	*4.00303*		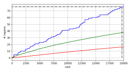	link	link	TF binding sites
252	☐ taipale__HNF4A_full_RRGTCCAAAGGTCAA<br>Description: HNF4A dimeric nuclearreceptor full<br>Possible TFs: HNF4A	*4.00271*	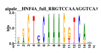	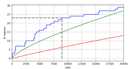	link	link	PWMs
253	☐ elemento__CCTGCCC<br>Description: Conserved regulatory element CCTGCCC between Hs and Mm	*3.99175*	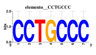	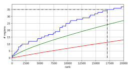	link	link	PWMs
254	☐ hocomoco__PPARA_HUMAN.H11MO.0.B<br>Description: PPARA_HUMAN<br>Possible TFs: PPARA	*3.98933*	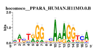	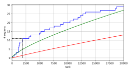	link	link	PWMs
255	☐ ENCFF001SPA<br>Description: DNase-seq on human Huh-7	*3.98553*		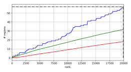	link	link	DHS & FAIRE
256	☐ E037-H3K4me1<br>Description: H3K4me1 in Primary T helper memory cells from peripheral blood 2 (E037,)	*3.98121*		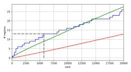	link	link	Histone modifications
257	☐ hdpi__FGF19<br>Description: FGF19<br>Possible TFs: FGF19	*3.98079*	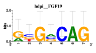	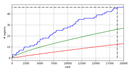	link	link	PWMs
258	☐ transfac_pro__M01270<br>Description: V$PPARG_Q6: PPARgamma<br>Possible TFs: PPARG	*3.97611*	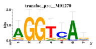	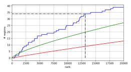	link	link	PWMs
259	☐ ENCFF001UGO<br>Description: MBD4 ChIP-seq protocol v042211.1 on human HepG2	*3.96963*		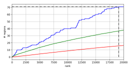	link	link	TF binding sites
260	☐ hdpi__CBX7<br>Description: CBX7<br>Possible TFs: CBX7	*3.95548*	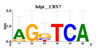	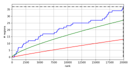	link	link	PWMs
261	☐ transfac_pro__M03547<br>Description: V$ERALPHA_Q6_01: ER-alpha<br>Possible TFs: ESR1	*3.95363*	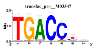	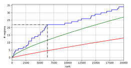	link	link	PWMs
262	☐ cisbp__M0468<br>Description: M0468	*3.94936*	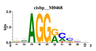	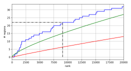	link	link	PWMs
263	☐ hocomoco__RXRA_HUMAN.H11MO.0.A<br>Description: RXRA_HUMAN<br>Possible TFs: RXRA	*3.94895*	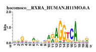	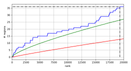	link	link	PWMs
264	☐ transfac_pro__M01725<br>Description: V$TR4_Q2: TR4<br>Possible TFs: NR2C2	*3.94638*	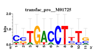	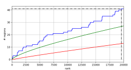	link	link	PWMs

265		*3.94428*	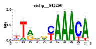	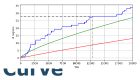	link	link	PWMs
**#**	**Feature**	**NES**	**Logo**	**Curve**	**Candidate targets**	**All regions in top 20000**	**Database**

☐ cisbp__M2250
Description: CG11152[gene ID: "FBgn0039937" species: "Drosophila melanogaster" TF status: "inferred" TF family: "Forkhead" DBDs: "Fork_head"]; CG9571[gene ID: "FBgn0031086" species: "Drosophila melanogaster" TF status: "inferred" TF family: "Forkhead" DBDs: "Fork_head"]; FOXB2[gene ID: "ENSG00000204612" species: "Homo sapiens" TF status: "inferred" TF family: "Forkhead" DBDs: "Fork_head"]; FOXD4L1[gene ID: "ENSG00000184492" species: "Homo sapiens" TF status: "inferred" TF family: "Forkhead" DBDs: "Fork_head"]; FOXD4L3[gene ID: "ENSG00000187559" species: "Homo sapiens" TF status: "inferred" TF family: "Forkhead" DBDs: "Fork_head"]; FOXD4L5[gene ID: "ENSG00000204779" species: "Homo sapiens" TF status: "inferred" TF family: "Forkhead" DBDs: "Fork_head"]; FOXD4L6[gene ID: "ENSG00000204793" species: "Homo sapiens" TF status: "inferred" TF family: "Forkhead" DBDs: "Fork_head"]; FOXD4[gene ID: "ENSG00000170122" species: "Homo sapiens" TF status: "inferred" TF family: "Forkhead" DBDs: "Fork_head"]; FOXE1[gene ID: "ENSG00000178919" species: "Homo sapiens" TF status: "inferred" TF family: "Forkhead" DBDs: "Fork_head"]; FOXE3[gene ID: "ENSG00000186790" species: "Homo sapiens" TF status: "inferred" TF family: "Forkhead" DBDs: "Fork_head"]; FOXI2[gene ID: "ENSG00000186766" species: "Homo sapiens" TF status: "inferred" TF family: "Forkhead" DBDs: "Fork_head"]; FOXK2[gene ID: "ENSG00000141568" species: "Homo sapiens" TF status: "inferred" TF family: "Forkhead" DBDs: "Fork_head"]; FOXS1[gene ID: "ENSG00000179772" species: "Homo sapiens" TF status: "inferred" TF family: "Forkhead" DBDs: "Fork_head"]; Foxa1[gene ID: "ENSMUSG00000035451" species: "Mus musculus" TF status: "inferred" TF family: "Forkhead" DBDs: "Fork_head"]; Foxa3[gene ID: "ENSMUSG00000040891" species: "Mus musculus" TF status: "inferred" TF family: "Forkhead" DBDs: "Fork_head"]; Foxb2[gene ID: "ENSMUSG00000056829" species: "Mus musculus" TF status: "inferred" TF family: "Forkhead" DBDs: "Fork_head"]; Foxd2[gene ID: "ENSMUSG00000055210" species: "Mus musculus" TF status: "inferred" TF family: "Forkhead" DBDs: "Fork_head"]; Foxd3[gene ID: "ENSMUSG00000067261" species: "Mus musculus" TF status: "inferred" TF family: "Forkhead" DBDs: "Fork_head"]; Foxd4[gene ID: "ENSMUSG00000051490" species: "Mus musculus" TF status: "inferred" TF family: "Forkhead" DBDs: "Fork_head"]; Foxe1[gene ID: "ENSMUSG00000070990" species: "Mus musculus" TF status: "inferred" TF family: "Forkhead" DBDs: "Fork_head"]; Foxe3[gene ID: "ENSMUSG00000044518" species: "Mus musculus" TF status: "inferred" TF family: "Forkhead" DBDs: "Fork_head"]; Foxf1a[gene ID: "ENSMUSG00000042812" species: "Mus musculus" TF status: "inferred" TF family: "Forkhead" DBDs: "Fork_head"]; Foxf2[gene ID: "ENSMUSG00000038402" species: "Mus musculus" TF status: "inferred" TF family: "Forkhead" DBDs: "Fork_head"]; Foxi1[gene ID: "ENSMUSG00000047861" species: "Mus musculus" TF status: "inferred" TF family: "Forkhead" DBDs: "Fork_head"]; Foxi2[gene ID: "ENSMUSG00000048377" species: "Mus musculus" TF status: "inferred" TF family: "Forkhead" DBDs: "Fork_head"]; Foxi3[gene ID: "ENSMUSG00000055874" species: "Mus musculus" TF status: "inferred" TF family: "Forkhead" DBDs: "Fork_head"]; Foxk2[gene ID: "ENSMUSG00000039275" species: "Mus musculus" TF status: "inferred" TF family: "Forkhead" DBDs: "Fork_head"]; Foxl2[gene ID: "ENSMUSG00000050397" species: "Mus musculus" TF status: "inferred" TF family: "Forkhead" DBDs: "Fork_head"]; Foxq1[gene ID: "ENSMUSG00000038415" species: "Mus musculus" TF status: "inferred" TF family: "Forkhead" DBDs: "Fork_head"]; Foxs1[gene ID: "ENSMUSG00000074676" species: "Mus musculus" TF status: "inferred" TF family: "Forkhead" DBDs: "Fork_head"]; KIAA0415[gene ID: "ENSG00000242802" species: "Homo sapiens" TF status: "inferred" TF family: "Forkhead" DBDs: "Fork_head"]; Mnf[gene ID: "FBgn0036134" species: "Drosophila melanogaster" TF status: "inferred" TF family: "Forkhead" DBDs: "Fork_head"]; fd59A[gene ID: "FBgn0004896" species: "Drosophila melanogaster" TF status: "inferred" TF family: "Forkhead" DBDs: "Fork_head"]; fd96Ca[gene ID: "FBgn0004897" species: "Drosophila melanogaster" TF status: "inferred" TF family: "Forkhead" DBDs: "Fork_head"]; fd96Cb[gene ID: "FBgn0004898" species: "Drosophila melanogaster" TF status: "inferred" TF

#	Feature	NES	Logo	Recovery Curve	Candidate targets	All regions in top 20000	Database
	family: "Forkhead" DBDs: "Fork_head"]; fkh[gene ID: "FBgn0000659" species: "Drosophila melanogaster" TF status: "direct" TF family: "Forkhead" DBDs: "Fork_head"]<br>Possible TFs: FOXA1, FOXA3, FOXA2						
266	☐ hocomoco__FOXA1_HUMAN.H11MO.0.A<br>Description: FOXA1_HUMAN<br>Possible TFs: FOXA1	*3.93799*	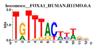	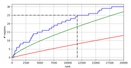	link	link	PWMs
267	☐ neph__UW.Motif.0576<br>Description: gNNNcctggNNcNNNc	*3.93558*	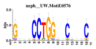	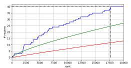	link	link	PWMs
268	☐ hdpi__NUP133<br>Description: NUP133<br>Possible TFs: NUP133	*3.93235*	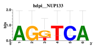	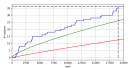	link	link	PWMs
269	☐ tfdimers__MD00333<br>Description: M01859_forward_7_M01721_forward dimer: AP-2 gamma / PUR1<br>Possible TFs: PURA, TFAP2C	*3.93139*	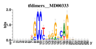	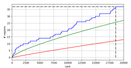	link	link	PWMs
270	☐ hocomoco__ZFX_HUMAN.H11MO.0.A<br>Description: ZFX_HUMAN<br>Possible TFs: ZFX	*3.91390*	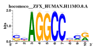	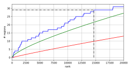	link	link	PWMs
271	☐ E084-H3K4me1-broadpeak<br>Description: H3K4me1 in Fetal Intestine Large (E084, broadpeak)	*3.91095*		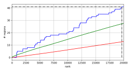	link	link	Histone modifications
272	☐ hdpi__ZNF503<br>Description: ZNF503<br>Possible TFs: ZNF503	*3.90640*	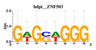	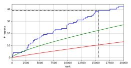	link	link	PWMs
273	☐ predrem__nrMotif120<br>Description: 10_CACO2-DS8235.M955	*3.85047*	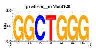	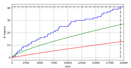	link	link	PWMs
274	☐ transfac_pro__M01282<br>Description: V$PPARA_Q6: PPARA<br>Possible TFs: PPARA	*3.85007*	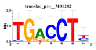	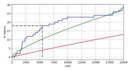	link	link	PWMs
275	☐ cisbp__M1542<br>Description: M1542	*3.84958*	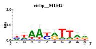	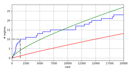	link	link	PWMs
276	☐ E110-H3K4me1<br>Description: H3K4me1 in Stomach Mucosa (E110,)	*3.83985*		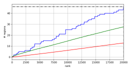	link	link	Histone modifications
277	☐ cisbp__M2303<br>Description: NR2C2[gene ID: "ENSG00000177463" species: "Homo sapiens" TF status: "direct" TF family: "Nuclear receptor" DBDs: "zf-C4"]<br>Possible TFs: NR2C2	*3.82799*	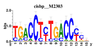	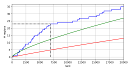	link	link	PWMs
278	☐ transfac_pro__M01822<br>Description: V$CPBP_Q6: CPBP<br>Possible TFs: KLF6	*3.82702*	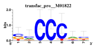	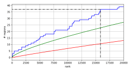	link	link	PWMs
279	☐ dbcorrdb__FOXA2__ENCSR000BNI_1__m1<br>Description: FOXA2 (ENCSR000BNI-1, motif 1)<br>Possible TFs: FOXA2	*3.81646*	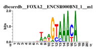	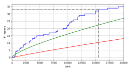	link	link	PWMs
280	☐ transfac_pro__M07125<br>Description: V$RXRA_11: RXR-alpha<br>Possible TFs: RXRA	*3.81525*	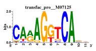	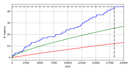	link	link	PWMs
281	☐ hdpi__HLCS<br>Description: HLCS<br>Possible TFs: HLCS	*3.81050*	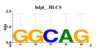	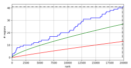	link	link	PWMs
282	☐ E077-H3K4me1<br>Description: H3K4me1 in Duodenum Mucosa (E077,)	*3.81010*		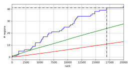	link	link	Histone modifications
283	☐ dbcorrdb__TCF12__ENCSR000BQQ_1__m1<br>Description: TCF12 (ENCSR000BQQ-1, motif 1)<br>Possible TFs: TCF12	*3.78285*	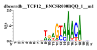	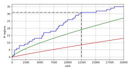	link	link	PWMs

#	Feature	NES	Logo	Recovery Curve	Candidate targets	All regions in top 20000	Database
284	☐ predrem__nrMotif688<br>Description: 132_fLung-DS14751.M20	*3.78076*	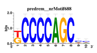	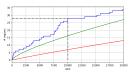	**link**	**link**	PWMs
285	☐ dbcorrdb__POLR2AphosphoS5__ENCSR000BQC_1__m1<br>Description: POLR2AphosphoS5 (ENCSR000BQC-1, motif 1)	*3.77238*	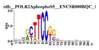	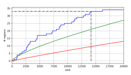	**link**	**link**	PWMs
286	☐ cisbp__M4664<br>Description: CG11152[gene ID: "FBgn0039937" species: "Drosophila melanogaster" TF status: "inferred" TF family: "Forkhead" DBDs: "Fork_head"]; CG9571[gene ID: "FBgn0031086" species: "Drosophila melanogaster" TF status: "inferred" TF family: "Forkhead" DBDs: "Fork_head"]; FOXA1[gene ID: "ENSG00000129514" species: "Homo sapiens" TF status: "direct" TF family: "Forkhead" DBDs: "Fork_head"]; FOXB2[gene ID: "ENSG00000204612" species: "Homo sapiens" TF status: "inferred" TF family: "Forkhead" DBDs: "Fork_head"]; FOXD4L1[gene ID: "ENSG00000184492" species: "Homo sapiens" TF status: "inferred" TF family: "Forkhead" DBDs: "Fork_head"]; FOXD4L3[gene ID: "ENSG00000187559" species: "Homo sapiens" TF status: "inferred" TF family: "Forkhead" DBDs: "Fork_head"]; FOXD4L5[gene ID: "ENSG00000204779" species: "Homo sapiens" TF status: "inferred" TF family: "Forkhead" DBDs: "Fork_head"]; FOXD4L6[gene ID: "ENSG00000204793" species: "Homo sapiens" TF status: "inferred" TF family: "Forkhead" DBDs: "Fork_head"]; FOXD4[gene ID: "ENSG00000170122" species: "Homo sapiens" TF status: "inferred" TF family: "Forkhead" DBDs: "Fork_head"]; FOXE1[gene ID: "ENSG00000178919" species: "Homo sapiens" TF status: "inferred" TF family: "Forkhead" DBDs: "Fork_head"]; FOXE3[gene ID: "ENSG00000186790" species: "Homo sapiens" TF status: "inferred" TF family: "Forkhead" DBDs: "Fork_head"]; FOXI2[gene ID: "ENSG00000186766" species: "Homo sapiens" TF status: "inferred" TF family: "Forkhead" DBDs: "Fork_head"]; FOXK2[gene ID: "ENSG00000141568" species: "Homo sapiens" TF status: "inferred" TF family: "Forkhead" DBDs: "Fork_head"]; FOXS1[gene ID: "ENSG00000179772" species: "Homo sapiens" TF status: "inferred" TF family: "Forkhead" DBDs: "Fork_head"]; Foxa1[gene ID: "ENSMUSG00000035451" species: "Mus musculus" TF status: "inferred" TF family: "Forkhead" DBDs: "Fork_head"]; Foxa3[gene ID: "ENSMUSG00000040891" species: "Mus musculus" TF status: "inferred" TF family: "Forkhead" DBDs: "Fork_head"]; Foxb2[gene ID: "ENSMUSG00000056829" species: "Mus musculus" TF status: "inferred" TF family: "Forkhead" DBDs: "Fork_head"]; Foxd2[gene ID: "ENSMUSG00000055210" species: "Mus musculus" TF status: "inferred" TF family: "Forkhead" DBDs: "Fork_head"]; Foxd3[gene ID: "ENSMUSG00000067261" species: "Mus musculus" TF status: "inferred" TF family: "Forkhead" DBDs: "Fork_head"]; Foxd4[gene ID: "ENSMUSG00000051490" species: "Mus musculus" TF status: "inferred" TF family: "Forkhead" DBDs: "Fork_head"]; Foxe1[gene ID: "ENSMUSG00000070990" species: "Mus musculus" TF status: "inferred" TF family: "Forkhead" DBDs: "Fork_head"]; Foxe3[gene ID: "ENSMUSG00000044518" species: "Mus musculus" TF status: "inferred" TF family: "Forkhead" DBDs: "Fork_head"]; Foxf1a[gene ID: "ENSMUSG00000042812" species: "Mus musculus" TF status: "inferred" TF family: "Forkhead" DBDs: "Fork_head"]; Foxf2[gene ID: "ENSMUSG00000038402" species: "Mus musculus" TF status: "inferred" TF family: "Forkhead" DBDs: "Fork_head"]; Foxi1[gene ID: "ENSMUSG00000047861" species: "Mus musculus" TF status: "inferred" TF family: "Forkhead" DBDs: "Fork_head"]; Foxi2[gene ID: "ENSMUSG00000048377" species: "Mus musculus" TF status: "inferred" TF family: "Forkhead" DBDs: "Fork_head"]; Foxi3[gene ID: "ENSMUSG00000055874" species: "Mus musculus" TF status: "inferred" TF family: "Forkhead" DBDs: "Fork_head"]; Foxk2[gene ID: "ENSMUSG00000039275" species: "Mus musculus" TF status: "inferred" TF family: "Forkhead" DBDs: "Fork_head"]; Foxl2[gene ID: "ENSMUSG00000050397" species: "Mus musculus" TF status: "inferred" TF family: "Forkhead" DBDs: "Fork_head"]; Foxq1[gene ID: "ENSMUSG00000038415" species: "Mus musculus" TF status: "inferred" TF family: "Forkhead" DBDs: "Fork_head"]; Foxs1[gene ID: "ENSMUSG00000074676" species: "Mus musculus" TF status: "inferred" TF family: "Forkhead" DBDs: "Fork_head"]; KIAA0415[gene ID: "ENSG00000242802" species: "Homo sapiens" TF status: "inferred" TF family:	*3.77214*	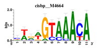	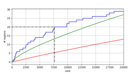	**link**	**link**	PWMs

#	Feature	NES	Logo	Recovery Curve	Candidate targets	All regions in top 20000	Database
	"Forkhead" DBDs: "Fork_head"]; Mnf[gene ID: "FBgn0036134" species: "Drosophila melanogaster" TF status: "inferred" TF family: "Forkhead" DBDs: "Fork_head"]; fd59A[gene ID: "FBgn0004896" species: "Drosophila melanogaster" TF status: "inferred" TF family: "Forkhead" DBDs: "Fork_head"]; fd96Ca[gene ID: "FBgn0004897" species: "Drosophila melanogaster" TF status: "inferred" TF family: "Forkhead" DBDs: "Fork_head"]; fd96Cb[gene ID: "FBgn0004898" species: "Drosophila melanogaster" TF status: "inferred" TF family: "Forkhead" DBDs: "Fork_head"]<br>Possible TFs: FOXA1						
287	☐ cisbp__M5527<br>Description: HNF4A[gene ID: "ENSG00000101076" species: "Homo sapiens" TF status: "direct" TF family: "Nuclear receptor" DBDs: "zf-C4"]; Hnf4g[gene ID: "ENSMUSG00000017688" species: "Mus musculus" TF status: "inferred" TF family: "Nuclear receptor" DBDs: "zf-C4"]<br>Possible TFs: HNF4A	*3.75424*	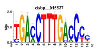	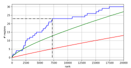	link	link	PWMs
288	☐ E044-H3K4me1-broadpeak<br>Description: H3K4me1 in Primary T regulatory cells from peripheral blood (E044, broadpeak)	*3.75174*		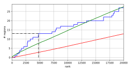	link	link	Histone modifications
289	☐ transfac_pro__M00720<br>Description: V$CACBINDINGPROTEIN_Q6: CAC-binding protein	*3.74804*	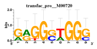	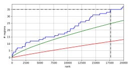	link	link	PWMs
290	☐ cisbp__M1552<br>Description: AC168977.1[gene ID: "ENSMUSG00000079808" species: "Mus musculus" TF status: "inferred" TF family: "SAND" DBDs: "SAND"]; Sp110[gene ID: "ENSMUSG00000070034" species: "Mus musculus" TF status: "direct" TF family: "SAND" DBDs: "SAND"]<br>Possible TFs: SP110	*3.74183*	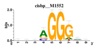	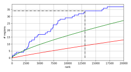	link	link	PWMs
291	☐ predrem__nrMotif279<br>Description: 144_fLung_L-DS17739.M743	*3.73611*	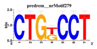	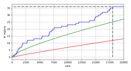	link	link	PWMs
292	☐ ENCFF001UGV<br>Description: NR2F2 ChIP-seq protocol v042211.1 on human HepG2	*3.73372*		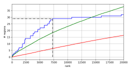	link	link	TF binding sites
293	☐ E069-H3K36me3<br>Description: H3K36me3 in Brain Cingulate Gyrus (E069,)	*3.73044*		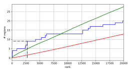	link	link	Histone modifications
294	☐ dbcorrdb__TCF7L2__ENCSR000EWT_1__m2<br>Description: TCF7L2 (ENCSR000EWT-1, motif 2)<br>Possible TFs: TCF7L2	*3.72612*	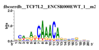	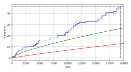	link	link	PWMs
295	☐ jaspar__MA0504.1<br>Description: NR2C2<br>Possible TFs: NR2C2	*3.72007*	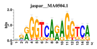	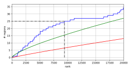	link	link	PWMs
296	☐ E056-H3K4me1-broadpeak<br>Description: H3K4me1 in Foreskin Fibroblast Primary Cells skin02 (E056, broadpeak)	*3.71489*		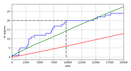	link	link	Histone modifications
297	☐ cisbp__M4703<br>Description: NR2F2[gene ID: "ENSG00000185551" species: "Homo sapiens" TF status: "direct" TF family: "Nuclear receptor" DBDs: "zf-C4"]<br>Possible TFs: NR2F2	*3.70927*	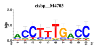	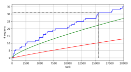	link	link	PWMs
298	☐ predrem__nrMotif356<br>Description: 10_CACO2-DS8235.M39	*3.70508*	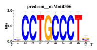	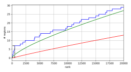	link	link	PWMs
299	☐ E030-H3K4me1-broadpeak<br>Description: H3K4me1 in Primary neutrophils from peripheral blood (E030, broadpeak)	*3.69881*		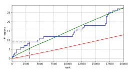	link	link	Histone modifications
300	☐ cisbp__M4564<br>Description: ESR1[gene ID: "ENSG00000091831" species: "Homo sapiens" TF status: "direct" TF family: "Nuclear receptor" DBDs: "zf-C4"]; Esr2[gene ID: "ENSMUSG00000021055" species: "Mus musculus" TF status: "inferred" TF family: "Nuclear receptor" DBDs: "zf-C4"]<br>Possible TFs: ESR1	*3.69316*	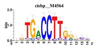	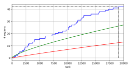	link	link	PWMs

#	Feature	NES	Logo	Recovery Curve	Candidate targets	All regions in top 20000	Database
301	☐ hocomoco__THB_MOUSE.H11MO.0.D<br>Description: THB_MOUSE<br>Possible TFs: THRB	*3.67704*	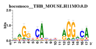	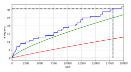	link	link	PWMs
302	☐ hocomoco__ZFX_HUMAN.H11MO.1.A<br>Description: ZFX_HUMAN<br>Possible TFs: ZFX	*3.67212*	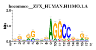	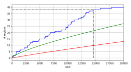	link	link	PWMs
303	☐ ENCFF001TSH<br>Description: FOXA2 ChIP-seq (protocol v041610.2) on A549 treated with ethanol at 0.02%	*3.67019*		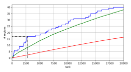	link	link	TF binding sites
304	☐ homer__AGGCCTAG_ZNF711<br>Description: ZNF711(Zf)/SHSY5Y-ZNF711-ChIP-Seq(GSE20673)/Homer<br>Possible TFs: ZNF711	*3.65496*	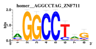	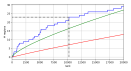	link	link	PWMs
305	☐ hdpi__RPS10<br>Description: RPS10<br>Possible TFs: RPS10	*3.63835*	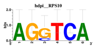	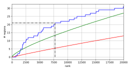	link	link	PWMs
306	☐ hocomoco__PPARG_MOUSE.H11MO.0.A<br>Description: PPARG_MOUSE	*3.63819*	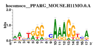	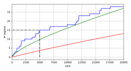	link	link	PWMs
307	☐ dbcorrdb__FOXA1__ENCSR000BPX_1__m2<br>Description: FOXA1 (ENCSR000BPX-1, motif 2)<br>Possible TFs: FOXA1	*3.62304*	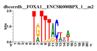	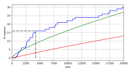	link	link	PWMs
308	☐ dbcorrdb__MBD4__ENCSR000BQW_1__m1<br>Description: MBD4 (ENCSR000BQW-1, motif 1)<br>Possible TFs: MBD4	*3.62280*	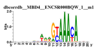	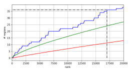	link	link	PWMs
309	☐ transfac_pro__M09224<br>Description: P$ATHB5_04: ATHB5	*3.61982*	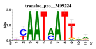	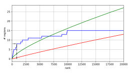	link	link	PWMs
310	☐ transfac_pro__M04811<br>Description: V$RELA_03: RelA-p65<br>Possible TFs: RELA	*3.61788*	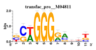	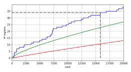	link	link	PWMs
311	☐ cisbp__M6532<br>Description: VDR[gene ID: "ENSG00000111424" species: "Homo sapiens" TF status: "direct" TF family: "Nuclear receptor" DBDs: "zf-C4"]<br>Possible TFs: VDR	*3.61256*	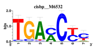	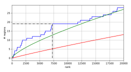	link	link	PWMs
312	☐ transfac_pro__M03568<br>Description: V$TR4_Q2_01: TR4<br>Possible TFs: NR2C2	*3.60692*	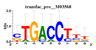	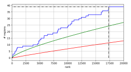	link	link	PWMs
313	☐ hocomoco__PRD16_MOUSE.H11MO.0.B<br>Description: PRD16_MOUSE<br>Possible TFs: PRDM16	*3.60289*	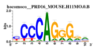	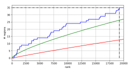	link	link	PWMs
314	☐ transfac_pro__M07341<br>Description: V$TORC2_Q3: TORC2<br>Possible TFs: CRTC2	*3.59177*	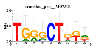	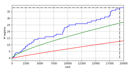	link	link	PWMs
315	☐ hdpi__GRHPR<br>Description: GRHPR<br>Possible TFs: GRHPR	*3.57718*	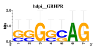	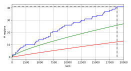	link	link	PWMs
316	☐ neph__UW.Motif.0020<br>Description: aatNattNac	*3.55534*	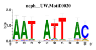	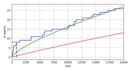	link	link	PWMs
317	☐ ENCFF001UGP<br>Description: MBD4 ChIP-seq protocol v042211.1 on human HepG2	*3.55260*		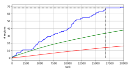	link	link	TF binding sites
318	☐ ENCFF001UGK<br>Description: JUND ChIP-seq protocol PCR1x on human HepG2	*3.54654*		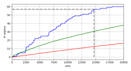	link	link	TF binding sites
319	☐ hocomoco__COT1_MOUSE.H11MO.0.B<br>Description: COT1_MOUSE	*3.54374*	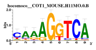	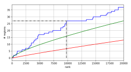	link	link	PWMs

#	Feature	NES	Logo	Recovery Curve	Candidate targets	All regions in top 20000	Database
320	☐ hocomoco__VDR_HUMAN.H11MO.1.A<br>Description: VDR_HUMAN<br>Possible TFs: VDR	*3.54205*	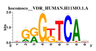	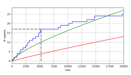	**link**	**link**	PWMs
321	☐ cisbp__M0854<br>Description: M0854	*3.53939*	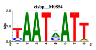	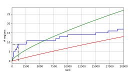	**link**	**link**	PWMs
322	☐ hdpi__C9orf156<br>Description: C9orf156<br>Possible TFs: TRMO	*3.53640*	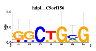	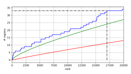	**link**	**link**	PWMs
323	☐ hocomoco__PPARG_MOUSE.H11MO.1.A<br>Description: PPARG_MOUSE	*3.52883*	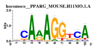	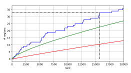	**link**	**link**	PWMs
324	☐ cisbp__M4566<br>Description: CG11152[gene ID: "FBgn0039937" species: "Drosophila melanogaster" TF status: "inferred" TF family: "Forkhead" DBDs: "Fork_head"]; CG9571[gene ID: "FBgn0031086" species: "Drosophila melanogaster" TF status: "inferred" TF family: "Forkhead" DBDs: "Fork_head"]; FOXA1[gene ID: "ENSG00000129514" species: "Homo sapiens" TF status: "direct" TF family: "Forkhead" DBDs: "Fork_head"]; FOXB2[gene ID: "ENSG00000204612" species: "Homo sapiens" TF status: "inferred" TF family: "Forkhead" DBDs: "Fork_head"]; FOXD4L1[gene ID: "ENSG00000184492" species: "Homo sapiens" TF status: "inferred" TF family: "Forkhead" DBDs: "Fork_head"]; FOXD4L3[gene ID: "ENSG00000187559" species: "Homo sapiens" TF status: "inferred" TF family: "Forkhead" DBDs: "Fork_head"]; FOXD4L5[gene ID: "ENSG00000204779" species: "Homo sapiens" TF status: "inferred" TF family: "Forkhead" DBDs: "Fork_head"]; FOXD4L6[gene ID: "ENSG00000204793" species: "Homo sapiens" TF status: "inferred" TF family: "Forkhead" DBDs: "Fork_head"]; FOXD4[gene ID: "ENSG00000170122" species: "Homo sapiens" TF status: "inferred" TF family: "Forkhead" DBDs: "Fork_head"]; FOXE1[gene ID: "ENSG00000178919" species: "Homo sapiens" TF status: "inferred" TF family: "Forkhead" DBDs: "Fork_head"]; FOXE3[gene ID: "ENSG00000186790" species: "Homo sapiens" TF status: "inferred" TF family: "Forkhead" DBDs: "Fork_head"]; FOXI2[gene ID: "ENSG00000186766" species: "Homo sapiens" TF status: "inferred" TF family: "Forkhead" DBDs: "Fork_head"]; FOXK2[gene ID: "ENSG00000141568" species: "Homo sapiens" TF status: "inferred" TF family: "Forkhead" DBDs: "Fork_head"]; FOXS1[gene ID: "ENSG00000179772" species: "Homo sapiens" TF status: "inferred" TF family: "Forkhead" DBDs: "Fork_head"]; Foxa1[gene ID: "ENSMUSG00000035451" species: "Mus musculus" TF status: "inferred" TF family: "Forkhead" DBDs: "Fork_head"]; Foxa3[gene ID: "ENSMUSG00000040891" species: "Mus musculus" TF status: "inferred" TF family: "Forkhead" DBDs: "Fork_head"]; Foxb2[gene ID: "ENSMUSG00000056829" species: "Mus musculus" TF status: "inferred" TF family: "Forkhead" DBDs: "Fork_head"]; Foxd2[gene ID: "ENSMUSG00000055210" species: "Mus musculus" TF status: "inferred" TF family: "Forkhead" DBDs: "Fork_head"]; Foxd3[gene ID: "ENSMUSG00000067261" species: "Mus musculus" TF status: "inferred" TF family: "Forkhead" DBDs: "Fork_head"]; Foxd4[gene ID: "ENSMUSG00000051490" species: "Mus musculus" TF status: "inferred" TF family: "Forkhead" DBDs: "Fork_head"]; Foxe1[gene ID: "ENSMUSG00000070990" species: "Mus musculus" TF status: "inferred" TF family: "Forkhead" DBDs: "Fork_head"]; Foxe3[gene ID: "ENSMUSG00000044518" species: "Mus musculus" TF status: "inferred" TF family: "Forkhead" DBDs: "Fork_head"]; Foxf1a[gene ID: "ENSMUSG00000042812" species: "Mus musculus" TF status: "inferred" TF family: "Forkhead" DBDs: "Fork_head"]; Foxf2[gene ID: "ENSMUSG00000038402" species: "Mus musculus" TF status: "inferred" TF family: "Forkhead" DBDs: "Fork_head"]; Foxi1[gene ID: "ENSMUSG00000047861" species: "Mus musculus" TF status: "inferred" TF family: "Forkhead" DBDs: "Fork_head"]; Foxi2[gene ID: "ENSMUSG00000048377" species: "Mus musculus" TF status: "inferred" TF family: "Forkhead" DBDs: "Fork_head"]; Foxi3[gene ID: "ENSMUSG00000055874" species: "Mus musculus" TF status: "inferred" TF family: "Forkhead" DBDs: "Fork_head"]; Foxk2[gene ID: "ENSMUSG00000039275" species: "Mus musculus" TF status: "inferred" TF family: "Forkhead" DBDs: "Fork_head"]; Foxl2[gene ID:	*3.52528*	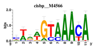	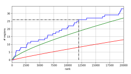	**link**	**link**	PWMs

#	Feature	NES	Logo	Recovery Curve	Candidate targets	All regions in top 20000	Database
	"ENSMUSG00000050397" species: "Mus musculus" TF status: "inferred" TF family: "Forkhead" DBDs: "Fork_head"]; Foxq1[gene ID: "ENSMUSG00000038415" species: "Mus musculus" TF status: "inferred" TF family: "Forkhead" DBDs: "Fork_head"]; Foxs1[gene ID: "ENSMUSG00000074676" species: "Mus musculus" TF status: "inferred" TF family: "Forkhead" DBDs: "Fork_head"]; KIAA0415[gene ID: "ENSG00000242802" species: "Homo sapiens" TF status: "inferred" TF family: "Forkhead" DBDs: "Fork_head"]; Mnf[gene ID: "FBgn0036134" species: "Drosophila melanogaster" TF status: "inferred" TF family: "Forkhead" DBDs: "Fork_head"]; fd59A[gene ID: "FBgn0004896" species: "Drosophila melanogaster" TF status: "inferred" TF family: "Forkhead" DBDs: "Fork_head"]; fd96Ca[gene ID: "FBgn0004897" species: "Drosophila melanogaster" TF status: "inferred" TF family: "Forkhead" DBDs: "Fork_head"]; fd96Cb[gene ID: "FBgn0004898" species: "Drosophila melanogaster" TF status: "inferred" TF family: "Forkhead" DBDs: "Fork_head"]<br>Possible TFs: FOXA1						
325	☐ taipale_cyt_meth__HNF4A_NRGGTCAAAGGTCAN_eDBD_meth<br>Description: HNF4A [Nuclear_receptor, CpG-meth]<br>Possible TFs: HNF4A	3.52149	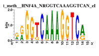	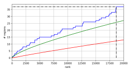	link	link	PWMs
326	☐ transfac_public__M00528<br>Description: V$PPARG_03: PPAR<br>Possible TFs: PPARG	3.51045	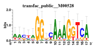	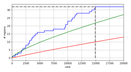	link	link	PWMs
327	☐ factorbook__UA13<br>Description: UA13	3.50997	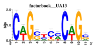	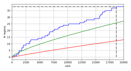	link	link	PWMs
328	☐ E102-H3K4me1-broadpeak<br>Description: H3K4me1 in Rectal Mucosa Donor 31 (E102, broadpeak)	3.50609		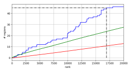	link	link	Histone modifications
329	☐ transfac_pro__M00646<br>Description: V$LFA1_Q6: LF-A1<br>Possible TFs: HNF1B	3.50457	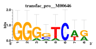	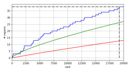	link	link	PWMs
330	☐ cisbp__M1495<br>Description: Nr2c2[gene ID: "ENSMUSG00000005893" species: "Mus musculus" TF status: "inferred" TF family: "Nuclear receptor" DBDs: "zf-C4"]	3.50054	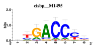	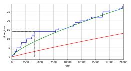	link	link	PWMs
331	☐ E079-H3K4me1<br>Description: H3K4me1 in Esophagus (E079,)	3.46924		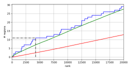	link	link	Histone modifications
332	☐ cisbp__M6177<br>Description: NR2F1[gene ID: "ENSG00000175745" species: "Homo sapiens" TF status: "direct" TF family: "Nuclear receptor" DBDs: "zf-C4"]<br>Possible TFs: NR2F1	3.44904	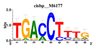	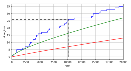	link	link	PWMs
333	☐ transfac_pro__M01269<br>Description: V$NURR1_Q3: NURR1<br>Possible TFs: NR4A2	3.44421	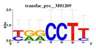	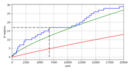	link	link	PWMs
334	☐ E041-H3K4me1<br>Description: H3K4me1 in Primary T helper cells PMA-I stimulated (E041,)	3.44376		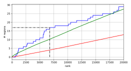	link	link	Histone modifications
335	☐ E029-H3K4me1<br>Description: H3K4me1 in Primary monocytes from peripheral blood (E029,)	3.44262		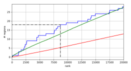	link	link	Histone modifications
336	☐ transfac_pro__M00820<br>Description: P$HAHB4_01: HAHB-4	3.43784	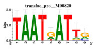	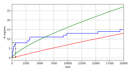	link	link	PWMs
337	☐ transfac_pro__M06090<br>Description: V$ZNF325_01: ZNF325<br>Possible TFs: ZNF12	3.42680	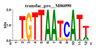	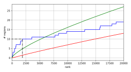	link	link	PWMs
338	☐ hocomoco__ETV4_MOUSE.H11MO.0.B<br>Description: ETV4_MOUSE<br>Possible TFs: ETV4	3.42495	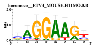	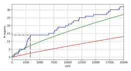	link	link	PWMs
339	☐ transfac_pro__M09242<br>Description: P$ATHB18_01: ATHB18	3.42462	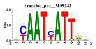	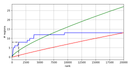	link	link	PWMs

#	Feature	NES	Logo	Recovery Curve	Candidate targets	All regions in top 20000	Database
340	☐ jaspar__MA0148.3<br>Description: FOXA1<br>Possible TFs: FOXA1	*3.42390*	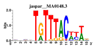	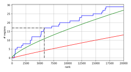	link	link	PWMs
341	☐ hdpi__AGGF1<br>Description: AGGF1<br>Possible TFs: AGGF1	*3.42277*	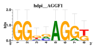	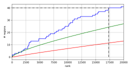	link	link	PWMs
342	☐ cisbp__M4511<br>Description: AL662824.5[gene ID: "ENSG00000227322" species: "Homo sapiens" TF status: "inferred" TF family: "Nuclear receptor" DBDs: "zf-C4"]; AL844527.7[gene ID: "ENSG00000206289" species: "Homo sapiens" TF status: "inferred" TF family: "Nuclear receptor" DBDs: "zf-C4"]; CR354565.3[gene ID: "ENSG00000231321" species: "Homo sapiens" TF status: "inferred" TF family: "Nuclear receptor" DBDs: "zf-C4"]; CR759733.4[gene ID: "ENSG00000228333" species: "Homo sapiens" TF status: "inferred" TF family: "Nuclear receptor" DBDs: "zf-C4"]; CR936877.3[gene ID: "ENSG00000235712" species: "Homo sapiens" TF status: "inferred" TF family: "Nuclear receptor" DBDs: "zf-C4"]; RXRA[gene ID: "ENSG00000186350" species: "Homo sapiens" TF status: "direct" TF family: "Nuclear receptor" DBDs: "zf-C4"]<br>Possible TFs: RXRA	*3.41971*	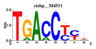	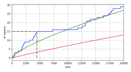	link	link	PWMs
343	☐ hdpi__DUS3L<br>Description: DUS3L<br>Possible TFs: DUS3L	*3.41890*	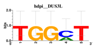	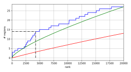	link	link	PWMs
344	☐ ENCFF001UHA<br>Description: EP300 ChIP-seq protocol v041610.1 on human HepG2	*3.41733*		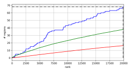	link	link	TF binding sites
345	☐ dbcorrdb__BCL3__ENCSR000BQH_1__m1<br>Description: BCL3 (ENCSR000BQH-1, motif 1)<br>Possible TFs: BCL3	*3.41697*	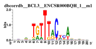	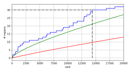	link	link	PWMs
346	☐ ENCFF001UFZ<br>Description: FOXA2 ChIP-seq protocol v041610.1 on human HepG2	*3.39938*		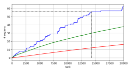	link	link	TF binding sites
347	☐ predrem__nrMotif805<br>Description: 10_CACO2-DS8235.M170	*3.39932*	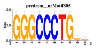	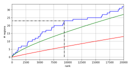	link	link	PWMs
348	☐ ENCFF001VKF<br>Description: HNF4A ChIP-seq on human HepG2 treated with forskolin	*3.39038*		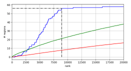	link	link	TF binding sites
349	☐ taipale_cyt_meth__HNF4A_NRGGTCAAAGGTCAN_eDBD<br>Description: HNF4A [Nuclear_receptor]<br>Possible TFs: HNF4A	*3.37885*	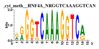	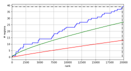	link	link	PWMs
350	☐ homer__ACAGGAAGTG_ERG<br>Description: ERG(ETS)/VCaP-ERG-ChIP-Seq(GSE14097)/Homer<br>Possible TFs: ERG	*3.37578*	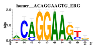	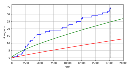	link	link	PWMs
351	☐ neph__UW.Motif.0283<br>Description: ggccNNNNggga	*3.37409*	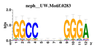	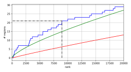	link	link	PWMs
352	☐ E118-H3K4me3<br>Description: H3K4me3 in HepG2 Hepatocellular Carcinoma Cell Line (E118,)	*3.37340*		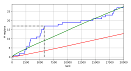	link	link	Histone modifications
353	☐ dbcorrdb__EP300__ENCSR000BPW_1__m2<br>Description: EP300 (ENCSR000BPW-1, motif 2)<br>Possible TFs: EP300	*3.37184*	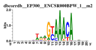	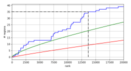	link	link	PWMs
354	☐ jaspar__MA0951.1<br>Description: ATHB-16	*3.37095*	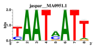	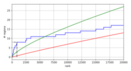	link	link	PWMs
355	☐ tfdimers__MD00041<br>Description: M01456_forward_14_M01712_reverse dimer: Hmbox1 / CRX<br>Possible TFs: CRX, HMBOX1	*3.36982*	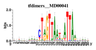	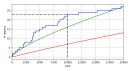	link	link	PWMs
356	☐ hocomoco__FOXM1_HUMAN.H11MO.0.A<br>Description: FOXM1_HUMAN<br>Possible TFs: FOXM1	*3.36918*	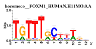	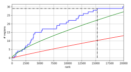	link	link	PWMs

#	Feature	NES	Logo	Recovery Curve	Candidate targets	All regions in top 20000	Database
357	☐ cisbp__M5524<br>Description: HNF4A[gene ID: "ENSG00000101076" species: "Homo sapiens" TF status: "direct" TF family: "Nuclear receptor" DBDs: "zf-C4"]; Hnf4g[gene ID: "ENSMUSG00000017688" species: "Mus musculus" TF status: "inferred" TF family: "Nuclear receptor" DBDs: "zf-C4"]<br>Possible TFs: HNF4A	3.36789	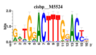	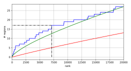	link	link	PWMs
358	☐ hocomoco__SNAI1_HUMAN.H11MO.0.C<br>Description: SNAI1_HUMAN<br>Possible TFs: SNAI1	3.35612	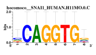	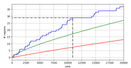	link	link	PWMs
359	☐ predrem__nrMotif1125<br>Description: 10_CACO2-DS8235.M848	3.35612	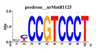	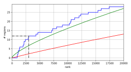	link	link	PWMs
360	☐ transfac_pro__M08881<br>Description: V$ETSLIKE_Q4: ETSLIKE<br>Possible TFs: FEV, ETV2, FLI1, ETS2, ERF, ERG, GABPA, ETS1	3.35394	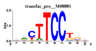	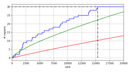	link	link	PWMs
361	☐ cisbp__M6114<br>Description: CG11152[gene ID: "FBgn0039937" species: "Drosophila melanogaster" TF status: "inferred" TF family: "Forkhead" DBDs: "Fork_head"]; CG9571[gene ID: "FBgn0031086" species: "Drosophila melanogaster" TF status: "inferred" TF family: "Forkhead" DBDs: "Fork_head"]; FOXA1[gene ID: "ENSG00000129514" species: "Homo sapiens" TF status: "direct" TF family: "Forkhead" DBDs: "Fork_head"]; FOXB2[gene ID: "ENSG00000204612" species: "Homo sapiens" TF status: "inferred" TF family: "Forkhead" DBDs: "Fork_head"]; FOXD4L1[gene ID: "ENSG00000184492" species: "Homo sapiens" TF status: "inferred" TF family: "Forkhead" DBDs: "Fork_head"]; FOXD4L3[gene ID: "ENSG00000187559" species: "Homo sapiens" TF status: "inferred" TF family: "Forkhead" DBDs: "Fork_head"]; FOXD4L5[gene ID: "ENSG00000204779" species: "Homo sapiens" TF status: "inferred" TF family: "Forkhead" DBDs: "Fork_head"]; FOXD4L6[gene ID: "ENSG00000204793" species: "Homo sapiens" TF status: "inferred" TF family: "Forkhead" DBDs: "Fork_head"]; FOXD4[gene ID: "ENSG00000170122" species: "Homo sapiens" TF status: "inferred" TF family: "Forkhead" DBDs: "Fork_head"]; FOXE1[gene ID: "ENSG00000178919" species: "Homo sapiens" TF status: "inferred" TF family: "Forkhead" DBDs: "Fork_head"]; FOXE3[gene ID: "ENSG00000186790" species: "Homo sapiens" TF status: "inferred" TF family: "Forkhead" DBDs: "Fork_head"]; FOXI2[gene ID: "ENSG00000186766" species: "Homo sapiens" TF status: "inferred" TF family: "Forkhead" DBDs: "Fork_head"]; FOXK2[gene ID: "ENSG00000141568" species: "Homo sapiens" TF status: "inferred" TF family: "Forkhead" DBDs: "Fork_head"]; FOXS1[gene ID: "ENSG00000179772" species: "Homo sapiens" TF status: "inferred" TF family: "Forkhead" DBDs: "Fork_head"]; Foxa1[gene ID: "ENSMUSG00000035451" species: "Mus musculus" TF status: "inferred" TF family: "Forkhead" DBDs: "Fork_head"]; Foxa3[gene ID: "ENSMUSG00000040891" species: "Mus musculus" TF status: "inferred" TF family: "Forkhead" DBDs: "Fork_head"]; Foxb2[gene ID: "ENSMUSG00000056829" species: "Mus musculus" TF status: "inferred" TF family: "Forkhead" DBDs: "Fork_head"]; Foxd2[gene ID: "ENSMUSG00000055210" species: "Mus musculus" TF status: "inferred" TF family: "Forkhead" DBDs: "Fork_head"]; Foxd3[gene ID: "ENSMUSG00000067261" species: "Mus musculus" TF status: "inferred" TF family: "Forkhead" DBDs: "Fork_head"]; Foxd4[gene ID: "ENSMUSG00000051490" species: "Mus musculus" TF status: "inferred" TF family: "Forkhead" DBDs: "Fork_head"]; Foxe1[gene ID: "ENSMUSG00000070990" species: "Mus musculus" TF status: "inferred" TF family: "Forkhead" DBDs: "Fork_head"]; Foxe3[gene ID: "ENSMUSG00000044518" species: "Mus musculus" TF status: "inferred" TF family: "Forkhead" DBDs: "Fork_head"]; Foxf1a[gene ID: "ENSMUSG00000042812" species: "Mus musculus" TF status: "inferred" TF family: "Forkhead" DBDs: "Fork_head"]; Foxf2[gene ID: "ENSMUSG00000038402" species: "Mus musculus" TF status: "inferred" TF family: "Forkhead" DBDs: "Fork_head"]; Foxi1[gene ID: "ENSMUSG00000047861" species: "Mus musculus" TF status: "inferred" TF family: "Forkhead" DBDs: "Fork_head"]; Foxi2[gene ID: "ENSMUSG00000048377" species: "Mus musculus" TF status: "inferred" TF family: "Forkhead" DBDs: "Fork_head"]; Foxi3[gene ID: "ENSMUSG00000055874" species: "Mus musculus" TF status: "inferred" TF	3.35088	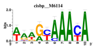	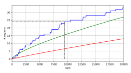	link	link	PWMs

#	Feature	NES	Logo	Recovery Curve	Candidate targets	All regions in top 20000	Database
	family: "Forkhead" DBDs: "Fork_head"]; Foxk2[gene ID: "ENSMUSG00000039275" species: "Mus musculus" TF status: "inferred" TF family: "Forkhead" DBDs: "Fork_head"]; Foxl2[gene ID: "ENSMUSG00000050397" species: "Mus musculus" TF status: "inferred" TF family: "Forkhead" DBDs: "Fork_head"]; Foxq1[gene ID: "ENSMUSG00000038415" species: "Mus musculus" TF status: "inferred" TF family: "Forkhead" DBDs: "Fork_head"]; Foxs1[gene ID: "ENSMUSG00000074676" species: "Mus musculus" TF status: "inferred" TF family: "Forkhead" DBDs: "Fork_head"]; KIAA0415[gene ID: "ENSG00000242802" species: "Homo sapiens" TF status: "inferred" TF family: "Forkhead" DBDs: "Fork_head"]; Mnf[gene ID: "FBgn0036134" species: "Drosophila melanogaster" TF status: "inferred" TF family: "Forkhead" DBDs: "Fork_head"]; fd59A[gene ID: "FBgn0004896" species: "Drosophila melanogaster" TF status: "inferred" TF family: "Forkhead" DBDs: "Fork_head"]; fd96Ca[gene ID: "FBgn0004897" species: "Drosophila melanogaster" TF status: "inferred" TF family: "Forkhead" DBDs: "Fork_head"]; fd96Cb[gene ID: "FBgn0004898" species: "Drosophila melanogaster" TF status: "inferred" TF family: "Forkhead" DBDs: "Fork_head"]<br>Possible TFs: FOXA1						
362	☐ ENCFF001USX<br>Description: MYC ChIP-seq on human HepG2	*3.33559*		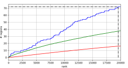	**link**	**link**	TF binding sites
363	☐ hocomoco__FOXA3_HUMAN.H11MO.0.B<br>Description: FOXA3_HUMAN<br>Possible TFs: FOXA3	*3.33533*	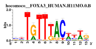	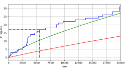	**link**	**link**	PWMs
364	☐ cisbp__M3919<br>Description: SP1[gene ID: "ENSG00000185591" species: "Homo sapiens" TF status: "direct" TF family: "C2H2 ZF" DBDs: "zf-C2H2"]; SP5[gene ID: "ENSG00000204335" species: "Homo sapiens" TF status: "inferred" TF family: "C2H2 ZF" DBDs: "zf-C2H2"]; SP6[gene ID: "ENSG00000189120" species: "Homo sapiens" TF status: "inferred" TF family: "C2H2 ZF" DBDs: "zf-C2H2"]; SP7[gene ID: "ENSG00000170374" species: "Homo sapiens" TF status: "inferred" TF family: "C2H2 ZF" DBDs: "zf-C2H2"]; SP9[gene ID: "ENSG00000217236" species: "Homo sapiens" TF status: "inferred" TF family: "C2H2 ZF" DBDs: "zf-C2H2"]; Sp2[gene ID: "ENSMUSG00000018678" species: "Mus musculus" TF status: "inferred" TF family: "C2H2 ZF" DBDs: "zf-C2H2"]; Sp3[gene ID: "ENSMUSG00000027109" species: "Mus musculus" TF status: "inferred" TF family: "C2H2 ZF" DBDs: "zf-C2H2"]; Sp5[gene ID: "ENSMUSG00000075304" species: "Mus musculus" TF status: "inferred" TF family: "C2H2 ZF" DBDs: "zf-C2H2"]; Sp6[gene ID: "ENSMUSG00000038560" species: "Mus musculus" TF status: "inferred" TF family: "C2H2 ZF" DBDs: "zf-C2H2"]; Sp7[gene ID: "ENSMUSG00000060284" species: "Mus musculus" TF status: "inferred" TF family: "C2H2 ZF" DBDs: "zf-C2H2"]; Sp8[gene ID: "ENSMUSG00000048562" species: "Mus musculus" TF status: "inferred" TF family: "C2H2 ZF" DBDs: "zf-C2H2"]; Sp9[gene ID: "ENSMUSG00000068859" species: "Mus musculus" TF status: "inferred" TF family: "C2H2 ZF" DBDs: "zf-C2H2"]<br>Possible TFs: SP1	*3.33154*	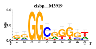	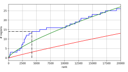	**link**	**link**	PWMs
365	☐ hocomoco__RARA_HUMAN.H11MO.0.A<br>Description: RARA_HUMAN<br>Possible TFs: RARA	*3.32235*	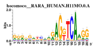	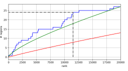	**link**	**link**	PWMs
366	☐ ENCFF001SVW<br>Description: H3K27ac ChIP-seq on human HeLa-S3	*3.31734*		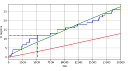	**link**	**link**	Histone modifications
367	☐ swissregulon__hs__NR1H4.p2<br>Description: hs__NR1H4.p2<br>Possible TFs: NR1H4	*3.31445*	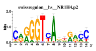	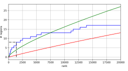	**link**	**link**	PWMs
368	☐ hocomoco__SMAD3_HUMAN.H11MO.0.B<br>Description: SMAD3_HUMAN<br>Possible TFs: SMAD3	*3.30986*	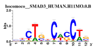	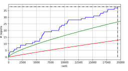	**link**	**link**	PWMs

#	Feature	NES	Logo	Recovery Curve	Candidate targets	All regions in top 20000	Database
369	☐ cisbp__M6433<br>Description: PPARG[gene ID: "ENSG00000132170" species: "Homo sapiens" TF status: "direct" TF family: "Nuclear receptor" DBDs: "zf-C4"]; Ppara[gene ID: "ENSMUSG00000022383" species: "Mus musculus" TF status: "inferred" TF family: "Nuclear receptor" DBDs: "zf-C4"]; Ppard[gene ID: "ENSMUSG00000002250" species: "Mus musculus" TF status: "inferred" TF family: "Nuclear receptor" DBDs: "zf-C4"]; Pparg[gene ID: "ENSMUSG00000000440" species: "Mus musculus" TF status: "inferred" TF family: "Nuclear receptor" DBDs: "zf-C4"]<br>Possible TFs: PPARG	*3.29551*	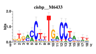	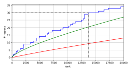	link	link	PWMs
370	☐ transfac_public__M00008<br>Description: V$SP1_01: Sp1<br>Possible TFs: SP1	*3.29100*	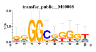	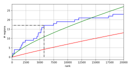	link	link	PWMs
371	☐ cisbp__M0801<br>Description: GZF3[gene ID: "YJL110C" species: "Saccharomyces cerevisiae" TF status: "direct" TF family: "GATA" DBDs: "GATA"]<br>Possible TFs: TRPS1, GATA6, GATA4, GATA5, GATA2, GATA3, GATA1	*3.28915*	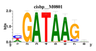	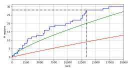	link	link	PWMs
372	☐ homer__AAAGTAAACA_FOXA1_GSE26831<br>Description: FOXA1(Forkhead)/MCF7-FOXA1-ChIP-Seq(GSE26831)/Homer<br>Possible TFs: FOXA1	*3.28842*	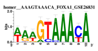	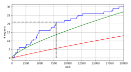	link	link	PWMs
373	☐ homer__NNNVCTGWGYAAACASN_Fox_Ebox<br>Description: Fox:Ebox(Forkhead,bHLH)/Panc1-Foxa2-ChIP-Seq(GSE47459)/Homer<br>Possible TFs: FOXA2	*3.28496*	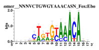	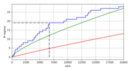	link	link	PWMs
374	☐ homer__AAAGTAAACA_FOXA1_GSE27824<br>Description: FOXA1(Forkhead)/LNCAP-FOXA1-ChIP-Seq(GSE27824)/Homer<br>Possible TFs: FOXA1	*3.28052*	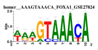	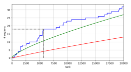	link	link	PWMs
375	☐ transfac_pro__M03819<br>Description: V$ELK1_Q6: Elk-1<br>Possible TFs: ELK1	*3.27980*	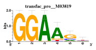	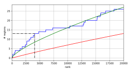	link	link	PWMs
376	☐ hocomoco__PPARA_MOUSE.H11MO.0.A<br>Description: PPARA_MOUSE<br>Possible TFs: PPARA	*3.26932*	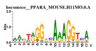	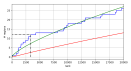	link	link	PWMs
377	☐ E101-H3K27ac<br>Description: H3K27ac in Rectal Mucosa Donor 29 (E101,)	*3.25919*		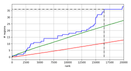	link	link	Histone modifications
378	☐ hocomoco__RARB_HUMAN.H11MO.0.D<br>Description: RARB_HUMAN<br>Possible TFs: RARB	*3.25844*	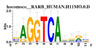	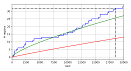	link	link	PWMs
379	☐ cisbp__M6222<br>Description: ENSMUSG00000044690[gene ID: "ENSMUSG00000044690" species: "Mus musculus" TF status: "inferred" TF family: "Ets" DBDs: "Ets"]; ETV4[gene ID: "ENSG00000175832" species: "Homo sapiens" TF status: "direct" TF family: "Ets" DBDs: "Ets"]; Ets2[gene ID: "ENSMUSG00000022895" species: "Mus musculus" TF status: "inferred" TF family: "Ets" DBDs: "Ets"]; Etv2[gene ID: "ENSMUSG00000006311" species: "Mus musculus" TF status: "inferred" TF family: "Ets" DBDs: "Ets"]; Fev[gene ID: "ENSMUSG00000055197" species: "Mus musculus" TF status: "inferred" TF family: "Ets" DBDs: "Ets"]<br>Possible TFs: ETV4	*3.25756*	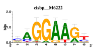	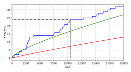	link	link	PWMs
380	☐ neph__UW.Motif.0010<br>Description: ctgggNNNNNNNcca	*3.25280*	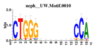	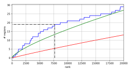	link	link	PWMs
381	☐ cisbp__M1457<br>Description: Nr2f1[gene ID: "ENSMUSG00000069171" species: "Mus musculus" TF status: "direct" TF family: "Nuclear receptor" DBDs: "zf-C4"]<br>Possible TFs: NR2F1	*3.24676*	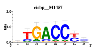	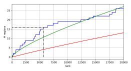	link	link	PWMs
382	☐ neph__UW.Motif.0131<br>Description: aggccNNNNNgcc	*3.24523*	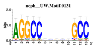	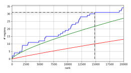	link	link	PWMs
383	☐ cisbp__M0534<br>Description: RGM1[gene ID: "YMR182C" species: "Saccharomyces cerevisiae" TF status: "direct" TF family: "C2H2 ZF" DBDs: "zf-C2H2"]	*3.24369*	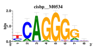	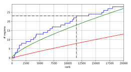	link	link	PWMs

#	Feature	NES	Logo	Recovery Curve	Candidate targets	All regions in top 20000	Database
384	☐ ENCFF001UGT<br>Description: NFIC ChIP-seq protocol v042211.1 on human HepG2	3.23622		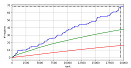	link	link	TF binding sites
385	☐ factorbook__NR2C2<br>Description: NR2C2<br>Possible TFs: NR2C2	3.23459	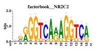	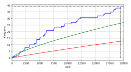	link	link	PWMs
386	☐ E123-H3K4me1<br>Description: H3K4me1 in K562 Leukemia Cells (E123,)	3.23319		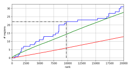	link	link	Histone modifications
387	☐ neph__UW.Motif.0116<br>Description: cNNNNccNNcaggg	3.22548	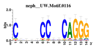	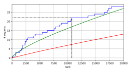	link	link	PWMs
388	☐ ENCFF001UFI<br>Description: CEBPB ChIP-seq protocol v041610.1 on human HepG2	3.21733		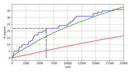	link	link	TF binding sites
389	☐ yetfasco__YMR037C_1380<br>Description: YMR037C_1380<br>Possible TFs: MECOM, PRDM16, PRDM13	3.20920	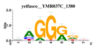	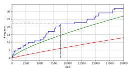	link	link	PWMs
390	☐ cisbp__M0394<br>Description: Plagl1[gene ID: "ENSMUSG00000019817" species: "Mus musculus" TF status: "direct" TF family: "C2H2 ZF" DBDs: "zf-C2H2"]<br>Possible TFs: PLAGL1	3.20896	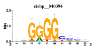	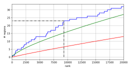	link	link	PWMs
391	☐ E117-H3K4me1<br>Description: H3K4me1 in HeLa-S3 Cervical Carcinoma Cell Line (E117,)	3.20782		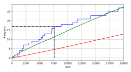	link	link	Histone modifications
392	☐ cisbp__M6445<br>Description: RARB[gene ID: "ENSG00000077092" species: "Homo sapiens" TF status: "direct" TF family: "Nuclear receptor" DBDs: "zf-C4"]<br>Possible TFs: RARB	3.20187	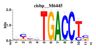	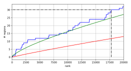	link	link	PWMs
393	☐ transfac_pro__M00339<br>Description: V$ETS1_B: c-Ets-1<br>Possible TFs: ETS1	3.20163	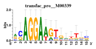	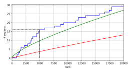	link	link	PWMs
394	☐ fantom__motif97_TTCCCT<br>Description: DENOVO_DMF_HUMAN: motif984	3.19969	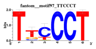	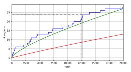	link	link	PWMs
395	☐ transfac_pro__M00967<br>Description: V$HNF4_Q6: HNF-4, COUP<br>Possible TFs: NR2F1, HNF4G, HNF4A, NR2F2	3.19856	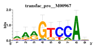	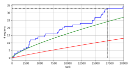	link	link	PWMs
396	☐ transfac_pro__M03579<br>Description: V$PEA3_Q6_02: PEA3<br>Possible TFs: ETV4	3.19340	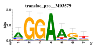	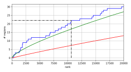	link	link	PWMs
397	☐ hocomoco__COT2_MOUSE.H11MO.0.A<br>Description: COT2_MOUSE<br>Possible TFs: NR2F2	3.19026	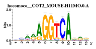	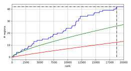	link	link	PWMs
398	☐ cisbp__M1644<br>Description: M1644	3.18043	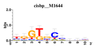	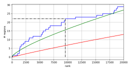	link	link	PWMs
399	☐ hdpi__MCTP2<br>Description: MCTP2<br>Possible TFs: MCTP2	3.17850	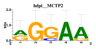	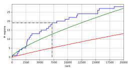	link	link	PWMs
400	☐ hocomoco__ESR2_HUMAN.H11MO.1.A<br>Description: ESR2_HUMAN<br>Possible TFs: ESR2	3.16979	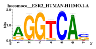	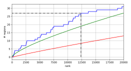	link	link	PWMs
401	☐ cisbp__M1965<br>Description: CG11152[gene ID: "FBgn0039937" species: "Drosophila melanogaster" TF status: "inferred" TF family: "Forkhead" DBDs: "Fork_head"]; CG9571[gene ID: "FBgn0031086" species: "Drosophila melanogaster" TF status: "inferred" TF family: "Forkhead" DBDs: "Fork_head"]; FOXA1[gene ID: "ENSG00000129514" species: "Homo sapiens" TF status: "direct" TF family: "Forkhead" DBDs: "Fork_head"]; FOXB2[gene ID: "ENSG00000204612" species: "Homo sapiens" TF status: "inferred" TF family: "Forkhead" DBDs: "Fork_head"]; FOXD4L1[gene ID: "ENSG00000184492" species: "Homo sapiens"	3.16882	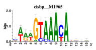	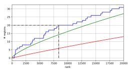	link	link	PWMs

#	Feature	NES	Logo	Recovery Curve	Candidate targets	All regions in top 20000	Database
	TF status: "inferred" TF family: "Forkhead" DBDs: "Fork_head"]; FOXD4L3[gene ID: "ENSG00000187559" species: "Homo sapiens" TF status: "inferred" TF family: "Forkhead" DBDs: "Fork_head"]; FOXD4L5[gene ID: "ENSG00000204779" species: "Homo sapiens" TF status: "inferred" TF family: "Forkhead" DBDs: "Fork_head"]; FOXD4L6[gene ID: "ENSG00000204793" species: "Homo sapiens" TF status: "inferred" TF family: "Forkhead" DBDs: "Fork_head"]; FOXD4[gene ID: "ENSG00000170122" species: "Homo sapiens" TF status: "inferred" TF family: "Forkhead" DBDs: "Fork_head"]; FOXE1[gene ID: "ENSG00000178919" species: "Homo sapiens" TF status: "inferred" TF family: "Forkhead" DBDs: "Fork_head"]; FOXE3[gene ID: "ENSG00000186790" species: "Homo sapiens" TF status: "inferred" TF family: "Forkhead" DBDs: "Fork_head"]; FOXI2[gene ID: "ENSG00000186766" species: "Homo sapiens" TF status: "inferred" TF family: "Forkhead" DBDs: "Fork_head"]; FOXK2[gene ID: "ENSG00000141568" species: "Homo sapiens" TF status: "inferred" TF family: "Forkhead" DBDs: "Fork_head"]; FOXS1[gene ID: "ENSG00000179772" species: "Homo sapiens" TF status: "inferred" TF family: "Forkhead" DBDs: "Fork_head"]; Foxa1[gene ID: "ENSMUSG00000035451" species: "Mus musculus" TF status: "inferred" TF family: "Forkhead" DBDs: "Fork_head"]; Foxa3[gene ID: "ENSMUSG00000040891" species: "Mus musculus" TF status: "inferred" TF family: "Forkhead" DBDs: "Fork_head"]; Foxb2[gene ID: "ENSMUSG00000056829" species: "Mus musculus" TF status: "inferred" TF family: "Forkhead" DBDs: "Fork_head"]; Foxd2[gene ID: "ENSMUSG00000055210" species: "Mus musculus" TF status: "inferred" TF family: "Forkhead" DBDs: "Fork_head"]; Foxd3[gene ID: "ENSMUSG00000067261" species: "Mus musculus" TF status: "inferred" TF family: "Forkhead" DBDs: "Fork_head"]; Foxd4[gene ID: "ENSMUSG00000051490" species: "Mus musculus" TF status: "inferred" TF family: "Forkhead" DBDs: "Fork_head"]; Foxe1[gene ID: "ENSMUSG00000070990" species: "Mus musculus" TF status: "inferred" TF family: "Forkhead" DBDs: "Fork_head"]; Foxe3[gene ID: "ENSMUSG00000044518" species: "Mus musculus" TF status: "inferred" TF family: "Forkhead" DBDs: "Fork_head"]; Foxf1a[gene ID: "ENSMUSG00000042812" species: "Mus musculus" TF status: "inferred" TF family: "Forkhead" DBDs: "Fork_head"]; Foxf2[gene ID: "ENSMUSG00000038402" species: "Mus musculus" TF status: "inferred" TF family: "Forkhead" DBDs: "Fork_head"]; Foxi1[gene ID: "ENSMUSG00000047861" species: "Mus musculus" TF status: "inferred" TF family: "Forkhead" DBDs: "Fork_head"]; Foxi2[gene ID: "ENSMUSG00000048377" species: "Mus musculus" TF status: "inferred" TF family: "Forkhead" DBDs: "Fork_head"]; Foxi3[gene ID: "ENSMUSG00000055874" species: "Mus musculus" TF status: "inferred" TF family: "Forkhead" DBDs: "Fork_head"]; Foxk2[gene ID: "ENSMUSG00000039275" species: "Mus musculus" TF status: "inferred" TF family: "Forkhead" DBDs: "Fork_head"]; Foxl2[gene ID: "ENSMUSG00000050397" species: "Mus musculus" TF status: "inferred" TF family: "Forkhead" DBDs: "Fork_head"]; Foxq1[gene ID: "ENSMUSG00000038415" species: "Mus musculus" TF status: "inferred" TF family: "Forkhead" DBDs: "Fork_head"]; Foxs1[gene ID: "ENSMUSG00000074676" species: "Mus musculus" TF status: "inferred" TF family: "Forkhead" DBDs: "Fork_head"]; KIAA0415[gene ID: "ENSG00000242802" species: "Homo sapiens" TF status: "inferred" TF family: "Forkhead" DBDs: "Fork_head"]; Mnf[gene ID: "FBgn0036134" species: "Drosophila melanogaster" TF status: "inferred" TF family: "Forkhead" DBDs: "Fork_head"]; fd59A[gene ID: "FBgn0004896" species: "Drosophila melanogaster" TF status: "inferred" TF family: "Forkhead" DBDs: "Fork_head"]; fd96Ca[gene ID: "FBgn0004897" species: "Drosophila melanogaster" TF status: "inferred" TF family: "Forkhead" DBDs: "Fork_head"]; fd96Cb[gene ID: "FBgn0004898" species: "Drosophila melanogaster" TF status: "inferred" TF family: "Forkhead" DBDs: "Fork_head"]<br>Possible TFs: FOXA1						
402	☐ transfac_pro__M09244<br>Description: P$ATHB6_02: ATHB6	*3.16649*	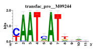	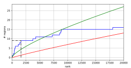	link	link	PWMs
403	☐ transfac_pro__M01161<br>Description: P$ASR1_01: ASR-1	*3.15980*	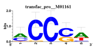	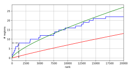	link	link	PWMs

#	Feature	NES	Logo	Recovery Curve	Candidate targets	All regions in top 20000	Database
404	☐ transfac_pro__M07733<br>Description: I$SRP_01: srp<br>Possible TFs: GATA6, GATA4, GATA5	3.15601	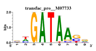	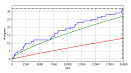	link	link	PWMs
405	☐ transfac_pro__M02111<br>Description: V$NR1B2_Q6: NR1B2<br>Possible TFs: RARB	3.15391	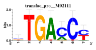	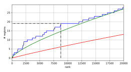	link	link	PWMs
406	☐ ENCFF001UHI<br>Description: RXRA ChIP-seq protocol PCR1x on human HepG2	3.15130		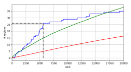	link	link	TF binding sites
407	☐ ENCFF001WYD<br>Description: H3K27me3 ChIP-seq on human GM12878	3.14759		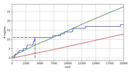	link	link	Histone modifications
408	☐ tfdimers__MD00248<br>Description: M00271_forward_1_M00790_reverse dimer: AML1a / HNF1<br>Possible TFs: HNF1B, HNF1A, RUNX1	3.14295	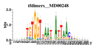	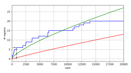	link	link	PWMs
409	☐ dbcorrdb__MYC__ENCSR000DKU_1__m1<br>Description: MYC (ENCSR000DKU-1, motif 1)<br>Possible TFs: MYC	3.13876	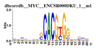	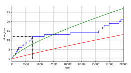	link	link	PWMs
410	☐ transfac_pro__M07285<br>Description: V$ESE1_Q6: ESE-1<br>Possible TFs: ELF3	3.13747	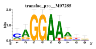	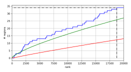	link	link	PWMs
411	☐ tfdimers__MD00461<br>Description: M01721_reverse_10_M01721_forward dimer: PUR1 / PUR1<br>Possible TFs: PURA	3.13127	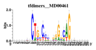	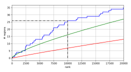	link	link	PWMs
412	☐ elemento__CCCTGCC<br>Description: Conserved regulatory element CCCTGCC between Hs and Mm	3.12958	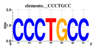	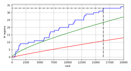	link	link	PWMs
413	☐ cisbp__M0505<br>Description: NRG1[gene ID: "YDR043C" species: "Saccharomyces cerevisiae" TF status: "direct" TF family: "C2H2 ZF" DBDs: "zf-C2H2"]	3.12780	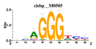	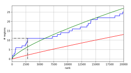	link	link	PWMs
414	☐ dbcorrdb__MYBL2__ENCSR000BRO_1__m1<br>Description: MYBL2 (ENCSR000BRO-1, motif 1)<br>Possible TFs: MYBL2	3.12506	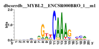	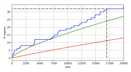	link	link	PWMs
415	☐ E098-H3K27ac<br>Description: H3K27ac in Pancreas (E098,)	3.12190		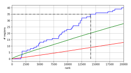	link	link	Histone modifications
416	☐ hocomoco__ZFHX3_HUMAN.H11MO.0.D<br>Description: ZFHX3_HUMAN<br>Possible TFs: ZFHX3	3.10846	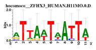	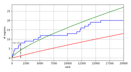	link	link	PWMs
417	☐ hocomoco__FOXA2_MOUSE.H11MO.0.A<br>Description: FOXA2_MOUSE<br>Possible TFs: FOXA2	3.10089	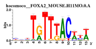	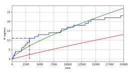	link	link	PWMs
418	☐ homer__GGTCANYTGAGGWCA_THRa<br>Description: THRa(NR)/C17.2-THRa-ChIP-Seq(GSE38347)/Homer<br>Possible TFs: THRA	3.09790	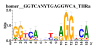	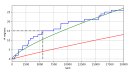	link	link	PWMs
419	☐ cisbp__M1963<br>Description: ZFY[gene ID: "ENSG00000067646" species: "Homo sapiens" TF status: "inferred" TF family: "C2H2 ZF" DBDs: "zf-C2H2"]; Zfa[gene ID: "ENSMUSG00000049576" species: "Mus musculus" TF status: "inferred" TF family: "C2H2 ZF" DBDs: "zf-C2H2"]; Zfx[gene ID: "ENSMUSG00000079509" species: "Mus musculus" TF status: "direct" TF family: "C2H2 ZF" DBDs: "zf-C2H2"]; Zfy1[gene ID: "ENSMUSG00000053211" species: "Mus musculus" TF status: "inferred" TF family: "C2H2 ZF" DBDs: "zf-C2H2"]<br>Possible TFs: ZFY	3.09718	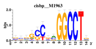	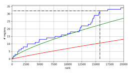	link	link	PWMs
420	☐ homer__AGGTCANTGACCTN_FXR<br>Description: FXR(NR),IR1/Liver-FXR-ChIP-Seq(Chong et al.)/Homer<br>Possible TFs: NR1H4	3.09677	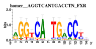	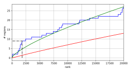	link	link	PWMs
421	☐<br>taipale_tf_pairs__TFAP2C_ETV7_SCCNNNGGSNNNGGAAGNNNTTCCNN_CAP_repr<br>Description: TFAP2C [AP2] - ETV7 [ETS]<br>Possible TFs: TFAP2C, ETV7	3.09452	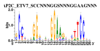	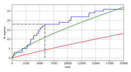	link	link	PWMs

#	Feature	NES	Logo	Recovery Curve	Candidate targets	All regions in top 20000	Database
422	☐ hocomoco__RXRA_MOUSE.H11MO.1.A<br>Description: RXRA_MOUSE<br>Possible TFs: RXRA	*3.07953*	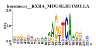	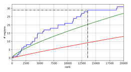	**link**	**link**	PWMs
423	☐ jaspar__MA0146.2<br>Description: Zfx<br>Possible TFs: ZFY	*3.07864*	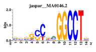	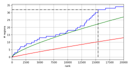	**link**	**link**	PWMs
424	☐ cisbp__M0539<br>Description: USV1[gene ID: "YPL230W" species: "Saccharomyces cerevisiae" TF status: "direct" TF family: "C2H2 ZF" DBDs: "zf-C2H2"]	*3.07558*	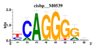	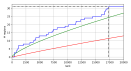	**link**	**link**	PWMs
425	☐ transfac_pro__M01261<br>Description: V$HNF3A_01: HNF3A<br>Possible TFs: FOXA1	*3.07268*	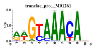	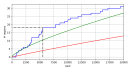	**link**	**link**	PWMs
426	☐ transfac_pro__M04816<br>Description: V$BCL3_03: BCL-3<br>Possible TFs: BCL3	*3.06897*	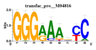	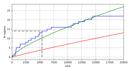	**link**	**link**	PWMs
427	☐ neph__UW.Motif.0169<br>Description: cccaNNNNcagg	*3.06091*	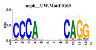	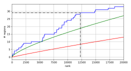	**link**	**link**	PWMs
428	☐ yetfasco__YDR043C_2148<br>Description: YDR043C_2148	*3.05108*	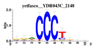	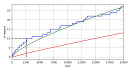	**link**	**link**	PWMs
429	☐ cisbp__M1422<br>Description: Rorc[gene ID: "ENSMUSG00000028150" species: "Mus musculus" TF status: "inferred" TF family: "Nuclear receptor" DBDs: "zf-C4"]	*3.05052*	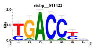	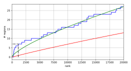	**link**	**link**	PWMs
430	☐ transfac_pro__M03846<br>Description: V$SMAD5_Q5: SMAD5<br>Possible TFs: SMAD5	*3.04915*	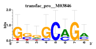	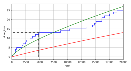	**link**	**link**	PWMs
431	☐ transfac_pro__M09249<br>Description: P$HAT22_01: HAT22	*3.03883*	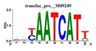	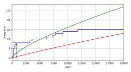	**link**	**link**	PWMs
432	☐ E055-H3K4me1-broadpeak<br>Description: H3K4me1 in Foreskin Fibroblast Primary Cells skin01 (E055, broadpeak)	*3.03880*		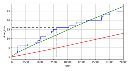	**link**	**link**	Histone modifications
433	☐ hocomoco__NFIC_MOUSE.H11MO.1.A<br>Description: NFIC_MOUSE<br>Possible TFs: NFIC	*3.03424*	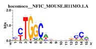	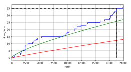	**link**	**link**	PWMs
434	☐ transfac_pro__M01619<br>Description: F$DAL80_01: Dal80p<br>Possible TFs: TRPS1, GATA6, GATA4, GATA5, GATA2, GATA3, GATA1	*3.02698*	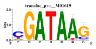	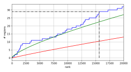	**link**	**link**	PWMs
435	☐ cisbp__M1934<br>Description: ESR1[gene ID: "ENSG00000091831" species: "Homo sapiens" TF status: "direct" TF family: "Nuclear receptor" DBDs: "zf-C4"]; Esr2[gene ID: "ENSMUSG00000021055" species: "Mus musculus" TF status: "inferred" TF family: "Nuclear receptor" DBDs: "zf-C4"]<br>Possible TFs: ESR1	*3.02682*	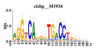	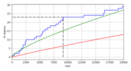	**link**	**link**	PWMs
436	☐ hocomoco__PPARA_HUMAN.H11MO.1.B<br>Description: PPARA_HUMAN<br>Possible TFs: PPARA	*3.02658*	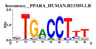	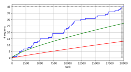	**link**	**link**	PWMs
437	☐ jaspar__MA0289.1<br>Description: DAL80<br>Possible TFs: TRPS1, GATA6, GATA4, GATA5, GATA2, GATA3, GATA1	*3.02593*	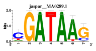	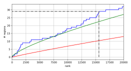	**link**	**link**	PWMs
438	☐ transfac_public__M00009<br>Description: I$TTK69_01: Ttk 69K	*3.01675*	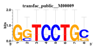	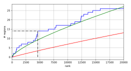	**link**	**link**	PWMs
439	☐ transfac_pro__M01177<br>Description: V$SREBP2_Q6: SREBP-2<br>Possible TFs: SREBF2	*3.00224*	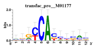	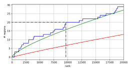	**link**	**link**	PWMs
440	☐ E036-H3K4me1-broadpeak<br>Description: H3K4me1 in Primary hematopoietic stem cells short term culture (E036, broadpeak)	*3.00080*		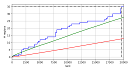	**link**	**link**	Histone modifications

© 2015-2019 **KULeuven** | Authors: Gert Hulselmans & Hana Imrichova | Web design (CSS) by **Andreas Viklund**.
