# Supplementary material for: Chromatin accessibility and regulatory vocabulary across indicine cattle tissues
Source: Genome Biol. 2021 Sep 21;22:273. doi: 10.1186/s13059-021-02489-7 (PMC8454054; doi:10.1186/s13059-021-02489-7)
Supplement: Supplementary file 12 — Additional file 12. Enriched regulatory features in muscle-specific peaks according to i-cisTarget online tool [68]. [file 13059_2021_2489_MOESM12_ESM.pdf]

i-cisTarget

An integrative genomics method for the prediction of regulatory features and cis-regulatory modules.

Parameters and statistics for muscle

Number of features	29890
Number of enriched features (NES > 3.0)	459
Total number of ranked regions	1223024
Type of input query	bed
Number of **i-cisTarget regions in input set**	9197 (**Results of the region mapping**)
Minimum fraction of overlap	0.4
Normalized enrichment score (NES) threshold	3.0
AUC threshold (fraction / # of ranked regions)	0.005 (6115)
Recovery curve threshold (# of regions)	20000

AUC distribution

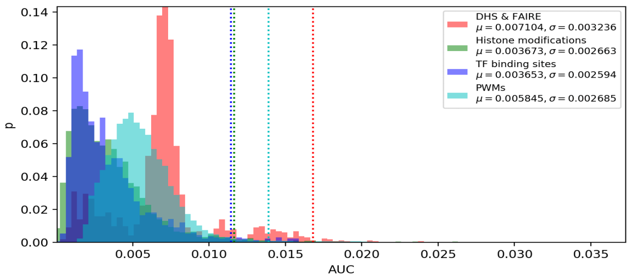

Recovery of best feature

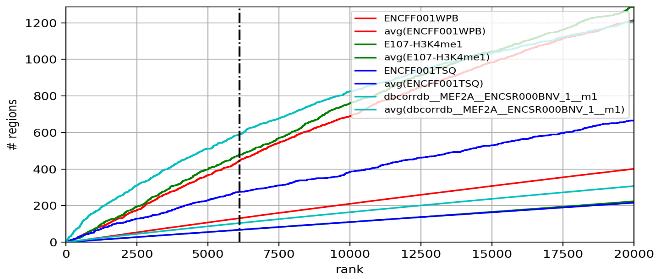

Results for muscle

Select features in the table below, select an operation and proceed.

1. Use candidate target regions as **filter** and use as input for i-cisTarget again.
2. **Scan** candidate target regions of selected features for multiple homotypic CRMs.
3. **Create SIF file** for the selected features.

This report is also available as an **archive**.

#	Feature	NES	Logo	Recovery Curve	Candidate targets	All regions in top 20000	Database
1	dbcorrdb__MEF2A__ENCSR000BNV_1__m1<br>Description: MEF2A (ENCSR000BNV-1, motif 1)<br>Possible TFs: MEF2A	*11.70740*	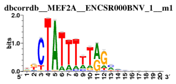	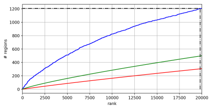	link	link	PWMs
2	yetfasco__YPL089C_419<br>Description: YPL089C_419<br>Possible TFs: MEF2C, MEF2B, MEF2A, BORCS8-MEF2B, MEF2D	*11.63170*	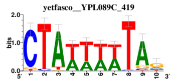	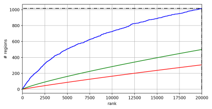	link	link	PWMs
3	hocomoco__MEF2D_MOUSE.H11MO.0.A<br>Description: MEF2D_MOUSE<br>Possible TFs: MEF2D	*11.60521*	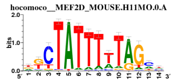	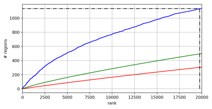	link	link	PWMs
4	hocomoco__MEF2D_HUMAN.H11MO.0.A<br>Description: MEF2D_HUMAN<br>Possible TFs: MEF2D	*11.29151*	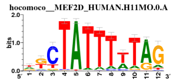	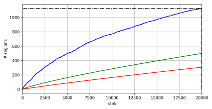	link	link	PWMs

#	Feature	NES	Logo	Recovery Curve	Candidate targets	All regions in top 20000	Database
5	☐ cisbp__M1884<br>Description: AC002126.6[gene ID: "ENSG00000064489" species: "Homo sapiens" TF status: "inferred" TF family: "MADS box" DBDs: "SRF-TF"]; MEF2A[gene ID: "ENSG00000068305" species: "Homo sapiens" TF status: "direct" TF family: "MADS box" DBDs: "SRF-TF"]; Mef2a[gene ID: "ENSMUSG00000030557" species: "Mus musculus" TF status: "inferred" TF family: "MADS box" DBDs: "SRF-TF"]; Mef2c[gene ID: "ENSMUSG00000005583" species: "Mus musculus" TF status: "inferred" TF family: "MADS box" DBDs: "SRF-TF"]; Mef2d[gene ID: "ENSMUSG00000001419" species: "Mus musculus" TF status: "inferred" TF family: "MADS box" DBDs: "SRF-TF"]<br>Possible TFs: MEF2A	*11.11669*	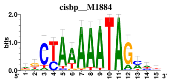	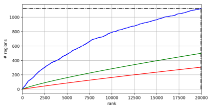	**link**	**link**	PWMs
6	☐ transfac_pro__M04758<br>Description: V$MEF2C_02: MEF-2C<br>Possible TFs: MEF2C	*11.06075*	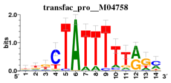	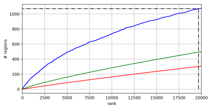	**link**	**link**	PWMs
7	☐ cisbp__M6340<br>Description: AC002126.6[gene ID: "ENSG00000064489" species: "Homo sapiens" TF status: "inferred" TF family: "MADS box" DBDs: "SRF-TF"]; MEF2A[gene ID: "ENSG00000068305" species: "Homo sapiens" TF status: "direct" TF family: "MADS box" DBDs: "SRF-TF"]; Mef2a[gene ID: "ENSMUSG00000030557" species: "Mus musculus" TF status: "inferred" TF family: "MADS box" DBDs: "SRF-TF"]; Mef2c[gene ID: "ENSMUSG00000005583" species: "Mus musculus" TF status: "inferred" TF family: "MADS box" DBDs: "SRF-TF"]; Mef2d[gene ID: "ENSMUSG00000001419" species: "Mus musculus" TF status: "inferred" TF family: "MADS box" DBDs: "SRF-TF"]<br>Possible TFs: MEF2A	*11.03602*	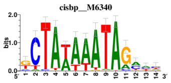	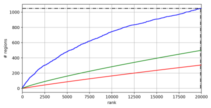	**link**	**link**	PWMs
8	☐ hocomoco__MEF2C_HUMAN.H11MO.0.A<br>Description: MEF2C_HUMAN<br>Possible TFs: MEF2C	*11.03301*	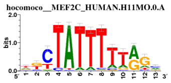	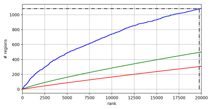	**link**	**link**	PWMs
9	☐ transfac_pro__M07218<br>Description: V$MEF2A_06: MEF-2A<br>Possible TFs: MEF2A	*10.95322*	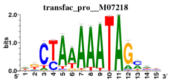	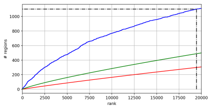	**link**	**link**	PWMs
10	☐ cisbp__M4467<br>Description: AC002126.6[gene ID: "ENSG00000064489" species: "Homo sapiens" TF status: "inferred" TF family: "MADS box" DBDs: "SRF-TF"]; MEF2C[gene ID: "ENSG00000081189" species: "Homo sapiens" TF status: "direct" TF family: "MADS box" DBDs: "SRF-TF"]; Mef2a[gene ID: "ENSMUSG00000030557" species: "Mus musculus" TF status: "inferred" TF family: "MADS box" DBDs: "SRF-TF"]; Mef2c[gene ID: "ENSMUSG00000005583" species: "Mus musculus" TF status: "inferred" TF family: "MADS box" DBDs: "SRF-TF"]; Mef2d[gene ID: "ENSMUSG00000001419" species: "Mus musculus" TF status: "inferred" TF family: "MADS box" DBDs: "SRF-TF"]<br>Possible TFs: MEF2C	*10.84565*	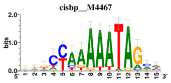	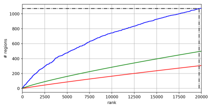	**link**	**link**	PWMs
11	☐ homer__DCYAAAAATAGM_Mef2c<br>Description: Mef2c(MADS)/GM12878-Mef2c-ChIP-Seq(GSE32465)/Homer<br>Possible TFs: MEF2C	*10.75640*	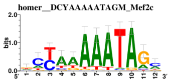	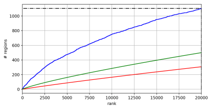	**link**	**link**	PWMs

#	Feature	NES	Logo	Recovery Curve	Candidate targets	All regions in top 20000	Database
12	☐ cisbp__M4675<br>Description: AC002126.6[gene ID: "ENSG00000064489" species: "Homo sapiens" TF status: "inferred" TF family: "MADS box" DBDs: "SRF-TF"]; Mef2[gene ID: "FBgn0011656" species: "Drosophila melanogaster" TF status: "direct" TF family: "MADS box" DBDs: "SRF-TF"]; Mef2a[gene ID: "ENSMUSG00000030557" species: "Mus musculus" TF status: "inferred" TF family: "MADS box" DBDs: "SRF-TF"]; Mef2c[gene ID: "ENSMUSG00000005583" species: "Mus musculus" TF status: "inferred" TF family: "MADS box" DBDs: "SRF-TF"]; Mef2d[gene ID: "ENSMUSG00000001419" species: "Mus musculus" TF status: "inferred" TF family: "MADS box" DBDs: "SRF-TF"]<br>Possible TFs: MEF2C, MEF2B, MEF2A, BORCS8-MEF2B, MEF2D	*10.75480*	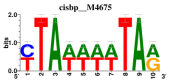	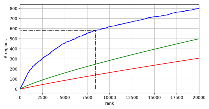	link	link	PWMs
13	☐ transfac_pro__M07424<br>Description: V$MEF2_Q6_03: MEF-2<br>Possible TFs: MYEF2, MEF2C, MEF2B, MEF2A, BORCS8-MEF2B, MEF2D	*10.75447*	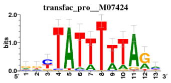	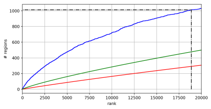	link	link	PWMs
14	☐ cisbp__M4466<br>Description: AC002126.6[gene ID: "ENSG00000064489" species: "Homo sapiens" TF status: "inferred" TF family: "MADS box" DBDs: "SRF-TF"]; MEF2A[gene ID: "ENSG00000068305" species: "Homo sapiens" TF status: "direct" TF family: "MADS box" DBDs: "SRF-TF"]; Mef2a[gene ID: "ENSMUSG00000030557" species: "Mus musculus" TF status: "inferred" TF family: "MADS box" DBDs: "SRF-TF"]; Mef2c[gene ID: "ENSMUSG00000005583" species: "Mus musculus" TF status: "inferred" TF family: "MADS box" DBDs: "SRF-TF"]; Mef2d[gene ID: "ENSMUSG00000001419" species: "Mus musculus" TF status: "inferred" TF family: "MADS box" DBDs: "SRF-TF"]<br>Possible TFs: MEF2A	*10.72265*	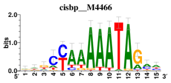	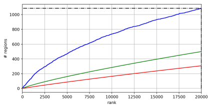	link	link	PWMs
15	☐ hocomoco__MEF2A_HUMAN.H11MO.0.A<br>Description: MEF2A_HUMAN<br>Possible TFs: MEF2A	*10.70925*	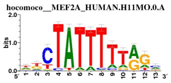	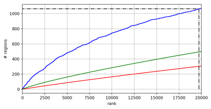	link	link	PWMs
16	☐ factorbook__MEF2<br>Description: MEF2<br>Possible TFs: MEF2C, MEF2A	*10.59823*	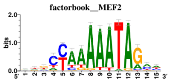	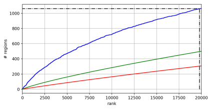	link	link	PWMs
17	☐ hocomoco__MEF2C_MOUSE.H11MO.0.A<br>Description: MEF2C_MOUSE<br>Possible TFs: MEF2C	*10.52692*	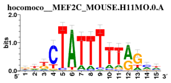	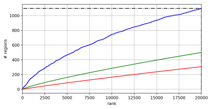	link	link	PWMs
18	☐ transfac_pro__M07326<br>Description: V$MEF2_Q6_02: Mef-2A<br>Possible TFs: MEF2A	*10.52051*	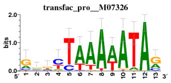	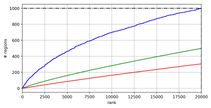	link	link	PWMs
19	☐ dbcorrdb__MEF2A__ENCSR000BKB_1__m1<br>Description: MEF2A (ENCSR000BKB-1, motif 1)<br>Possible TFs: MEF2A	*10.51387*	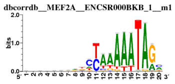	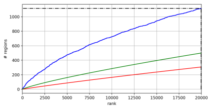	link	link	PWMs
20	☐ transfac_public__M00231<br>Description: V$MEF2_02: MEF-2A<br>Possible TFs: MEF2A	*10.46868*	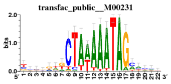	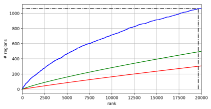	link	link	PWMs

#	Feature	NES	Logo	Recovery Curve	Candidate targets	All regions in top 20000	Database
21	☐ cisbp__M3551<br>Description: AC002126.6[gene ID: "ENSG00000064489" species: "Homo sapiens" TF status: "inferred" TF family: "MADS box" DBDs: "SRF-TF"]; MEF2A[gene ID: "ENSG00000068305" species: "Homo sapiens" TF status: "direct" TF family: "MADS box" DBDs: "SRF-TF"]; Mef2a[gene ID: "ENSMUSG00000030557" species: "Mus musculus" TF status: "inferred" TF family: "MADS box" DBDs: "SRF-TF"]; Mef2c[gene ID: "ENSMUSG00000005583" species: "Mus musculus" TF status: "inferred" TF family: "MADS box" DBDs: "SRF-TF"]; Mef2d[gene ID: "ENSMUSG00000001419" species: "Mus musculus" TF status: "inferred" TF family: "MADS box" DBDs: "SRF-TF"]<br>Possible TFs: MEF2A	*10.46730*	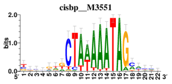	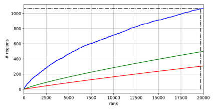	**link**	**link**	PWMs
22	☐ stark__YTAWWWWTAR<br>Description: Myocyte enhancing factor 2<br>Possible TFs: MEF2C, MEF2B, MEF2A, BORCS8-MEF2B, MEF2D	*10.40636*	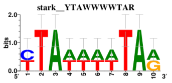	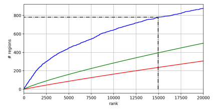	**link**	**link**	PWMs
23	☐ cisbp__M6342<br>Description: AC002126.6[gene ID: "ENSG00000064489" species: "Homo sapiens" TF status: "inferred" TF family: "MADS box" DBDs: "SRF-TF"]; MEF2D[gene ID: "ENSG00000116604" species: "Homo sapiens" TF status: "direct" TF family: "MADS box" DBDs: "SRF-TF"]; Mef2a[gene ID: "ENSMUSG00000030557" species: "Mus musculus" TF status: "inferred" TF family: "MADS box" DBDs: "SRF-TF"]; Mef2c[gene ID: "ENSMUSG00000005583" species: "Mus musculus" TF status: "inferred" TF family: "MADS box" DBDs: "SRF-TF"]; Mef2d[gene ID: "ENSMUSG00000001419" species: "Mus musculus" TF status: "inferred" TF family: "MADS box" DBDs: "SRF-TF"]<br>Possible TFs: MEF2D	*10.33123*	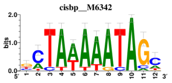	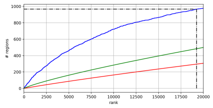	**link**	**link**	PWMs
24	☐ dbcorrdb__MEF2C__ENCSR000BNG_1__m1<br>Description: MEF2C (ENCSR000BNG-1, motif 1)<br>Possible TFs: MEF2C	*10.22563*	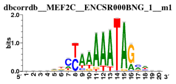	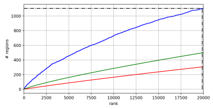	**link**	**link**	PWMs
25	☐ cisbp__M5615<br>Description: AC002126.6[gene ID: "ENSG00000064489" species: "Homo sapiens" TF status: "inferred" TF family: "MADS box" DBDs: "SRF-TF"]; MEF2A[gene ID: "ENSG00000068305" species: "Homo sapiens" TF status: "direct" TF family: "MADS box" DBDs: "SRF-TF"]; Mef2a[gene ID: "ENSMUSG00000030557" species: "Mus musculus" TF status: "inferred" TF family: "MADS box" DBDs: "SRF-TF"]; Mef2c[gene ID: "ENSMUSG00000005583" species: "Mus musculus" TF status: "inferred" TF family: "MADS box" DBDs: "SRF-TF"]; Mef2d[gene ID: "ENSMUSG00000001419" species: "Mus musculus" TF status: "inferred" TF family: "MADS box" DBDs: "SRF-TF"]<br>Possible TFs: MEF2A	*10.08671*	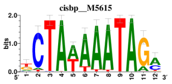	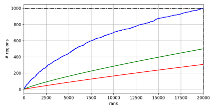	**link**	**link**	PWMs
26	☐ hocomoco__MEF2A_MOUSE.H11MO.0.A<br>Description: MEF2A_MOUSE<br>Possible TFs: MEF2A	*10.08578*	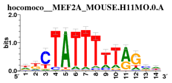	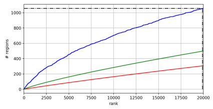	**link**	**link**	PWMs
27	☐ scertf__spivak.RLM1<br>Description: spivak.RLM1<br>Possible TFs: MEF2C, MEF2B, MEF2A, BORCS8-MEF2B, MEF2D	*9.95288*	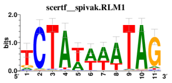	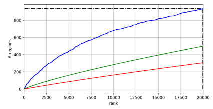	**link**	**link**	PWMs
28	☐ swissregulon__hs__MEF2_A_B_C_D_.p2<br>Description: hs__MEF2{A,B,C,D}.p2<br>Possible TFs: MEF2C, MEF2B, MEF2A, MEF2D	*9.84009*	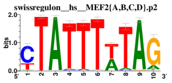	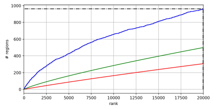	**link**	**link**	PWMs

#	Feature	NES	Logo	Recovery Curve	Candidate targets	All regions in top 20000	Database
29	☐ taipale__MEF2A_DBD_KCTAWAAATAGM_repr<br>Description: MEF2A dimeric MADS DBD<br>Possible TFs: MEF2A	*9.64210*	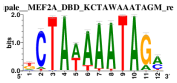	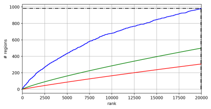	**link**	**link**	PWMs
30	☐ cisbp__M2297<br>Description: AC002126.6[gene ID: "ENSG00000064489" species: "Homo sapiens" TF status: "inferred" TF family: "MADS box" DBDs: "SRF-TF"]; MEF2C[gene ID: "ENSG00000081189" species: "Homo sapiens" TF status: "direct" TF family: "MADS box" DBDs: "SRF-TF"]; Mef2a[gene ID: "ENSMUSG00000030557" species: "Mus musculus" TF status: "inferred" TF family: "MADS box" DBDs: "SRF-TF"]; Mef2c[gene ID: "ENSMUSG00000005583" species: "Mus musculus" TF status: "inferred" TF family: "MADS box" DBDs: "SRF-TF"]; Mef2d[gene ID: "ENSMUSG00000001419" species: "Mus musculus" TF status: "inferred" TF family: "MADS box" DBDs: "SRF-TF"]<br>Possible TFs: MEF2C	*9.49181*	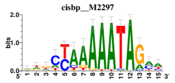	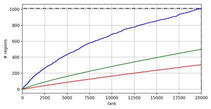	**link**	**link**	PWMs
31	☐ jaspar__MA0497.1<br>Description: MEF2C<br>Possible TFs: MEF2C	*9.48010*	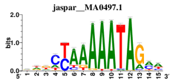	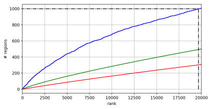	**link**	**link**	PWMs
32	☐ homer__CCAAAAATAG_Mef2a<br>Description: Mef2a(MADS)/HL1-Mef2a.biotin-ChIP-Seq(GSE21529)/Homer<br>Possible TFs: MEF2A	*9.39804*	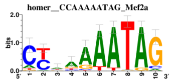	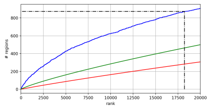	**link**	**link**	PWMs
33	☐ transfac_public__M00026<br>Description: V$RSRFC4_01: RSRFC4<br>Possible TFs: MEF2A	*9.37305*	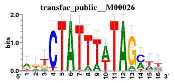	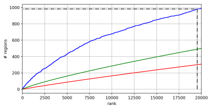	**link**	**link**	PWMs
34	☐ hocomoco__MEF2B_HUMAN.H11MO.0.A<br>Description: MEF2B_HUMAN<br>Possible TFs: MEF2B	*9.16224*	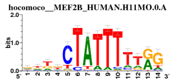	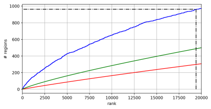	**link**	**link**	PWMs
35	☐ cisbp__M4694<br>Description: AC002126.6[gene ID: "ENSG00000064489" species: "Homo sapiens" TF status: "inferred" TF family: "MADS box" DBDs: "SRF-TF"]; MEF2A[gene ID: "ENSG00000068305" species: "Homo sapiens" TF status: "direct" TF family: "MADS box" DBDs: "SRF-TF"]; Mef2a[gene ID: "ENSMUSG00000030557" species: "Mus musculus" TF status: "inferred" TF family: "MADS box" DBDs: "SRF-TF"]; Mef2c[gene ID: "ENSMUSG00000005583" species: "Mus musculus" TF status: "inferred" TF family: "MADS box" DBDs: "SRF-TF"]; Mef2d[gene ID: "ENSMUSG00000001419" species: "Mus musculus" TF status: "inferred" TF family: "MADS box" DBDs: "SRF-TF"]<br>Possible TFs: MEF2A	*9.12250*	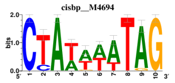	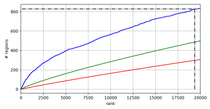	**link**	**link**	PWMs
36	☐ cisbp__M5616<br>Description: AC002126.6[gene ID: "ENSG00000064489" species: "Homo sapiens" TF status: "inferred" TF family: "MADS box" DBDs: "SRF-TF"]; MEF2B[gene ID: "ENSG00000213999" species: "Homo sapiens" TF status: "direct" TF family: "MADS box" DBDs: "SRF-TF"]; Mef2a[gene ID: "ENSMUSG00000030557" species: "Mus musculus" TF status: "inferred" TF family: "MADS box" DBDs: "SRF-TF"]; Mef2c[gene ID: "ENSMUSG00000005583" species: "Mus musculus" TF status: "inferred" TF family: "MADS box" DBDs: "SRF-TF"]; Mef2d[gene ID: "ENSMUSG00000001419" species: "Mus musculus" TF status: "inferred" TF family: "MADS box" DBDs: "SRF-TF"]<br>Possible TFs: MEF2B	*8.94367*	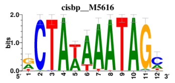	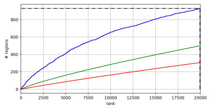	**link**	**link**	PWMs

#	Feature	NES	Logo	Recovery Curve	Candidate targets	All regions in top 20000	Database
37	☐ transfac_pro__M00407<br>Description: V$RSRFC4_Q2: RSRFC4<br>Possible TFs: MEF2A	*8.93677*	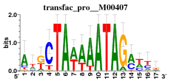	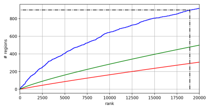	**link**	**link**	PWMs
38	☐ cisbp__M3846<br>Description: AC002126.6[gene ID: "ENSG00000064489" species: "Homo sapiens" TF status: "inferred" TF family: "MADS box" DBDs: "SRF-TF"]; MEF2A[gene ID: "ENSG00000068305" species: "Homo sapiens" TF status: "direct" TF family: "MADS box" DBDs: "SRF-TF"]; Mef2a[gene ID: "ENSMUSG00000030557" species: "Mus musculus" TF status: "inferred" TF family: "MADS box" DBDs: "SRF-TF"]; Mef2c[gene ID: "ENSMUSG00000005583" species: "Mus musculus" TF status: "inferred" TF family: "MADS box" DBDs: "SRF-TF"]; Mef2d[gene ID: "ENSMUSG00000001419" species: "Mus musculus" TF status: "inferred" TF family: "MADS box" DBDs: "SRF-TF"]<br>Possible TFs: MEF2A	*8.93221*	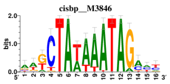	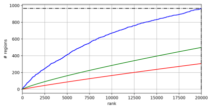	**link**	**link**	PWMs
39	☐ transfac_pro__M00941<br>Description: V$MEF2_Q6_01: MEF-2<br>Possible TFs: MEF2C, MEF2A, MEF2D	*8.84459*	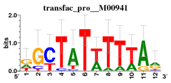	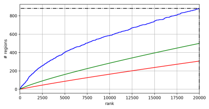	**link**	**link**	PWMs
40	☐ transfac_pro__M01301<br>Description: V$MEF2A_05: MEF-2A<br>Possible TFs: MEF2A	*8.80407*	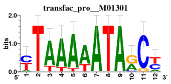	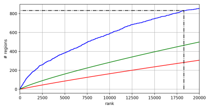	**link**	**link**	PWMs
41	☐ cisbp__M5617<br>Description: AC002126.6[gene ID: "ENSG00000064489" species: "Homo sapiens" TF status: "inferred" TF family: "MADS box" DBDs: "SRF-TF"]; MEF2D[gene ID: "ENSG00000116604" species: "Homo sapiens" TF status: "direct" TF family: "MADS box" DBDs: "SRF-TF"]; Mef2a[gene ID: "ENSMUSG00000030557" species: "Mus musculus" TF status: "inferred" TF family: "MADS box" DBDs: "SRF-TF"]; Mef2c[gene ID: "ENSMUSG00000005583" species: "Mus musculus" TF status: "inferred" TF family: "MADS box" DBDs: "SRF-TF"]; Mef2d[gene ID: "ENSMUSG00000001419" species: "Mus musculus" TF status: "inferred" TF family: "MADS box" DBDs: "SRF-TF"]<br>Possible TFs: MEF2D	*8.79135*	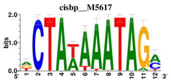	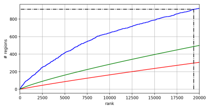	**link**	**link**	PWMs
42	☐ taipale__MEF2B_full_RCTAWAAATAGM<br>Description: MEF2B dimeric MADS full<br>Possible TFs: MEF2B	*8.70120*	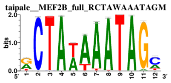	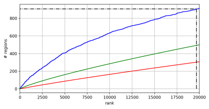	**link**	**link**	PWMs
43	☐ cisbp__M6341<br>Description: AC002126.6[gene ID: "ENSG00000064489" species: "Homo sapiens" TF status: "inferred" TF family: "MADS box" DBDs: "SRF-TF"]; MEF2C[gene ID: "ENSG00000081189" species: "Homo sapiens" TF status: "direct" TF family: "MADS box" DBDs: "SRF-TF"]; Mef2a[gene ID: "ENSMUSG00000030557" species: "Mus musculus" TF status: "inferred" TF family: "MADS box" DBDs: "SRF-TF"]; Mef2c[gene ID: "ENSMUSG00000005583" species: "Mus musculus" TF status: "inferred" TF family: "MADS box" DBDs: "SRF-TF"]; Mef2d[gene ID: "ENSMUSG00000001419" species: "Mus musculus" TF status: "inferred" TF family: "MADS box" DBDs: "SRF-TF"]<br>Possible TFs: MEF2C	*8.64773*	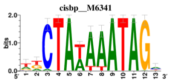	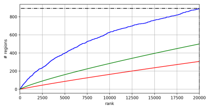	**link**	**link**	PWMs
44	☐ E107-H3K4me1<br>Description: H3K4me1 in Skeletal Muscle Male (E107,)	*8.44495*		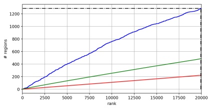	**link**	**link**	Histone modifications

#	Feature	NES	Logo	Recovery Curve	Candidate targets	All regions in top 20000	Database
45	☐ taipale__MEF2D_DBD_NCTAWAAATAGM<br>Description: MEF2D dimeric MADS DBD<br>Possible TFs: MEF2D	*8.33052*	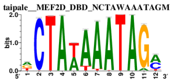	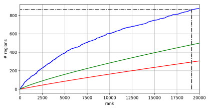	link	link	PWMs
46	☐ transfac_public__M00232<br>Description: V$MEF2_03: MEF-2A<br>Possible TFs: MEF2A	*7.95119*	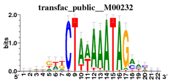	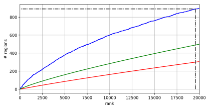	link	link	PWMs
47	☐ cisbp__M3552<br>Description: AC002126.6[gene ID: "ENSG00000064489" species: "Homo sapiens" TF status: "inferred" TF family: "MADS box" DBDs: "SRF-TF"]; MEF2A[gene ID: "ENSG00000068305" species: "Homo sapiens" TF status: "direct" TF family: "MADS box" DBDs: "SRF-TF"]; Mef2a[gene ID: "ENSMUSG00000030557" species: "Mus musculus" TF status: "inferred" TF family: "MADS box" DBDs: "SRF-TF"]; Mef2c[gene ID: "ENSMUSG00000005583" species: "Mus musculus" TF status: "inferred" TF family: "MADS box" DBDs: "SRF-TF"]; Mef2d[gene ID: "ENSMUSG00000001419" species: "Mus musculus" TF status: "inferred" TF family: "MADS box" DBDs: "SRF-TF"]<br>Possible TFs: MEF2A	*7.95055*	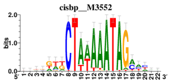	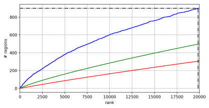	link	link	PWMs
48	☐ transfac_pro__M01809<br>Description: P$RIN_01: RIN	*7.90532*	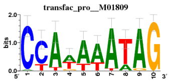	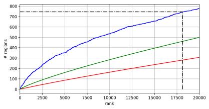	link	link	PWMs
49	☐ E108-H3K27ac<br>Description: H3K27ac in Skeletal Muscle Female (E108,)	*7.50966*		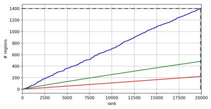	link	link	Histone modifications
50	☐ transfac_pro__M02024<br>Description: V$MEF2A_Q6: mef-2A<br>Possible TFs: MEF2A	*7.38602*	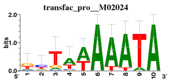	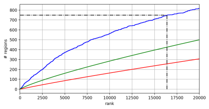	link	link	PWMs
51	☐ jaspar__MA0369.1<br>Description: RLM1<br>Possible TFs: MEF2C, MEF2B, MEF2A, BORCS8-MEF2B, MEF2D	*7.06279*	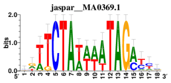	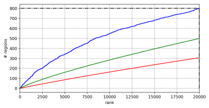	link	link	PWMs
52	☐ E108-H3K4me1<br>Description: H3K4me1 in Skeletal Muscle Female (E108,)	*7.04864*		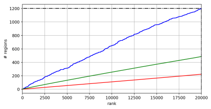	link	link	Histone modifications
53	☐ cisbp__M2174<br>Description: RLM1[gene ID: "YPL089C" species: "Saccharomyces cerevisiae" TF status: "direct" TF family: "MADS box" DBDs: "SRF-TF"]<br>Possible TFs: MEF2C, MEF2B, MEF2A, BORCS8-MEF2B, MEF2D	*6.73216*	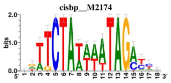	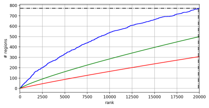	link	link	PWMs
54	☐ stark__GNCTANWWATA<br>Description: Myocyte enhancing factor 2<br>Possible TFs: MEF2C, MEF2B, MEF2A, BORCS8-MEF2B, MEF2D	*6.72425*	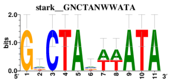	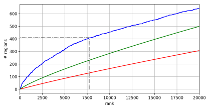	link	link	PWMs
55	☐ scertf__morozov.SMP1<br>Description: morozov.SMP1<br>Possible TFs: MEF2C, MEF2B, MEF2A, BORCS8-MEF2B, MEF2D	*6.60848*	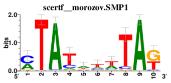	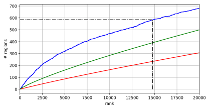	link	link	PWMs
56	☐ dbcorrdb__SMARCC1__ENCSR000EDM_1__m1<br>Description: SMARCC1 (ENCSR000EDM-1, motif 1)<br>Possible TFs: SMARCC1	*6.13087*	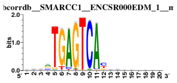	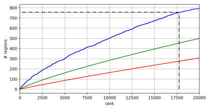	link	link	PWMs

#	Feature	NES	Logo	Recovery Curve	Candidate targets	All regions in top 20000	Database
57	☐ transfac_pro__M00406<br>Description: V$HMEF2_Q6: MEF-2A<br>Possible TFs: MEF2A	*5.98999*	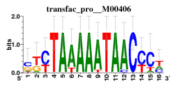	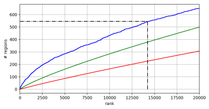	link	link	PWMs
58	☐ transfac_pro__M01164<br>Description: P$SQUA_01: SQUA	*5.94037*	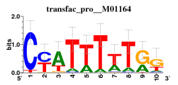	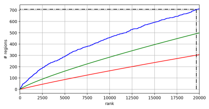	link	link	PWMs
59	☐ dbcorrdb__SMARCB1__ENCSR000EDK_1__m1<br>Description: SMARCB1 (ENCSR000EDK-1, motif 1)<br>Possible TFs: SMARCB1	*5.93865*	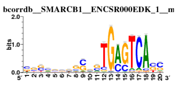	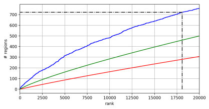	link	link	PWMs
60	☐ transfac_pro__M00403<br>Description: V$AMEF2_Q6: aMEF-2<br>Possible TFs: MEF2A	*5.91252*	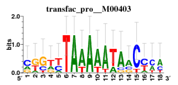	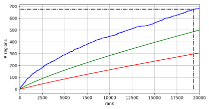	link	link	PWMs
61	☐ cisbp__M6467<br>Description: SMARCC1[gene ID: "ENSG00000173473" species: "Homo sapiens" TF status: "direct" TF family: "Myb/SANT" DBDs: "Myb_DNA-binding"]; Smarcc1[gene ID: "ENSMUSG00000032481" species: "Mus musculus" TF status: "inferred" TF family: "Myb/SANT" DBDs: "Myb_DNA-binding"]; Smarcc2[gene ID: "ENSMUSG00000025369" species: "Mus musculus" TF status: "inferred" TF family: "Myb/SANT" DBDs: "Myb_DNA-binding"]; mor[gene ID: "FBgn0002783" species: "Drosophila melanogaster" TF status: "inferred" TF family: "Myb/SANT" DBDs: "Myb_DNA-binding"]<br>Possible TFs: SMARCC1	*5.81883*	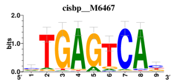	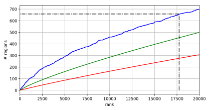	link	link	PWMs
62	☐ dbcorrdb__TCF7L2__ENCSR000EUV_1__m2<br>Description: TCF7L2 (ENCSR000EUV-1, motif 2)<br>Possible TFs: TCF7L2	*5.80529*	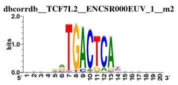	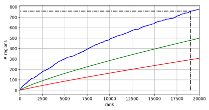	link	link	PWMs
63	☐ cisbp__M4526<br>Description: SMARCC1[gene ID: "ENSG00000173473" species: "Homo sapiens" TF status: "direct" TF family: "Myb/SANT" DBDs: "Myb_DNA-binding"]; Smarcc1[gene ID: "ENSMUSG00000032481" species: "Mus musculus" TF status: "inferred" TF family: "Myb/SANT" DBDs: "Myb_DNA-binding"]; Smarcc2[gene ID: "ENSMUSG00000025369" species: "Mus musculus" TF status: "inferred" TF family: "Myb/SANT" DBDs: "Myb_DNA-binding"]; mor[gene ID: "FBgn0002783" species: "Drosophila melanogaster" TF status: "inferred" TF family: "Myb/SANT" DBDs: "Myb_DNA-binding"]<br>Possible TFs: SMARCC1	*5.78971*	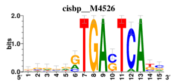	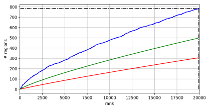	link	link	PWMs
64	☐ swissregulon__hs__BACH2.p2<br>Description: hs__BACH2.p2<br>Possible TFs: BACH2	*5.73984*	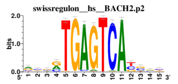	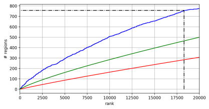	link	link	PWMs
65	☐ hocomoco__JUN_MOUSE.H11MO.0.A<br>Description: JUN_MOUSE<br>Possible TFs: JUN	*5.69027*	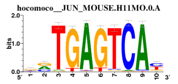	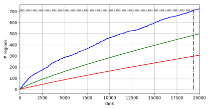	link	link	PWMs
66	☐ hocomoco__FOSL2_MOUSE.H11MO.0.A<br>Description: FOSL2_MOUSE<br>Possible TFs: FOSL2	*5.64206*	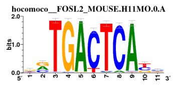	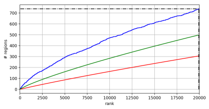	link	link	PWMs

#	Feature	NES	Logo	Recovery Curve	Candidate targets	All regions in top 20000	Database
67	☐ hocomoco__ATF3_MOUSE.H11MO.0.A<br>Description: ATF3_MOUSE<br>Possible TFs: ATF3	*5.64103*	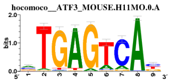	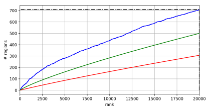	**link**	**link**	PWMs
68	☐ hocomoco__HAND1_HUMAN.H11MO.1.D<br>Description: HAND1_HUMAN<br>Possible TFs: HAND1	*5.62149*	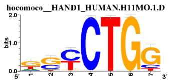	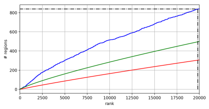	**link**	**link**	PWMs
69	☐ hocomoco__SIX2_MOUSE.H11MO.0.A<br>Description: SIX2_MOUSE<br>Possible TFs: SIX2	*5.61874*	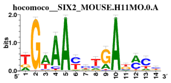	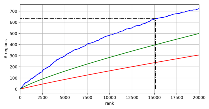	**link**	**link**	PWMs
70	☐ transfac_public__M00517<br>Description: V$AP1_01: AP-1<br>Possible TFs: FOS, FOSB, JUNB, JUN, JUND, FOSL1	*5.61043*	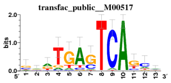	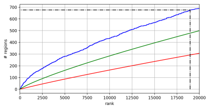	**link**	**link**	PWMs
71	☐ dbcorrdb__JUND__ENCSR000BGK_1__m1<br>Description: JUND (ENCSR000BGK-1, motif 1)<br>Possible TFs: JUND	*5.59943*	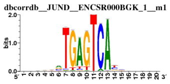	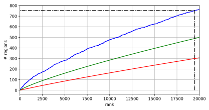	**link**	**link**	PWMs
72	☐ hocomoco__JUND_HUMAN.H11MO.0.A<br>Description: JUND_HUMAN<br>Possible TFs: JUND	*5.58850*	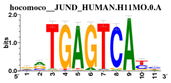	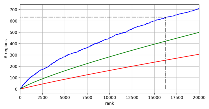	**link**	**link**	PWMs
73	☐ hocomoco__FOSL1_HUMAN.H11MO.0.A<br>Description: FOSL1_HUMAN<br>Possible TFs: FOSL1	*5.57058*	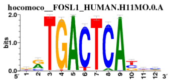	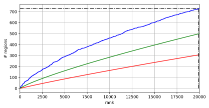	**link**	**link**	PWMs
74	☐ dbcorrdb__FOSL1__ENCSR000BMV_1__m1<br>Description: FOSL1 (ENCSR000BMV-1, motif 1)<br>Possible TFs: FOSL1	*5.56507*	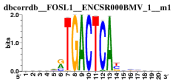	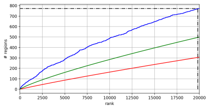	**link**	**link**	PWMs
75	☐ dbcorrdb__JUN__ENCSR000EFS_1__m1<br>Description: JUN (ENCSR000EFS-1, motif 1)<br>Possible TFs: JUN	*5.53644*	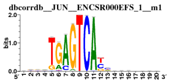	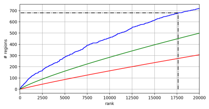	**link**	**link**	PWMs
76	☐ E107-H3K4me1-broadpeak<br>Description: H3K4me1 in Skeletal Muscle Male (E107, broadpeak)	*5.52440*		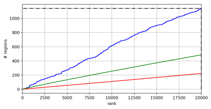	**link**	**link**	Histone modifications
77	☐ hocomoco__SIX1_HUMAN.H11MO.0.A<br>Description: SIX1_HUMAN<br>Possible TFs: SIX1	*5.50778*	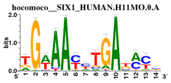	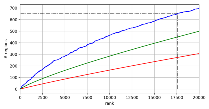	**link**	**link**	PWMs
78	☐ cisbp__M2279<br>Description: FOSL1[gene ID: "ENSG00000175592" species: "Homo sapiens" TF status: "direct" TF family: "bZIP" DBDs: "bZIP_1"]; Fos[gene ID: "ENSMUSG00000021250" species: "Mus musculus" TF status: "inferred" TF family: "bZIP" DBDs: "bZIP_1"]; Fosb[gene ID: "ENSMUSG00000003545" species: "Mus musculus" TF status: "inferred" TF family: "bZIP" DBDs: "bZIP_1"]<br>Possible TFs: FOSL1	*5.50160*	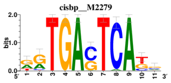	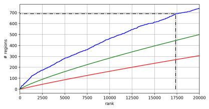	**link**	**link**	PWMs
79	☐ transfac_public__M00199<br>Description: V$AP1_C: AP-1<br>Possible TFs: JUN, FOS	*5.49823*	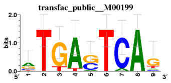	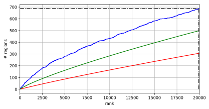	**link**	**link**	PWMs

#	Feature	NES	Logo	Recovery Curve	Candidate targets	All regions in top 20000	Database
80	☐ homer__ATGACTCATC_AP-1<br>Description: AP-1(bZIP)/ThioMac-PU.1-ChIP-Seq(GSE21512)/Homer<br>Possible TFs: JUN	5.48775	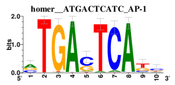	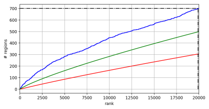	link	link	PWMs
81	☐ elemento__TGACTCA<br>Description: Conserved regulatory element TGACTCA between Dmel and Dpse	5.48008	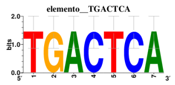	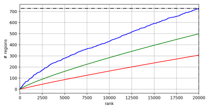	link	link	PWMs
82	☐ jaspar__MA0477.1<br>Description: FOSL1<br>Possible TFs: FOSL1	5.47728	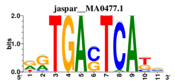	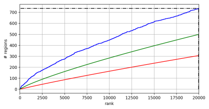	link	link	PWMs
83	☐ transfac_pro__M07600<br>Description: V$CFOS_Q4: C-Fos<br>Possible TFs: FOS	5.44862	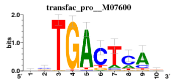	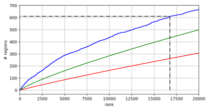	link	link	PWMs
84	☐ dbcorrdb__RCOR1__ENCSR000ECM_1__m1<br>Description: RCOR1 (ENCSR000ECM-1, motif 1)<br>Possible TFs: RCOR1	5.44532	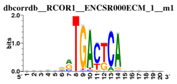	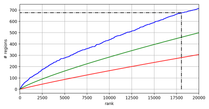	link	link	PWMs
85	☐ hocomoco__FOS_HUMAN.H11MO.0.A<br>Description: FOS_HUMAN<br>Possible TFs: FOS	5.42826	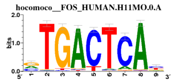	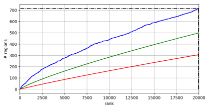	link	link	PWMs
86	☐ hocomoco__JUNB_HUMAN.H11MO.0.A<br>Description: JUNB_HUMAN<br>Possible TFs: JUNB	5.41975	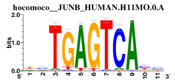	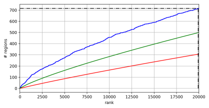	link	link	PWMs
87	☐ cisbp__M6230<br>Description: FOSL2[gene ID: "ENSG00000075426" species: "Homo sapiens" TF status: "direct" TF family: "bZIP" DBDs: "bZIP_1"]<br>Possible TFs: FOSL2	5.41829	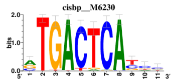	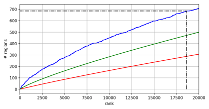	link	link	PWMs
88	☐ factorbook__AP1<br>Description: AP1<br>Possible TFs: BATF, FOS, JUNB, JUN, JUND, FOSL1, FOSL2	5.41055	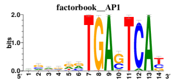	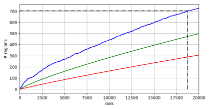	link	link	PWMs
89	☐ cisbp__M6318<br>Description: JUND[gene ID: "ENSG00000130522" species: "Homo sapiens" TF status: "direct" TF family: "bZIP" DBDs: "bZIP_1"]<br>Possible TFs: JUND	5.35490	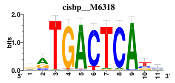	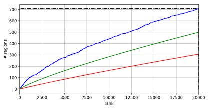	link	link	PWMs
90	☐ dbcorrdb__JUN__ENCSR000FAH_1__m1<br>Description: JUN (ENCSR000FAH-1, motif 1)<br>Possible TFs: JUN	5.35486	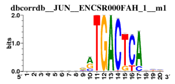	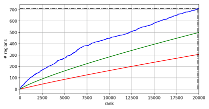	link	link	PWMs
91	☐ cisbp__M6319<br>Description: JUN[gene ID: "ENSG00000177606" species: "Homo sapiens" TF status: "direct" TF family: "bZIP" DBDs: "bZIP_1"]<br>Possible TFs: JUN	5.34745	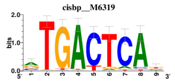	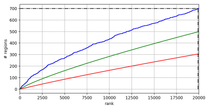	link	link	PWMs
92	☐ transfac_pro__M03869<br>Description: V$FRA1_Q6_01: Fra-1<br>Possible TFs: FOSL1	5.34055	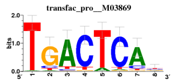	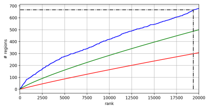	link	link	PWMs

#	Feature	NES	Logo	Recovery Curve	Candidate targets	All regions in top 20000	Database
93	homer__GATGACTCATCN_Jun-AP1<br>Description: Jun-AP1(bZIP)/K562-cJun-ChIP-Seq(GSE31477)/Homer<br>Possible TFs: JUN	*5.32192*	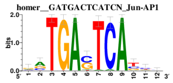	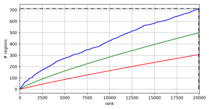	**link**	**link**	PWMs
94	dbcorrdb__ATF3__ENCSR000BNU_1__m1<br>Description: ATF3 (ENCSR000BNU-1, motif 1)<br>Possible TFs: ATF3	*5.31145*	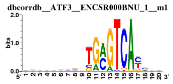	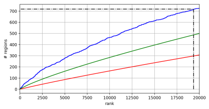	**link**	**link**	PWMs
95	hocomoco__FOSB_MOUSE.H11MO.0.A<br>Description: FOSB_MOUSE<br>Possible TFs: FOSB	*5.31021*	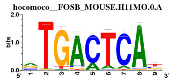	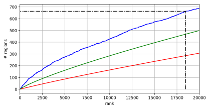	**link**	**link**	PWMs
96	dbcorrdb__eGFP-FOS__ENCSR000DKB_1__m1<br>Description: eGFP-FOS (ENCSR000DKB-1, motif 1)	*5.29979*	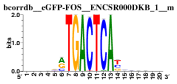	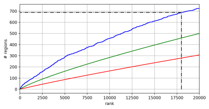	**link**	**link**	PWMs
97	dbcorrdb__MEF2A__ENCSR000BNV_1__m2<br>Description: MEF2A (ENCSR000BNV-1, motif 2)<br>Possible TFs: MEF2A	*5.29835*	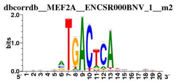	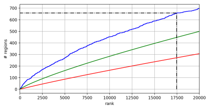	**link**	**link**	PWMs
98	transfac_pro__M02095<br>Description: V$FRA1_Q6: Fra-1<br>Possible TFs: FOSL1	*5.27661*	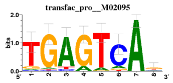	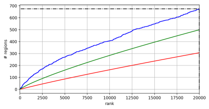	**link**	**link**	PWMs
99	dbcorrdb__JUND__ENCSR000EGN_1__m1<br>Description: JUND (ENCSR000EGN-1, motif 1)<br>Possible TFs: JUND	*5.26601*	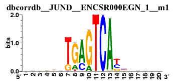	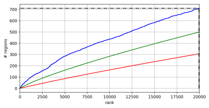	**link**	**link**	PWMs
100	cisbp__M4590<br>Description: FOS[gene ID: "ENSG00000170345" species: "Homo sapiens" TF status: "direct" TF family: "bZIP" DBDs: "bZIP_1"]; Fos[gene ID: "ENSMUSG00000021250" species: "Mus musculus" TF status: "inferred" TF family: "bZIP" DBDs: "bZIP_1"]; Fosb[gene ID: "ENSMUSG00000003545" species: "Mus musculus" TF status: "inferred" TF family: "bZIP" DBDs: "bZIP_1"]<br>Possible TFs: FOS	*5.26474*	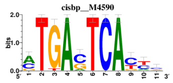	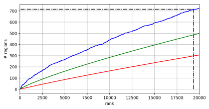	**link**	**link**	PWMs
101	yetfasco__YIL056W_2091<br>Description: YIL056W_2091	*5.26432*	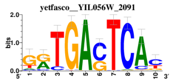	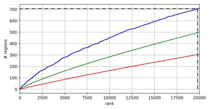	**link**	**link**	PWMs
102	cisbp__M4625<br>Description: JUND[gene ID: "ENSG00000130522" species: "Homo sapiens" TF status: "direct" TF family: "bZIP" DBDs: "bZIP_1"]<br>Possible TFs: JUND	*5.25420*	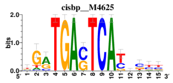	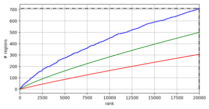	**link**	**link**	PWMs
103	cisbp__M4623<br>Description: JUNB[gene ID: "ENSG00000171223" species: "Homo sapiens" TF status: "direct" TF family: "bZIP" DBDs: "bZIP_1"]<br>Possible TFs: JUNB	*5.25362*	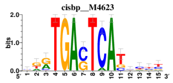	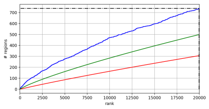	**link**	**link**	PWMs
104	hocomoco__JUND_MOUSE.H11MO.0.A<br>Description: JUND_MOUSE<br>Possible TFs: JUND	*5.24884*	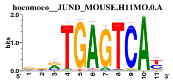	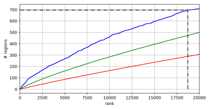	**link**	**link**	PWMs

#	Feature	NES	Logo	Recovery Curve	Candidate targets	All regions in top 20000	Database
105	☐ cisbp__M2291<br>Description: JUNB[gene ID: "ENSG00000171223" species: "Homo sapiens" TF status: "direct" TF family: "bZIP" DBDs: "bZIP_1"]<br>Possible TFs: JUNB	*5.24541*	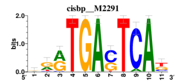	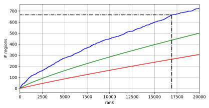	link	link	PWMs
106	☐ cisbp__M4607<br>Description: FOS[gene ID: "ENSG00000170345" species: "Homo sapiens" TF status: "direct" TF family: "bZIP" DBDs: "bZIP_1"]; Fos[gene ID: "ENSMUSG00000021250" species: "Mus musculus" TF status: "inferred" TF family: "bZIP" DBDs: "bZIP_1"]; Fosb[gene ID: "ENSMUSG00000003545" species: "Mus musculus" TF status: "inferred" TF family: "bZIP" DBDs: "bZIP_1"]<br>Possible TFs: FOS	*5.24468*	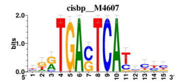	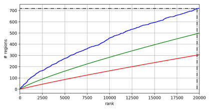	link	link	PWMs
107	☐ hocomoco__JUNB_MOUSE.H11MO.0.A<br>Description: JUNB_MOUSE<br>Possible TFs: JUNB	*5.24321*	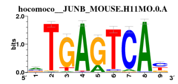	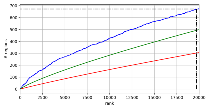	link	link	PWMs
108	☐ predrem__nrMotif2175<br>Description: 74_fIntestine_Sm-DS17643.M608	*5.24109*	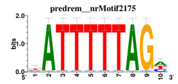	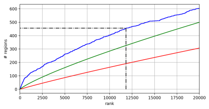	link	link	PWMs
109	☐ ENCFF001WPB<br>Description: DNase-seq on human LHCN-M2 differentiated for 4 days	*5.23867*		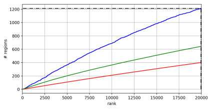	link	link	DHS & FAIRE
110	☐ transfac_public__M00006<br>Description: V$MEF2_01: MEF-2A<br>Possible TFs: MEF2A	*5.22759*	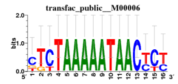	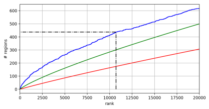	link	link	PWMs
111	☐ dbcorrdb__FOSL2__ENCSR000BHP_1__m1<br>Description: FOSL2 (ENCSR000BHP-1, motif 1)<br>Possible TFs: FOSL2	*5.22002*	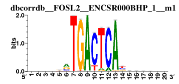	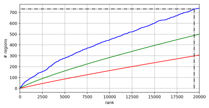	link	link	PWMs
112	☐ transfac_public__M00174<br>Description: V$AP1_Q6: AP-1<br>Possible TFs: JUN, FOS	*5.19155*	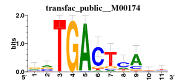	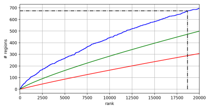	link	link	PWMs
113	☐ cisbp__M4506<br>Description: JUND[gene ID: "ENSG00000130522" species: "Homo sapiens" TF status: "direct" TF family: "bZIP" DBDs: "bZIP_1"]<br>Possible TFs: JUND	*5.18886*	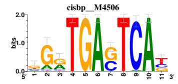	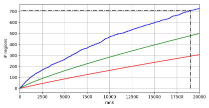	link	link	PWMs
114	☐ dbcorrdb__POLR2AphosphoS5__ENCSR000BQC_1__m2<br>Description: POLR2AphosphoS5 (ENCSR000BQC-1, motif 2)	*5.17980*	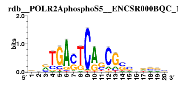	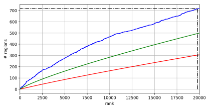	link	link	PWMs
115	☐ jaspar__MA0490.1<br>Description: JUNB<br>Possible TFs: JUNB	*5.17909*	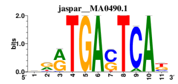	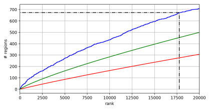	link	link	PWMs
116	☐ cisbp__M4608<br>Description: JUN[gene ID: "ENSG00000177606" species: "Homo sapiens" TF status: "direct" TF family: "bZIP" DBDs: "bZIP_1"]<br>Possible TFs: JUN	*5.16951*	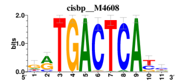	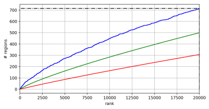	link	link	PWMs

#	Feature	NES	Logo	Recovery Curve	Candidate targets	All regions in top 20000	Database
117	☐ yetfasco__YEL009C_1363<br>Description: YEL009C_1363	5.16120	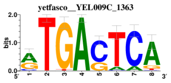	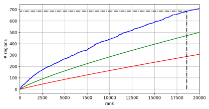	link	link	PWMs
118	☐ cisbp__M6317<br>Description: JUNB[gene ID: "ENSG00000171223" species: "Homo sapiens" TF status: "direct" TF family: "bZIP" DBDs: "bZIP_1"]<br>Possible TFs: JUNB	5.15373	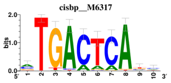	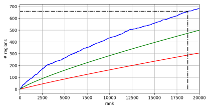	link	link	PWMs
119	☐ E111-H3K4me1<br>Description: H3K4me1 in Stomach Smooth Muscle (E111,)	5.14942		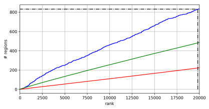	link	link	Histone modifications
120	☐ dbcorrdb__MYC__ENCSR000DOM_1__m2<br>Description: MYC (ENCSR000DOM-1, motif 2)<br>Possible TFs: MYC	5.14111	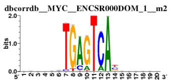	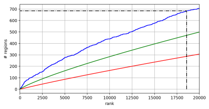	link	link	PWMs
121	☐ cisbp__M6229<br>Description: FOSL1[gene ID: "ENSG00000175592" species: "Homo sapiens" TF status: "direct" TF family: "bZIP" DBDs: "bZIP_1"]; Fos[gene ID: "ENSMUSG00000021250" species: "Mus musculus" TF status: "inferred" TF family: "bZIP" DBDs: "bZIP_1"]; Fosb[gene ID: "ENSMUSG00000003545" species: "Mus musculus" TF status: "inferred" TF family: "bZIP" DBDs: "bZIP_1"]<br>Possible TFs: FOSL1	5.12654	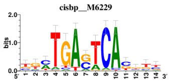	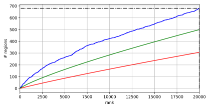	link	link	PWMs
122	☐ transfac_pro__M03870<br>Description: V$FRA2_Q4_01: Fra-2<br>Possible TFs: FOSL2	5.12081	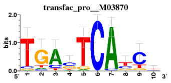	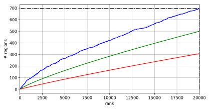	link	link	PWMs
123	☐ hdpi__NAP1L1<br>Description: NAP1L1<br>Possible TFs: NAP1L1	5.10954	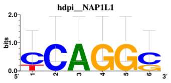	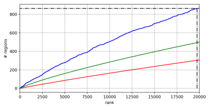	link	link	PWMs
124	☐ hocomoco__JUN_HUMAN.H11MO.0.A<br>Description: JUN_HUMAN<br>Possible TFs: JUN	5.09796	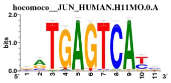	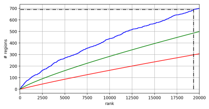	link	link	PWMs
125	☐ dbcorrdb__RCOR1__ENCSR000EFG_1__m1<br>Description: RCOR1 (ENCSR000EFG-1, motif 1)<br>Possible TFs: RCOR1	5.09688	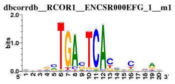	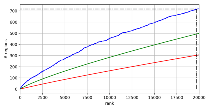	link	link	PWMs
126	☐ dbcorrdb__eGFP-JUNB__ENCSR000DJY_1__m1<br>Description: eGFP-JUNB (ENCSR000DJY-1, motif 1)	5.07569	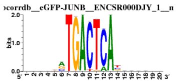	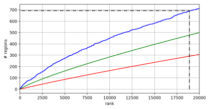	link	link	PWMs
127	☐ E103-H3K4me1<br>Description: H3K4me1 in Rectal Smooth Muscle (E103,)	5.06236		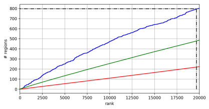	link	link	Histone modifications
128	☐ transfac_pro__M07467<br>Description: V$SIX4_Q3: Six-4<br>Possible TFs: SIX4	5.05672	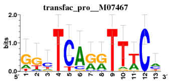	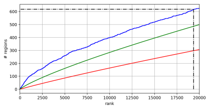	link	link	PWMs
129	☐ transfac_pro__M03866<br>Description: V$CFOSCJUN_Q5: c-Fos:c-Jun<br>Possible TFs: JUN, FOS	5.04763	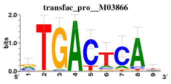	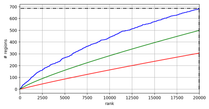	link	link	PWMs

#	Feature	NES	Logo	Recovery Curve	Candidate targets	All regions in top 20000	Database
130	☐ swissregulon__hs__FOSL2.p2<br>Description: hs__FOSL2.p2<br>Possible TFs: FOSL2	*5.03857*	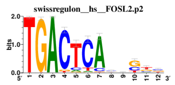	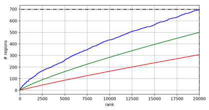	link	link	PWMs
131	☐ hocomoco__FOS_MOUSE.H11MO.0.A<br>Description: FOS_MOUSE<br>Possible TFs: FOS	*5.02571*	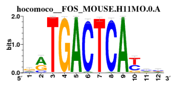	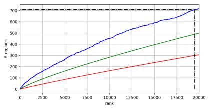	link	link	PWMs
132	☐ cisbp__M2280<br>Description: FOSL2[gene ID: "ENSG00000075426" species: "Homo sapiens" TF status: "direct" TF family: "bZIP" DBDs: "bZIP_1"]<br>Possible TFs: FOSL2	*5.02381*	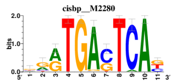	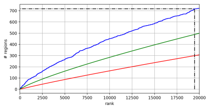	link	link	PWMs
133	☐ homer__NATGASTCABNN_Fosl2<br>Description: Fosl2(bZIP)/3T3L1-Fosl2-ChIP-Seq(GSE56872)/Homer<br>Possible TFs: FOSL2	*4.97282*	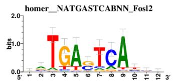	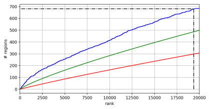	link	link	PWMs
134	☐ cisbp__M4619<br>Description: FOSL1[gene ID: "ENSG00000175592" species: "Homo sapiens" TF status: "direct" TF family: "bZIP" DBDs: "bZIP_1"]; Fos[gene ID: "ENSMUSG00000021250" species: "Mus musculus" TF status: "inferred" TF family: "bZIP" DBDs: "bZIP_1"]; Fosb[gene ID: "ENSMUSG00000003545" species: "Mus musculus" TF status: "inferred" TF family: "bZIP" DBDs: "bZIP_1"]<br>Possible TFs: FOSL1	*4.97204*	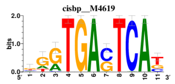	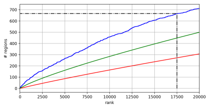	link	link	PWMs
135	☐ hocomoco__FOSB_HUMAN.H11MO.0.A<br>Description: FOSB_HUMAN<br>Possible TFs: FOSB	*4.96734*	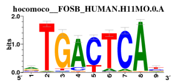	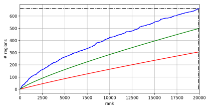	link	link	PWMs
136	☐ E108-H3K4me1-broadpeak<br>Description: H3K4me1 in Skeletal Muscle Female (E108, broadpeak)	*4.96540*		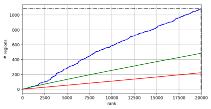	link	link	Histone modifications
137	☐ transfac_pro__M01721<br>Description: V$PUR1_Q4: PUR1<br>Possible TFs: PURA	*4.95801*	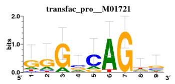	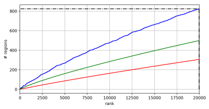	link	link	PWMs
138	☐ jaspar__MA0478.1<br>Description: FOSL2<br>Possible TFs: FOSL2	*4.95793*	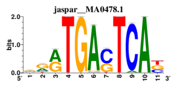	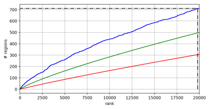	link	link	PWMs
139	☐ flyfactorsurvey__kay_Jra_SANGER_5_FBgn0001291<br>Description: kay_Jra_SANGER_5_FBgn0001291<br>Possible TFs: JUNB, JUN, JUND	*4.94330*	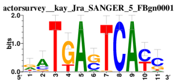	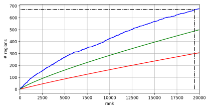	link	link	PWMs
140	☐ transfac_pro__M03538<br>Description: V$AP1_Q6_02: AP-1<br>Possible TFs: JUN, FOS	*4.92597*	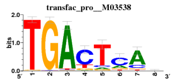	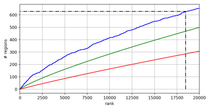	link	link	PWMs
141	☐ cisbp__M4565<br>Description: FOSL2[gene ID: "ENSG00000075426" species: "Homo sapiens" TF status: "direct" TF family: "bZIP" DBDs: "bZIP_1"]; Fos[gene ID: "ENSMUSG00000021250" species: "Mus musculus" TF status: "inferred" TF family: "bZIP" DBDs: "bZIP_1"]; Fosb[gene ID: "ENSMUSG00000003545" species: "Mus musculus" TF status: "inferred" TF family: "bZIP" DBDs: "bZIP_1"]<br>Possible TFs: FOSL2	*4.91434*	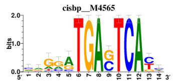	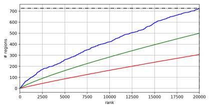	link	link	PWMs

#	Feature	NES	Logo	Recovery Curve	Candidate targets	All regions in top 20000	Database
142	☐ hdpi__RBM17<br>Description: RBM17<br>Possible TFs: RBM17	4.89867	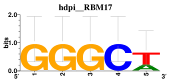	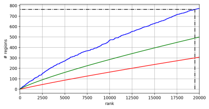	link	link	PWMs
143	☐ transfac_pro__M04685<br>Description: V$NFE2_04: NF-E2<br>Possible TFs: NFE2	4.88351	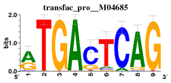	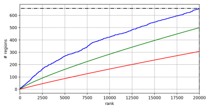	link	link	PWMs
144	☐ transfac_pro__M08926<br>Description: V$JUNB_02: JUNB<br>Possible TFs: JUNB	4.87393	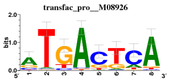	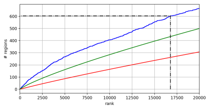	link	link	PWMs
145	☐ homer__DATGASTCATHN_Atf3<br>Description: Atf3(bZIP)/GBM-ATF3-ChIP-Seq(GSE33912)/Homer<br>Possible TFs: ATF3	4.86065	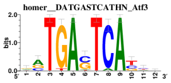	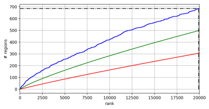	link	link	PWMs
146	☐ cisbp__M4570<br>Description: JUND[gene ID: "ENSG00000130522" species: "Homo sapiens" TF status: "direct" TF family: "bZIP" DBDs: "bZIP_1"]<br>Possible TFs: JUND	4.86011	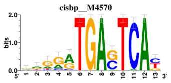	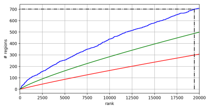	link	link	PWMs
147	☐ E090-H3K4me1<br>Description: H3K4me1 in Fetal Muscle Leg (E090,)	4.85807		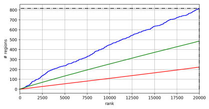	link	link	Histone modifications
148	☐ transfac_pro__M01267<br>Description: V$FRA1_Q5: Fra-1<br>Possible TFs: FOSL1	4.84509	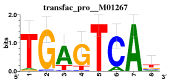	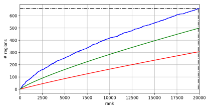	link	link	PWMs
149	☐ taipale_cyt_meth__JDP2_NRTGASTCAYN_FL_meth<br>Description: JDP2 [bZIP, CpG-meth]<br>Possible TFs: JDP2	4.84429	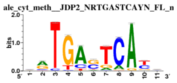	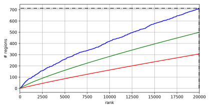	link	link	PWMs
150	☐ swissregulon__hs__FOS_FOS_B_L1__JUN_B_D_.p2<br>Description: hs__FOS_FOS{B,L1}_JUN{B,D}.p2<br>Possible TFs: FOSB, JUNB, FOSL1, JUND, FOS	4.84186	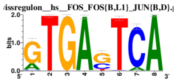	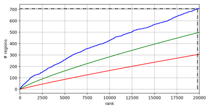	link	link	PWMs
151	☐ cisbp__M5050<br>Description: Jra[gene ID: "FBgn0001291" species: "Drosophila melanogaster" TF status: "direct" TF family: "bZIP" DBDs: "bZIP_1"]<br>Possible TFs: JUNB, JUN, JUND	4.83083	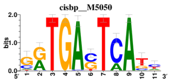	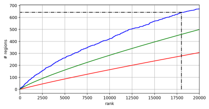	link	link	PWMs
152	☐ hdpi__FGF19<br>Description: FGF19<br>Possible TFs: FGF19	4.82554	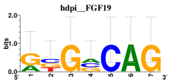	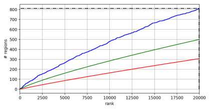	link	link	PWMs
153	☐ transfac_pro__M03552<br>Description: V$JUND_Q6: JunD<br>Possible TFs: JUND	4.82482	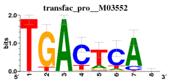	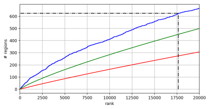	link	link	PWMs
154	☐ homer__DATGASTCAT_BATF<br>Description: BATF(bZIP)/Th17-BATF-ChIP-Seq(GSE39756)/Homer<br>Possible TFs: BATF	4.82092	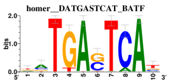	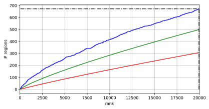	link	link	PWMs

#	Feature	NES	Logo	Recovery Curve	Candidate targets	All regions in top 20000	Database
155	☐ dbcorrdb__GATA2__ENCSR000EVW_1__m3<br>Description: GATA2 (ENCSR000EVW-1, motif 3)<br>Possible TFs: GATA2	*4.81417*	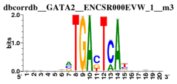	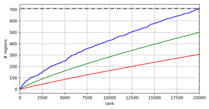	**link**	**link**	PWMs
156	☐ E095-H3K27ac<br>Description: H3K27ac in Left Ventricle (E095,)	*4.80370*		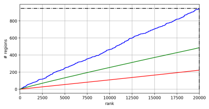	**link**	**link**	Histone modifications
157	☐ dbcorrdb__JUND__ENCSR000EBZ_1__m1<br>Description: JUND (ENCSR000EBZ-1, motif 1)<br>Possible TFs: JUND	*4.79675*	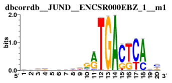	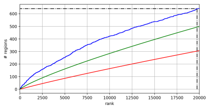	**link**	**link**	PWMs
158	☐ transfac_pro__M00926<br>Description: V$AP1_Q4_01: AP-1<br>Possible TFs: FOS, FOSB, JUNB, JUN, JUND, FOSL1, FOSL2	*4.79116*	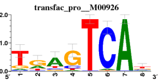	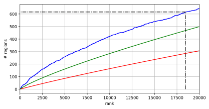	**link**	**link**	PWMs
159	☐ dbcorrdb__JUN__ENCSR000EGH_1__m1<br>Description: JUN (ENCSR000EGH-1, motif 1)<br>Possible TFs: JUN	*4.78748*	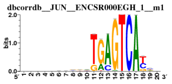	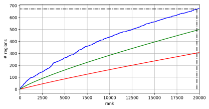	**link**	**link**	PWMs
160	☐ hocomoco__FOSL2_HUMAN.H11MO.0.A<br>Description: FOSL2_HUMAN<br>Possible TFs: FOSL2	*4.78734*	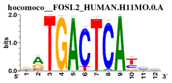	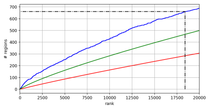	**link**	**link**	PWMs
161	☐ transfac_pro__M03815<br>Description: V$CFOS_Q6: c-Fos<br>Possible TFs: FOS	*4.78655*	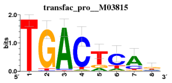	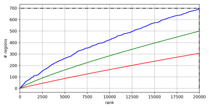	**link**	**link**	PWMs
162	☐ ENCFF001TSQ<br>Description: NR3C1 ChIP-seq (protocol PCR1x) on A549 treated with dexamethasone at 50nM	*4.75352*		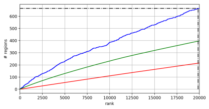	**link**	**link**	TF binding sites
163	☐ dbcorrdb__JUN__ENCSR000EZT_1__m1<br>Description: JUN (ENCSR000EZT-1, motif 1)<br>Possible TFs: JUN	*4.75151*	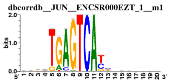	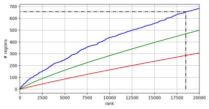	**link**	**link**	PWMs
164	☐ dbcorrdb__eGFP-JUND__ENCSR000DJX_1__m1<br>Description: eGFP-JUND (ENCSR000DJX-1, motif 1)	*4.73855*	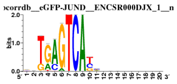	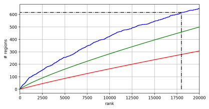	**link**	**link**	PWMs
165	☐ transfac_public__M00490<br>Description: V$BACH2_01: Bach2<br>Possible TFs: BACH2	*4.72446*	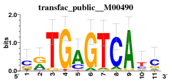	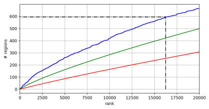	**link**	**link**	PWMs
166	☐ ENCFF001UKJ<br>Description: MEF2A ChIP-seq protocol v041610.1 on human K562	*4.72216*		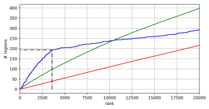	**link**	**link**	TF binding sites
167	☐ dbcorrdb__EP300__ENCSR000BPW_1__m1<br>Description: EP300 (ENCSR000BPW-1, motif 1)<br>Possible TFs: EP300	*4.70987*	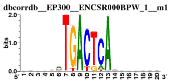	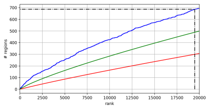	**link**	**link**	PWMs
168	☐ dbcorrdb__STAT3__ENCSR000DOU_1__m2<br>Description: STAT3 (ENCSR000DOU-1, motif 2)<br>Possible TFs: STAT3	*4.70214*	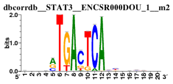	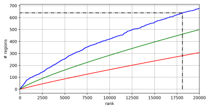	**link**	**link**	PWMs

#	Feature	NES	Logo	Recovery Curve	Candidate targets	All regions in top 20000	Database
169	☐ hocomoco__SIX2_HUMAN.H11MO.0.A<br>Description: SIX2_HUMAN<br>Possible TFs: SIX2	*4.69097*	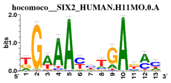	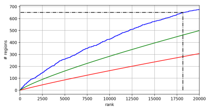	**link**	**link**	PWMs
170	☐ transfac_pro__M03551<br>Description: V$JUNB_Q6: JunB<br>Possible TFs: JUNB	*4.68204*	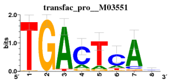	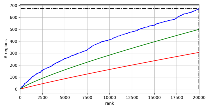	**link**	**link**	PWMs
171	☐ cisbp__M4530<br>Description: FOS[gene ID: "ENSG00000170345" species: "Homo sapiens" TF status: "direct" TF family: "bZIP" DBDs: "bZIP_1"]; Fos[gene ID: "ENSMUSG00000021250" species: "Mus musculus" TF status: "inferred" TF family: "bZIP" DBDs: "bZIP_1"]; Fosb[gene ID: "ENSMUSG00000003545" species: "Mus musculus" TF status: "inferred" TF family: "bZIP" DBDs: "bZIP_1"]<br>Possible TFs: FOS	*4.67904*	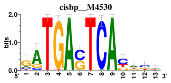	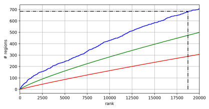	**link**	**link**	PWMs
172	☐ E083-H3K4me1<br>Description: H3K4me1 in Fetal Heart (E083,)	*4.67874*		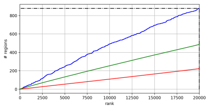	**link**	**link**	Histone modifications
173	☐ ENCFF001TSU<br>Description: NR3C1 ChIP-seq (protocol PCR2x) on A549 treated with dexamethasone at 100nM	*4.66093*		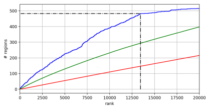	**link**	**link**	TF binding sites
174	☐ dbcorrdb__CEBPB__ENCSR000EFM_1__m2<br>Description: CEBPB (ENCSR000EFM-1, motif 2)<br>Possible TFs: CEBPB	*4.66042*	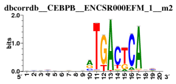	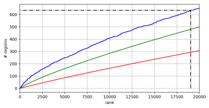	**link**	**link**	PWMs
175	☐ dbcorrdb__FOS__ENCSR000EZE_1__m1<br>Description: FOS (ENCSR000EZE-1, motif 1)<br>Possible TFs: FOS	*4.65175*	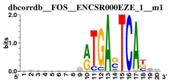	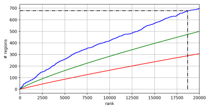	**link**	**link**	PWMs
176	☐ dbcorrdb__JUN__ENCSR000EZW_1__m1<br>Description: JUN (ENCSR000EZW-1, motif 1)<br>Possible TFs: JUN	*4.64601*	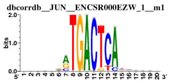	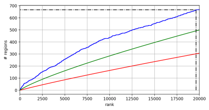	**link**	**link**	PWMs
177	☐ taipale_cyt_meth__ZNF32_NYGTAACNYGAYACN_FL<br>Description: ZNF32 [Znf_C2H2]<br>Possible TFs: ZNF32	*4.64574*	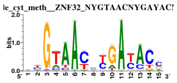	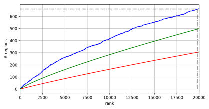	**link**	**link**	PWMs
178	☐ homer__NNATGASTCATH_Fra1<br>Description: Fra1(bZIP)/BT549-Fra1-ChIP-Seq(GSE46166)/Homer	*4.64286*	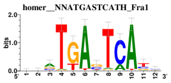	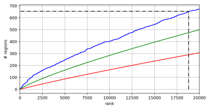	**link**	**link**	PWMs
179	☐ E128-H3K4me1<br>Description: H3K4me1 in NHLF Lung Fibroblast Primary Cells (E128,)	*4.62795*		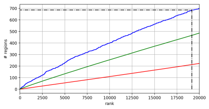	**link**	**link**	Histone modifications
180	☐ cisbp__M2980<br>Description: BACH2[gene ID: "ENSG00000112182" species: "Homo sapiens" TF status: "direct" TF family: "bZIP" DBDs: "bZIP_1"]; Bach1[gene ID: "ENSMUSG00000025612" species: "Mus musculus" TF status: "inferred" TF family: "bZIP" DBDs: "bZIP_1"]; Bach2[gene ID: "ENSMUSG00000040270" species: "Mus musculus" TF status: "inferred" TF family: "bZIP" DBDs: "bZIP_1"]<br>Possible TFs: BACH2	*4.61800*	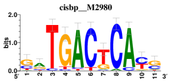	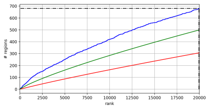	**link**	**link**	PWMs

#	Feature	NES	Logo	Recovery Curve	Candidate targets	All regions in top 20000	Database
181	☐ hdpi__HLCS<br>Description: HLCS<br>Possible TFs: HLCS	4.61574	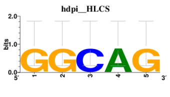	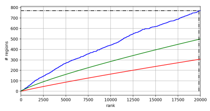	link	link	PWMs
182	☐ ENCFF001UPN<br>Description: MEF2A ChIP-seq protocol v042211.1 on human SK-N-SH	4.60621		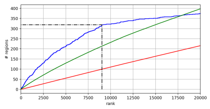	link	link	TF binding sites
183	☐ hdpi__SMAP1L<br>Description: SMAP1L<br>Possible TFs: SMAP2	4.58995	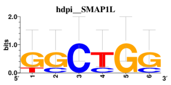	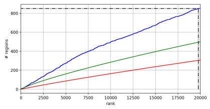	link	link	PWMs
184	☐ dbcorrdb__FOS__ENCSR000EVU_1__m1<br>Description: FOS (ENCSR000EVU-1, motif 1)<br>Possible TFs: FOS	4.58725	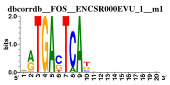	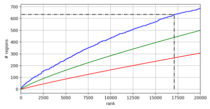	link	link	PWMs
185	☐ jaspar__MA0489.1<br>Description: JUN(var.2)<br>Possible TFs: JUN	4.58041	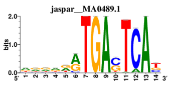	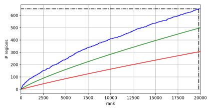	link	link	PWMs
186	☐ transfac_pro__M00405<br>Description: V$MMEF2_Q6: MEF-2A<br>Possible TFs: MEF2A	4.57968	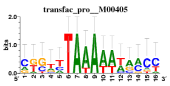	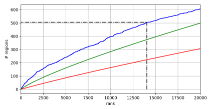	link	link	PWMs
187	☐ hocomoco__NFIC_MOUSE.H11MO.1.A<br>Description: NFIC_MOUSE<br>Possible TFs: NFIC	4.56803	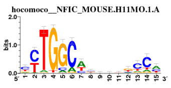	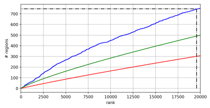	link	link	PWMs
188	☐ transfac_pro__M01859<br>Description: V$AP2GAMMA_Q5: AP-2 gamma<br>Possible TFs: TFAP2C	4.56652	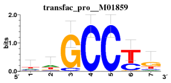	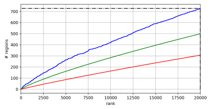	link	link	PWMs
189	☐ hdpi__TRIM21<br>Description: TRIM21<br>Possible TFs: TRIM21	4.56525	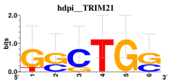	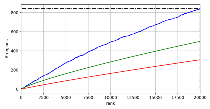	link	link	PWMs
190	☐ cisbp__M6158<br>Description: BATF[gene ID: "ENSG00000156127" species: "Homo sapiens" TF status: "direct" TF family: "bZIP" DBDs: "bZIP_1"]; Batf3[gene ID: "ENSMUSG00000026630" species: "Mus musculus" TF status: "inferred" TF family: "bZIP" DBDs: "bZIP_1"]; Batf[gene ID: "ENSMUSG00000034266" species: "Mus musculus" TF status: "inferred" TF family: "bZIP" DBDs: "bZIP_1"]<br>Possible TFs: BATF	4.55730	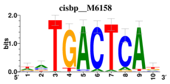	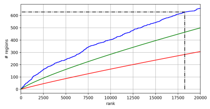	link	link	PWMs
191	☐ ENCFF001TSD<br>Description: FOSL2 ChIP-seq (protocol v042211.1) on A549 treated with ethanol at 0.02%	4.55363		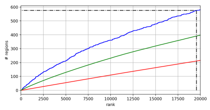	link	link	TF binding sites
192	☐ dbcorrdb__BCL3__ENCSR000BQH_1__m2<br>Description: BCL3 (ENCSR000BQH-1, motif 2)<br>Possible TFs: BCL3	4.55212	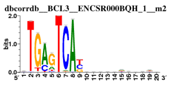	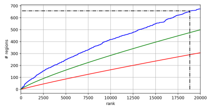	link	link	PWMs
193	☐ E107-H3K9ac<br>Description: H3K9ac in Skeletal Muscle Male (E107,)	4.54109		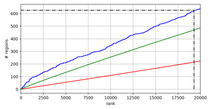	link	link	Histone modifications

#	Feature	NES	Logo	Recovery Curve	Candidate targets	All regions in top 20000	Database
194	☐ E083-H3K4me1-broadpeak<br>Description: H3K4me1 in Fetal Heart (E083, broadpeak)	*4.53199*		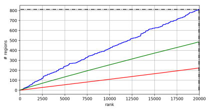	link	link	Histone modifications
195	☐ cisbp__M2278<br>Description: FOS[gene ID: "ENSG00000170345" species: "Homo sapiens" TF status: "direct" TF family: "bZIP" DBDs: "bZIP_1"]; Fos[gene ID: "ENSMUSG00000021250" species: "Mus musculus" TF status: "inferred" TF family: "bZIP" DBDs: "bZIP_1"]; Fosb[gene ID: "ENSMUSG00000003545" species: "Mus musculus" TF status: "inferred" TF family: "bZIP" DBDs: "bZIP_1"]<br>Possible TFs: FOS	*4.52202*	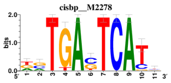	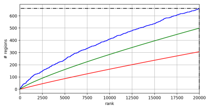	link	link	PWMs
196	☐ cisbp__M2428<br>Description: GCN4[gene ID: "YEL009C" species: "Saccharomyces cerevisiae" TF status: "direct" TF family: "bZIP" DBDs: "bZIP_1"]	*4.52009*	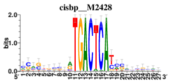	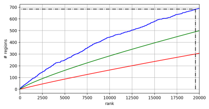	link	link	PWMs
197	☐ predrem__nrMotif196<br>Description: 111_fKidney_renal_pelvis-DS17381.M915	*4.51868*	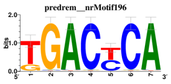	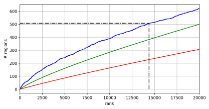	link	link	PWMs
198	☐ transfac_public__M00038<br>Description: F$GCN4_01: Gcn4p	*4.51363*	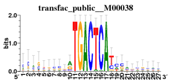	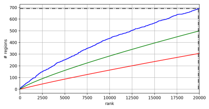	link	link	PWMs
199	☐ hocomoco__ZN554_HUMAN.H11MO.1.D<br>Description: ZN554_HUMAN<br>Possible TFs: ZNF554	*4.50957*	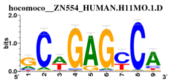	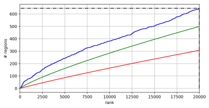	link	link	PWMs
200	☐ ENCFF001TST<br>Description: NR3C1 ChIP-seq (protocol PCR2x) on A549 treated with dexamethasone at 100nM	*4.50624*		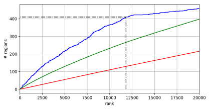	link	link	TF binding sites
201	☐ cisbp__M2290<br>Description: JUN[gene ID: "ENSG00000177606" species: "Homo sapiens" TF status: "direct" TF family: "bZIP" DBDs: "bZIP_1"]<br>Possible TFs: JUN	*4.50208*	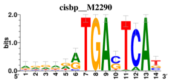	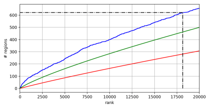	link	link	PWMs
202	☐ dbcorrdb__FOSL2__ENCSR000BQO_1__m1<br>Description: FOSL2 (ENCSR000BQO-1, motif 1)<br>Possible TFs: FOSL2	*4.49304*	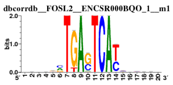	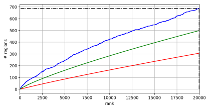	link	link	PWMs
203	☐ dbcorrdb__SIN3A__ENCSR000BRM_1__m1<br>Description: SIN3A (ENCSR000BRM-1, motif 1)<br>Possible TFs: SIN3A	*4.49246*	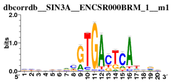	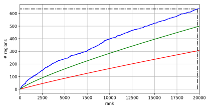	link	link	PWMs
204	☐ cisbp__M6228<br>Description: FOSB[gene ID: "ENSG00000125740" species: "Homo sapiens" TF status: "direct" TF family: "bZIP" DBDs: "bZIP_1"]; Fos[gene ID: "ENSMUSG00000021250" species: "Mus musculus" TF status: "inferred" TF family: "bZIP" DBDs: "bZIP_1"]; Fosb[gene ID: "ENSMUSG00000003545" species: "Mus musculus" TF status: "inferred" TF family: "bZIP" DBDs: "bZIP_1"]<br>Possible TFs: FOSB	*4.48454*	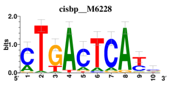	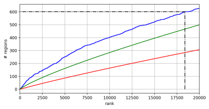	link	link	PWMs
205	☐ homer__TGCTGAGTCA_Bach2<br>Description: Bach2(bZIP)/OCILy7-Bach2-ChIP-Seq(GSE44420)/Homer<br>Possible TFs: BACH2	*4.46786*	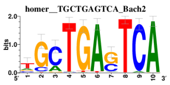	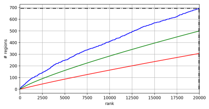	link	link	PWMs

#	Feature	NES	Logo	Recovery Curve	Candidate targets	All regions in top 20000	Database
206	☐ neph__UW.Motif.0001<br>Description: atgactca	4.46652	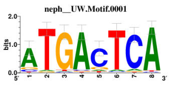	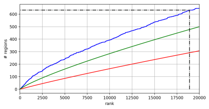	link	link	PWMs
207	☐ transfac_pro__M08940<br>Description: V$JUNFRA2_01: C-JUN:FRA-2<br>Possible TFs: FOSL2	4.44699	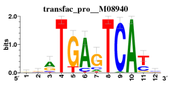	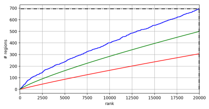	link	link	PWMs
208	☐ E104-H3K27ac<br>Description: H3K27ac in Right Atrium (E104,)	4.44276		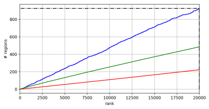	link	link	Histone modifications
209	☐ ENCFF001TSP<br>Description: NR3C1 ChIP-seq (protocol PCR1x) on A549 treated with dexamethasone at 50nM	4.42660		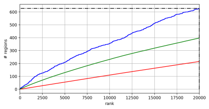	link	link	TF binding sites
210	☐ homer__GKVTCADRTTWC_Six1<br>Description: Six1(Homeobox)/Myoblast-Six1-ChIP-Chip(GSE20150)/Homer<br>Possible TFs: SIX1	4.42648	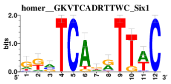	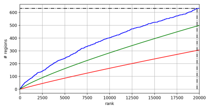	link	link	PWMs
211	☐ dbcorrdb__JUN__ENCSR000EZX_1__m1<br>Description: JUN (ENCSR000EZX-1, motif 1)<br>Possible TFs: JUN	4.42495	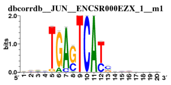	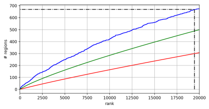	link	link	PWMs
212	☐ transfac_pro__M00925<br>Description: V$AP1_Q6_01: AP-1<br>Possible TFs: FOS, FOSB, JUNB, JUN, JUND, FOSL1, FOSL2	4.41776	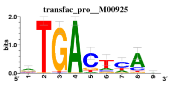	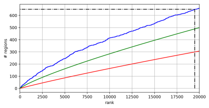	link	link	PWMs
213	☐ transfac_pro__M07602<br>Description: V$CP2_Q4: CP2<br>Possible TFs: TFCP2	4.40039	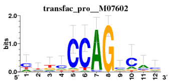	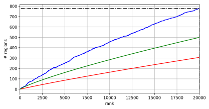	link	link	PWMs
214	☐ jaspar__MA0099.2<br>Description: FOS::JUN<br>Possible TFs: JUN	4.40033	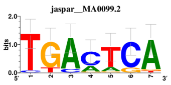	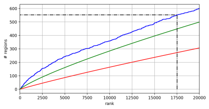	link	link	PWMs
215	☐ dbcorrdb__STAT3__ENCSR000EDC_1__m1<br>Description: STAT3 (ENCSR000EDC-1, motif 1)<br>Possible TFs: STAT3	4.39999	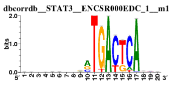	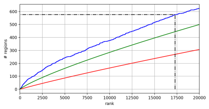	link	link	PWMs
216	☐ transfac_pro__M08942<br>Description: V$FOSJUN_02: C-FOS:C-JUN<br>Possible TFs: JUN	4.39982	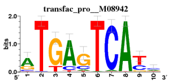	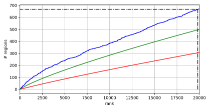	link	link	PWMs
217	☐ transfac_pro__M08936<br>Description: V$JUNFOSB_01: C-JUN:FOSB<br>Possible TFs: FOSB	4.39923	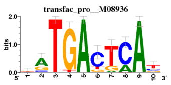	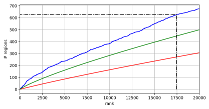	link	link	PWMs
218	☐ E108-H3K27ac-broadpeak<br>Description: H3K27ac in Skeletal Muscle Female (E108, broadpeak)	4.38153		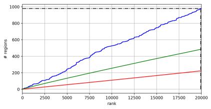	link	link	Histone modifications
219	☐ cisbp__M4531<br>Description: JUN[gene ID: "ENSG00000177606" species: "Homo sapiens" TF status: "direct" TF family: "bZIP" DBDs: "bZIP_1"]<br>Possible TFs: JUN	4.37390	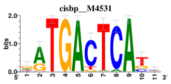	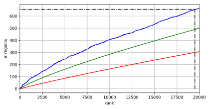	link	link	PWMs

#	Feature	NES	Logo	Recovery Curve	Candidate targets	All regions in top 20000	Database
220	☐ jaspar__MA0476.1<br>Description: FOS<br>Possible TFs: FOS	*4.36586*	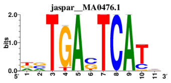	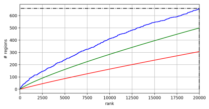	**link**	**link**	PWMs
221	☐ ENCFF001WAY<br>Description: DNase-seq on human HSMM	*4.36211*		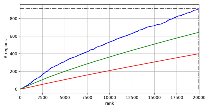	**link**	**link**	DHS & FAIRE
222	☐ dbcorrdb__TCF12__ENCSR000BQQ_1__m2<br>Description: TCF12 (ENCSR000BQQ-1, motif 2)<br>Possible TFs: TCF12	*4.36062*	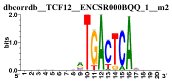	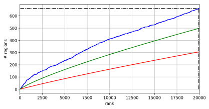	**link**	**link**	PWMs
223	☐ jaspar__MA0491.1<br>Description: JUND<br>Possible TFs: JUND	*4.35557*	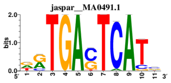	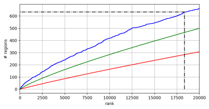	**link**	**link**	PWMs
224	☐ cisbp__M2292<br>Description: JUND[gene ID: "ENSG00000130522" species: "Homo sapiens" TF status: "direct" TF family: "bZIP" DBDs: "bZIP_1"]<br>Possible TFs: JUND	*4.34501*	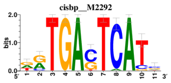	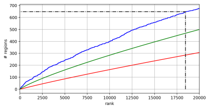	**link**	**link**	PWMs
225	☐ ENCFF001TSS<br>Description: NR3C1 ChIP-seq (protocol PCR1x) on A549 treated with dexamethasone at 5nM	*4.34195*		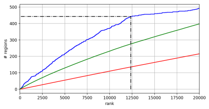	**link**	**link**	TF binding sites
226	☐ transfac_pro__M08933<br>Description: V$JUNDFOS_01: JUND:C-FOS<br>Possible TFs: JUND	*4.33050*	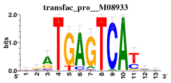	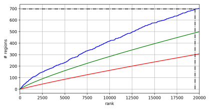	**link**	**link**	PWMs
227	☐ stark__TGANTCA<br>Description: activating-protein 1 (FOS-JUN heterodimer)<br>Possible TFs: FOS, FOSB, JUNB, JUN, JUND, FOSL1, FOSL2	*4.31790*	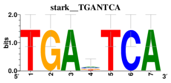	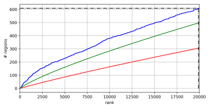	**link**	**link**	PWMs
228	☐ ENCFF001VHS<br>Description: SMARCC1 ChIP-seq on human HeLa-S3	*4.31051*		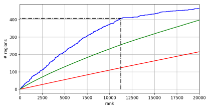	**link**	**link**	TF binding sites
229	☐ dbcorrdb__JUND__ENCSR000EIB_1__m1<br>Description: JUND (ENCSR000EIB-1, motif 1)<br>Possible TFs: JUND	*4.29061*	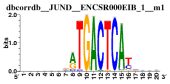	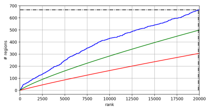	**link**	**link**	PWMs
230	☐ ENCFF001TVA<br>Description: NR3C1 ChIP-seq (protocol v041610.2) on ECC-1 treated with dexamethasone at 100 nM. Note- This experiment previously referred to its biosample as ECC-1, however it has been found that all currently available ECC-1 are actually Ishikawa cells.	*4.28711*		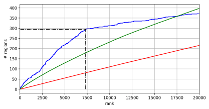	**link**	**link**	TF binding sites
231	☐ dbcorrdb__FOS__ENCSR000DOO_1__m1<br>Description: FOS (ENCSR000DOO-1, motif 1)<br>Possible TFs: FOS	*4.28025*	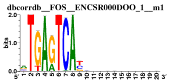	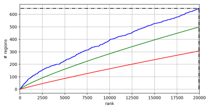	**link**	**link**	PWMs
232	☐ cisbp__M0352<br>Description: GCN4[gene ID: "YEL009C" species: "Saccharomyces cerevisiae" TF status: "direct" TF family: "bZIP" DBDs: "bZIP_1"]	*4.27461*	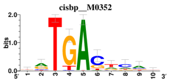	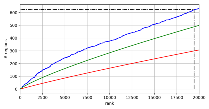	**link**	**link**	PWMs

#	Feature	NES	Logo	Recovery Curve	Candidate targets	All regions in top 20000	Database
233	☐ neph__UW.Motif.0034<br>Description: taaaaata	4.27418	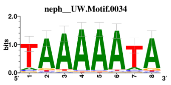	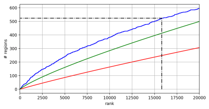	link	link	PWMs
234	☐ swissregulon__hs__NFE2.p2<br>Description: hs__NFE2.p2<br>Possible TFs: NFE2	4.27391	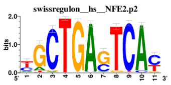	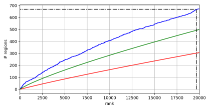	link	link	PWMs
235	☐ taipale_cyt_meth__ZNF32_NYGTAACNYGAYACN_FL_meth_repr<br>Description: ZNF32 [Znf_C2H2, CpG-meth]<br>Possible TFs: ZNF32	4.27382	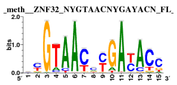	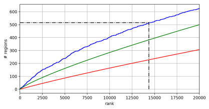	link	link	PWMs
236	☐ dbcorrdb__STAT3__ENCSR000DPB_1__m2<br>Description: STAT3 (ENCSR000DPB-1, motif 2)<br>Possible TFs: STAT3	4.26975	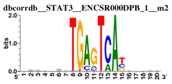	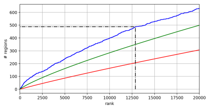	link	link	PWMs
237	☐ dbcorrdb__JUN__ENCSR000EFA_1__m1<br>Description: JUN (ENCSR000EFA-1, motif 1)<br>Possible TFs: JUN	4.26659	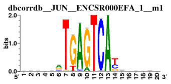	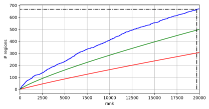	link	link	PWMs
238	☐ E065-H3K27ac<br>Description: H3K27ac in Aorta (E065,)	4.25224		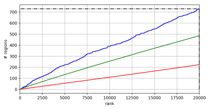	link	link	Histone modifications
239	☐ ENCFF001WMT<br>Description: DNase-seq on human HSMM	4.24895		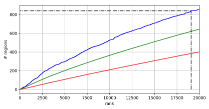	link	link	DHS & FAIRE
240	☐ transfac_pro__M08920<br>Description: V$JUNBFOSB_01: JUNB:FOSB<br>Possible TFs: JUNB	4.24636	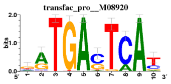	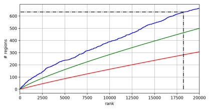	link	link	PWMs
241	☐ dbcorrdb__JUN__ENCSR000EZX_1__m2<br>Description: JUN (ENCSR000EZX-1, motif 2)<br>Possible TFs: JUN	4.24038	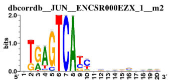	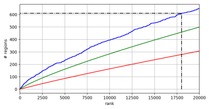	link	link	PWMs
242	☐ dbcorrdb__FOS__ENCSR000DOP_1__m1<br>Description: FOS (ENCSR000DOP-1, motif 1)<br>Possible TFs: FOS	4.23949	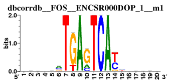	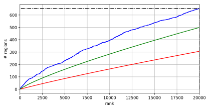	link	link	PWMs
243	☐ ENCFF001TUU<br>Description: ESR1 ChIP-seq protocol v041610.2 on human ECC-1 treated with 100 nM Genistein. Note- This experiment previously referred to its biosample as ECC-1, however it has been found that all currently available ECC-1 are actually Ishikawa cells.	4.23492		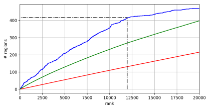	link	link	TF binding sites
244	☐ dbcorrdb__JUND__ENCSR000BKP_1__m1<br>Description: JUND (ENCSR000BKP-1, motif 1)<br>Possible TFs: JUND	4.23103	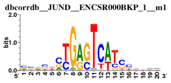	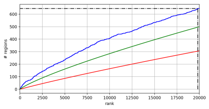	link	link	PWMs
245	☐ hdpi__MTHFD1<br>Description: MTHFD1<br>Possible TFs: MTHFD1	4.22257	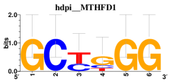	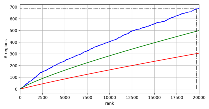	link	link	PWMs

#	Feature	NES	Logo	Recovery Curve	Candidate targets	All regions in top 20000	Database
246	☐ hocomoco__SIX4_MOUSE.H11MO.0.C<br>Description: SIX4_MOUSE<br>Possible TFs: SIX4	*4.21214*	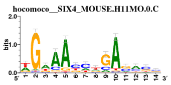	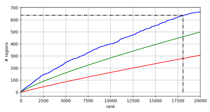	link	link	PWMs
247	☐ E100-DNase.hotspot.all.peaks-narrowpeak<br>Description: DNase in Psoas Muscle (E100, narrowpeak, hotspot)	*4.20596*		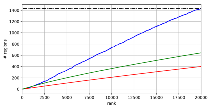	link	link	DHS & FAIRE
248	☐ dbcorrdb__JUN__ENCSR000EDG_1__m1<br>Description: JUN (ENCSR000EDG-1, motif 1)<br>Possible TFs: JUN	*4.20567*	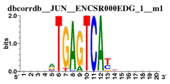	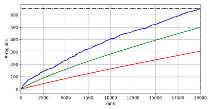	link	link	PWMs
249	☐ transfac_pro__M08922<br>Description: V$JUNBFRA1_01: JUNB:FRA-1<br>Possible TFs: JUNB	*4.20146*	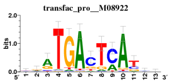	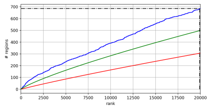	link	link	PWMs
250	☐ transfac_pro__M01033<br>Description: V$HNF4_Q6_03: HNF-4<br>Possible TFs: HNF4A	*4.17184*	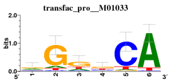	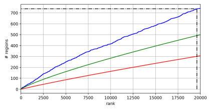	link	link	PWMs
251	☐ transfac_pro__M08931<br>Description: V$JUNDFRA2_01: JUND:FRA-2<br>Possible TFs: FOSL2	*4.15807*	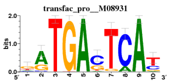	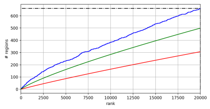	link	link	PWMs
252	☐ scertf__macisaac.RTG3<br>Description: macisaac.RTG3	*4.15111*	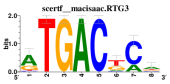	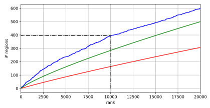	link	link	PWMs
253	☐ hdpi__PIR<br>Description: PIR<br>Possible TFs: PIR	*4.14670*	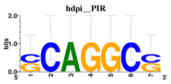	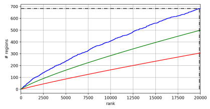	link	link	PWMs
254	☐ cisbp__M4591<br>Description: JUN[gene ID: "ENSG00000177606" species: "Homo sapiens" TF status: "direct" TF family: "bZIP" DBDs: "bZIP_1"]<br>Possible TFs: JUN	*4.13647*	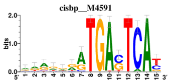	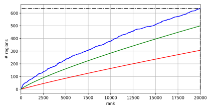	link	link	PWMs
255	☐ transfac_pro__M08925<br>Description: V$JUNBFOS_01: JUNB:C-FOS<br>Possible TFs: JUNB	*4.13204*	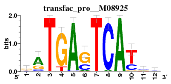	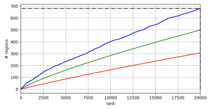	link	link	PWMs
256	☐ transfac_pro__M04850<br>Description: V$RAD21_10: Rad21<br>Possible TFs: RAD21	*4.12305*	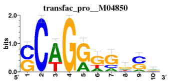	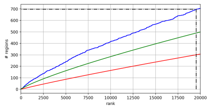	link	link	PWMs
257	☐ dbcorrdb__JUND__ENCSR000EDH_1__m1<br>Description: JUND (ENCSR000EDH-1, motif 1)<br>Possible TFs: JUND	*4.11962*	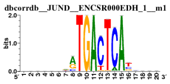	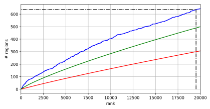	link	link	PWMs
258	☐ transfac_pro__M08938<br>Description: V$JUNFRA1_01: C-JUN:FRA-1<br>Possible TFs: FOSL1	*4.10306*	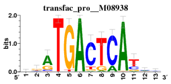	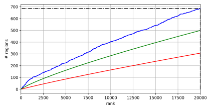	link	link	PWMs
259	☐ dbcorrdb__FOS__ENCSR000DOT_1__m1<br>Description: FOS (ENCSR000DOT-1, motif 1)<br>Possible TFs: FOS	*4.09724*	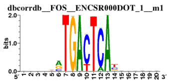	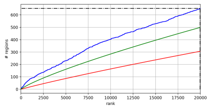	link	link	PWMs

#	Feature	NES	Logo	Recovery Curve	Candidate targets	All regions in top 20000	Database
260	☐ dbcorrdb__FOS__ENCSR000DON_1__m1<br>Description: FOS (ENCSR000DON-1, motif 1)<br>Possible TFs: FOS	*4.08206*	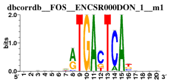	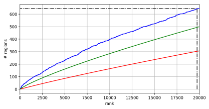	**link**	**link**	PWMs
261	☐ ENCFF001TSR<br>Description: NR3C1 ChIP-seq (protocol PCR1x) on A549 treated with dexamethasone at 5nM	*4.07521*		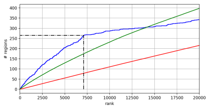	**link**	**link**	TF binding sites
262	☐ E125-H3K4me1<br>Description: H3K4me1 in NH-A Astrocytes Primary Cells (E125,)	*4.07182*		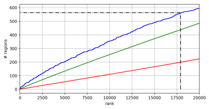	**link**	**link**	Histone modifications
263	☐ hocomoco__BACH2_MOUSE.H11MO.0.A<br>Description: BACH2_MOUSE<br>Possible TFs: BACH2	*4.07159*	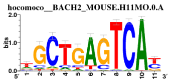	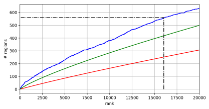	**link**	**link**	PWMs
264	☐ ENCFF001TUZ<br>Description: NR3C1 ChIP-seq (protocol v041610.2) on ECC-1 treated with dexamethasone at 100 nM. Note- This experiment previously referred to its biosample as ECC-1, however it has been found that all currently available ECC-1 are actually Ishikawa cells.	*4.04818*		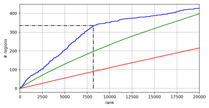	**link**	**link**	TF binding sites
265	☐ ENCFF001TUR<br>Description: ESR1 ChIP-seq (protocol v041610.2) on ECC-1 treated with estradiol at 10nM. Note- This experiment previously referred to its biosample as ECC-1, however it has been found that all currently available ECC-1 are actually Ishikawa cells.	*4.03626*		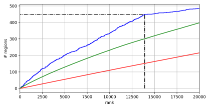	**link**	**link**	TF binding sites
266	☐ transfac_pro__M08923<br>Description: V$JUNBFRA2_01: JUNB:FRA-2<br>Possible TFs: JUNB	*4.03557*	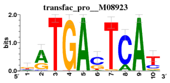	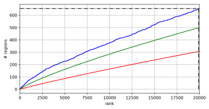	**link**	**link**	PWMs
267	☐ hdpi__NXPH3<br>Description: NXPH3<br>Possible TFs: NXPH3	*4.02614*	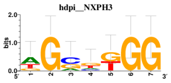	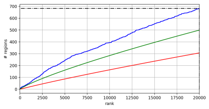	**link**	**link**	PWMs
268	☐ transfac_public__M00188<br>Description: V$AP1_Q4: AP-1<br>Possible TFs: JUN, FOS	*4.02420*	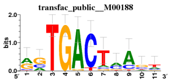	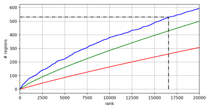	**link**	**link**	PWMs
269	☐ transfac_pro__M00924<br>Description: V$AP1_Q2_01: AP-1<br>Possible TFs: FOS, FOSB, JUNB, JUN, JUND, FOSL1, FOSL2	*4.01348*	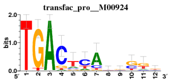	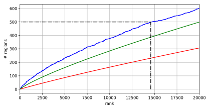	**link**	**link**	PWMs
270	☐ yetfasco__YER064C_2094<br>Description: YER064C_2094	*4.01174*	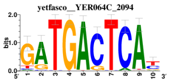	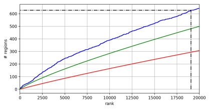	**link**	**link**	PWMs
271	☐ transfac_pro__M04931<br>Description: V$TCF12_05: HTF4<br>Possible TFs: TCF12	*3.98133*	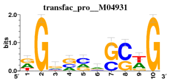	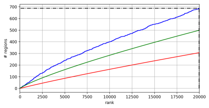	**link**	**link**	PWMs
272	☐ cisbp__M0344<br>Description: M0344	*3.96364*	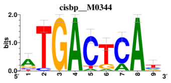	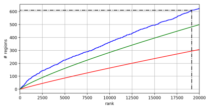	**link**	**link**	PWMs

#	Feature	NES	Logo	Recovery Curve	Candidate targets	All regions in top 20000	Database
273	☐ ENCFF001TUT<br>Description: ESR1 ChIP-seq protocol v041610.2 on human ECC-1 treated with 100 nM Genistein. Note- This experiment previously referred to its biosample as ECC-1, however it has been found that all currently available ECC-1 are actually Ishikawa cells.	*3.95718*		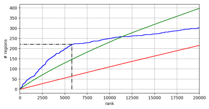	link	link	TF binding sites
274	☐ E076-H3K4me1<br>Description: H3K4me1 in Colon Smooth Muscle (E076,)	*3.94529*		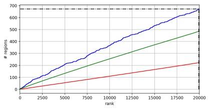	link	link	Histone modifications
275	☐ transfac_public__M00037<br>Description: V$NFE2_01: NF-E2<br>Possible TFs: MAFK, NFE2	*3.92568*	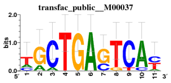	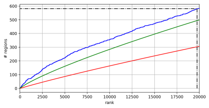	link	link	PWMs
276	☐ transfac_pro__M04904<br>Description: V$POLR3A_01: POLR3A<br>Possible TFs: POLR3A	*3.88470*	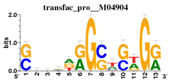	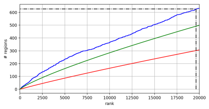	link	link	PWMs
277	☐ E090-H3K4me1-broadpeak<br>Description: H3K4me1 in Fetal Muscle Leg (E090, broadpeak)	*3.88173*		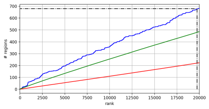	link	link	Histone modifications
278	☐ transfac_pro__M02022<br>Description: V$MAFK_Q3: MafK<br>Possible TFs: MAFK	*3.87215*	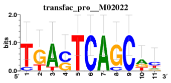	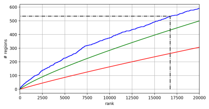	link	link	PWMs
279	☐ transfac_pro__M07296<br>Description: V$MAF_Q4: MAF<br>Possible TFs: NFE2L2, BACH2, BACH1, MAFK, NFE2, MAF, MAFB, NRF1, NFE2L1, MAFG	*3.85956*	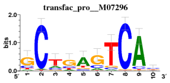	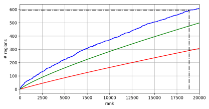	link	link	PWMs
280	☐ E076-H3K4me1-broadpeak<br>Description: H3K4me1 in Colon Smooth Muscle (E076, broadpeak)	*3.84956*		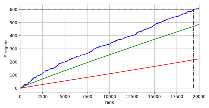	link	link	Histone modifications
281	☐ transfac_pro__M03554<br>Description: V$NF1A_Q6_01: NF-1A<br>Possible TFs: NFIA	*3.83957*	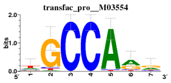	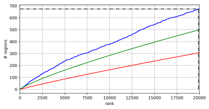	link	link	PWMs
282	☐ E013-H3K4me1<br>Description: H3K4me1 in hESC Derived CD56+ Mesoderm Cultured Cells (E013,)	*3.83550*		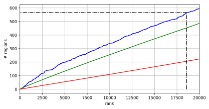	link	link	Histone modifications
283	☐ E103-H3K4me1-broadpeak<br>Description: H3K4me1 in Rectal Smooth Muscle (E103, broadpeak)	*3.80677*		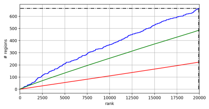	link	link	Histone modifications
284	☐ c2h2_zfs__M0442<br>Description: NCU05064	*3.80473*	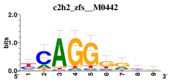	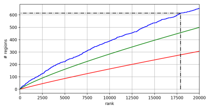	link	link	PWMs
285	☐ ENCFF001WMU<br>Description: DNase-seq on human HSMM	*3.79752*		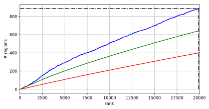	link	link	DHS & FAIRE

#	Feature	NES	Logo	Recovery Curve	Candidate targets	All regions in top 20000	Database
286	☐ cisbp__M4014<br>Description: TBPL2[gene ID: "ENSG00000182521" species: "Homo sapiens" TF status: "inferred" TF family: "TBP" DBDs: "TBP"]; TBP[gene ID: "ENSG00000112592" species: "Homo sapiens" TF status: "direct" TF family: "TBP" DBDs: "TBP"]; Tbp[gene ID: "FBgn0003687" species: "Drosophila melanogaster" TF status: "inferred" TF family: "TBP" DBDs: "TBP"]; Tbpl2[gene ID: "ENSMUSG00000061809" species: "Mus musculus" TF status: "inferred" TF family: "TBP" DBDs: "TBP"]<br>Possible TFs: TBP	*3.78300*	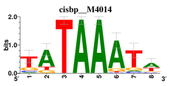	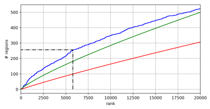	link	link	PWMs
287	☐ hocomoco__NFIB_HUMAN.H11MO.0.D<br>Description: NFIB_HUMAN<br>Possible TFs: NFIB	*3.78245*	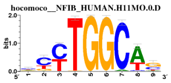	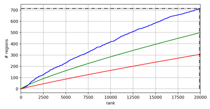	link	link	PWMs
288	☐ transfac_pro__M08914<br>Description: V$FRA2_01: FRA-2<br>Possible TFs: FOSL2	*3.76964*	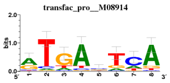	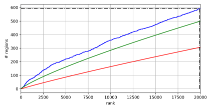	link	link	PWMs
289	☐ transfac_pro__M02026<br>Description: V$MEF2D_Q4: MEF-2D<br>Possible TFs: MEF2D	*3.76926*	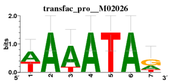	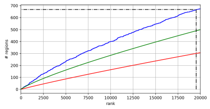	link	link	PWMs
290	☐ ENCFF001TUS<br>Description: ESR1 ChIP-seq (protocol v041610.2) on ECC-1 treated with estradiol at 10nM. Note- This experiment previously referred to its biosample as ECC-1, however it has been found that all currently available ECC-1 are actually Ishikawa cells.	*3.76623*		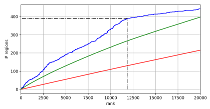	link	link	TF binding sites
291	☐ ENCFF001UPC<br>Description: FOSL2 ChIP-seq protocol v042211.1 on human SK-N-SH	*3.75748*		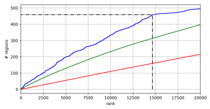	link	link	TF binding sites
292	☐ E111-H3K4me1-broadpeak<br>Description: H3K4me1 in Stomach Smooth Muscle (E111, broadpeak)	*3.75324*		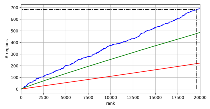	link	link	Histone modifications
293	☐ transfac_pro__M08929<br>Description: V$JUNDFRA1_01: JUND:FRA-1<br>Possible TFs: FOSL1	*3.74521*	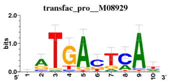	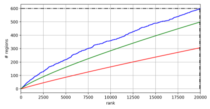	link	link	PWMs
294	☐ transfac_public__M00471<br>Description: V$TBP_01: TBP<br>Possible TFs: TBP	*3.74487*	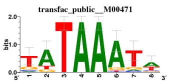	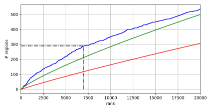	link	link	PWMs
295	☐ transfac_pro__M08944<br>Description: V$JUNBFOS_02: JUNB:C-FOS<br>Possible TFs: JUNB	*3.74249*	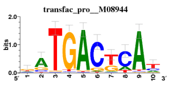	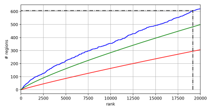	link	link	PWMs
296	☐ hdpi__AKR1A1<br>Description: AKR1A1<br>Possible TFs: AKR1A1	*3.73798*	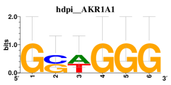	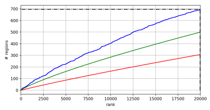	link	link	PWMs
297	☐ E107-H3K36me3-broadpeak<br>Description: H3K36me3 in Skeletal Muscle Male (E107, broadpeak)	*3.73026*		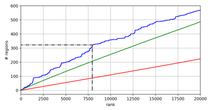	link	link	Histone modifications

#	Feature	NES	Logo	Recovery Curve	Candidate targets	All regions in top 20000	Database
298	dbcorrdb__EP300__ENCSR000BHB_1__m2<br>Description: EP300 (ENCSR000BHB-1, motif 2)<br>Possible TFs: EP300	*3.69778*	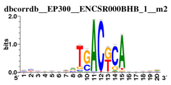	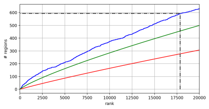	link	link	PWMs
299	dbcorrdb__JUND__ENCSR000EBZ_1__m2<br>Description: JUND (ENCSR000EBZ-1, motif 2)<br>Possible TFs: JUND	*3.68275*	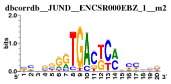	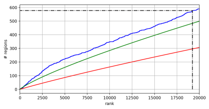	link	link	PWMs
300	cisbp__M2108<br>Description: GCN4[gene ID: "YEL009C" species: "Saccharomyces cerevisiae" TF status: "direct" TF family: "bZIP" DBDs: "bZIP_1"]	*3.67270*	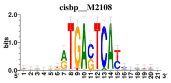	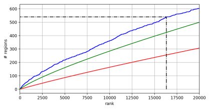	link	link	PWMs
301	transfac_pro__M01555<br>Description: F$GCN4_02: Gcn4p	*3.67261*	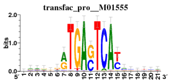	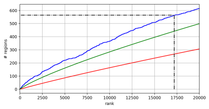	link	link	PWMs
302	ENCFF001WBM<br>Description: UW human LHCN-M2 DNase-seq	*3.67256*		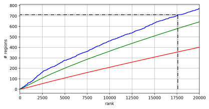	link	link	DHS & FAIRE
303	transfac_pro__M07051<br>Description: V$NF1B_Q6: NF-1B<br>Possible TFs: NFIB	*3.66559*	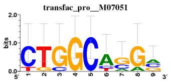	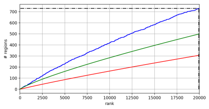	link	link	PWMs
304	ENCFF001WBK<br>Description: DNase-seq on human LHCN-M2 differentiated for 4 days	*3.66440*		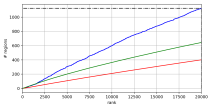	link	link	DHS & FAIRE
305	scertf__macisaac.ARG81<br>Description: macisaac.ARG81	*3.66076*	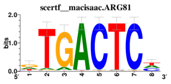	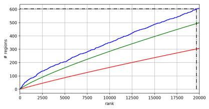	link	link	PWMs
306	transfac_pro__M00629<br>Description: I$EVE_Q6: Eve<br>Possible TFs: EVX1, EVX2	*3.65844*	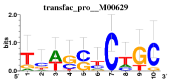	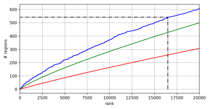	link	link	PWMs
307	hocomoco__SMAD3_HUMAN.H11MO.0.B<br>Description: SMAD3_HUMAN<br>Possible TFs: SMAD3	*3.64529*	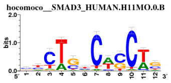	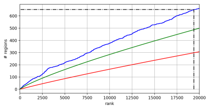	link	link	PWMs
308	cisbp__M2077<br>Description: ARG81[gene ID: "YML099C" species: "Saccharomyces cerevisiae" TF status: "direct" TF family: "Zinc cluster" DBDs: "Zn_clus"]	*3.64391*	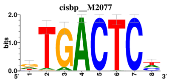	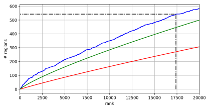	link	link	PWMs
309	transfac_pro__M00963<br>Description: V$T3R_Q6: T3R<br>Possible TFs: RARA, RARB, RARG, RXRG, THRB, RXRA, RXRB, THRA	*3.63852*	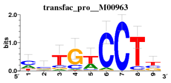	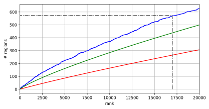	link	link	PWMs
310	jaspar__MA0272.1<br>Description: ARG81	*3.63425*	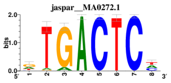	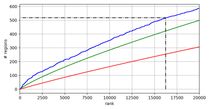	link	link	PWMs
311	scertf__spivak.GCN4<br>Description: spivak.GCN4	*3.61701*	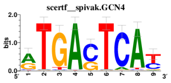	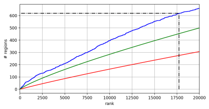	link	link	PWMs

#	Feature	NES	Logo	Recovery Curve	Candidate targets	All regions in top 20000	Database
312	☐ E099-H3K4me1<br>Description: H3K4me1 in Placenta Amnion (E099,)	*3.61475*		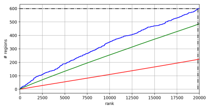	link	link	Histone modifications
313	☐ swissregulon__hs__NFE2L2.p2<br>Description: hs__NFE2L2.p2<br>Possible TFs: NFE2L2	*3.61130*	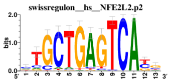	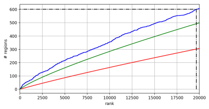	link	link	PWMs
314	☐ dbcorrdb__NR3C1__ENCSR000BJR_1__m1<br>Description: NR3C1 (ENCSR000BJR-1, motif 1)<br>Possible TFs: NR3C1	*3.61095*	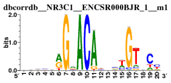	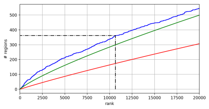	link	link	PWMs
315	☐ cisbp__M4010<br>Description: TBPL2[gene ID: "ENSG00000182521" species: "Homo sapiens" TF status: "inferred" TF family: "TBP" DBDs: "TBP"]; TBP[gene ID: "ENSG00000112592" species: "Homo sapiens" TF status: "direct" TF family: "TBP" DBDs: "TBP"]; Tbp[gene ID: "FBgn0003687" species: "Drosophila melanogaster" TF status: "inferred" TF family: "TBP" DBDs: "TBP"]; Tbpl2[gene ID: "ENSMUSG00000061809" species: "Mus musculus" TF status: "inferred" TF family: "TBP" DBDs: "TBP"]<br>Possible TFs: TBP	*3.60782*	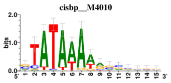	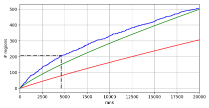	link	link	PWMs
316	☐ jaspar__MA0108.2<br>Description: TBP	*3.60459*	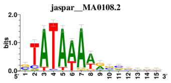	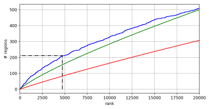	link	link	PWMs
317	☐ E089-H3K4me1<br>Description: H3K4me1 in Fetal Muscle Trunk (E089,)	*3.59562*		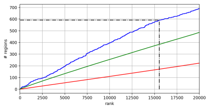	link	link	Histone modifications
318	☐ predrem__nrMotif728<br>Description: 251_fThymus-DS18382.M596	*3.59224*	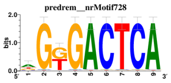	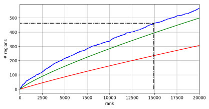	link	link	PWMs
319	☐ dbcorrdb__JUN__ENCSR000ECA_1__m1<br>Description: JUN (ENCSR000ECA-1, motif 1)<br>Possible TFs: JUN	*3.58248*	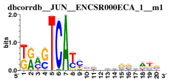	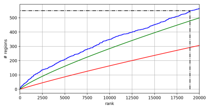	link	link	PWMs
320	☐ ENCFF001UPO<br>Description: MEF2A ChIP-seq protocol v042211.1 on human SK-N-SH	*3.57934*		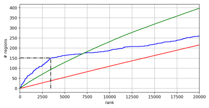	link	link	TF binding sites
321	☐ jaspar__MA0303.1<br>Description: GCN4	*3.56817*	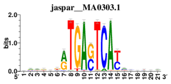	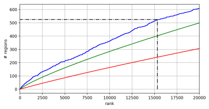	link	link	PWMs
322	☐ ENCFF001WPG<br>Description: UW human LHCN-M2 DNase-seq	*3.56458*		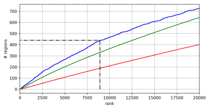	link	link	DHS & FAIRE
323	☐ yetfasco__YKR099W_402<br>Description: YKR099W_402<br>Possible TFs: MYBL1, MYB, MYBL2, SNAPC4	*3.55768*	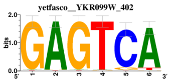	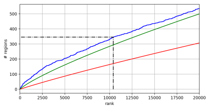	link	link	PWMs

#	Feature	NES	Logo	Recovery Curve	Candidate targets	All regions in top 20000	Database
324	☐ transfac_pro__M03868<br>Description: V$CP2_Q6: CP2<br>Possible TFs: TFCP2	*3.55563*	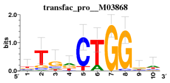	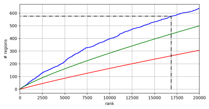	link	link	PWMs
325	☐ cisbp__M0322<br>Description: M0322	*3.53999*	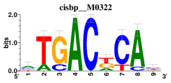	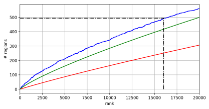	link	link	PWMs
326	☐ yetfasco__YBL103C_1445<br>Description: YBL103C_1445	*3.53900*	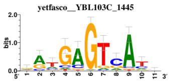	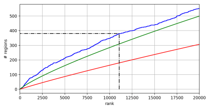	link	link	PWMs
327	☐ ENCFF001WUY<br>Description: H3K27ac ChIP-seq on human AG04450	*3.53093*		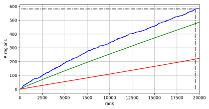	link	link	Histone modifications
328	☐ swissregulon__sacCer__GCN4<br>Description: sacCer__GCN4	*3.52935*	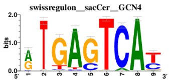	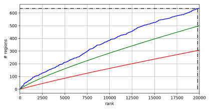	link	link	PWMs
329	☐ E105-H3K4me1<br>Description: H3K4me1 in Right Ventricle (E105,)	*3.52481*		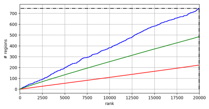	link	link	Histone modifications
330	☐ homer__NAGAACAGNCTGTTCT_GRE<br>Description: GRE(NR),IR3/A549-GR-ChIP-Seq(GSE32465)/Homer	*3.51468*	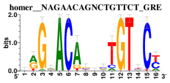	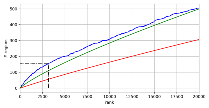	link	link	PWMs
331	☐ cisbp__M4541<br>Description: JUND[gene ID: "ENSG00000130522" species: "Homo sapiens" TF status: "direct" TF family: "bZIP" DBDs: "bZIP_1"]<br>Possible TFs: JUND	*3.50730*	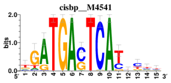	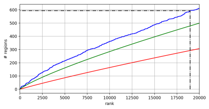	link	link	PWMs
332	☐ cisbp__M0365<br>Description: M0365	*3.50498*	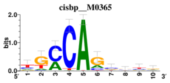	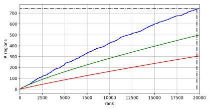	link	link	PWMs
333	☐ dbcorrdb__NR3C1__ENCSR000BHF_1__m1<br>Description: NR3C1 (ENCSR000BHF-1, motif 1)<br>Possible TFs: NR3C1	*3.49447*	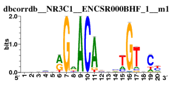	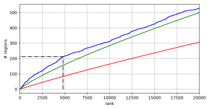	link	link	PWMs
334	☐ cisbp__M2979<br>Description: BACH1[gene ID: "ENSG00000156273" species: "Homo sapiens" TF status: "direct" TF family: "bZIP" DBDs: "bZIP_1"]; Bach1[gene ID: "ENSMUSG00000025612" species: "Mus musculus" TF status: "inferred" TF family: "bZIP" DBDs: "bZIP_1"]; Bach2[gene ID: "ENSMUSG00000040270" species: "Mus musculus" TF status: "inferred" TF family: "bZIP" DBDs: "bZIP_1"]<br>Possible TFs: BACH1	*3.48061*	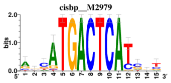	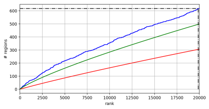	link	link	PWMs
335	☐ transfac_pro__M00983<br>Description: V$MAF_Q6_01: MAF<br>Possible TFs: NFE2L2, NFE2L3, BACH2, BACH1, MAFK, NFE2, MAF, MAFB, NFE2L1, MAFF, MAFG	*3.47618*	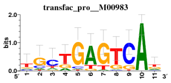	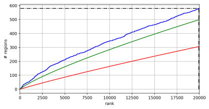	link	link	PWMs
336	☐ ENCFF001UJY<br>Description: FOSL1 ChIP-seq protocol v041610.1 on human K562	*3.47162*		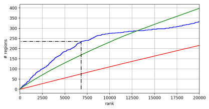	link	link	TF binding sites

#	Feature	NES	Logo	Recovery Curve	Candidate targets	All regions in top 20000	Database
337	☐ hocomoco__NFIB_MOUSE.H11MO.0.C<br>Description: NFIB_MOUSE<br>Possible TFs: NFIB	*3.47064*	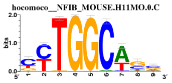	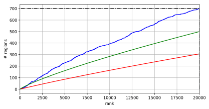	link	link	PWMs
338	☐ dbcorrdb__POLR2AphosphoS5__ENCSR000BOV_1__m3<br>Description: POLR2AphosphoS5 (ENCSR000BOV-1, motif 3)	*3.46476*	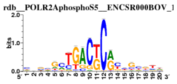	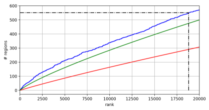	link	link	PWMs
339	☐ dbcorrdb__SMARCC2__ENCSR000EDL_1__m4<br>Description: SMARCC2 (ENCSR000EDL-1, motif 4)<br>Possible TFs: SMARCC2	*3.46403*	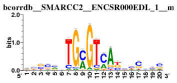	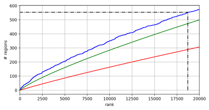	link	link	PWMs
340	☐ swissregulon__sacCer__NHP6A<br>Description: sacCer__NHP6A<br>Possible TFs: HMGB3, HMGB2, HMGB1, HMGB4	*3.45787*	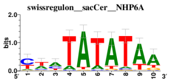	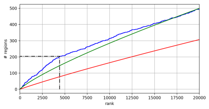	link	link	PWMs
341	☐ transfac_pro__M08934<br>Description: V$JUND_06: JUND<br>Possible TFs: JUND	*3.45274*	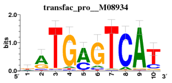	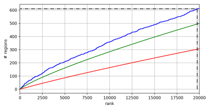	link	link	PWMs
342	☐ transfac_pro__M01687<br>Description: F$ARGRII_01: ARG RII	*3.44257*	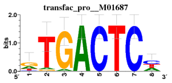	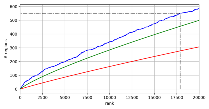	link	link	PWMs
343	☐ E096-H3K4me1<br>Description: H3K4me1 in Lung (E096,)	*3.44196*		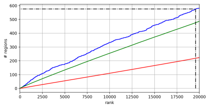	link	link	Histone modifications
344	☐ neph__UW.Motif.0032<br>Description: cagcccag	*3.44177*	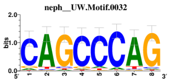	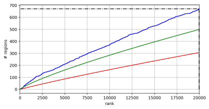	link	link	PWMs
345	☐ ENCFF001UMR<br>Description: FOSL2 ChIP-seq protocol v042211.1 on human MCF-7	*3.44117*		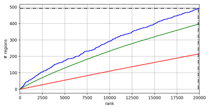	link	link	TF binding sites
346	☐ E097-H3K27ac<br>Description: H3K27ac in Ovary (E097,)	*3.43472*		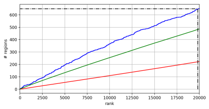	link	link	Histone modifications
347	☐ factorbook__NR3C1<br>Description: NR3C1<br>Possible TFs: NR3C1	*3.42713*	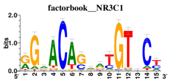	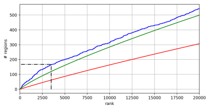	link	link	PWMs
348	☐ ENCFF001TVI<br>Description: EP300 ChIP-seq protocol v042211.1 on human ECC-1. Note- This experiment previously referred to its biosample as ECC-1, however it has been found that all currently available ECC-1 are actually Ishikawa cells.	*3.42607*		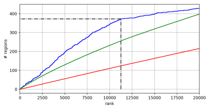	link	link	TF binding sites
349	☐ predrem__nrMotif1768<br>Description: 174_fMuscle_arm-DS19295.M321	*3.42466*	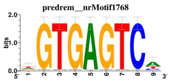	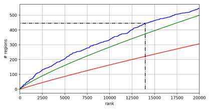	link	link	PWMs
350	☐ E080-H3K4me1<br>Description: H3K4me1 in Fetal Adrenal Gland (E080,)	*3.42214*		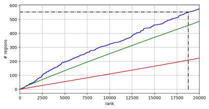	link	link	Histone modifications

#	Feature	NES	Logo	Recovery Curve	Candidate targets	All regions in top 20000	Database
351	☐ transfac_pro__M03541<br>Description: V$CJUN_Q6: C-Jun<br>Possible TFs: JUN	*3.42007*	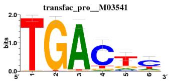	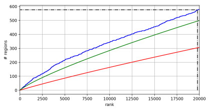	link	link	PWMs
352	☐ ENCFF001WMX<br>Description: DNase-seq on human HSMMtube	*3.41871*		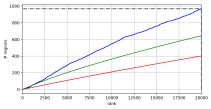	link	link	DHS & FAIRE
353	☐ cisbp__M3550<br>Description: AC002126.6[gene ID: "ENSG00000064489" species: "Homo sapiens" TF status: "inferred" TF family: "MADS box" DBDs: "SRF-TF"]; MEF2A[gene ID: "ENSG00000068305" species: "Homo sapiens" TF status: "direct" TF family: "MADS box" DBDs: "SRF-TF"]; Mef2a[gene ID: "ENSMUSG00000030557" species: "Mus musculus" TF status: "inferred" TF family: "MADS box" DBDs: "SRF-TF"]; Mef2c[gene ID: "ENSMUSG00000005583" species: "Mus musculus" TF status: "inferred" TF family: "MADS box" DBDs: "SRF-TF"]; Mef2d[gene ID: "ENSMUSG00000001419" species: "Mus musculus" TF status: "inferred" TF family: "MADS box" DBDs: "SRF-TF"]<br>Possible TFs: MEF2A	*3.41741*	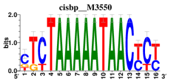	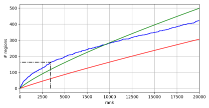	link	link	PWMs
354	☐ scertf__zhu.NHP6A<br>Description: zhu.NHP6A<br>Possible TFs: HMGB3, HMGB2, HMGB1, HMGB4	*3.41677*	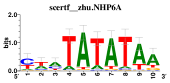	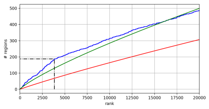	link	link	PWMs
355	☐ hdpi__HSPA1L<br>Description: HSPA1L<br>Possible TFs: HSPA1L	*3.41654*	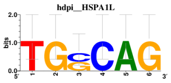	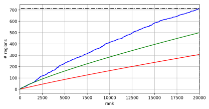	link	link	PWMs
356	☐ ENCFF001WPC<br>Description: DNase-seq on human LHCN-M2 differentiated for 4 days	*3.41496*		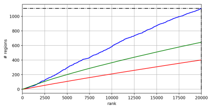	link	link	DHS & FAIRE
357	☐ E129-H3K4me1<br>Description: H3K4me1 in Osteoblast Primary Cells (E129,)	*3.41448*		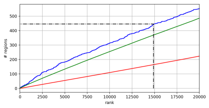	link	link	Histone modifications
358	☐ hocomoco__ANDR_MOUSE.H11MO.0.A<br>Description: ANDR_MOUSE<br>Possible TFs: AR	*3.40906*	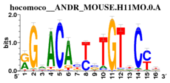	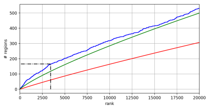	link	link	PWMs
359	☐ transfac_pro__M02876<br>Description: V$JUNDM2_04: JUNDM2 secondary motif<br>Possible TFs: JDP2	*3.40732*	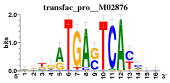	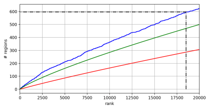	link	link	PWMs
360	☐ ENCFF001WPF<br>Description: UW human LHCN-M2 DNase-seq	*3.39851*		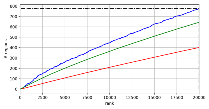	link	link	DHS & FAIRE
361	☐ transfac_pro__M02104<br>Description: V$NFE2_Q6: NF-E2<br>Possible TFs: MAFK, NFE2	*3.39759*	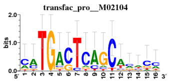	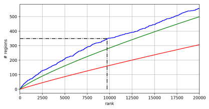	link	link	PWMs
362	☐ transfac_pro__M07363<br>Description: V$NF1FAMILY_Q4: NF-1<br>Possible TFs: NFIC, NFIB, NFIA	*3.38580*	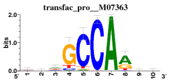	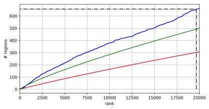	link	link	PWMs

#	Feature	NES	Logo	Recovery Curve	Candidate targets	All regions in top 20000	Database
363	☐ dbcorrdb__EP300__ENCSR000DZD_1__m3<br>Description: EP300 (ENCSR000DZD-1, motif 3)<br>Possible TFs: EP300	*3.37972*	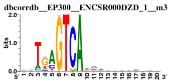	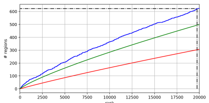	link	link	PWMs
364	☐ hdpi__RAB7A<br>Description: RAB7A<br>Possible TFs: RAB7A	*3.37529*	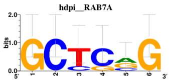	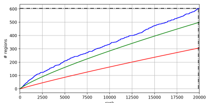	link	link	PWMs
365	☐ transfac_public__M00495<br>Description: V$BACH1_01: Bach1<br>Possible TFs: BACH1	*3.36548*	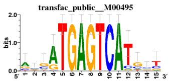	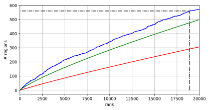	link	link	PWMs
366	☐ E121-H3K4me1<br>Description: H3K4me1 in HSMM cell derived Skeletal Muscle Myotubes Cells (E121,)	*3.35756*		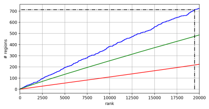	link	link	Histone modifications
367	☐ E104-H3K4me1<br>Description: H3K4me1 in Right Atrium (E104,)	*3.35020*		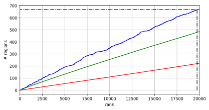	link	link	Histone modifications
368	☐ ENCFF001UPQ<br>Description: NFIC ChIP-seq protocol v042211.1 on human SK-N-SH	*3.34500*		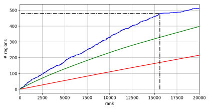	link	link	TF binding sites
369	☐ transfac_pro__M01795<br>Description: F$CAP1CA_01: Cap1	*3.34054*	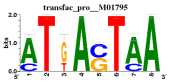	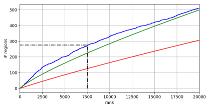	link	link	PWMs
370	☐ cisbp__M4440<br>Description: NR3C1[gene ID: "ENSG00000113580" species: "Homo sapiens" TF status: "direct" TF family: "Nuclear receptor" DBDs: "zf-C4"]; Nr3c2[gene ID: "ENSMUSG00000031618" species: "Mus musculus" TF status: "inferred" TF family: "Nuclear receptor" DBDs: "zf-C4"]; Pgr[gene ID: "ENSMUSG00000031870" species: "Mus musculus" TF status: "inferred" TF family: "Nuclear receptor" DBDs: "zf-C4"]<br>Possible TFs: NR3C1	*3.33600*	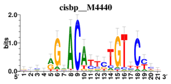	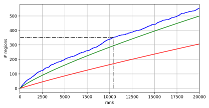	link	link	PWMs
371	☐ homer__AGAACAGNCTGTTCTT_ARE<br>Description: ARE(NR)/LNCAP-AR-ChIP-Seq(GSE27824)/Homer<br>Possible TFs: AR	*3.33521*	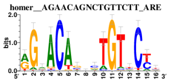	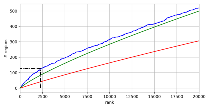	link	link	PWMs
372	☐ hocomoco__ZFX_HUMAN.H11MO.1.A<br>Description: ZFX_HUMAN<br>Possible TFs: ZFX	*3.33375*	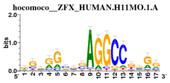	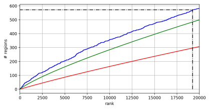	link	link	PWMs
373	☐ neph__UW.Motif.0012<br>Description: cagcctgg	*3.32971*	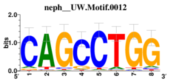	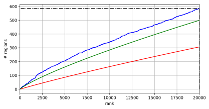	link	link	PWMs
374	☐ swissregulon__hs__TBP.p2<br>Description: hs__TBP.p2<br>Possible TFs: TBP	*3.32336*	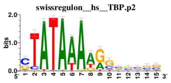	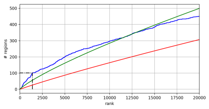	link	link	PWMs
375	☐ homer__VAGRACAKWCTGTYC_GRE<br>Description: GRE(NR),IR3/RAW264.7-GRE-ChIP-Seq(Unpublished)/Homer	*3.32221*	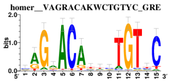	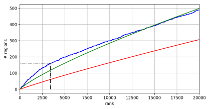	link	link	PWMs

#	Feature	NES	Logo	Recovery Curve	Candidate targets	All regions in top 20000	Database
376	☐ E100-H3K4me1<br>Description: H3K4me1 in Psoas Muscle (E100,)	*3.32163*		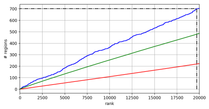	link	link	Histone modifications
377	☐ elemento__CCTGCCC<br>Description: Conserved regulatory element CCTGCCC between Hs and Mm	*3.32126*	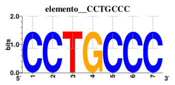	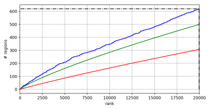	link	link	PWMs
378	☐ cisbp__M0476<br>Description: M0476	*3.31409*	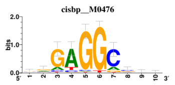	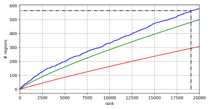	link	link	PWMs
379	☐ dbcorrdb__NR3C1__ENCSR000BHE_1__m1<br>Description: NR3C1 (ENCSR000BHE-1, motif 1)<br>Possible TFs: NR3C1	*3.31372*	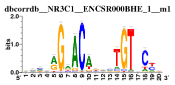	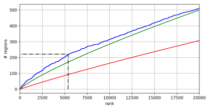	link	link	PWMs
380	☐ ENCFF001WFF<br>Description: UW human GM04504 DNase-seq	*3.31069*		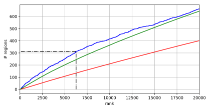	link	link	DHS & FAIRE
381	☐ dbcorrdb__EP300__ENCSR000ECV_1__m1<br>Description: EP300 (ENCSR000ECV-1, motif 1)<br>Possible TFs: EP300	*3.30678*	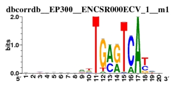	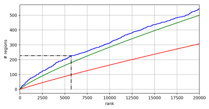	link	link	PWMs
382	☐ ENCFF001TSV<br>Description: JUND ChIP-seq (protocol v041610.2) on A549 treated with ethanol at 0.02%	*3.30322*		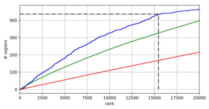	link	link	TF binding sites
383	☐ ENCFF001WCG<br>Description: DNase-seq on human SKMC	*3.30231*		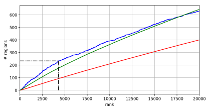	link	link	DHS & FAIRE
384	☐ dbcorrdb__NR3C1__ENCSR000BJC_1__m1<br>Description: NR3C1 (ENCSR000BJC-1, motif 1)<br>Possible TFs: NR3C1	*3.30041*	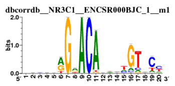	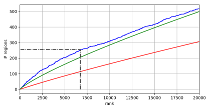	link	link	PWMs
385	☐ transfac_pro__M00319<br>Description: V$MEF3_B: MEF-3	*3.29663*	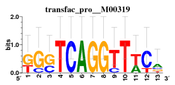	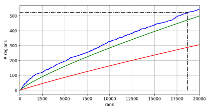	link	link	PWMs
386	☐ hdpi__AFF4<br>Description: AFF4<br>Possible TFs: AFF4	*3.29566*	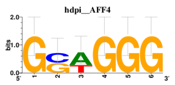	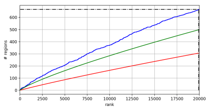	link	link	PWMs
387	☐ ENCFF001UMS<br>Description: FOSL2 ChIP-seq protocol v042211.1 on human MCF-7	*3.29381*		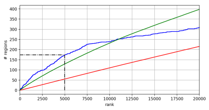	link	link	TF binding sites
388	☐ E089-H3K4me1-broadpeak<br>Description: H3K4me1 in Fetal Muscle Trunk (E089, broadpeak)	*3.28892*		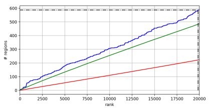	link	link	Histone modifications
389	☐ dbcorrdb__STAT3__ENCSR000DOX_1__m2<br>Description: STAT3 (ENCSR000DOX-1, motif 2)<br>Possible TFs: STAT3	*3.28177*	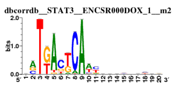	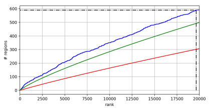	link	link	PWMs

#	Feature	NES	Logo	Recovery Curve	Candidate targets	All regions in top 20000	Database
390	☐ ENCFF001TSE<br>Description: FOSL2 ChIP-seq (protocol v042211.1) on A549 treated with ethanol at 0.02%	*3.27835*		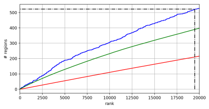	link	link	TF binding sites
391	☐ transfac_pro__M02025<br>Description: V$MEF2C_Q4: MEF-2C<br>Possible TFs: MEF2C	*3.27745*	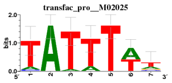	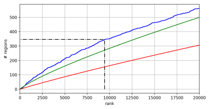	link	link	PWMs
392	☐ transfac_pro__M04924<br>Description: V$EBF1_05: COE1<br>Possible TFs: EBF1	*3.27606*	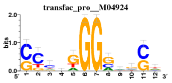	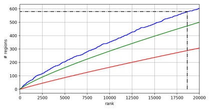	link	link	PWMs
393	☐ hocomoco__BACH1_HUMAN.H11MO.0.A<br>Description: BACH1_HUMAN<br>Possible TFs: BACH1	*3.27436*	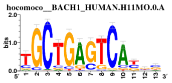	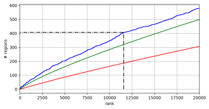	link	link	PWMs
394	☐ taipale_cyt_meth__MEF2C_CCWWATWWRG_FL_meth_repr<br>Description: MEF2C [MADS, CpG-meth]<br>Possible TFs: MEF2C	*3.27372*	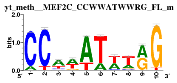	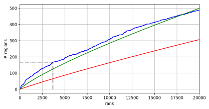	link	link	PWMs
395	☐ hdpi__SMPX<br>Description: SMPX<br>Possible TFs: SMPX	*3.27350*	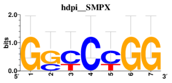	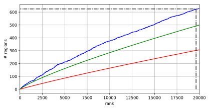	link	link	PWMs
396	☐ dbcorrdb__NR3C1__ENCSR000BHG_1__m1<br>Description: NR3C1 (ENCSR000BHG-1, motif 1)<br>Possible TFs: NR3C1	*3.27201*	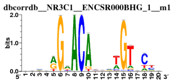	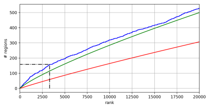	link	link	PWMs
397	☐ transfac_pro__M03845<br>Description: V$SMAD1_Q6: Smad1<br>Possible TFs: SMAD1	*3.26547*	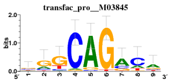	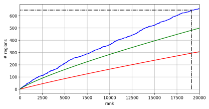	link	link	PWMs
398	☐ taipale_cyt_meth__JUNB_NATGASTCAYN_eDBD_meth<br>Description: JUNB [bZIP, CpG-meth]<br>Possible TFs: JUNB	*3.26332*	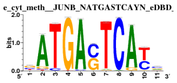	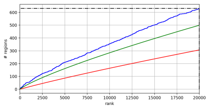	link	link	PWMs
399	☐ hocomoco__GCR_MOUSE.H11MO.0.A<br>Description: GCR_MOUSE	*3.25486*	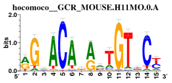	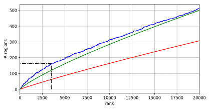	link	link	PWMs
400	☐ ENCFF001SSG<br>Description: DNase-seq on human SKMC	*3.25103*		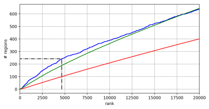	link	link	DHS & FAIRE
401	☐ ENCFF001WSN<br>Description: DNase-seq on human SKMC	*3.25103*		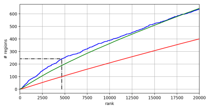	link	link	DHS & FAIRE
402	☐ E110-H3K4me1<br>Description: H3K4me1 in Stomach Mucosa (E110,)	*3.24521*		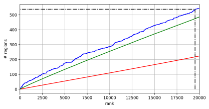	link	link	Histone modifications
403	☐ jaspar__MA0591.1<br>Description: Bach1::Mafk<br>Possible TFs: MAFK	*3.24494*	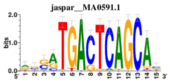	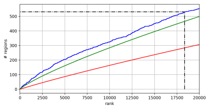	link	link	PWMs

#	Feature	NES	Logo	Recovery Curve	Candidate targets	All regions in top 20000	Database
404	☐ transfac_pro__M07610<br>Description: V$NRF2_Q3: Nrf2<br>Possible TFs: NFE2L2	*3.23474*	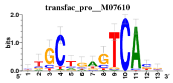	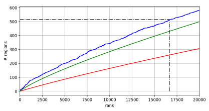	**link**	**link**	PWMs
405	☐ taipale_cyt_meth__JUN_NATGACKCATN_FL<br>Description: JUN [bZIP]<br>Possible TFs: JUN	*3.23066*	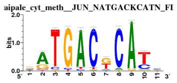	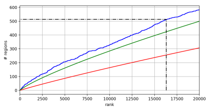	**link**	**link**	PWMs
406	☐ transfac_public__M00172<br>Description: V$AP1FJ_Q2: AP-1<br>Possible TFs: JUN, FOS	*3.22532*	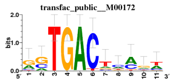	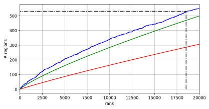	**link**	**link**	PWMs
407	☐ fantom__motif169_GCCTGGCC<br>Description: DENOVO_DMF_MOUSE: Motif229	*3.20884*	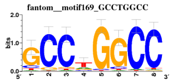	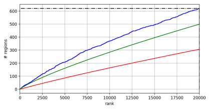	**link**	**link**	PWMs
408	☐ stark__STATAWAWRSVVV<br>Description: TATA<br>Possible TFs: TBP, TBPL2	*3.20102*	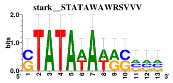	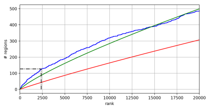	**link**	**link**	PWMs
409	☐ dbcorrdb__TRIM28__ENCSR000EYC_1__m1<br>Description: TRIM28 (ENCSR000EYC-1, motif 1)<br>Possible TFs: TRIM28	*3.19022*	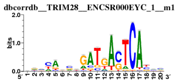	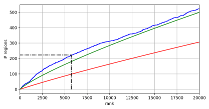	**link**	**link**	PWMs
410	☐ cisbp__M1836<br>Description: M1836	*3.19015*	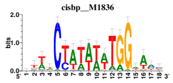	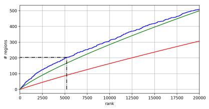	**link**	**link**	PWMs
411	☐ cisbp__M0296<br>Description: Fos[gene ID: "ENSMUSG00000021250" species: "Mus musculus" TF status: "inferred" TF family: "bZIP" DBDs: "bZIP_1"]; Fosb[gene ID: "ENSMUSG00000003545" species: "Mus musculus" TF status: "inferred" TF family: "bZIP" DBDs: "bZIP_1"]; Fosl1[gene ID: "ENSMUSG00000024912" species: "Mus musculus" TF status: "direct" TF family: "bZIP" DBDs: "bZIP_1"]<br>Possible TFs: FOSL1	*3.18898*	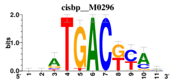	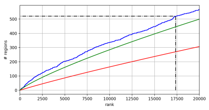	**link**	**link**	PWMs
412	☐ taipale_cyt_meth__JDP2_NRTGASTCAYN_FL<br>Description: JDP2 [bZIP]<br>Possible TFs: JDP2	*3.18507*	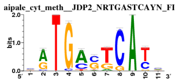	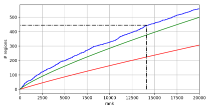	**link**	**link**	PWMs
413	☐ transfac_pro__M00720<br>Description: V$CACBINDINGPROTEIN_Q6: CAC-binding protein	*3.18394*	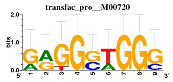	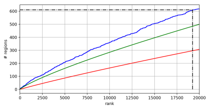	**link**	**link**	PWMs
414	☐ hdpi__TCEAL6<br>Description: TCEAL6<br>Possible TFs: TCEAL6	*3.18309*	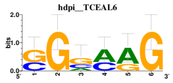	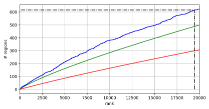	**link**	**link**	PWMs
415	☐ transfac_pro__M00304<br>Description: F$DDE1_B: Dde box	*3.17713*	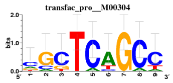	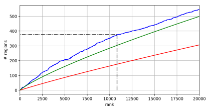	**link**	**link**	PWMs
416	☐ jaspar__MA0001.2<br>Description: AGL3	*3.17129*	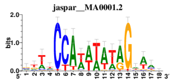	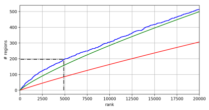	**link**	**link**	PWMs

#	Feature	NES	Logo	Recovery Curve	Candidate targets	All regions in top 20000	Database
417	☐ yetfasco__YMR037C_1380<br>Description: YMR037C_1380<br>Possible TFs: MECOM, PRDM16, PRDM13	*3.16711*	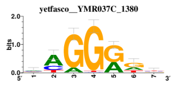	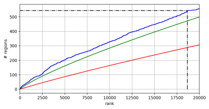	link	link	PWMs
418	☐ taipale_cyt_meth__JUN_NATGACTCATN_FL_meth<br>Description: JUN [bZIP, CpG-meth]<br>Possible TFs: JUN	*3.15946*	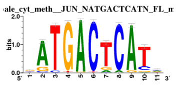	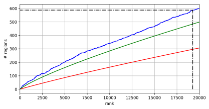	link	link	PWMs
419	☐ cisbp__M2619<br>Description: M2619	*3.15437*	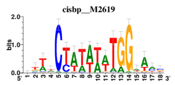	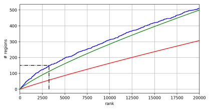	link	link	PWMs
420	☐ transfac_pro__M04693<br>Description: V$FOS_02: c-Fos<br>Possible TFs: FOS	*3.15061*	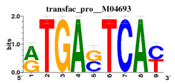	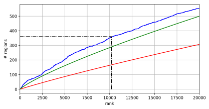	link	link	PWMs
421	☐ transfac_pro__M07270<br>Description: V$TEF3_Q3: TEF-3<br>Possible TFs: TEAD4	*3.14993*	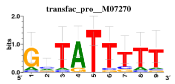	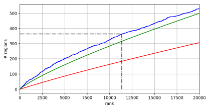	link	link	PWMs
422	☐ ENCFF001UQB<br>Description: RXRA ChIP-seq protocol v042211.1 on human SK-N-SH	*3.13651*		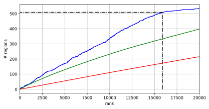	link	link	TF binding sites
423	☐ taipale_cyt_meth__NFE2_NATGASTCATN_eDBD<br>Description: NFE2 [bZIP]<br>Possible TFs: NFE2	*3.13480*	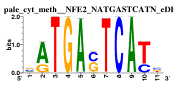	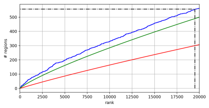	link	link	PWMs
424	☐ transfac_pro__M04811<br>Description: V$RELA_03: RelA-p65<br>Possible TFs: RELA	*3.12627*	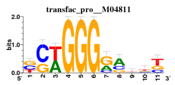	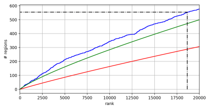	link	link	PWMs
425	☐ E125-H3K4me1-broadpeak<br>Description: H3K4me1 in NH-A Astrocytes Primary Cells (E125, broadpeak)	*3.11900*		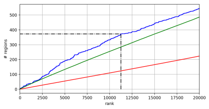	link	link	Histone modifications
426	☐ E128-H3K4me1-broadpeak<br>Description: H3K4me1 in NHLF Lung Fibroblast Primary Cells (E128, broadpeak)	*3.11519*		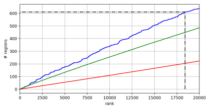	link	link	Histone modifications
427	☐ cisbp__M4684<br>Description: ATF3[gene ID: "ENSG00000162772" species: "Homo sapiens" TF status: "direct" TF family: "bZIP" DBDs: "bZIP_1"]<br>Possible TFs: ATF3	*3.11363*	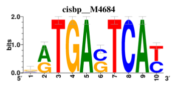	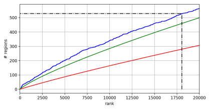	link	link	PWMs
428	☐ transfac_pro__M07266<br>Description: V$P300_Q5: p300<br>Possible TFs: APEX1, EP300	*3.10738*	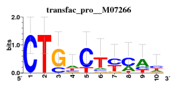	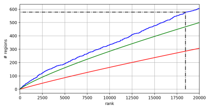	link	link	PWMs
429	☐ ENCFF001TVR<br>Description: TCF12 ChIP-seq protocol v042211.1 on human ECC-1. Note- This experiment previously referred to its biosample as ECC-1, however it has been found that all currently available ECC-1 are actually Ishikawa cells.	*3.10579*		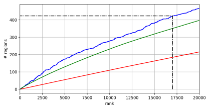	link	link	TF binding sites

#	Feature	NES	Logo	Recovery Curve	Candidate targets	All regions in top 20000	Database
430	☐ hocomoco__BACH2_HUMAN.H11MO.0.A<br>Description: BACH2_HUMAN<br>Possible TFs: BACH2	*3.10087*	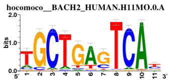	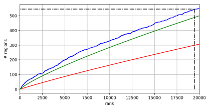	link	link	PWMs
431	☐ elemento__CCAGGCC<br>Description: Conserved regulatory element CCAGGCC between Hs and Mm	*3.09893*	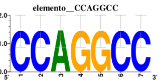	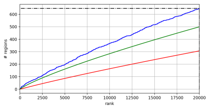	link	link	PWMs
432	☐ ENCFF001WSO<br>Description: DNase-seq on human SKMC	*3.09773*		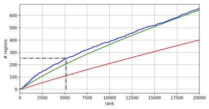	link	link	DHS & FAIRE
433	☐ transfac_pro__M02050<br>Description: V$CTF_01: CTF/NF1	*3.09178*	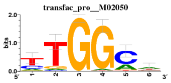	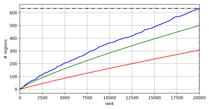	link	link	PWMs
434	☐ E055-H3K4me1<br>Description: H3K4me1 in Foreskin Fibroblast Primary Cells skin01 (E055,)	*3.08927*		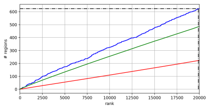	link	link	Histone modifications
435	☐ hocomoco__THA_MOUSE.H11MO.1.C<br>Description: THA_MOUSE<br>Possible TFs: THRA	*3.08861*	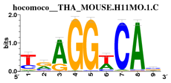	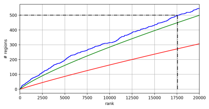	link	link	PWMs
436	☐ hocomoco__ANDR_HUMAN.H11MO.1.A<br>Description: ANDR_HUMAN<br>Possible TFs: AR	*3.07989*	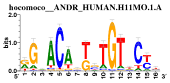	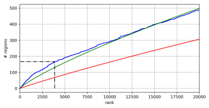	link	link	PWMs
437	☐ hocomoco__NFIC_HUMAN.H11MO.1.A<br>Description: NFIC_HUMAN<br>Possible TFs: NFIC	*3.07943*	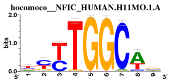	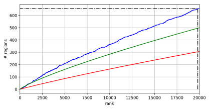	link	link	PWMs
438	☐ cisbp__M0320<br>Description: Jund[gene ID: "ENSMUSG00000071076" species: "Mus musculus" TF status: "direct" TF family: "bZIP" DBDs: "bZIP_1"]<br>Possible TFs: JUND	*3.07432*	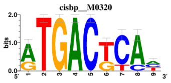	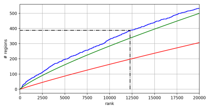	link	link	PWMs
439	☐ elemento__CCCAGCC<br>Description: Conserved regulatory element CCCAGCC between Hs and Mm	*3.07270*	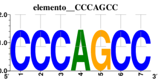	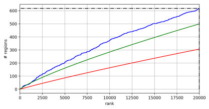	link	link	PWMs
440	☐ transfac_pro__M01822<br>Description: V$CPBP_Q6: CPBP<br>Possible TFs: KLF6	*3.07056*	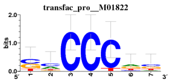	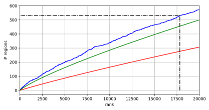	link	link	PWMs
441	☐ ENCFF001UQC<br>Description: RXRA ChIP-seq protocol v042211.1 on human SK-N-SH	*3.05974*		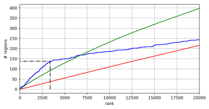	link	link	TF binding sites
442	☐ taipale_tf_pairs__BACH1_ATGACTCAT_HT<br>Description: BACH1 [bZIP]<br>Possible TFs: BACH1	*3.05076*	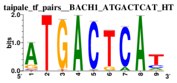	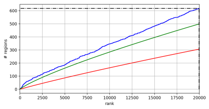	link	link	PWMs
443	☐ hocomoco__GCR_HUMAN.H11MO.0.A<br>Description: GCR_HUMAN<br>Possible TFs: NR3C1	*3.04461*	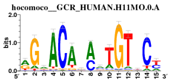	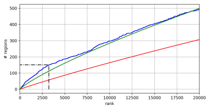	link	link	PWMs

#	Feature	NES	Logo	Recovery Curve	Candidate targets	All regions in top 20000	Database
444	☐ E126-H3K4me1<br>Description: H3K4me1 in NHDF-Ad Adult Dermal Fibroblast Primary Cells (E126,)	*3.04416*		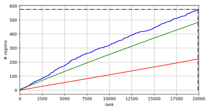	link	link	Histone modifications
445	☐ transfac_pro__M07374<br>Description: V$BACH1_Q3: Bach1<br>Possible TFs: BACH1	*3.04308*	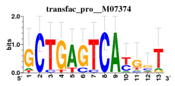	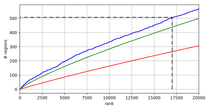	link	link	PWMs
446	☐ ENCFF001UJX<br>Description: FOSL1 ChIP-seq protocol v041610.1 on human K562	*3.03876*		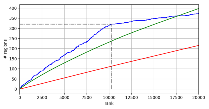	link	link	TF binding sites
447	☐<br>taipale_cyt_meth__NFE2_NATGASTCATN_eDBD_meth_repr<br>Description: NFE2 [bZIP, CpG-meth]<br>Possible TFs: NFE2	*3.03508*	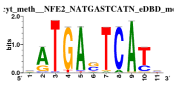	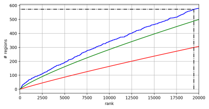	link	link	PWMs
448	☐ dbcorrdb__BACH1__ENCSR000EBQ_1__m1<br>Description: BACH1 (ENCSR000EBQ-1, motif 1)<br>Possible TFs: BACH1	*3.03232*	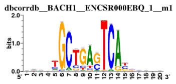	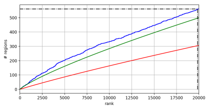	link	link	PWMs
449	☐ ENCFF001WAE<br>Description: DNase-seq on human HMF	*3.03194*		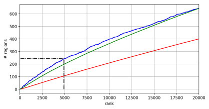	link	link	DHS & FAIRE
450	☐ dbcorrdb__TBL1XR1__ENCSR000EGB_1__m2<br>Description: TBL1XR1 (ENCSR000EGB-1, motif 2)<br>Possible TFs: TBL1XR1	*3.02082*	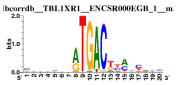	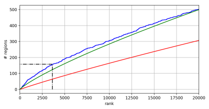	link	link	PWMs
451	☐ ENCFF001UPB<br>Description: FOSL2 ChIP-seq protocol v042211.1 on human SK-N-SH	*3.01563*		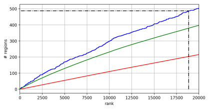	link	link	TF binding sites
452	☐ transfac_pro__M04854<br>Description: V$SMC3_03: SMC-3<br>Possible TFs: SMC3	*3.01416*	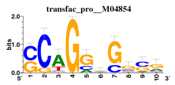	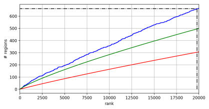	link	link	PWMs
453	☐ cisbp__M4428<br>Description: NR3C1[gene ID: "ENSG00000113580" species: "Homo sapiens" TF status: "direct" TF family: "Nuclear receptor" DBDs: "zf-C4"]; Nr3c2[gene ID: "ENSMUSG00000031618" species: "Mus musculus" TF status: "inferred" TF family: "Nuclear receptor" DBDs: "zf-C4"]; Pgr[gene ID: "ENSMUSG00000031870" species: "Mus musculus" TF status: "inferred" TF family: "Nuclear receptor" DBDs: "zf-C4"]<br>Possible TFs: NR3C1	*3.01289*	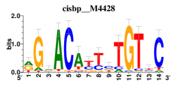	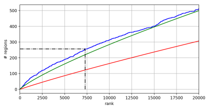	link	link	PWMs
454	☐ predrem__nrMotif408<br>Description: 157_fLung_R-DS17954.M636	*3.01196*	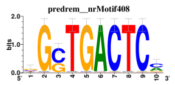	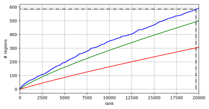	link	link	PWMs
455	☐ transfac_pro__M04750<br>Description: V$NR3C1_04: GR<br>Possible TFs: NR3C1	*3.01125*	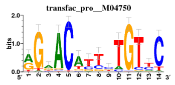	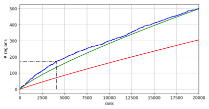	link	link	PWMs
456	☐ hdpi__UBB<br>Description: UBB<br>Possible TFs: UBB	*3.01008*	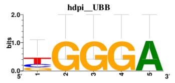	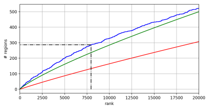	link	link	PWMs

#	Feature	NES	Logo	Recovery Curve	Candidate targets	All regions in top 20000	Database
457	☐ E117-H3K4me1<br>Description: H3K4me1 in HeLa-S3 Cervical Carcinoma Cell Line (E117,)	*3.00731*		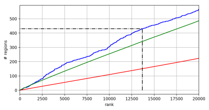	**link**	**link**	Histone modifications
458	☐ taipale_cyt_meth__JUN_NATGASTCATN_FL_meth<br>Description: JUN [bZIP, CpG-meth]<br>Possible TFs: JUN	*3.00712*	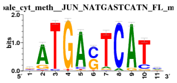	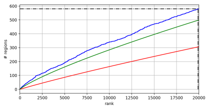	**link**	**link**	PWMs
459	☐ dbcorrdb__NFE2__ENCSR000FAF_1__m2<br>Description: NFE2 (ENCSR000FAF-1, motif 2)<br>Possible TFs: NFE2	*3.00332*	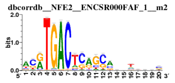	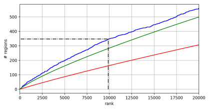	**link**	**link**	PWMs

© 2015-2019 **KULeuven** | Authors: Gert Hulselmans & Hana Imrichova | Web design (CSS) by **Andreas Viklund**.
