# Supplementary material for: Chromatin accessibility and regulatory vocabulary across indicine cattle tissues
Source: Genome Biol. 2021 Sep 21;22:273. doi: 10.1186/s13059-021-02489-7 (PMC8454054; doi:10.1186/s13059-021-02489-7)
Supplement: Supplementary file 14 — Additional file 14. Enriched regulatory features in hypothalamus-specific peaks according to i-cisTarget online tool [68]. [file 13059_2021_2489_MOESM14_ESM.pdf]

i-cisTarget

An integrative genomics method for the prediction of regulatory features and cis-regulatory modules.

Parameters and statistics for hypo

Number of features	29890
Number of enriched features (NES > 3.0)	208
Total number of ranked regions	1223024
Type of input query	bed
Number of **i-cisTarget regions in input set**	43761 (**Results of the region mapping**)
Minimum fraction of overlap	0.4
Normalized enrichment score (NES) threshold	3.0
AUC threshold (fraction / # of ranked regions)	0.005 (6115)
Recovery curve threshold (# of regions)	20000

AUC distribution

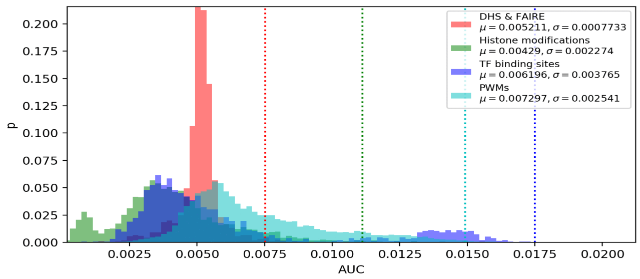

Recovery of best feature

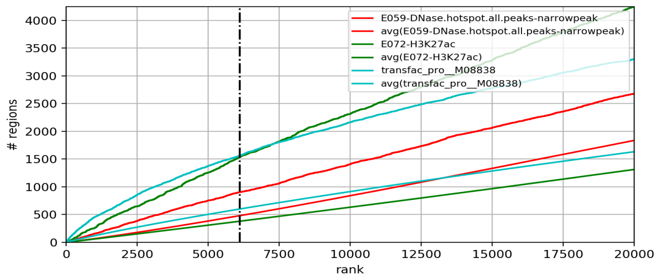

Results for hypo

Select features in the table below, select an operation and proceed.

1. ○ Use candidate target regions as **filter** and use as input for i-cisTarget again.
2. ○ **Scan** candidate target regions of selected features for multiple homotypic CRMs.
3. ○ **Create SIF file** for the selected features.

This report is also available as an **archive**.

For some databases, randomly generated features were enriched. Also, non-random features in these same databases that have a lower enrichment than the first randomly generated enriched feature were removed from the report. The involved databases and their adjusted NES thresholds are: TF binding sites (3.00000).

#	Feature	NES	Logo	Recovery Curve	Candidate targets	All regions in top 20000	Database
1	☐ E059-DNase.hotspot.all.peaks-narrowpeak<br>Description: DNase in Foreskin Melanocyte Primary Cells skin01 (E059, narrowpeak, hotspot)	*6.98587*		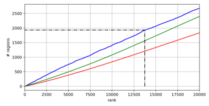	link	link	DHS & FAIRE
2	☐ E072-H3K27ac<br>Description: H3K27ac in Brain Inferior Temporal Lobe (E072,)	*5.97118*		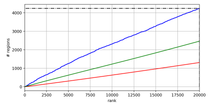	link	link	Histone modifications
3	☐ ENCFF001SPB<br>Description: DNase-seq on human iPS	*5.95974*		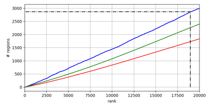	link	link	DHS & FAIRE
4	☐ E074-H3K27ac<br>Description: H3K27ac in Brain Substantia Nigra (E074,)	*5.80927*		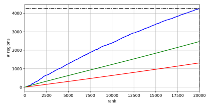	link	link	Histone modifications

#	Feature	NES	Logo	Recovery Curve	Candidate targets	All regions in top 20000	Database
5	E074-H3K4me1<br>Description: H3K4me1 in Brain Substantia Nigra (E074,)	*5.62262*		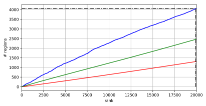	**link**	**link**	Histone modifications
6	transfac_pro__M08838<br>Description: V$SOX9_09: Sox-9<br>Possible TFs: SOX9	*5.48489*	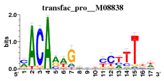	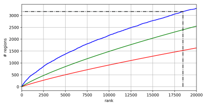	**link**	**link**	PWMs
7	ENCFF001SOF<br>Description: DNase-seq on human 8988T	*5.47943*		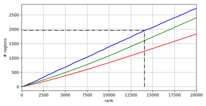	**link**	**link**	DHS & FAIRE
8	hocomoco__SOX9_HUMAN.H11MO.0.B<br>Description: SOX9_HUMAN<br>Possible TFs: SOX9	*5.44825*	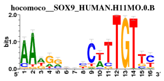	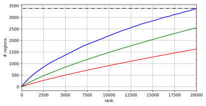	**link**	**link**	PWMs
9	E071-H3K4me1<br>Description: H3K4me1 in Brain Hippocampus Middle (E071,)	*5.41850*		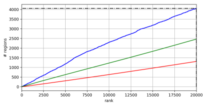	**link**	**link**	Histone modifications
10	E072-H3K4me1<br>Description: H3K4me1 in Brain Inferior Temporal Lobe (E072,)	*5.32328*		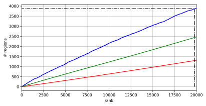	**link**	**link**	Histone modifications
11	hocomoco__SOX10_HUMAN.H11MO.0.B<br>Description: SOX10_HUMAN<br>Possible TFs: SOX10	*5.11977*	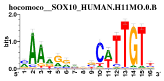	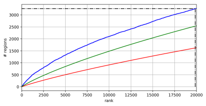	**link**	**link**	PWMs
12	taipale_cyt_meth__SOX8_ACAATNNNNNNATTGT_FL<br>Description: SOX8 [HMG]<br>Possible TFs: SOX8	*5.05694*	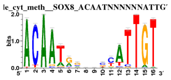	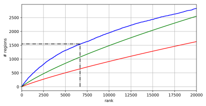	**link**	**link**	PWMs
13	E069-H3K4me1<br>Description: H3K4me1 in Brain Cingulate Gyrus (E069,)	*5.02405*		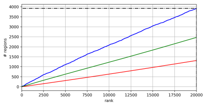	**link**	**link**	Histone modifications
14	E069-H3K27ac<br>Description: H3K27ac in Brain Cingulate Gyrus (E069,)	*4.96353*		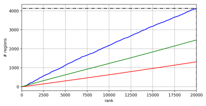	**link**	**link**	Histone modifications
15	taipale_cyt_meth__SOX10_AACAATNNNNNNATTGTT_FL_meth<br>Description: SOX10 [HMG, CpG-meth]<br>Possible TFs: SOX10	*4.74836*	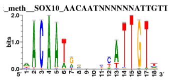	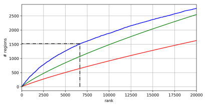	**link**	**link**	PWMs
16	taipale_cyt_meth__SOX10_AACAATNNNNNNATTGTT_FL_repr<br>Description: SOX10 [HMG]<br>Possible TFs: SOX10	*4.74452*	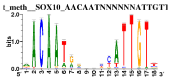	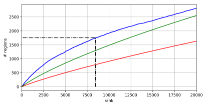	**link**	**link**	PWMs
17	E068-H3K4me1<br>Description: H3K4me1 in Brain Anterior Caudate (E068,)	*4.68035*		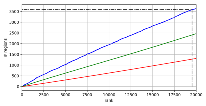	**link**	**link**	Histone modifications
18	E071-H3K4me1-broadpeak<br>Description: H3K4me1 in Brain Hippocampus Middle (E071, broadpeak)	*4.67569*		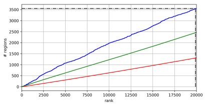	**link**	**link**	Histone modifications

#	Feature	NES	Logo	Recovery Curve	Candidate targets	All regions in top 20000	Database
19	☐ E069-H3K4me1-broadpeak<br>Description: H3K4me1 in Brain Cingulate Gyrus (E069, broadpeak)	*4.58990*		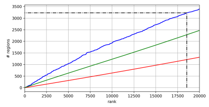	**link**	**link**	Histone modifications
20	☐ taipale_cyt_meth__SOX8_ACAATNNNNNNATTGT_FL_meth<br>Description: SOX8 [HMG, CpG-meth]<br>Possible TFs: SOX8	*4.58464*	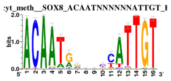	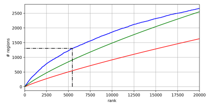	**link**	**link**	PWMs
21	☐ ENCFF001SPG<br>Description: Duke human Medullo DNase-seq	*4.58199*		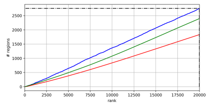	**link**	**link**	DHS & FAIRE
22	☐ E074-H3K4me1-broadpeak<br>Description: H3K4me1 in Brain Substantia Nigra (E074, broadpeak)	*4.53705*		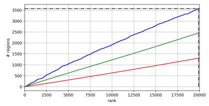	**link**	**link**	Histone modifications
23	☐ ENCFF001SOV<br>Description: DNase-seq on human Hepatocytes	*4.45191*		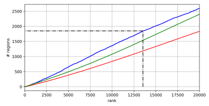	**link**	**link**	DHS & FAIRE
24	☐ E067-H3K27ac<br>Description: H3K27ac in Brain Angular Gyrus (E067,)	*4.43969*		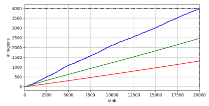	**link**	**link**	Histone modifications
25	☐ E067-H3K4me1<br>Description: H3K4me1 in Brain Angular Gyrus (E067,)	*4.35469*		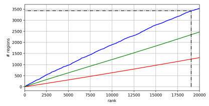	**link**	**link**	Histone modifications
26	☐ E053-H3K4me1<br>Description: H3K4me1 in Cortex derived primary cultured neurospheres (E053,)	*4.32833*		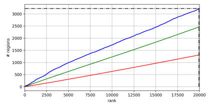	**link**	**link**	Histone modifications
27	☐ cisbp__M0542<br>Description: YPR015C[gene ID: "YPR015C" species: "Saccharomyces cerevisiae" TF status: "direct" TF family: "C2H2 ZF" DBDs: "zf-C2H2"]	*4.30710*	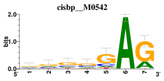	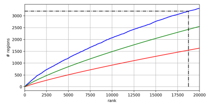	**link**	**link**	PWMs
28	☐ ENCFF001UWA<br>Description: Duke human HSMMtube DNase-seq	*4.06473*		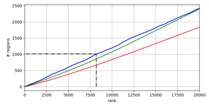	**link**	**link**	DHS & FAIRE
29	☐ E097-H3K4me3<br>Description: H3K4me3 in Ovary (E097,)	*3.97709*		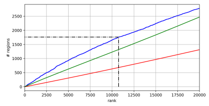	**link**	**link**	Histone modifications
30	☐ ENCFF001UVM<br>Description: Duke human H1-hESC DNase-seq	*3.96917*		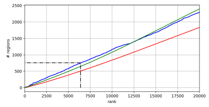	**link**	**link**	DHS & FAIRE
31	☐ ENCFF001UYJ<br>Description: FAIRE-seq on H1-hESC	*3.95718*		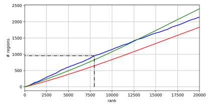	**link**	**link**	DHS & FAIRE
32	☐ E068-H3K4me1-broadpeak<br>Description: H3K4me1 in Brain Anterior Caudate (E068, broadpeak)	*3.92946*		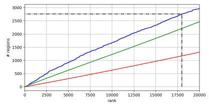	**link**	**link**	Histone modifications

#	Feature	NES	Logo	Recovery Curve	Candidate targets	All regions in top 20000	Database
33	☐ E008-H3K23me2<br>Description: H3K23me2 in H9 Cells (E008,)	*3.90623*		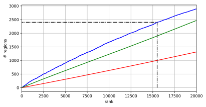	link	link	Histone modifications
34	☐ E073-H3K4me1<br>Description: H3K4me1 in Brain_Dorsolateral_Prefrontal_Cortex (E073,)	*3.90233*		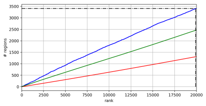	link	link	Histone modifications
35	☐ hdpi__ZCCHC14<br>Description: ZCCHC14<br>Possible TFs: ZCCHC14	*3.86228*	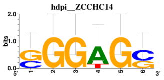	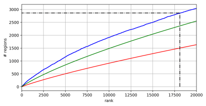	link	link	PWMs
36	☐ ENCFF001SOZ<br>Description: DNase-seq on human Huh-7.5	*3.82218*		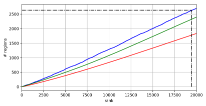	link	link	DHS & FAIRE
37	☐ ENCFF001SOT<br>Description: Duke human H9ES DNase-seq	*3.80714*		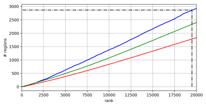	link	link	DHS & FAIRE
38	☐ E054-H3K4me1<br>Description: H3K4me1 in Ganglion Eminence derived primary cultured neurospheres (E054,)	*3.77807*		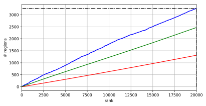	link	link	Histone modifications
39	☐ homer__CCWTTGTYYB_Sox10<br>Description: Sox10(HMG)/SciaticNerve-Sox3-ChIP-Seq(GSE35132)/Homer	*3.77597*	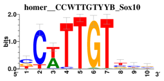	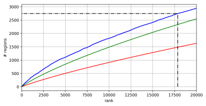	link	link	PWMs
40	☐ E029-DNase.hotspot.all.peaks-narrowpeak<br>Description: DNase in Primary monocytes from peripheral blood (E029, narrowpeak, hotspot)	*3.69976*		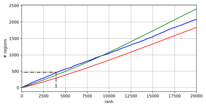	link	link	DHS & FAIRE
41	☐ E072-H3K27ac-broadpeak<br>Description: H3K27ac in Brain Inferior Temporal Lobe (E072, broadpeak)	*3.68776*		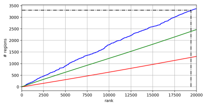	link	link	Histone modifications
42	☐ E071-H3K27ac<br>Description: H3K27ac in Brain Hippocampus Middle (E071,)	*3.64691*		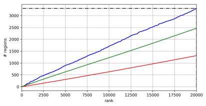	link	link	Histone modifications
43	☐ hdpi__PDLIM5<br>Description: PDLIM5<br>Possible TFs: PDLIM5	*3.64358*	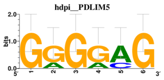	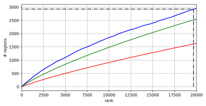	link	link	PWMs
44	☐ E061-H3K4me1<br>Description: H3K4me1 in Foreskin Melanocyte Primary Cells skin03 (E061,)	*3.63736*		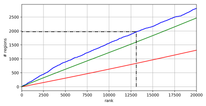	link	link	Histone modifications
45	☐ hocomoco__SOX3_MOUSE.H11MO.0.C<br>Description: SOX3_MOUSE	*3.61796*	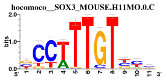	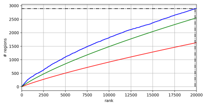	link	link	PWMs
46	☐ E059-DNase.macs2-narrowpeak<br>Description: DNase in Foreskin Melanocyte Primary Cells skin01 (E059, narrowpeak, macs2)	*3.59731*		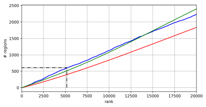	link	link	DHS & FAIRE

#	Feature	NES	Logo	Recovery Curve	Candidate targets	All regions in top 20000	Database
47	☐ E074-H3K27ac-broadpeak<br>Description: H3K27ac in Brain Substantia Nigra (E074, broadpeak)	*3.59303*		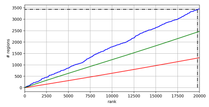	**link**	**link**	Histone modifications
48	☐ homer__CCATTGTTNY_Sox6<br>Description: Sox6(HMG)/Myotubes-Sox6-ChIP-Seq(GSE32627)/Homer<br>Possible TFs: SOX6	*3.58969*	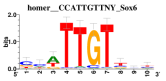	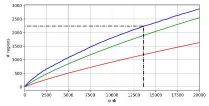	**link**	**link**	PWMs
49	☐ hdpi__PGAM2<br>Description: PGAM2<br>Possible TFs: PGAM2	*3.58587*	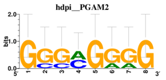	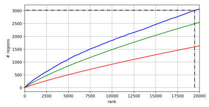	**link**	**link**	PWMs
50	☐ tfdimers__MD00293<br>Description: M01308_forward_9_M01590_reverse dimer: SOX4 / SMAD1<br>Possible TFs: SMAD1, SOX4	*3.57233*	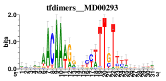	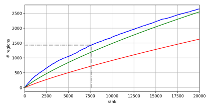	**link**	**link**	PWMs
51	☐ hdpi__GPD1<br>Description: GPD1<br>Possible TFs: GPD1	*3.53885*	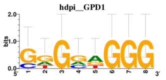	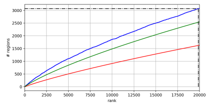	**link**	**link**	PWMs
52	☐ dbcorrdb__BRF1__ENCSR000DOJ_1__m2<br>Description: BRF1 (ENCSR000DOJ-1, motif 2)<br>Possible TFs: BRF1	*3.52581*	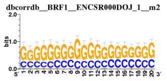	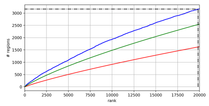	**link**	**link**	PWMs
53	☐ hocomoco__SOX3_HUMAN.H11MO.0.B<br>Description: SOX3_HUMAN<br>Possible TFs: SOX3	*3.52421*	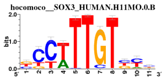	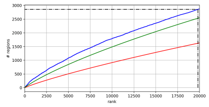	**link**	**link**	PWMs
54	☐ dbcorrdb__SREBF1__ENCSR000EZP_1__m1<br>Description: SREBF1 (ENCSR000EZP-1, motif 1)<br>Possible TFs: SREBF1	*3.52258*	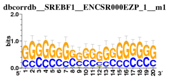	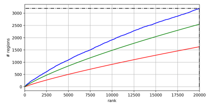	**link**	**link**	PWMs
55	☐ dbcorrdb__MAZ__ENCSR000EFF_1__m1<br>Description: MAZ (ENCSR000EFF-1, motif 1)<br>Possible TFs: MAZ	*3.51603*	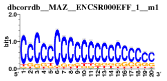	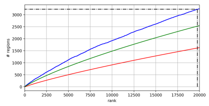	**link**	**link**	PWMs
56	☐ ENCFF001UWG<br>Description: DNase-seq on human iPS	*3.51318*		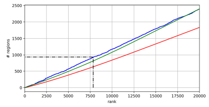	**link**	**link**	DHS & FAIRE
57	☐ hdpi__ASCC1<br>Description: ASCC1<br>Possible TFs: ASCC1	*3.49950*	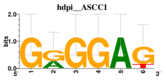	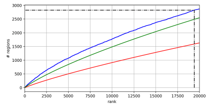	**link**	**link**	PWMs
58	☐ dbcorrdb__SREBF2__ENCSR000DYT_1__m7<br>Description: SREBF2 (ENCSR000DYT-1, motif 7)<br>Possible TFs: SREBF2	*3.48505*	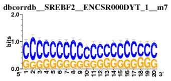	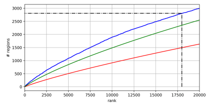	**link**	**link**	PWMs
59	☐ E067-H3K4me1-broadpeak<br>Description: H3K4me1 in Brain Angular Gyrus (E067, broadpeak)	*3.47953*		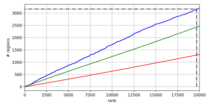	**link**	**link**	Histone modifications
60	☐ dbcorrdb__NELFE__ENCSR000DOF_1__m6<br>Description: NELFE (ENCSR000DOF-1, motif 6)<br>Possible TFs: NELFE	*3.47604*	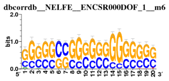	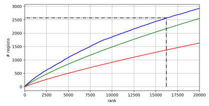	**link**	**link**	PWMs

#	Feature	NES	Logo	Recovery Curve	Candidate targets	All regions in top 20000	Database
61	☐ elemento__CGCGCTC<br>Description: Conserved regulatory element CGCGCTC between Hs and Mm	*3.47475*	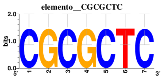	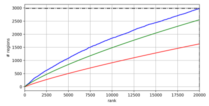	**link**	**link**	PWMs
62	☐ E059-H3K4me1<br>Description: H3K4me1 in Foreskin Melanocyte Primary Cells skin01 (E059,)	*3.45494*		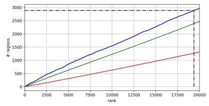	**link**	**link**	Histone modifications
63	☐ cisbp__M6483<br>Description: SP4[gene ID: "ENSG00000105866" species: "Homo sapiens" TF status: "direct" TF family: "C2H2 ZF" DBDs: "zf-C2H2"]; SP5[gene ID: "ENSG00000204335" species: "Homo sapiens" TF status: "inferred" TF family: "C2H2 ZF" DBDs: "zf-C2H2"]; SP6[gene ID: "ENSG00000189120" species: "Homo sapiens" TF status: "inferred" TF family: "C2H2 ZF" DBDs: "zf-C2H2"]; SP7[gene ID: "ENSG00000170374" species: "Homo sapiens" TF status: "inferred" TF family: "C2H2 ZF" DBDs: "zf-C2H2"]; SP9[gene ID: "ENSG00000217236" species: "Homo sapiens" TF status: "inferred" TF family: "C2H2 ZF" DBDs: "zf-C2H2"]; Sp2[gene ID: "ENSMUSG00000018678" species: "Mus musculus" TF status: "inferred" TF family: "C2H2 ZF" DBDs: "zf-C2H2"]; Sp3[gene ID: "ENSMUSG00000027109" species: "Mus musculus" TF status: "inferred" TF family: "C2H2 ZF" DBDs: "zf-C2H2"]; Sp5[gene ID: "ENSMUSG00000075304" species: "Mus musculus" TF status: "inferred" TF family: "C2H2 ZF" DBDs: "zf-C2H2"]; Sp6[gene ID: "ENSMUSG00000038560" species: "Mus musculus" TF status: "inferred" TF family: "C2H2 ZF" DBDs: "zf-C2H2"]; Sp7[gene ID: "ENSMUSG00000060284" species: "Mus musculus" TF status: "inferred" TF family: "C2H2 ZF" DBDs: "zf-C2H2"]; Sp8[gene ID: "ENSMUSG00000048562" species: "Mus musculus" TF status: "inferred" TF family: "C2H2 ZF" DBDs: "zf-C2H2"]; Sp9[gene ID: "ENSMUSG00000068859" species: "Mus musculus" TF status: "inferred" TF family: "C2H2 ZF" DBDs: "zf-C2H2"]<br>Possible TFs: SP4	*3.45197*	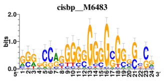	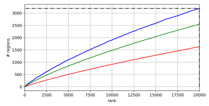	**link**	**link**	PWMs
64	☐ hocomoco__SOX10_MOUSE.H11MO.1.A<br>Description: SOX10_MOUSE<br>Possible TFs: SOX10	*3.45166*	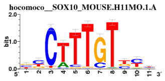	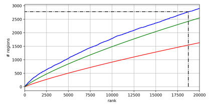	**link**	**link**	PWMs
65	☐ predrem__nrMotif2358<br>Description: 38_fBrain-DS20231.M303	*3.45092*	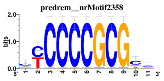	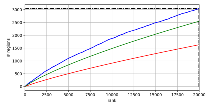	**link**	**link**	PWMs
66	☐ dbcorrdb__HDAC2__ENCSR000AQG_1__m4<br>Description: HDAC2 (ENCSR000AQG-1, motif 4)<br>Possible TFs: HDAC2	*3.44981*	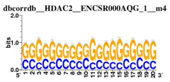	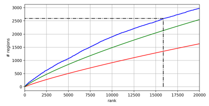	**link**	**link**	PWMs
67	☐ hocomoco__ZBT14_HUMAN.H11MO.0.C<br>Description: ZBT14_HUMAN<br>Possible TFs: ZBTB14	*3.44528*	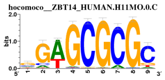	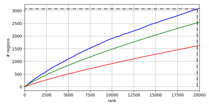	**link**	**link**	PWMs
68	☐ dbcorrdb__ZMIZ1__ENCSR000EFQ_1__m2<br>Description: ZMIZ1 (ENCSR000EFQ-1, motif 2)<br>Possible TFs: ZMIZ1	*3.44439*	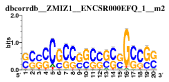	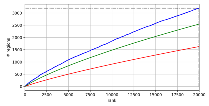	**link**	**link**	PWMs
69	☐ transfac_pro__M06208<br>Description: V$ZNF460_01: ZNF460<br>Possible TFs: ZNF460	*3.43869*	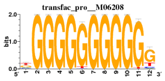	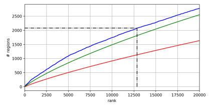	**link**	**link**	PWMs

#	Feature	NES	Logo	Recovery Curve	Candidate targets	All regions in top 20000	Database
70	☐ dbcorrdb__CTCF__ENCSR000BHW_1__m2<br>Description: CTCF (ENCSR000BHW-1, motif 2)<br>Possible TFs: CTCF	*3.43790*	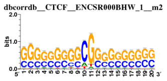	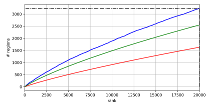	**link**	**link**	PWMs
71	☐ hdpi__VIL2<br>Description: VIL2<br>Possible TFs: EZR	*3.43152*	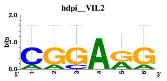	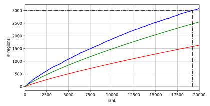	**link**	**link**	PWMs
72	☐ cisbp__M4536<br>Description: E2F1[gene ID: "ENSG00000101412" species: "Homo sapiens" TF status: "direct" TF family: "E2F" DBDs: "E2F_TDP"]; E2f1[gene ID: "ENSMUSG00000027490" species: "Mus musculus" TF status: "inferred" TF family: "E2F" DBDs: "E2F_TDP"]<br>Possible TFs: E2F1	*3.42649*	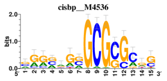	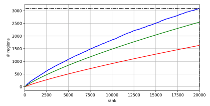	**link**	**link**	PWMs
73	☐ dbcorrdb__SMARCA4__ENCSR000EZC_1__m10<br>Description: SMARCA4 (ENCSR000EZC-1, motif 10)<br>Possible TFs: SMARCA4	*3.42110*	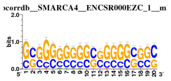	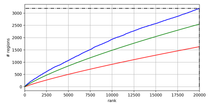	**link**	**link**	PWMs
74	☐ dbcorrdb__CEBPB__ENCSR000EBV_1__m2<br>Description: CEBPB (ENCSR000EBV-1, motif 2)<br>Possible TFs: CEBPB	*3.41963*	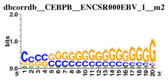	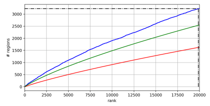	**link**	**link**	PWMs
75	☐ hdpi__AKR1A1<br>Description: AKR1A1<br>Possible TFs: AKR1A1	*3.41880*	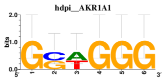	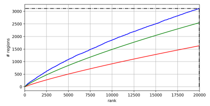	**link**	**link**	PWMs
76	☐ transfac_pro__M07344<br>Description: V$ZIC1_Q2: ZIC1<br>Possible TFs: ZIC1	*3.41770*	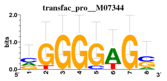	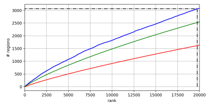	**link**	**link**	PWMs
77	☐ dbcorrdb__ESRRA__ENCSR000DYQ_1__m5<br>Description: ESRRA (ENCSR000DYQ-1, motif 5)<br>Possible TFs: ESRRA	*3.41656*	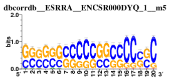	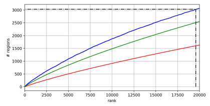	**link**	**link**	PWMs
78	☐ ENCFF001WEV<br>Description: DNase-seq on human CD4+ Naive (Wb78495824)	*3.41534*		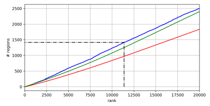	**link**	**link**	DHS & FAIRE
79	☐ hdpi__MCTP2<br>Description: MCTP2<br>Possible TFs: MCTP2	*3.41249*	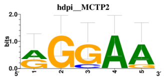	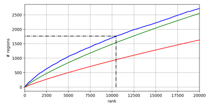	**link**	**link**	PWMs
80	☐ dbcorrdb__SUPT20H__ENCSR000ECQ_1__m5<br>Description: SUPT20H (ENCSR000ECQ-1, motif 5)<br>Possible TFs: SUPT20H	*3.41218*	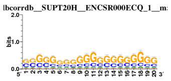	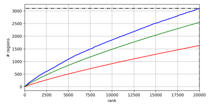	**link**	**link**	PWMs
81	☐ dbcorrdb__BRF1__ENCSR000DNW_1__m2<br>Description: BRF1 (ENCSR000DNW-1, motif 2)<br>Possible TFs: BRF1	*3.40613*	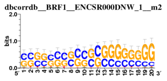	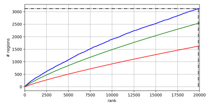	**link**	**link**	PWMs
82	☐ dbcorrdb__POLR2AphosphoS2__ENCSR000DYF_1__m10<br>Description: POLR2AphosphoS2 (ENCSR000DYF-1, motif 10)<br>Possible TFs: POLR2A	*3.40294*	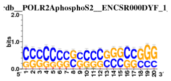	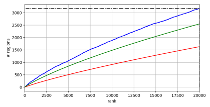	**link**	**link**	PWMs
83	☐ factorbook__E2F1<br>Description: E2F1<br>Possible TFs: E2F1	*3.40260*	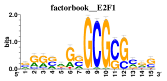	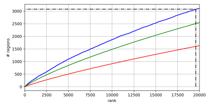	**link**	**link**	PWMs

#	Feature	NES	Logo	Recovery Curve	Candidate targets	All regions in top 20000	Database
84	☐ taipale_cyt_meth__SOX4_GAACAAAGRN_eDBD_repr<br>Description: SOX4 [HMG]<br>Possible TFs: SOX4	*3.40073*	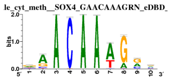	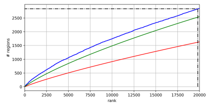	link	link	PWMs
85	☐ hdpi__ZDHHC5<br>Description: ZDHHC5<br>Possible TFs: ZDHHC5	*3.39930*	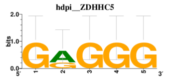	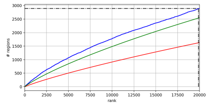	link	link	PWMs
86	☐ transfac_pro__M01721<br>Description: V$PUR1_Q4: PUR1<br>Possible TFs: PURA	*3.39888*	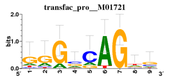	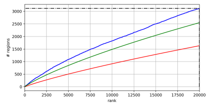	link	link	PWMs
87	☐ cisbp__M1604<br>Description: SOX11[gene ID: "ENSG00000176887" species: "Homo sapiens" TF status: "inferred" TF family: "Sox" DBDs: "HMG_box"]; Sox4[gene ID: "ENSMUSG00000076431" species: "Mus musculus" TF status: "direct" TF family: "Sox" DBDs: "HMG_box"]<br>Possible TFs: SOX4	*3.39773*	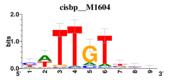	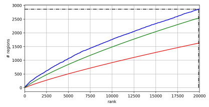	link	link	PWMs
88	☐ taipale_cyt_meth__SOX4_GAACAAAGRN_eDBD_meth<br>Description: SOX4 [HMG, CpG-meth]<br>Possible TFs: SOX4	*3.39551*	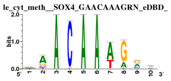	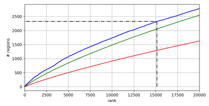	link	link	PWMs
89	☐ E007-H3K4me2<br>Description: H3K4me2 in H1 Derived Neuronal Progenitor Cultured Cells (E007,)	*3.37830*		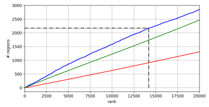	link	link	Histone modifications
90	☐ transfac_pro__M07269<br>Description: V$SOX9_Q5: Sox-9<br>Possible TFs: SOX9	*3.37401*	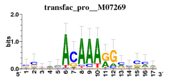	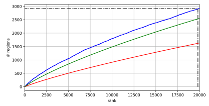	link	link	PWMs
91	☐ hdpi__HLCS<br>Description: HLCS<br>Possible TFs: HLCS	*3.35773*	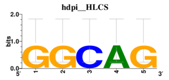	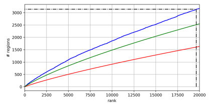	link	link	PWMs
92	☐ transfac_pro__M01835<br>Description: V$GKLF_Q4: GKLF<br>Possible TFs: KLF4	*3.35103*	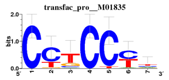	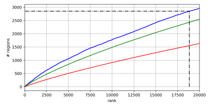	link	link	PWMs
93	☐ E072-H3K4me1-broadpeak<br>Description: H3K4me1 in Brain Inferior Temporal Lobe (E072, broadpeak)	*3.34443*		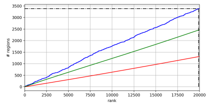	link	link	Histone modifications
94	☐ dbcorrdb__XRCC4__ENCSR000FAC_1__m1<br>Description: XRCC4 (ENCSR000FAC-1, motif 1)<br>Possible TFs: XRCC4	*3.34157*	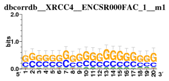	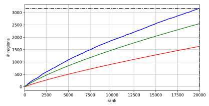	link	link	PWMs
95	☐ dbcorrdb__EZH2__ENCSR000AQE_1__m9<br>Description: EZH2 (ENCSR000AQE-1, motif 9)<br>Possible TFs: EZH2	*3.33557*	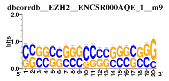	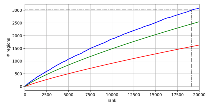	link	link	PWMs
96	☐ predrem__nrMotif2090<br>Description: 208_fPlacenta-DS17639.M33	*3.33173*	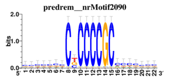	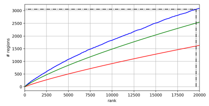	link	link	PWMs

#	Feature	NES	Logo	Recovery Curve	Candidate targets	All regions in top 20000	Database
97	☐ cisbp__M6539<br>Description: ZBTB7B[gene ID: "ENSG00000160685" species: "Homo sapiens" TF status: "direct" TF family: "C2H2 ZF" DBDs: "zf-C2H2"]<br>Possible TFs: ZBTB7B	*3.33095*	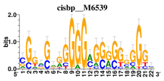	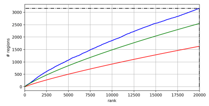	link	link	PWMs
98	☐ hdpi__ZMAT2<br>Description: ZMAT2<br>Possible TFs: ZMAT2	*3.32653*	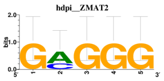	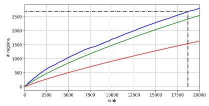	link	link	PWMs
99	☐ dbcorrdb__EZH2__ENCSR000ARD_1__m6<br>Description: EZH2 (ENCSR000ARD-1, motif 6)<br>Possible TFs: EZH2	*3.32345*	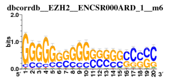	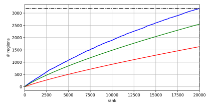	link	link	PWMs
100	☐ predrem__nrMotif1623<br>Description: 141_fLung_L-DS17154.M622	*3.31293*	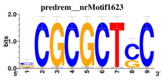	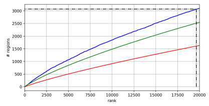	link	link	PWMs
101	☐ ENCFF001UUC<br>Description: DNase-seq on human 8988T	*3.30571*		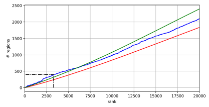	link	link	DHS & FAIRE
102	☐ transfac_pro__M08971<br>Description: V$SOX15_01: SOX-20<br>Possible TFs: SOX15	*3.30426*	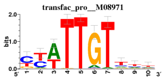	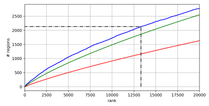	link	link	PWMs
103	☐ dbcorrdb__RBBP5__ENCSR000AQC_1__m5<br>Description: RBBP5 (ENCSR000AQC-1, motif 5)<br>Possible TFs: RBBP5	*3.29343*	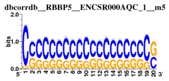	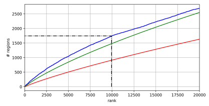	link	link	PWMs
104	☐ E053-H3K4me1-broadpeak<br>Description: H3K4me1 in Cortex derived primary cultured neurospheres (E053, broadpeak)	*3.27222*		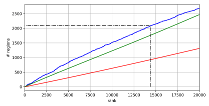	link	link	Histone modifications
105	☐ transfac_pro__M03876<br>Description: V$KAISO_Q2: Kaiso<br>Possible TFs: ZBTB33	*3.27096*	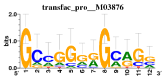	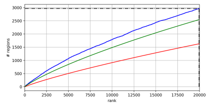	link	link	PWMs
106	☐ dbcorrdb__MXI1__ENCSR000EIA_1__m4<br>Description: MXI1 (ENCSR000EIA-1, motif 4)<br>Possible TFs: MXI1	*3.26275*	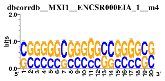	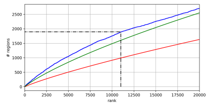	link	link	PWMs
107	☐ ENCFF001UUO<br>Description: DNase-seq on human ECC-1 treated with estradiol at 10nM for 30 minutes. Note: This experiment previously referred to its biosample as ECC-1, however it has been found that all currently available ECC-1 are actually Ishikawa cells (http://www.ncbi.nlm.nih.gov/pmc/articles/PMC3432677/).	*3.25763*		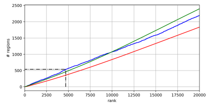	link	link	DHS & FAIRE
108	☐ ENCFF001WZF<br>Description: H3K27me3 ChIP-seq on human H7-hESC differentiated 14 days	*3.25403*		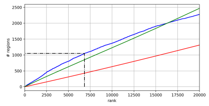	link	link	Histone modifications
109	☐ dbcorrdb__ZMIZ1__ENCSR000EFQ_1__m1<br>Description: ZMIZ1 (ENCSR000EFQ-1, motif 1)<br>Possible TFs: ZMIZ1	*3.25299*	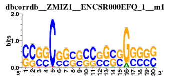	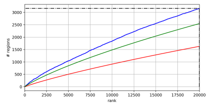	link	link	PWMs

#	Feature	NES	Logo	Recovery Curve	Candidate targets	All regions in top 20000	Database
110	☐ E069-H3K27ac-broadpeak<br>Description: H3K27ac in Brain Cingulate Gyrus (E069, broadpeak)	*3.24613*		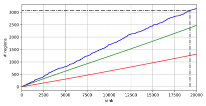	**link**	**link**	Histone modifications
111	☐ dbcorrdb__RAD21__ENCSR000EHX_1__m7<br>Description: RAD21 (ENCSR000EHX-1, motif 7)<br>Possible TFs: RAD21	*3.23977*	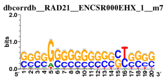	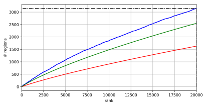	**link**	**link**	PWMs
112	☐ cisbp__M1594<br>Description: SOX12[gene ID: "ENSG00000177732" species: "Homo sapiens" TF status: "inferred" TF family: "Sox" DBDs: "HMG_box"]; Sox12[gene ID: "ENSMUSG00000051817" species: "Mus musculus" TF status: "direct" TF family: "Sox" DBDs: "HMG_box"]<br>Possible TFs: SOX12	*3.23597*	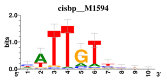	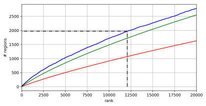	**link**	**link**	PWMs
113	☐ predrem__nrMotif95<br>Description: 260_H9_P42-DS18522.M174	*3.23410*	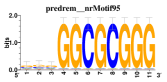	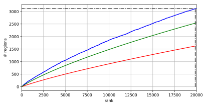	**link**	**link**	PWMs
114	☐ elemento__CCCGCGC<br>Description: Conserved regulatory element CCCGCGC between Hs and Mm	*3.23090*	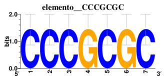	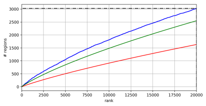	**link**	**link**	PWMs
115	☐ E070-H3K4me1<br>Description: H3K4me1 in Brain Germinal Matrix (E070,)	*3.22461*		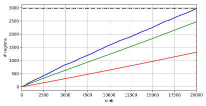	**link**	**link**	Histone modifications
116	☐ transfac_pro__M02281<br>Description: V$SP1_03: SP1<br>Possible TFs: SP1	*3.21938*	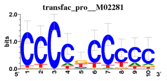	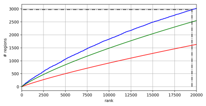	**link**	**link**	PWMs
117	☐ tfdimers__MD00569<br>Description: M00932_forward_10_M00716_forward dimer: Sp1 / ZF5<br>Possible TFs: SP1, SP2, SP3, SP4, ZBTB14	*3.21101*	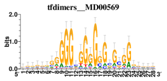	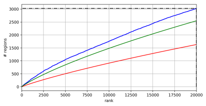	**link**	**link**	PWMs
118	☐ cisbp__M1601<br>Description: SOX11[gene ID: "ENSG00000176887" species: "Homo sapiens" TF status: "inferred" TF family: "Sox" DBDs: "HMG_box"]; Sox11[gene ID: "ENSMUSG00000063632" species: "Mus musculus" TF status: "direct" TF family: "Sox" DBDs: "HMG_box"]<br>Possible TFs: SOX11	*3.20793*	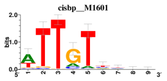	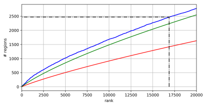	**link**	**link**	PWMs
119	☐ dbcorrdb__EZH2__ENCSR000ARK_1__m4<br>Description: EZH2 (ENCSR000ARK-1, motif 4)<br>Possible TFs: EZH2	*3.20423*	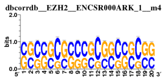	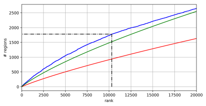	**link**	**link**	PWMs
120	☐ homer__CCWTTGTY_Sox3<br>Description: Sox3(HMG)/NPC-Sox3-ChIP-Seq(GSE33059)/Homer<br>Possible TFs: SOX3	*3.19834*	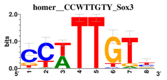	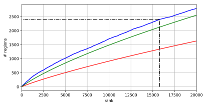	**link**	**link**	PWMs
121	☐ taipale_cyt_meth__E2F2_NCGCGCGCGCM_eDBD_meth<br>Description: E2F2 [E2F, CpG-meth]<br>Possible TFs: E2F2	*3.18597*	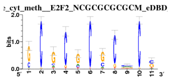	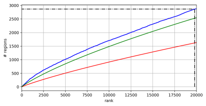	**link**	**link**	PWMs
122	☐ hdpi__ANXA11<br>Description: ANXA11<br>Possible TFs: ANXA11	*3.18389*	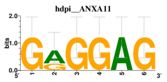	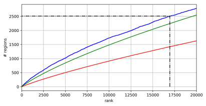	**link**	**link**	PWMs

#	Feature	NES	Logo	Recovery Curve	Candidate targets	All regions in top 20000	Database
123	☐ cisbp__M1592<br>Description: SOX3[gene ID: "ENSG00000134595" species: "Homo sapiens" TF status: "inferred" TF family: "Sox" DBDs: "HMG_box"]; Sox3[gene ID: "ENSMUSG00000045179" species: "Mus musculus" TF status: "direct" TF family: "Sox" DBDs: "HMG_box"]<br>Possible TFs: SOX3	*3.18044*	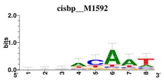	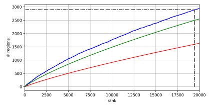	link	link	PWMs
124	☐ transfac_pro__M07397<br>Description: V$ZBP89_Q4_01: ZBP89<br>Possible TFs: ZNF148	*3.17866*	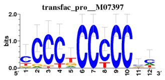	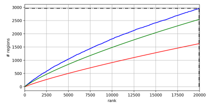	link	link	PWMs
125	☐ cisbp__M1789<br>Description: RSC30[gene ID: "YHR056C" species: "Saccharomyces cerevisiae" TF status: "direct" TF family: "Zinc cluster" DBDs: "Zn_clus"]	*3.17340*	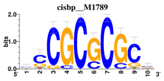	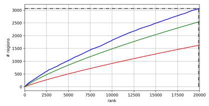	link	link	PWMs
126	☐ hocomoco__VEZF1_HUMAN.H11MO.1.C<br>Description: VEZF1_HUMAN<br>Possible TFs: VEZF1	*3.17071*	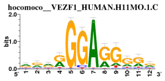	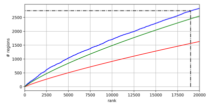	link	link	PWMs
127	☐ transfac_pro__M07040<br>Description: V$GKLF_Q3: GKLF<br>Possible TFs: KLF4	*3.16706*	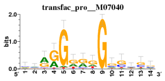	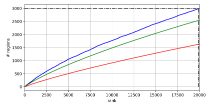	link	link	PWMs
128	☐ ENCFF001SPJ<br>Description: DNase-seq on human osteoblast	*3.16593*		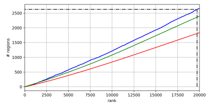	link	link	DHS & FAIRE
129	☐ E007-H3K4me2-broadpeak<br>Description: H3K4me2 in H1 Derived Neuronal Progenitor Cultured Cells (E007, broadpeak)	*3.15966*		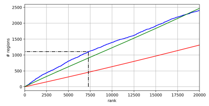	link	link	Histone modifications
130	☐ E097-H3K4me3-broadpeak<br>Description: H3K4me3 in Ovary (E097, broadpeak)	*3.15521*		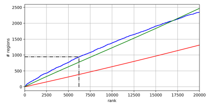	link	link	Histone modifications
131	☐ hdpi__PTPMT1<br>Description: PTPMT1<br>Possible TFs: PTPMT1	*3.15520*	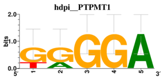	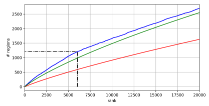	link	link	PWMs
132	☐ ENCFF001SPF<br>Description: DNase-seq on human MCF-7 treated with hypoxia lactic acid	*3.15508*		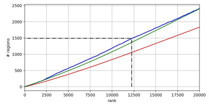	link	link	DHS & FAIRE
133	☐ hocomoco__ZBT17_MOUSE.H11MO.0.A<br>Description: ZBT17_MOUSE<br>Possible TFs: ZBTB17	*3.15480*	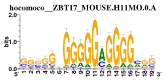	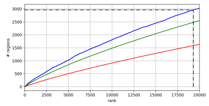	link	link	PWMs
134	☐ transfac_pro__M01219<br>Description: V$SP1SP3_Q4: SP1:SP3<br>Possible TFs: SP1, SP3	*3.15458*	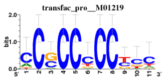	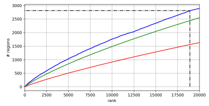	link	link	PWMs
135	☐ transfac_pro__M03577<br>Description: V$PAX5_Q6: Pax-5<br>Possible TFs: PAX5	*3.15375*	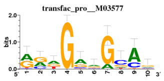	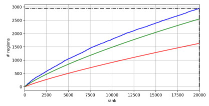	link	link	PWMs

#	Feature	NES	Logo	Recovery Curve	Candidate targets	All regions in top 20000	Database
136	☐ predrem__nrMotif1200<br>Description: 63_fIntestine_Lg-DS17841.M122	*3.15182*	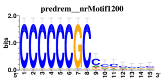	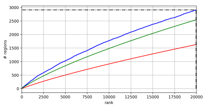	**link**	**link**	PWMs
137	☐ ENCFF001SPQ<br>Description: DNase-seq on human T-47D	*3.14868*		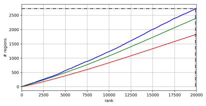	**link**	**link**	DHS & FAIRE
138	☐ elemento__CTCCCCG<br>Description: Conserved regulatory element CTCCCCG between Hs and Mm	*3.14575*	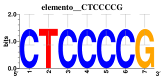	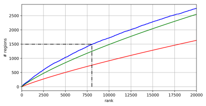	**link**	**link**	PWMs
139	☐ hocomoco__SOX9_MOUSE.H11MO.1.A<br>Description: SOX9_MOUSE<br>Possible TFs: SOX9	*3.14548*	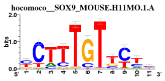	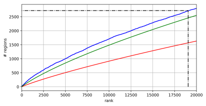	**link**	**link**	PWMs
140	☐ cisbp__M1595<br>Description: Sox6[gene ID: "ENSMUSG00000051910" species: "Mus musculus" TF status: "direct" TF family: "Sox" DBDs: "HMG_box"]<br>Possible TFs: SOX6	*3.14422*	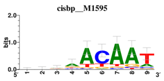	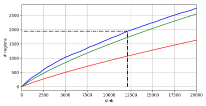	**link**	**link**	PWMs
141	☐ cisbp__M6535<br>Description: WT1[gene ID: "ENSG00000184937" species: "Homo sapiens" TF status: "direct" TF family: "C2H2 ZF" DBDs: "zf-C2H2"]<br>Possible TFs: WT1	*3.14406*	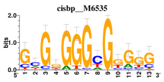	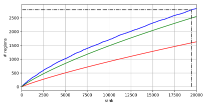	**link**	**link**	PWMs
142	☐ cisbp__M1599<br>Description: SOX1[gene ID: "ENSG00000182968" species: "Homo sapiens" TF status: "inferred" TF family: "Sox" DBDs: "HMG_box"]; Sox21[gene ID: "ENSMUSG00000061517" species: "Mus musculus" TF status: "direct" TF family: "Sox" DBDs: "HMG_box"]; Sox21a[gene ID: "FBgn0036411" species: "Drosophila melanogaster" TF status: "inferred" TF family: "Sox" DBDs: "HMG_box"]; Sox21b[gene ID: "FBgn0042630" species: "Drosophila melanogaster" TF status: "inferred" TF family: "Sox" DBDs: "HMG_box"]<br>Possible TFs: SOX21	*3.14126*	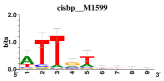	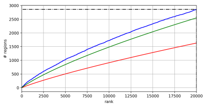	**link**	**link**	PWMs
143	☐ cisbp__M1597<br>Description: SOX1[gene ID: "ENSG00000182968" species: "Homo sapiens" TF status: "inferred" TF family: "Sox" DBDs: "HMG_box"]; Sox14[gene ID: "ENSMUSG00000053747" species: "Mus musculus" TF status: "direct" TF family: "Sox" DBDs: "HMG_box"]<br>Possible TFs: SOX14	*3.14039*	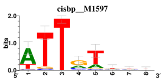	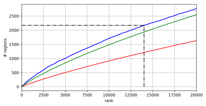	**link**	**link**	PWMs
144	☐ elemento__CCCCGCG<br>Description: Conserved regulatory element CCCCGCG between Hs and Mm	*3.13867*	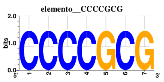	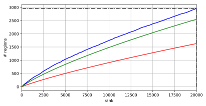	**link**	**link**	PWMs
145	☐ cisbp__M6552<br>Description: ZNF148[gene ID: "ENSG00000163848" species: "Homo sapiens" TF status: "direct" TF family: "C2H2 ZF" DBDs: "zf-C2H2"]; ZNF281[gene ID: "ENSG00000162702" species: "Homo sapiens" TF status: "inferred" TF family: "C2H2 ZF" DBDs: "zf-C2H2"]; Zfp148[gene ID: "ENSMUSG00000022811" species: "Mus musculus" TF status: "inferred" TF family: "C2H2 ZF" DBDs: "zf-C2H2"]<br>Possible TFs: ZNF148	*3.13234*	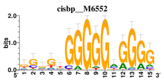	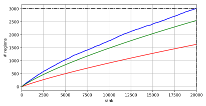	**link**	**link**	PWMs
146	☐ cisbp__M1216<br>Description: M1216	*3.12695*	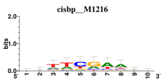	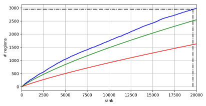	**link**	**link**	PWMs

#	Feature	NES	Logo	Recovery Curve	Candidate targets	All regions in top 20000	Database
147	☐ transfac_pro__M07297<br>Description: V$MAZ_Q5: MAZ<br>Possible TFs: MAZ	*3.12181*	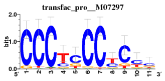	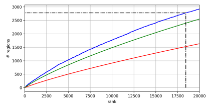	**link**	**link**	PWMs
148	☐ hocomoco__ZN148_HUMAN.H11MO.0.D<br>Description: ZN148_HUMAN<br>Possible TFs: ZNF148	*3.11574*	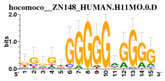	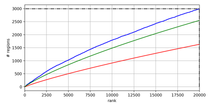	**link**	**link**	PWMs
149	☐ elemento__CGCGCCC<br>Description: Conserved regulatory element CGCGCCC between Hs and Mm	*3.11506*	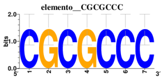	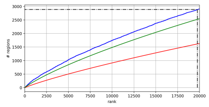	**link**	**link**	PWMs
150	☐ transfac_pro__M00649<br>Description: V$MAZ_Q6: MAZ<br>Possible TFs: MAZ	*3.10979*	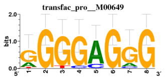	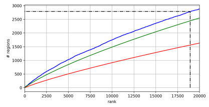	**link**	**link**	PWMs
151	☐ transfac_pro__M07289<br>Description: V$GKLF_Q3_01: GKLF<br>Possible TFs: KLF4	*3.10562*	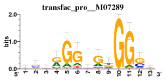	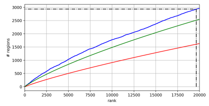	**link**	**link**	PWMs
152	☐ predrem__nrMotif657<br>Description: 231_fSpinal_cord-DS20530.M129	*3.10550*	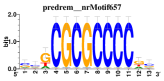	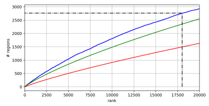	**link**	**link**	PWMs
153	☐ hocomoco__SP1_MOUSE.H11MO.0.A<br>Description: SP1_MOUSE<br>Possible TFs: SP1	*3.10230*	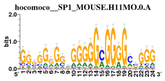	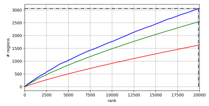	**link**	**link**	PWMs
154	☐ hdpi__GRHPR<br>Description: GRHPR<br>Possible TFs: GRHPR	*3.10015*	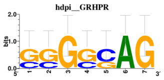	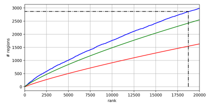	**link**	**link**	PWMs
155	☐ cisbp__M0140<br>Description: M0140	*3.09786*	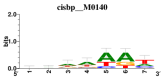	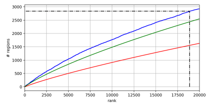	**link**	**link**	PWMs
156	☐ elemento__CGAGCGC<br>Description: Conserved regulatory element CGAGCGC between Hs and Mm	*3.09561*	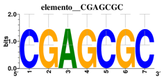	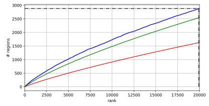	**link**	**link**	PWMs
157	☐ transfac_pro__M07277<br>Description: V$BTEB2_Q3_01: BTEB2<br>Possible TFs: KLF5	*3.09389*	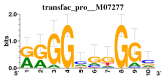	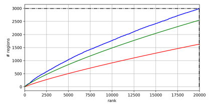	**link**	**link**	PWMs
158	☐ ENCFF001XEK<br>Description: H3K27me3 ChIP-seq on human HRE	*3.09388*		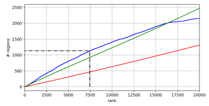	**link**	**link**	Histone modifications
159	☐ dbcorrdb__RCOR1__ENCSR000EFG_1__m5<br>Description: RCOR1 (ENCSR000EFG-1, motif 5)<br>Possible TFs: RCOR1	*3.09130*	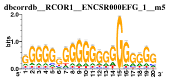	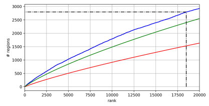	**link**	**link**	PWMs
160	☐ dbcorrdb__SREBF1__ENCSR000DYU_1__m4<br>Description: SREBF1 (ENCSR000DYU-1, motif 4)<br>Possible TFs: SREBF1	*3.08625*	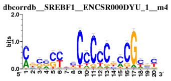	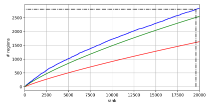	**link**	**link**	PWMs

#	Feature	NES	Logo	Recovery Curve	Candidate targets	All regions in top 20000	Database
161	cisbp__M0499<br>Description: M0499	*3.08479*	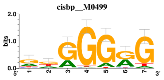	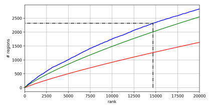	link	link	PWMs
162	neph__UW.Motif.0009<br>Description: ccccNcccc	*3.08385*	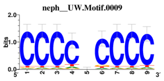	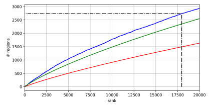	link	link	PWMs
163	E073-H3K4me1-broadpeak<br>Description: H3K4me1 in Brain_Dorsolateral_Prefrontal_Cortex (E073, broadpeak)	*3.07875*		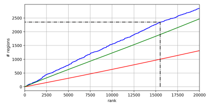	link	link	Histone modifications
164	cisbp__M4300<br>Description: RSC30[gene ID: "YHR056C" species: "Saccharomyces cerevisiae" TF status: "direct" TF family: "Zinc cluster" DBDs: "Zn_clus"]	*3.07694*	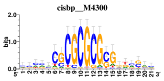	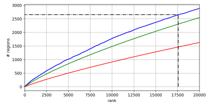	link	link	PWMs
165	cisbp__M1593<br>Description: Sox18[gene ID: "ENSMUSG00000046470" species: "Mus musculus" TF status: "direct" TF family: "Sox" DBDs: "HMG_box"]<br>Possible TFs: SOX18	*3.07650*	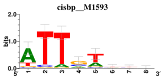	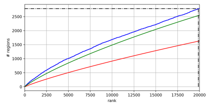	link	link	PWMs
166	transfac_pro__M01520<br>Description: F$RSC30_01: Rsc30p	*3.07624*	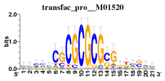	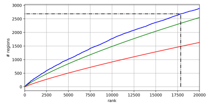	link	link	PWMs
167	transfac_pro__M04888<br>Description: V$TAF1_05: TAFII250<br>Possible TFs: TAF1	*3.07581*	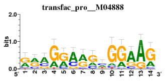	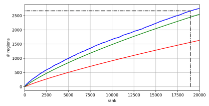	link	link	PWMs
168	E002-H3K4me3<br>Description: H3K4me3 in ES-WA7 Cells (E002,)	*3.07414*		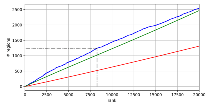	link	link	Histone modifications
169	hdpi__TRIM21<br>Description: TRIM21<br>Possible TFs: TRIM21	*3.07384*	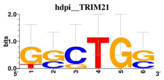	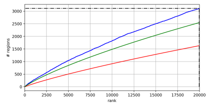	link	link	PWMs
170	predrem__nrMotif717<br>Description: 108_fKidney_renal_cortex_R-DS17545.M43	*3.06410*	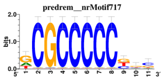	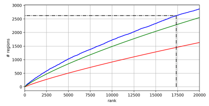	link	link	PWMs
171	tfdimers__MD00391<br>Description: M01588_forward_8_M01113_forward dimer: GKLF (KLF4) / CACD<br>Possible TFs: KLF4	*3.06377*	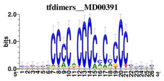	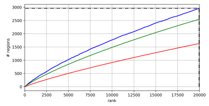	link	link	PWMs
172	transfac_pro__M00720<br>Description: V$CACBINDINGPROTEIN_Q6: CAC-binding protein	*3.06365*	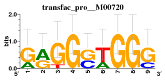	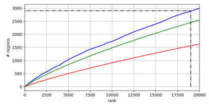	link	link	PWMs
173	factorbook__ZNF281<br>Description: ZNF281<br>Possible TFs: ZNF281	*3.06262*	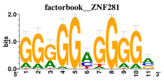	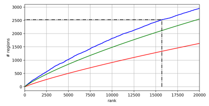	link	link	PWMs
174	cisbp__M0120<br>Description: M0120	*3.06087*	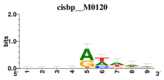	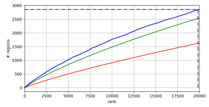	link	link	PWMs

#	Feature	NES	Logo	Recovery Curve	Candidate targets	All regions in top 20000	Database
175	☐ hdpi__AFF4<br>Description: AFF4<br>Possible TFs: AFF4	*3.05834*	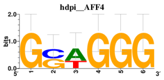	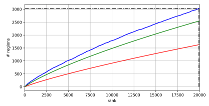	**link**	**link**	PWMs
176	☐ cisbp__M1781<br>Description: RSC3[gene ID: "YDR303C" species: "Saccharomyces cerevisiae" TF status: "direct" TF family: "Zinc cluster" DBDs: "Zn_clus"]	*3.05689*	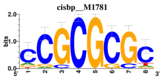	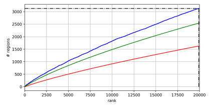	**link**	**link**	PWMs
177	☐ jaspar__MA0442.1<br>Description: SOX10<br>Possible TFs: SOX10	*3.05394*	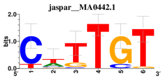	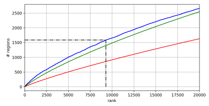	**link**	**link**	PWMs
178	☐ ENCFF001SPL<br>Description: Duke human PanIslets DNase-seq	*3.04851*		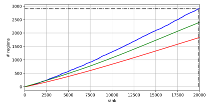	**link**	**link**	DHS & FAIRE
179	☐ predrem__nrMotif2584<br>Description: 201_fMuscle_lower_limb-DS18174.M483	*3.04476*	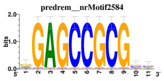	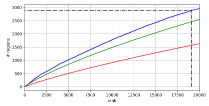	**link**	**link**	PWMs
180	☐ ENCFF001UVT<br>Description: DNase-seq on human Hepatocytes	*3.04370*		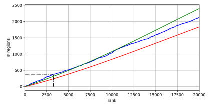	**link**	**link**	DHS & FAIRE
181	☐ tfdimers__MD00154<br>Description: M01173_reverse_7_M00083_forward dimer: SREBP1 / MZF1<br>Possible TFs: SREBF1, MZF1	*3.04159*	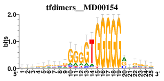	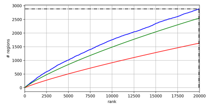	**link**	**link**	PWMs
182	☐ hdpi__NXPH3<br>Description: NXPH3<br>Possible TFs: NXPH3	*3.03988*	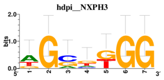	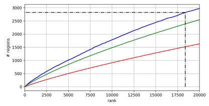	**link**	**link**	PWMs
183	☐ transfac_pro__M00982<br>Description: V$KROX_Q6: KROX<br>Possible TFs: EGR2, EGR3, EGR1, EGR4	*3.03953*	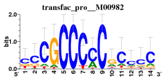	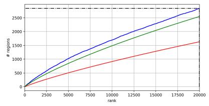	**link**	**link**	PWMs
184	☐ hdpi__RBM17<br>Description: RBM17<br>Possible TFs: RBM17	*3.03711*	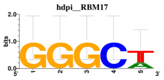	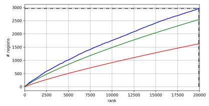	**link**	**link**	PWMs
185	☐ cisbp__M1600<br>Description: Sox7[gene ID: "ENSMUSG00000063060" species: "Mus musculus" TF status: "direct" TF family: "Sox" DBDs: "HMG_box"]	*3.03164*	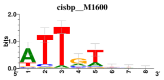	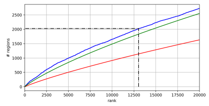	**link**	**link**	PWMs
186	☐ ENCFF001SPA<br>Description: DNase-seq on human Huh-7	*3.03125*		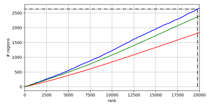	**link**	**link**	DHS & FAIRE
187	☐ taipale_cyt_meth__ZNF444_NCRTCCCCCTCCCCCN_FL<br>Description: ZNF444 [SCAN_Znf_C2H2]<br>Possible TFs: ZNF444	*3.02963*	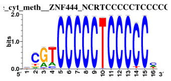	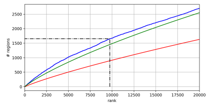	**link**	**link**	PWMs
188	☐ hdpi__TCEAL6<br>Description: TCEAL6<br>Possible TFs: TCEAL6	*3.02810*	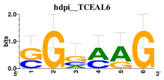	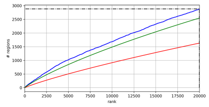	**link**	**link**	PWMs

#	Feature	NES	Logo	Recovery Curve	Candidate targets	All regions in top 20000	Database
189	☐ cisbp__M0130<br>Description: ORC2[gene ID: "YBR060C" species: "Saccharomyces cerevisiae" TF status: "inferred" TF family: "AT hook" DBDs: "AT_hook"]; Setbp1[gene ID: "ENSMUSG00000024548" species: "Mus musculus" TF status: "direct" TF family: "AT hook" DBDs: "AT_hook"]<br>Possible TFs: SETBP1	3.02406	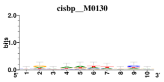	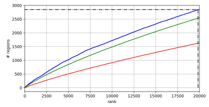	link	link	PWMs
190	☐ cisbp__M0144<br>Description: ORC2[gene ID: "YBR060C" species: "Saccharomyces cerevisiae" TF status: "inferred" TF family: "AT hook" DBDs: "AT_hook"]	3.02399	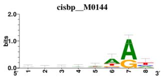	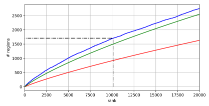	link	link	PWMs
191	☐ hocomoco__MAZ_HUMAN.H11MO.0.A<br>Description: MAZ_HUMAN<br>Possible TFs: MAZ	3.02335	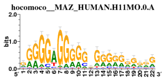	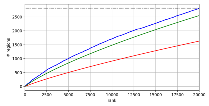	link	link	PWMs
192	☐ transfac_pro__M07436<br>Description: V$WT1_Q4: WT1<br>Possible TFs: WT1	3.02290	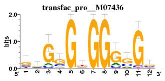	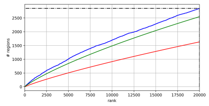	link	link	PWMs
193	☐ dbcorrdb__HA-E2F1__ENCSR000EVM_1__m1<br>Description: HA-E2F1 (ENCSR000EVM-1, motif 1)<br>Possible TFs: E2F1	3.02251	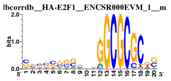	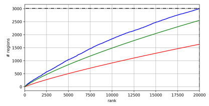	link	link	PWMs
194	☐ transfac_pro__M01122<br>Description: V$ZNF219_01: ZNF219<br>Possible TFs: ZNF219	3.02094	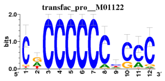	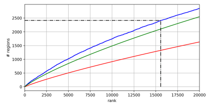	link	link	PWMs
195	☐ ENCFF001UXQ<br>Description: Duke human SK-N-SH DNase-seq	3.01402		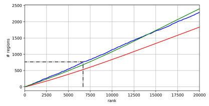	link	link	DHS & FAIRE
196	☐ hdpi__LUZP1<br>Description: LUZP1<br>Possible TFs: LUZP1	3.01399	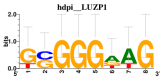	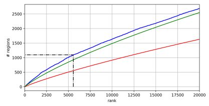	link	link	PWMs
197	☐ transfac_pro__M08899<br>Description: V$SOX_Q4: SOX<br>Possible TFs: SOX18, SOX21, SOX30, SRY, SOX10, SOX11, SOX13, SOX15, HBP1, SOX2, SOX8, SOX9, SOX17, SOX3, SOX1, SOX6, SOX7, SOX4, SOX5	3.01297	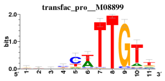	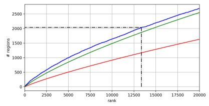	link	link	PWMs
198	☐ transfac_pro__M05512<br>Description: V$ZNF575_01: ZNF575<br>Possible TFs: ZNF575	3.01013	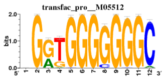	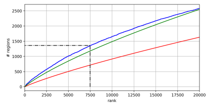	link	link	PWMs
199	☐ dbcorrdb__SREBF2__ENCSR000EZO_1__m1<br>Description: SREBF2 (ENCSR000EZO-1, motif 1)<br>Possible TFs: SREBF2	3.00826	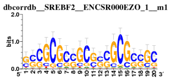	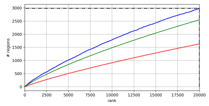	link	link	PWMs
200	☐ cisbp__M0122<br>Description: Hmga1-rs1[gene ID: "ENSMUSG00000078249" species: "Mus musculus" TF status: "inferred" TF family: "AT hook" DBDs: "AT_hook"]; Hmga1[gene ID: "ENSMUSG00000046711" species: "Mus musculus" TF status: "inferred" TF family: "AT hook" DBDs: "AT_hook"]	3.00820	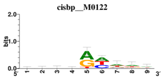	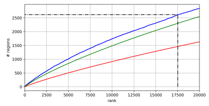	link	link	PWMs
201	☐ elemento__CGCCCGC<br>Description: Conserved regulatory element CGCCCGC between Hs and Mm	3.00732	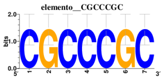	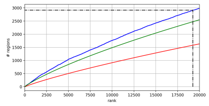	link	link	PWMs

#	Feature	NES	Logo	Recovery Curve	Candidate targets	All regions in top 20000	Database
202	☐ transfac_pro__M01175<br>Description: V$CKROX_Q2: CKROX<br>Possible TFs: ZBTB7B	*3.00718*	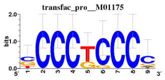	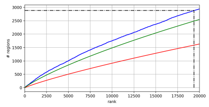	**link**	**link**	PWMs
203	☐ transfac_pro__M07039<br>Description: V$ETF_Q6_01: ETF<br>Possible TFs: TEAD2	*3.00636*	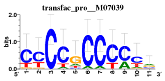	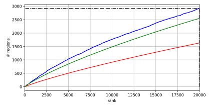	**link**	**link**	PWMs
204	☐ swissregulon__sacCer__RSC30<br>Description: sacCer__RSC30	*3.00571*	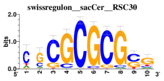	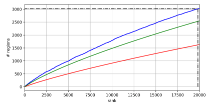	**link**	**link**	PWMs
205	☐ cisbp__M6471<br>Description: SOX13[gene ID: "ENSG00000143842" species: "Homo sapiens" TF status: "direct" TF family: "Sox" DBDs: "HMG_box"]<br>Possible TFs: SOX13	*3.00568*	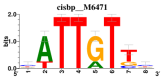	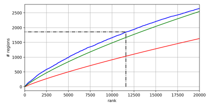	**link**	**link**	PWMs
206	☐ cisbp__M0132<br>Description: Hmga1-rs1[gene ID: "ENSMUSG00000078249" species: "Mus musculus" TF status: "inferred" TF family: "AT hook" DBDs: "AT_hook"]; Hmga1[gene ID: "ENSMUSG00000046711" species: "Mus musculus" TF status: "inferred" TF family: "AT hook" DBDs: "AT_hook"]; Hmga2[gene ID: "ENSMUSG00000056758" species: "Mus musculus" TF status: "direct" TF family: "AT hook" DBDs: "AT_hook"]<br>Possible TFs: HMGA2	*3.00284*	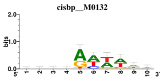	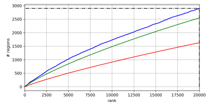	**link**	**link**	PWMs
207	☐ hdpi__SMAP1L<br>Description: SMAP1L<br>Possible TFs: SMAP2	*3.00220*	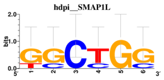	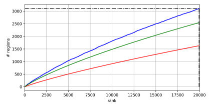	**link**	**link**	PWMs
208	☐<br>taipale_cyt_meth__ZNF444_NCRTCCCCCTCCCCCN_FL_meth_repr<br>Description: ZNF444 [SCAN_Znf_C2H2, CpG-meth]<br>Possible TFs: ZNF444	*3.00088*	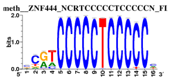	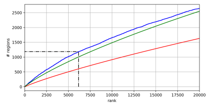	**link**	**link**	PWMs

© 2015-2019 **KULeuven** | Authors: Gert Hulselmans & Hana Imrichova | Web design (CSS) by **Andreas Viklund**.
