# Supplementary material for: Chromatin accessibility and regulatory vocabulary across indicine cattle tissues
Source: Genome Biol. 2021 Sep 21;22:273. doi: 10.1186/s13059-021-02489-7 (PMC8454054; doi:10.1186/s13059-021-02489-7)
Supplement: Supplementary file 20 — Additional file 20. Peer review history. [file 13059_2021_2489_MOESM20_ESM.docx]

**Review history**

**First round of review**

**Reviewer 1**

This manuscript describes an analysis of chromatin accessibility in three tissues of tropical indicine cattle. Liver, muscle and hypothalamus samples from 3 animals of the Bos taurus indicus subspecies (zebu breed) have been processed by ATAC-seq. A comparative analysis of the ATAC-seq data is presented between tissues. Analysis steps include peak calling, differential analysis of accessibility, motif enrichment analysis, gene assignment of the peaks and integration with expression data. The purpose of the study, the experimental design and the results all make sense. The paper is well written, although several parts would benefit from more detailed explanations. The motif discovery and gene expression network analyses produce nice and impressive results.

I have a couple of issues about the methods, that could have led to incorrect conclusions. Also, I am not sure whether these results are of interest to a broad readership beyond the field of cattle genomics, although this is to be estimated by the editorial team.

Major concerns

1. Methods should be better documented. For several steps of the computational/statistical analysis the name of a tool is mentioned but the version number, the specific command/function and the parameters are missing. I understand that the entire code does not have to be provided, yet simply mentioning an R package is not enough. Reproducibility is important.

2. The number of peaks is highly variable across samples, even within the same tissue. More than 4x between muscle samples and 2x in liver. Even after merging the samples per tissue there are still twice as many peaks in HYP vs. MUSC. The possible reasons of such a discrepancy should be mentioned and commented. For instance the discussion mentions the difference between HYP and the other tissues. Could everything be due to a higher number of peaks? Also, in Sup Table 1 only read statistics are shown. Could the authors provide a table that for each sample and for each "merged" sample (per tissue and for the grand total) indicates the number of reads, the number of peaks, the average peak length and the proportion of peaks near the TSS?

3. I believe that there is an issue with the way Tissue-Specific (TS) peaks have been obtained.

Typically, to define if a given peak (from one tissue) should be qualified as "tissue-specific" or not, what is usually done is to look for an overlap with the peaks from the other tissues. The peak is labelled TS is and only if no overlap is found. Doing this for each peak of each tissue gives a set of TS peaks, with each TS peak being an actual peak that was called from reads in a (merged) sample. Consistently, the text seems to describe this process: "we defined [...] peaks exclusive to one tissue as tissue-specific" (line 375, methods). A simple way to do this is to use the intersectBed function from the bedtools suite with the -v option.
However, this is not what has been done here. It seems that instead of defining if called peaks were TS or not, genomic regions have been subtracted from each other in order to identify tissue-specific sub-intervals instead of actual peaks. This leads to several issues. First, the resulting genomic intervals cannot be called "peaks" because nothing ensures that they actually contain the summit of a peak. For instance, if a small peak is entirely included within a larger peak, the resulting subtraction would produce two flanking intervals on each side of the small peak. These intervals artificially inflate the number of resulting "TS peaks" although none of them is an actual peak. Even worse, they correspond to peak tails, which is not biologically relevant.

I think that this issue can explain several strange biases in the results, in particular in Figure 2:
- According to Fig 1A, peaks in HYP seem larger than in MUS and LIV. Consequently, when the peaks are aligned, as expected at TSSs for instance, subtracting the corresponding intervals is expected to generate trailing peak fragments more frequently in HYP. This could explain the HYP-TS pattern in Fig 2A better than a biological "footprint". A footprint effect is possible with ATAC-seq data, but it is visible at the level of the mapped reads (the peak level is too low resolution). Considering the difference in peak length this would also explain why the "footprint" is only visible for HYP.
- As I explained, not only does peak fragmentation result in spurious peaks but it also artificially inflates the number of peaks. Since this is expected to happen more often at TSSs, it could explain why the proportion of peaks in promoters is much higher for HYP-TS "peaks" vs. MUS or LIV in Figure 2B.

This issue can be addressed by assigning correctly the TS status to actual peaks, by iteratively using "bedtools interesct -v" commands for instance.
Then all subsequent analyses should be redone. Depending on the results, some parts of the conclusion should be rewritten accordingly, in particular the one about the footprinting hypothesis (if disproved). The methods section should be updated too of course.

4. Similarly, the way the authors describe "constitutive peaks" is misleading too. For instance, line 140 (results) "Peaks consistently appearing in all three tissues were considered constitutive" and line 374 (methods) "we defined peak appearing in all tissues as constitutive". This suggests that *the same peak* has to be detected in the three tissues to be qualified as constitutive. The same peak implies either the same top position or the same interval. I believe that what has been done is an intersection of the three intervals and that the resulting genomic regions are called "constitutive peaks", although they do not necessarily correspond to a "peak". The way this is done is not as much of an issue as it is for tissue-specific peaks in terms of potential bias on the subsequent results, and I understand that it is convenient to call these regions "constitutive peaks", but the distinction should be made clear at least in the methods before referring to these regions as "peaks".

5. There is a serious problem with Figure 3. I cannot figure out what is going on with the MA plots (3B-D). Could the authors explain why they seem so atypical? It looks like a subset of the dots are missing (along the 0 logFC axis). Also, the fact that high counts gather around a constant logFC value to form these horizontal spikes is very odd. I can only think about a batch effect, a threshold effect or an issue about the normalization. How were count values normalized? This is unfortunately not specified in the methods. In terms of normalization I think it is important to use a method that has been shown to be relevant with ATAC-seq data. I would recommend the cyclic loess method from the csaw package. It generates MA plots before and after normalization, that could be compared with the one from Fig 3. It would be interesting to see the HAC in Fig 3A before and after normalization too.

6. Sequencing data has to be made available. Please provide the accession numbers of the reads.

Minor issues

Sup Table 1: Duplicates numbers are missing. Was there really no PCR duplicate at all? ). Also, number and proportion of reads on the MT chromosome?

119: Not clear how the 169,301 peaks were obtained. Were the 6 bam files merged and MACS2 used to call peaks on the mapped reads? Were the 3 tissue peak bed files merged? Please precise in the manuscript.

122: A  better description of Figure 1A is needed, in particular for the three upper panels. X-axis=? y-axis=? What does the color intensity represent? How are peaks ranked vertically? Where do the values come from? How is the figure generated? The upper and lower parts need to be described distinctively.

130: Where do the Fig 1C TFBSs come from? I could not find the corresponding part in the Methods.

164: Figure 3A is not described. How is the hierarchical clustering performed? The euclidian distance metric is used, but on which values exactly? Which linkage criterion?

167: To be considered as differential in a given tissue, a peak has to be detected in the two comparisons that involve this tissue, is that correct? Could you indicate the number of differential peaks for the 3 pairwise comparisons of Figure 3B-D in each tissue? It looks like 55,513 peaks significantly differ between liver and hypothalamus (line 177), yet only 1,101 qualify as differential for liver (line 107). Could you complete and comment these numbers?

180: 65% of the DE genes were associated with DO region. What about the non-DE genes?

195: are master regulators themselves expressed/highly expressed/DE in their respective tissues? 22% of the predicted targets are "co-expressed": is that a lot? How does this proportion compare with that of other (non target) genes? Are there more DE genes among these target genes that in expressed but non target genes?

222: What is the significance of these results? I do not understand the interest of this analysis.

348: The link to http://ftp.faang.ebi.ac.uk/ftp/protocols/assays/ does not work.

360: How were the peaks called? Parameters of the MACS2 command?

385: HTS-DESeq2: how were values normalized? How were p-values adjusted?

References: please check the bibliography format. Many references are incomplete and/or incorrectly formatted.

**Reviewer 2**

The manuscript reports the chromatin accessibility regions for three important tissues in Bos taurus indicus. The manuscript is clearly and concisely written for the majority of the text. The work is quite competently done and is a good contribution to the functional genomics in cattle. There are a number of small and a couple larger concerns with the work as presented, specifically the extent to which the authors use existing data on Bos taurus taurus, since they have a paragraph in the Introduction to breed comparisons. There is also apparently a Method section missing, as well as some minor editorial suggestions.

Scientific issues and questions.

For a paper that discusses a cross-breed comparison would be valuable to understand the differences between topical and other cattle is important (l. 73-79), I was expecting a comparison of the accessible regions between taurus and indicine. There has been at least liver data available for some time (2019, PMID: 31884969), as well as all of the three tissues analyzed here (2020, PMID: 33028202), all in Bos taurus so a direct comparison would have been possible. Potentially linking to the selective sweeps overlap would be an interesting story, greatly increasing the value of the reported data herein, and would increase the interest of the work for the Genome Biology audience. I also did not find a Methods section on the selective sweeps analysis.

Conclusions section- Certainly you have new data, I am unclear what "new insights" were revealed about "tissue-specificity of gene regulation"- please describe. Further, I do not know what "transcriptional control alphabet specific to the bovine indicine lineage" is; please expand, especially as you indicate in the next sentence that this is different from the "finding of open chromatin regions overlapping with signatures of selection". Since there was no detailed comparison across breeds, I don't see how you can talk about finding anything specific to indicine related to transcriptional control.

Figure 2A. The explanation for the unusual pattern of ATAC-seq reads just for hypothalamus was unsatisfying. Is it possible that this is caused by a difference in 5' end/ promoter usage affecting annotation in hypothalamus?

Minor issues

l. 180- "65% were associated with differential open chromatin regions" First, was this a statistically significant association? Second, was the direction of the ratio of DE of the gene the same direction of the Differential accessibility? I don't see that in Suppl Table 5.
l. 196 Is this 22% statistically significant?
l. 224 and 227- similarly to the comments for l. 180/196, what does "associated" mean, and was this overlap statistically significant?
L 252-255. This is a useful observation, but also shows the weakness in assuming that "nearest gene" to the peak is the target gene.
l. 267-276. Yes, but it is also true that the liver has quite a few immune-centric cell types, including Kupfer cells, T cells, and NK cells and there doesn't have to be an "immune response" in liver to predict that whole-tissue liver samples such as these could show open chromatin at genes annotated for immunity.

Small editorial items:
Figure 1C- I didn't find a Methods description for this work. Also a typo in this part of the figure legend.
l. 177 where should be were
l. 186- Define NES in text.
l. 248: I think you want to say "up to a few kilobases from a TSS" since by definition a promoter is directly adjacent to the TSS.
l. 374- please describe the criteria used to define (identify as corresponding) peaks as overlapping across tissues.

**Authors’ response to reviewers**

Reviewer #1:

This manuscript describes an analysis of chromatin accessibility in three tissues of tropical indicine cattle. Liver, muscle and hypothalamus samples from 3 animals of the Bos taurus indicus subspecies (zebu breed) have been processed by ATAC-seq. A comparative analysis of the ATAC-seq data is presented between tissues. Analysis steps include peak calling, differential analysis of accessibility, motif enrichment analysis, gene assignment of the peaks and integration with expression data. The purpose of the study, the experimental design and the results all make sense. The paper is well written, although several parts would benefit from more detailed explanations. The motif discovery and gene expression network analyses produce nice and impressive results.

I have a couple of issues about the methods, that could have led to incorrect conclusions. Also, I am not sure whether these results are of interest to a broad readership beyond the field of cattle genomics, although this is to be estimated by the editorial team.

Major concerns

Methods should be better documented. For several steps of the computational/statistical analysis the name of a tool is mentioned but the version number, the specific command/function and the parameters are missing. I understand that the entire code does not have to be provided, yet simply mentioning an R package is not enough. Reproducibility is important.

R: All the methods section was reviewed to improve reproducibility.

The number of peaks is highly variable across samples, even within the same tissue. More than 4x between muscle samples and 2x in liver. Even after merging the samples per tissue there are still twice as many peaks in HYP vs. MUSC. The possible reasons of such a discrepancy should be mentioned and commented. For instance the discussion mentions the difference between HYP and the other tissues. Could everything be due to a higher number of peaks? Also, in Sup Table 1 only read statistics are shown. Could the authors provide a table that for each sample and for each "merged" sample (per tissue and for the grand total) indicates the number of reads, the number of peaks, the average peak length and the proportion of peaks near the TSS?

R: As we will comment in more detail in the answer to question 5, we agree that differences in the number of peaks called for each biological replicate could be affecting the differential accessibility results. To overcome that, we repeated the analysis calling only peaks appearing in all three biological replicates. This provided us with a set of peaks per tissue that allowed us to confidently define tissue-specific peaks by direct comparison. But regarding differences in the number of peaks identified per tissue, the differences reported in our studies are not so different from other studies. For instance, Halstead et al. (2020, DOI: 10.1186/s12864-020-07078-9) found the same range of peaks between liver, muscle and hypothalamus of Bos taurus cattle, except that hypothalamus presented the lowest number (20,045) and muscle the highest (77,378). In Table 4 of this same study, we can see results for several other tissues in pigs, cattle and mouse and a highly variable number of peaks between tissues can be observed for all three species. Table 1 was created to summarize peak calling metrics as requested.

3.	I believe that there is an issue with the way Tissue-Specific (TS) peaks have been obtained.

Typically, to define if a given peak (from one tissue) should be qualified as "tissue- specific" or not, what is usually done is to look for an overlap with the peaks from the other tissues. The peak is labelled TS is and only if no overlap is found. Doing this for each peak of each tissue gives a set of TS peaks, with each TS peak being an actual peak that was called from reads in a (merged) sample. Consistently, the text seems to describe this process: "we defined [...] peaks exclusive to one tissue as tissue-specific" (line 375, methods). A simple way to do this is to use the intersectBed function from the bedtools suite with the -v option.

However, this is not what has been done here. It seems that instead of defining if called peaks were TS or not, genomic regions have been subtracted from each other in order to identify tissue-specific sub-intervals instead of actual peaks. This leads to several issues. First, the resulting genomic intervals cannot be called "peaks" because nothing ensures that they actually contain the summit of a peak. For instance, if a small peak is entirely included within a larger peak, the resulting subtraction would produce two flanking intervals on each side of the small peak. These intervals artificially inflate the number of resulting "TS peaks" although none of them is an actual peak. Even worse, they correspond to peak tails, which is not biologically relevant.

I think that this issue can explain several strange biases in the results, in particular in Figure 2:

-	According to Fig 1A, peaks in HYP seem larger than in MUS and LIV. Consequently, when the peaks are aligned, as expected at TSSs for instance, subtracting the corresponding intervals is expected to generate trailing peak fragments more frequently in HYP. This could explain the HYP-TS pattern in Fig 2A better than a biological "footprint". A footprint effect is possible with ATAC-seq data, but it is visible at the level of the mapped reads (the peak level is too low resolution). Considering the difference in peak length this would also explain why the "footprint" is only visible for HYP.

-	As I explained, not only does peak fragmentation result in spurious peaks but it also artificially inflates the number of peaks. Since this is expected to happen more often at TSSs, it could explain why the proportion of peaks in promoters is much higher for HYP-TS "peaks" vs. MUS or LIV in Figure 2B.

This issue can be addressed by assigning correctly the TS status to actual peaks, by iteratively using "bedtools interesct -v" commands for instance.

Then all subsequent analyses should be redone. Depending on the results, some parts of the conclusion should be rewritten accordingly, in particular the one about the footprinting hypothesis (if disproved). The methods section should be updated too of course.

R: You are absolutely right. We apologize for our mistake in the analysis and thank the reviewer for the valuable insights. The analyses were redone for tissue-specific peaks and all material and methods, results and discussion were updated. In terms of functional enrichment results and biological insights, changes were subtitle and appropriate adjustments were made to the manuscript.

4.	Similarly, the way the authors describe "constitutive peaks" is misleading too. For instance, line 140 (results) "Peaks consistently appearing in all three tissues were considered constitutive" and line 374 (methods) "we defined peak appearing in all tissues as constitutive". This suggests that *the same peak* has to be detected in the three tissues to be qualified as constitutive. The same peak implies either the same top position or the same interval. I believe that what has been done is an intersection of the three intervals and that the resulting genomic regions are called "constitutive peaks", although they do not necessarily correspond to a "peak". The way this is done is not as much of an issue as it is for tissue-specific peaks in terms of potential bias on the subsequent results, and I understand that it is convenient to call these regions "constitutive peaks", but the distinction should be made clear at least in the methods before referring to these regions as "peaks".

R: We agree that calling overlapping regions between the three tissues “peaks” can be misleading. We explained the difference between how TS peaks and constitutive “regions” were identified and replaced “constitutive peaks” with “constitutive regions” to make the difference clearer.

5.	There is a serious problem with Figure 3. I cannot figure out what is going on with the MA plots (3B-D). Could the authors explain why they seem so atypical? It looks like a subset of the dots are missing (along the 0 logFC axis). Also, the fact that high counts gather around a constant logFC value to form these horizontal spikes is very odd. I can only think about a batch effect, a threshold effect or an issue about the normalization. How were count values normalized? This is unfortunately not specified in the methods. In terms of normalization I think it is important to use a method that has been shown to be relevant with ATAC-seq data. I would recommend the cyclic loess method from the csaw package. It generates MA plots before and after normalization, that could be compared with the one from Fig 3. It would be interesting to see the HAC in Fig 3A before and after normalization too.

R: We tried different normalization methods as suggested but results were still looking atypical. We could only conclude that differences in the number of peaks called for each biological replicate was affecting the results. So we decided on a more robust method for peak calling: using Genrich (https://github.com/jsh58/Genrich) we called only “consensus” peaks, peaks appearing in all biological replicates. Then, we compared peaks across tissues using bedtools interesct -v, as suggested, to define tissue-specific peaks. This approach has been previously used to define tissue-specific peaks in Bos indicus (DOI: 10.1186/s12864-020-07078-9).

6.	Sequencing data has to be made available. Please provide the accession numbers of the reads.

R: Data was made available and the appropriate details were added to the manuscript. Minor issues

Sup Table 1: Duplicates numbers are missing. Was there really no PCR duplicate at all? ). Also, number and proportion of reads on the MT chromosome?

R: Requested information was added to Supplementary Table 1.

119: Not clear how the 169,301 peaks were obtained. Were the 6 bam files merged and MACS2 used to call peaks on the mapped reads? Were the 3 tissue peak bed files merged? Please precise in the manuscript.

167: To be considered as differential in a given tissue, a peak has to be detected in the two comparisons that involve this tissue, is that correct? Could you indicate the number of differential peaks for the 3 pairwise comparisons of Figure 3B-D in each tissue? It looks like 55,513 peaks significantly differ between liver and hypothalamus (line 177), yet only 1,101 qualify as differential for liver (line 107). Could you complete and comment these numbers?

R: In the revised version of the manuscript the differential peak analysis was removed, and tissue-specific peaks were defined by directly comparing consensus peaks in each tissue. That is because we believe that focusing only on peaks that appear in all biological replicates provide us with more trustable results. This method for defining tissue-specific peaks had been previously used in Bos taurus (DOI: 10.1186/s12864- 020-07078-9).

122: A better description of Figure 1A is needed, in particular for the three upper panels. X-axis=? y-axis=? What does the color intensity represent? How are peaks ranked vertically? Where do the values come from? How is the figure generated? The upper and lower parts need to be described distinctively.

R: Information was added to the figure, caption, and methods as recommended.

130: Where do the Fig 1C TFBSs come from? I could not find the corresponding part in the Methods.

R: TFBS actually correspond to peaks. The title and x-axis labels are the default of ChIPseeker plotDistToTSS function, but we agree that it is confusing. We have replaced both and improved the description of how the figure was generated in the methods section.

164: Figure 3A is not described. How is the hierarchical clustering performed? The euclidian distance metric is used, but on which values exactly? Which linkage criterion?

R: This figure is not present anymore in the revised version.

180: 65% of the DE genes were associated with DO region. What about the non-DE genes?

R: Considering that the differential expression analysis was only taking into account one of the three possible contrasts (since we do not have muscle RNAseq data) and that differential accessibility analysis was removed, we decided to use the RNAseq data only to test the co-expression of master tissue regulators and their target genes.

195: are master regulators themselves expressed/highly expressed/DE in their respective tissues? 22% of the predicted targets are "co-expressed": is thata lot? How does this proportion compare with that of other (non target) genes? Are there more DE genes among these target genes that in expressed but non target genes?

R: Our intention with the co-expression network was rather than generate a statistical value of significance for “validated” co-expressed target, to overlap different sources of information to identify potential targets with high confusability. ATAC-seq and RNA-seq data were not generated from the same samples and, therefore, not necessarily completely overlap. At the same time, predicting target genes based on human data have its limitations. Intersections between analyses are a way to indent more accurate results. In addition, we added to the results which of the top 5 enriched transcription factors in liver and hypothalamus were actually expressed within the tissues.

222: What is the significance of these results? I do not understand the interest of this analysis.

R: Indeed, revising the manuscript we agree that looking at signatures of selection without a direct comparison between Bos indicus and Bos taurus data was not producing relevant results. We now added the comparison between species and included the search for overlaps between indicine-specific peaks and indicine selective sweeps in this context.

348: The link to http://ftp.faang.ebi.ac.uk/ftp/protocols/assays/ does not work. R: A new link was provided

360: How were the peaks called? Parameters of the MACS2 command?

R: Peak calling was now performed using Genrich and a detailed description was added.

385: HTS-DESeq2: how were values normalized? How were p-values adjusted?

R: This analysis was no longer performed, but a description of how expression data was normalized for co-expression analysis was added.

References: please check the bibliography format. Many references are incomplete and/or incorrectly formatted.

R: Thanks for pointing that out, we fixed all the references.

Reviewer #2:

The manuscript reports the chromatin accessibility regions for three important tissues in Bos taurus indicus. The manuscript is clearly and concisely written for the majority of the text. The work is quite competently done and is a good contribution to the functional genomics in cattle. There are a number of small and a couple larger concerns with the work as presented, specifically the extent to which the authors use existing data on Bos taurus taurus, since they have a paragraph in the Introduction to breed comparisons. There is also apparently a Method section missing, as well as some minor editorial suggestions.

Scientific issues and questions.

For a paper that discusses a cross-breed comparison would be valuable to understand the differences between topical and other cattle is important (l. 73-79), I was expecting a comparison of the accessible regions between taurus and indicine. There has been at least liver data available for some time (2019, PMID: 31884969), as well as all of the three tissues analyzed here (2020, PMID: 33028202), all in Bos taurus so a direct comparison would have been possible. Potentially linking to the selective sweeps overlap would be an interesting story, greatly increasing the value of the reported data herein, and would increase the interest of the work for the Genome Biology audience. I also did not find a Methods section on the selective sweeps analysis.

R: Thank you for the suggestion which, in our view, indeed greatly increased the relevance of our results. We added the analysis as recommended using data from (2020, PMID: 33028202) and making sure all relevant methods description was added to the manuscript, including the precedence of the selective sweeps data.

Conclusions section- Certainly you have new data, I am unclear what "new insights" were revealed about "tissue-specificity of gene regulation"- please describe. Further, I do not know what "transcriptional control alphabet specific to the bovine indicine lineage" is; please expand, especially as you indicate in the next sentence that this is different from the "finding of open chromatin regions overlapping with signatures of selection". Since there was no detailed comparison across breeds, I don't see how you can talk about finding anything specific to indicine related to transcriptional control.

R: We certainly understand the Reviews’ point and for that reason, we added the comparison between indicus and taurus data and put the selective sweeps analysis in that context. We are confident that the new results and discussion will address the reviewers’ concerns.

Figure 2A. The explanation for the unusual pattern of ATAC-seq reads just for hypothalamus was unsatisfying. Is it possible that this is caused by a difference in 5' end/ promoter usage affecting annotation in hypothalamus?

R: The reviewer is right, and we apologize for our mistake in the analysis. The unusual pattern for hypothalamus-specific peaks was due to the method used to identify those peaks which originally enriched flanking peak regions rather than the actual peak. We redid the analyses making sure a whole peak was considered TS. The adjustment removed the unusual patterns without major effect in the overall results and discussion that was already proposed.

Minor issues

l. 180- "65% were associated with differential open chromatin regions" First, was this a statistically significant association? Second, was the direction of the ratio of DE of the gene the same direction of the Differential accessibility? I don't see that in Suppl Table 5.

R: In accordance with adjustments made to address Reviewers 1 concerns, both differential peaks and differential expression analysis were removed, therefore, this question is no longer applicable.

l. 196 Is this 22% statistically significant?

R: Our intention with the co-expression network was rather than generate a statistical value of significance for “validated” co-expressed target, to overlap different sources of information to identify potential targets with high confusability. ATAC-seq and RNA-seq data were not generated from the same samples and, therefore, not necessary completely overlap. At the same time, predicting target genes based on human data have its limitations. Intersections between analysis are a way to indent more accurate results.

l. 224 and 227- similarly to the comments for l. 180/196, what does "associated" mean, and was this overlap statistically significant?

R: These sentences and related analysis were removed from the manuscript.

L 252-255. This is a useful observation, but also shows the weakness in assuming that "nearest gene" to the peak is the target gene.

R: Indeed, there is a weakness in associating peaks to the nearest gene, particularly if the peaks fall in intergenic regions. That is the case of most indicine-specific peaks to which we can’t confidently attribute biological meaning because of the uncertainty of the true targets. Thanks for bringing that out, we included in the discussion.

l. 267-276. Yes, but it is also true that the liver has quite a few immune-centriccell types, including Kupfer cells, T cells, and NK cells and there doesn't have to be an "immune response" in liver to predict that whole-tissue liver samples such as these could show open chromatin at genes annotated for immunity.

R: True, thanks for the insight, we adjusted the discussion accordingly although in this reviewed version no terms directly related to immune response were significantly enriched.

Small editorial items:

Figure 1C- I didn't find a Methods description for this work. Also a typo in this part of the figure legend.

R: Typo was fixed, and methods description was improved.

l. 177 where should be were R: Edited.

l. 186- Define NES in text.

R: NES was defined at its first appearance in the text. It is defined again in the methods section where the term is explained in detail.

l. 248: I think you want to say "up to a few kilobases from a TSS" since by definition a promoter is directly adjacent to the TSS.

R: That is right. Adjusted as recommended.

l. 374- please describe the criteria used to define (identify as corresponding) peaks as overlapping across tissues.

R: As we revised the manuscript, and based on comments of Reviewer 1, we realized that calling regions of overlapping peaks between the three tissues as “constitutive peaks” was misleading. We changed for “constitutive regions” and provided a better explanation in the methods section of how we defined these regions. “…bedtools multiIntersectBed [25] was used to identify overlapping regions across the three tissues. Although a perfect overlap of peaks from different tissues is unlikely, regions of overlap can be of biological significance and will be referred to as constitutive regions.”

**Second round of review**

**Reviewer 1**

This is a revised version of the manuscript about a chromatin accessibility study in Bos indicus cattle, with ATAC-seq data from liver, muscle and hypothalamus.
Globally, the authors did a good job to address the issues that were raised. Methods are better described. The technical mistake to define peak specificity was acknowledged and properly fixed. The subsequent analyses and results have been updated. The differential analysis attempt was removed.
The new way of defining consensus and tissue-specific (TS) peaks makes sense. Obviously, this does not let many peaks left (from 286K to 12K consensus to 2K TS in liver for instance is quite a drastic reduction) but subsequent results are still relevant. Since the data is available anyone can try alternative specificity thresholds.

The new part about the indicus/taurus comparison is interesting. I am not convinced by the comments on the differences between the plots in Figure 5A though.
l 231-234: "Clearly, the higher number of peaks in hypothalamus identified in our study in comparison with the B. taurus data... with caution"
and
l 354-356: "In our study, indicine-specific ... due to the large difference in identified peaks in both datasets"
This is not specific to hypothalamus. About the same difference between indicus vs taurus peak numbers is actually observed in muscle for instance, but the other way around: more peaks in the taurus dataset than in the indicus one. One might expect a symmetrical Figure 5A pattern using Bos taurus specific peaks, with a TSS enrichment for MUS and LIV and a flat profile at TSS for HYP. A possible explanation could be that the dataset with more peaks is likely to cover most of the common promoters, and removing them to identify peaks that are specific to the other group might mostly reveal group-specific regulators (enhancers and silencers). As the authors rightly pointed out in the discussion, those are less conserved than promoter sites.
Anyway, the provided explanation does not sound totally satisfying to me. Generating the corresponding distributions for the taurus-specific peaks could clarify the discrepancy observed in figure 5A. There might be better hypotheses though.

Minor
l.166: Figure 4A => 3A
l.188 and 193: The word significant requires to precise p-value and statistical test
l.189: Figure 5 => Figure 4
l.279 change => changes?
l.330 "Admittedly, our definition of tissue-specific peaks is limited as we are only comparing three tissues...". This is a highly relevant and important statement (just a comment).
l. 356: in identified peaks in both datasets => between datasets?

**Reviewer 2**

Responses were appropriate and the additional analysis using Bos Taurus data was appreciated. There are minor typographical errors in the new sections that I trust will be caught by journal staff.
No further concerns.

**Authors’ response to reviewers**

Reviewer reports:

Reviewer #2: Responses were appropriate and the additional analysis using Bos Taurus data was appreciated. There are minor typographical errors in the new sections that I trust will be caught by journal staff.

No further concerns.

R: Minor typographical errors were fixed

Reviewer #1:

The new part about the indicus/taurus comparison is interesting. I am not convinced by the comments on the differences between the plots in Figure 5A though.

l 231-234: "Clearly, the higher number of peaks in hypothalamus identified in our study in comparison with the B. taurus data... with caution"

and

l 354-356: "In our study, indicine-specific ... due to the large difference in identified peaks in both datasets"

This is not specific to hypothalamus. About the same difference between indicus vs taurus peak numbers is actually observed in muscle for instance, but the other way around: more peaks in the taurus dataset than in the indicus one. One might expect a symmetrical Figure 5A pattern using Bos taurus specific peaks, with a TSS enrichment for MUS and LIV and a flat profile at TSS for HYP.

A possible explanation could be that the dataset with more peaks is likely to cover most of the common promoters and removing them to identify peaks that are specific to the other group might mostly reveal group-specific regulators (enhancers and silencers).

As the authors rightly pointed out in the discussion, those are less conserved than promoter sites.

Anyway, the provided explanation does not sound totally satisfying to me. Generating the corresponding distributions for the taurus-specific peaks could clarify the discrepancy observed in figure 5A. There might be better hypotheses though.

R: Yes, the Reviewer is right about the symmetry of the analysis when considering indicus and taurus. However, for the purpose of defining indicus-specific peaks, the higher number of muscle peaks in taurus only increases our confidence that we are indeed left with indicus-specific peaks when comparing the two datasets. Conversely, the low number of peaks identified in taurus hypothalamus data decrease our confidence that we are left with only indicus-specific peaks. The depletion of species- specific peaks around the TSS is reported in the literature, so it makes sense that the same pattern is observed when comparing sub-species.

We agree that “the dataset with more peaks is likely to cover most of the common promoters and removing them to identify peaks that are specific to the other group might mostly reveal group-specific regulators (enhancers and silencers).” That was exactly our intention, to identify group-specific regulators which are expected to fall within enhancers and silencers rather than promoter regions.

Generating the corresponding distributions for the taurus-specific peaks could also be interesting, but it would extend the discussion out of the intents of this work, which is focused on indicus cattle. Moreover, following the Editor’s instructions, no extra analysis will be added at this point.

Minor

l.166: Figure 4A => 3A R: Fixed

l.188 and 193: The word significant requires to precise p-value and statistical test R: Indeed. However, PCIT uses a relative approach to establish significant co-expression based on analyzing all possible trios of genes, thus, no hard threshold is established. From the abstract of the original paper “Results show that, as opposed to the constant cutoff approach commonly used in the literature, the PCIT algorithm can identify and allow for more moderate, yet not less significant, estimates of correlation

(r) to still establish a connection in the GCN. We show that PCIT is more sensitive than established methods and capable of detecting functionally validated gene-gene interactions coming from absolute r values as low as 0.3. These bona fide associations, which often relate to genes with low variation in expression patterns, are beyond the detection limits of conventional fixed-threshold methods and would be overlooked by studies relying on those methods.”

l.189: Figure 5 => Figure 4 R: Fixed

l.279 change => changes?

R: Edited as recommended

l.330 "Admittedly, our definition of tissue-specific peaks is limited as we are only comparing three tissues...". This is a highly relevant and important statement (just a comment).

R: We agree

l. 356: in identified peaks in both datasets => between datasets? R: Edited as recommended
